# Comparison of machine learning algorithms to predict clinically significant prostate cancer of the peripheral zone with multiparametric MRI using clinical assessment categories and radiomic features

**DOI:** 10.1007/s00330-020-07064-5

**Published:** 2020-07-16

**Authors:** Simon Bernatz, Jörg Ackermann, Philipp Mandel, Benjamin Kaltenbach, Yauheniya Zhdanovich, Patrick N. Harter, Claudia Döring, Renate Hammerstingl, Boris Bodelle, Kevin Smith, Andreas Bucher, Moritz Albrecht, Nicolas Rosbach, Lajos Basten, Ibrahim Yel, Mike Wenzel, Katrin Bankov, Ina Koch, Felix K.-H. Chun, Jens Köllermann, Peter J. Wild, Thomas J. Vogl

**Affiliations:** 1 Department of Diagnostic and Interventional Radiology, University Hospital Frankfurt, Theodor-Stern-Kai 7, 60590 Frankfurt am Main, Germany; 2 Dr. Senckenberg Institute for Pathology, University Hospital Frankfurt, Frankfurt am Main, Germany; 3 Frankfurt Cancer Institute, Goethe University, Frankfurt am Main, Germany; 4 Department of Molecular Bioinformatics, Institute of Computer Science, Johann Wolfgang Goethe-University, Frankfurt am Main, Germany; 5 Department of Urology, University Hospital Frankfurt, Frankfurt am Main, Germany; 6 Neurological Institute (Edinger Institute), University Hospital, Goethe University Frankfurt am Main, Frankfurt am Main, Germany; 7 German Cancer Consortium (DKTK) Partner Site Frankfurt/Mainz, Frankfurt am Main, Germany; 8 German Cancer Research Center (DKFZ), Heidelberg, Germany

**Keywords:** Prostate cancer, Multiparametric MRI, Machine learning, Artificial intelligence, Radiomics

## Abstract

**Objectives:**

To analyze the performance of radiological assessment categories and quantitative computational analysis of apparent diffusion coefficient (ADC) maps using variant machine learning algorithms to differentiate clinically significant versus insignificant prostate cancer (PCa).

**Methods:**

Retrospectively, 73 patients were included in the study. The patients (mean age, 66.3 ± 7.6 years) were examined with multiparametric MRI (mpMRI) prior to radical prostatectomy (*n* = 33) or targeted biopsy (*n* = 40). The index lesion was annotated in MRI ADC and the equivalent histologic slides according to the highest Gleason Grade Group (GrG). Volumes of interest (VOIs) were determined for each lesion and normal-appearing peripheral zone. VOIs were processed by radiomic analysis. For the classification of lesions according to their clinical significance (GrG ≥ 3), principal component (PC) analysis, univariate analysis (UA) with consecutive support vector machines, neural networks, and random forest analysis were performed.

**Results:**

PC analysis discriminated between benign and malignant prostate tissue. PC evaluation yielded no stratification of PCa lesions according to their clinical significance, but UA revealed differences in clinical assessment categories and radiomic features. We trained three classification models with fifteen feature subsets. We identified a subset of shape features which improved the diagnostic accuracy of the clinical assessment categories (maximum increase in diagnostic accuracy ΔAUC = + 0.05, *p* < 0.001) while also identifying combinations of features and models which reduced overall accuracy.

**Conclusions:**

The impact of radiomic features to differentiate PCa lesions according to their clinical significance remains controversial. It depends on feature selection and the employed machine learning algorithms. It can result in improvement or reduction of diagnostic performance.

**Key Points:**

*• Quantitative imaging features differ between normal and malignant tissue of the peripheral zone in prostate cancer.*

*• Radiomic feature analysis of clinical routine multiparametric MRI has the potential to improve the stratification of clinically significant versus insignificant prostate cancer lesions in the peripheral zone.*

*• Certain combinations of standard multiparametric MRI reporting and assessment categories with feature subsets and machine learning algorithms reduced the diagnostic performance over standard clinical assessment categories alone.*

**Electronic supplementary material:**

The online version of this article (10.1007/s00330-020-07064-5) contains supplementary material, which is available to authorized users.

## Introduction

The diagnosis of prostate cancer (PCa) must be confirmed by tumor tissue [1, 2]. Magnetic resonance imaging (MRI)–guided biopsies or ultrasonography (US)-/MRI-fusion biopsies can improve the detection rate of PCa [1, 3–5]. Multiparametric MRI (mpMRI) improves patient selection for biopsy and may reduce the amount of unnecessary invasive workup [4]. Even with image guidance, sampling bias represents a key challenge as confirmation of diagnosis is compromised by multifocality and the high degree of temporal and spatial intratumoral heterogeneity [6–9]. The sampling bias is problematic as the risk group influences the therapeutic approach [1, 4, 8, 10]. Definition of clinically significant PCa is a challenging dynamic process with ongoing debates [10–13]. Patients with Gleason Grade Group (GrG) ≤ 2 have a much better prognosis than those with GrG ≥ 3 [11, 12]. Furthermore, patients with GrG ≤ 2 may be feasible for active surveillance or ablative therapies [13]. There is a high need to optimize non-invasive risk stratification [14]. mpMRI is the basis of the Prostate Imaging Reporting and Data System (PI-RADS), a standardized protocol for acquisition, examination, and reporting [3, 15]. As opposed to the reader-dependent subjective PI-RADS [3, 15, 16], radiomic analyses represent another strategy to evaluate PCa in a quantitative and computational manner beyond visual perception [9, 17, 18]. The ability of radiomics to support diagnostic decision-making has been shown in numerous cancer entities [3, 9, 17, 18]. Yet, the understanding of suitable features and classification algorithms is still limited [3, 19, 20]. Bonekamp et al. have revealed an improved prediction to differentiate GrG ≤ 1 against GrG ≥ 2 [21]. Apparent diffusion coefficient (ADC) has yielded the highest relevance to differentiate variant GrGs [21]. Numerous studies are being conducted to stratify the best working models for MRI-based PCa classification [3, 22–25]. The classification method has a strong impact on the variation in performance [19]. Yet, the question has not been addressed to what extend specific feature and prediction model effect the diagnostic performance to differentiate GrG ≤ 2 against GrG ≥ 3 [3, 22–26]. The purpose of this study was to evaluate the application of the clinical assessment categories PI-RADS and ADC-derived radiomic features to build and compare three prediction models and to analyze their influence on the differentiation of clinically significant PCa.

## Materials and methods

### Patient population

**Fig. 1:**
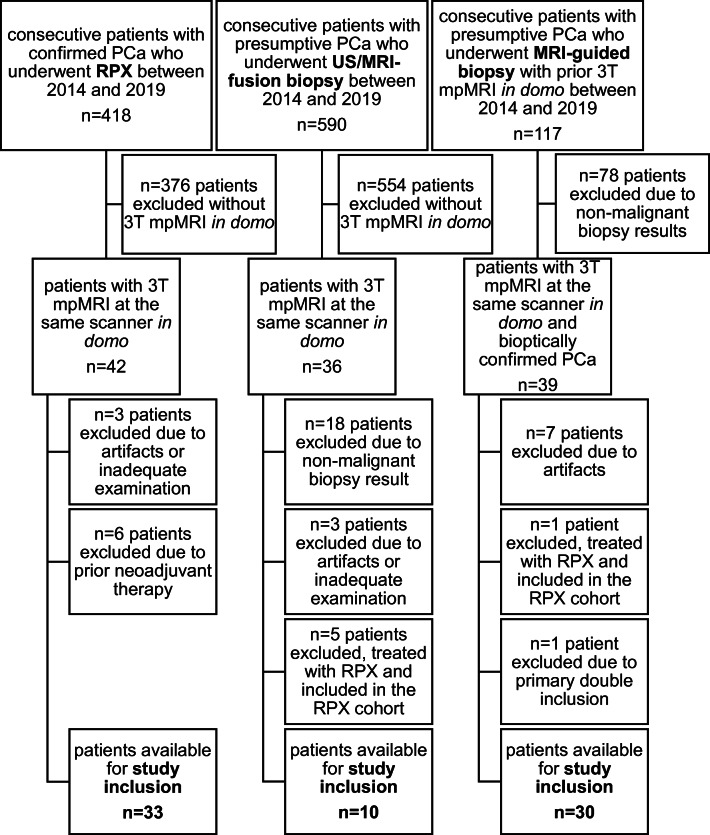
Flowchart of patient inclusion. In total, 73 patients were included into the final study, consisting of three distinct groups: radical prostatectomy (RPX), US-/MRI-fusion biopsy, MRI-guided biopsy. mpMRI, multiparametric magnetic resonance imaging; PCa, prostate cancer; *n*, absolute number; RPX, radical prostatectomy; T, tesla; US, ultrasonography

The study was approved by the institutional Review Board of the Ethical Committee at the University Hospital Frankfurt (project-number: 41/19). In total, 1125 patients were screened for study inclusion, examined between 2014 and 2019. Figure 1 shows the inclusion algorithm. Inclusion criteria were (a) targeted biopsy (US-/MRI-fusion biopsy, MRI-guided biopsy) or radical prostatectomy (RPX) in domo, (b) histologically confirmed PCa, and (c) imaging at the same 3-T (T) MRI scanner. Exclusion criteria were (a) incomplete/inadequate examination protocol, (b) artifacts on mpMRI images, and (c) neoadjuvant therapy with regressive changes. The median time from mpMRI to biopsy/RPX was 0 months with a maximum of 7 months. Table 1 summarizes the clinical and epidemiological characteristics.

**Table 1:** Clinical and epidemiological characteristics of included patients

Variable	Study cohort
Patients	73 (100)
Radical prostatectomy (RPX)	33 (45)
MRI-guided biopsy	30 (41)
MRI-/US-fusion biopsy	10 (14)
Median age at definite diagnosis (years)*	66 (35–83)
Median time (months)*, MRI to tissue (biopsy, RPX)	0 (0–7)
Prior biopsy with post-biopsy changes in T1w***	30 (41.1)
Mean PSA (ng/mL)**	12.14 (13.9; 15.8; 8.4) [NA: 16]
Localization (index lesion)	
PZ	66
PZ/AFS	7
Median number of intra-prostatic lesions*	2 (1–3)
PI-RADS, index lesion***	
3	8 (11)
4	26 (36)
5	39 (53)
Gleason score, index lesion***	
3 + 3	15 (21)
3 + 4	23 (32)
4 + 3	15 (21)
4 + 4	5 (7)
4 + 5	11 (15)
5 + 3	1 (1)
5 + 4	1 (1)
5 + 5	2 (3)
Gleason Grade Group, index lesion***	
1	15 (21)
2	23 (32)
3	15 (21)
4	6 (8)
5	14 (19)
Available, sufficient quality of sequences ***	
T2w	72 (99)
ADC	73 (100)
DCE	68 (93)
pTNM, RPX-cohort***	33 (100)
pT2a	2 (6)
pT2b	1 (3)
pT2c	13 (40)
pT3a	12 (36)
pT3b	5 (15)
pN0	29 (88)
pN1	2 (6)
pNX	2 (6)
pM0	31 (94)
pM1	1 (3)
pMX	1 (3)
pR0	24 (73)
pR1	8 (24)
pRX	1 (3)

If not otherwise depicted, the numbers without parenthesis depict absolute numbers. *Data in round parenthesis are the min/max values (interquartile range); **Data in round parenthesis are standard deviation and ± 95% confidence interval; ***Data in round parenthesis are relative values; Data in square parenthesis are not available values, excluded in the analysis; due to mathematical rounding, the summed relative values may differ slightly from 100. *ADC*, apparent diffusion coefficient; *AFS*, anterior fibromuscular stroma; *DCE*, dynamic contrast enhanced; *MRI*, magnetic resonance imaging; *NA*, not available; *PI-RADS*, Prostate Imaging Reporting and Data System; *PSA*, prostate-specific antigen; *PZ*, peripheral zone; *RPX*, radical prostatectomy; *T1w*, T1-weighted; *T2w*, T2-weighted; *US*, ultrasonography

### MR imaging acquisition and examination

**Fig. 2:**
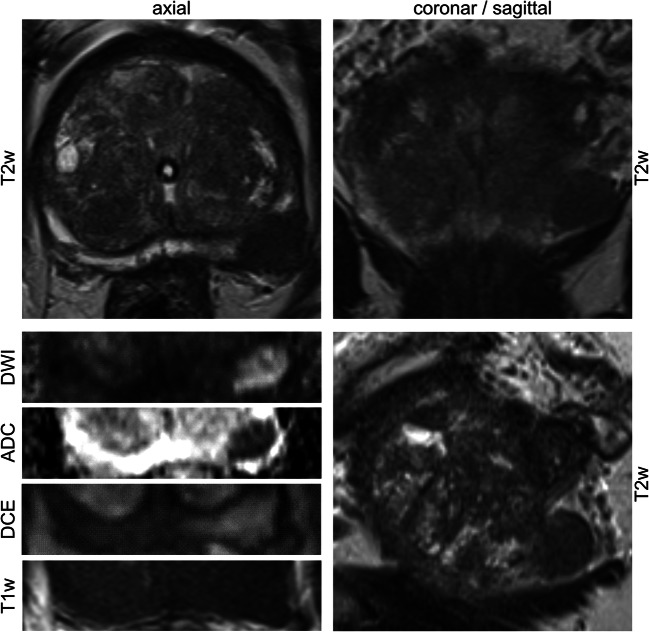
Example of a representative multiparametric MRI examination of the prostate. Multiparametric magnetic resonance imaging (mpMRI) examination consisting of anatomical (T2w, T2-weighted; T1w, T1-weighted) and functional (DWI, diffusion-weighted imaging; ADC, apparent diffusion coefficient; DCE, dynamic contrast-enhanced) images. Anatomical T2w images were acquired in multiparametric axial, sagittal, and coronal planes. Functional images and T1w images were acquired in axial plane. Typical characteristics of a malignant prostate cancer lesion of the left peripheral zone are shown of a 60-year-old patient who presented himself with a maximum prostate specific antigen level of 64 ng/mL. The respective Prostate Imaging Reporting and Data System category equaled 5 and histopathologic examination of the radical prostatectomy specimen revealed a Gleason Grade Group of 5

All examinations were performed on a single 3-T scanner in clinical routine with a standard 32-channel body coil (Magnetom Prisma^FIT^, Siemens Healthineers) and built-in spine phased-array coil. MRI examinations were performed according to the European Society of Urogenital Radiology (ESUR) guidelines including T1-weighted (T1w), T2-weighted (T2w), diffusion-weighted imaging (DWI), ADC, and dynamic contrast-enhanced (DCE) sequences. Figure 2 shows an example of a typical mpMRI of the prostate. All MRI examinations were primarily performed and read by an experienced radiologist and confirmed by a board-certified radiologist. Each prostatic lesion was categorized by applying PI-RADS v2 [15]. For the consecutive radiomics analysis, the T2w, ADC map (derived from DWI with *b* value of 0/1000 or 50/1000 s/mm^2^ (*n* = 64; *n* = 9)), and DCE MR images were extracted in “Digital Imaging and Communications in Medicine” (DICOM) format. Table 2 depicts the acquisition parameters in detail.

**Table 2:** Multiparametric MRI sequence parameters

Sequence parameter	T2 TSE, mean (*n* = 72)	ADC, mean (*n* = 73)	DCE, mean (*n* = 68)
TR (ms)	7511.1	3395.9	5.08
TE (ms)	104.3	59.8	1.8
Averages	2.3	7.9	1.0
Flip angle (°)	157.0	90.0	13.5
FOV (mm^2^)	202.8 × 202.8	94.8 × 203.2	257.9 × 259.0
Matrix (px^2^)	297.2 × 345.9	52.9 × 149.0	153.1 × 192.0
Bandwidth (Hz)	202.0	1203.7	260.0
Slice thickness (mm)	3.1	3.0	3.5
Orientation	transversal	transversal	transversal
*b*0_1000 (*n*)		64	
*b*50_1000 (*n*)		9	

Sequence parameter for all patients included into the study (*N* = 73). *ADC*, apparent diffusion coefficient; *DCE*, dynamic contrast enhanced; *FOV*, field of view; *n*, absolute number; *TE*, echo time; *TR*, repetition time; *TSE*, turbo-spin-echo

### MRI segmentation

**Fig. 3:**
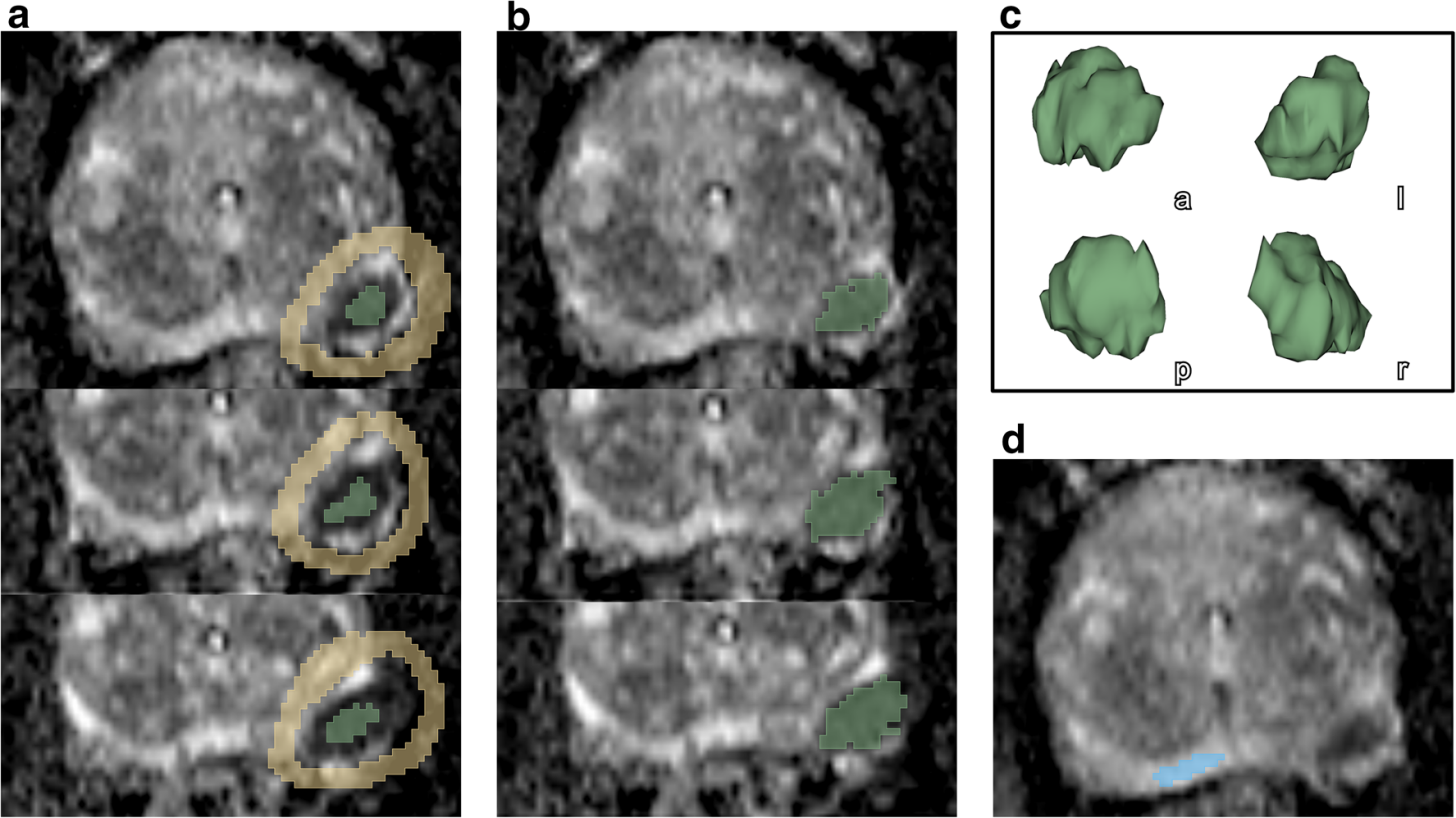
Habitat definition and volume rendering. After manual definition of tumor-bearing area (**a**, green) and surrounding normal-appearing tissue (**a**, orange) in representative image series, a semi-automatic grow from seeds algorithm was applied (**b**) obtaining a three-dimensional habitat of the whole volume of interest (VOI) with exemplary volume rendering from four points of view being shown (**c**: (a) anterior; (p) posterior; (l) left; (r) right). For each specimen, normal-appearing peripheral zone was delineated by manual VOI placement (**d**, blue)

In direct correlation to an institutional workstation and the respective clinical reports, the extracted series were re-reviewed by one investigator (S.B. with 6 months of experience and special training in uropathological imaging) under the supervision of a board-certified radiologist (T.J.V., B.B. with 18 or 10 years of experience in uropathological imaging) using the open-source 3D Slicer software platform (http://slicer.org, version 4.9.0) [27, 28] with consecutive VOI placement. T2w and DCE images were applied within the 3D Slicer computing platform to visually correlate for lesion definition in the ADC maps. We performed the consecutive quantitative analysis on ADC in concordance with the study of Bonekamp et al [21]. Figure 3 depicts the standardized, semi-automatic algorithm of the VOI annotation with volume renderings. Manual parts were performed by applying the paint tool of the segment editor. The benign tissue VOI was manually defined for each patient by delineating normal-appearing tissue of the peripheral zone (PZ) in maximum distance to the index lesion. The whole-habitat VOI was generated by a semi-automatic grow from seeds algorithm with subsequent manual correction of artifacts with a brush-erase tool [28–30].

### Feature extraction

The Imaging Biomarkers Standardization Initiative (IBSI) does currently not cover image preprocessing [31]. There is no consensus with regard to preprocessing or normalization algorithms [32, 33]. Digital image manipulation may hamper reproducibility and it is proposed to report all processing details [32, 33]. Therefore, to ensure best transparency and comparability, we have limited image manipulation to the minimum by using unchanged, naïve image data, in accordance with the algorithm performed by Aerts et al [18]. We employed the open-source package PyRadiomics [34] which gains increasing establishment as reference standard for radiomics analysis [32, 34] as extension within 3D Slicer [27, 28]. From seven feature classes, all standard features were extracted: first order statistics, shape-based, Gray Level Co-occurrence Matrix (GLCM), Gray Level Run Length Matrix (GLRLM), Gray Level Size Zone Matrix (GLSZM), Gray Level Dependence Matrix (GLDM), Neighboring Gray Tone Difference Matrix (NGTDM) leading to 105 features/VOIs in ADC (http://pyradiomics.readthedocs.io) [34]. We extracted the features with the default settings within PyRadiomics, i.e., no resampling or filtering, no wavelet-based features, bin width 25, and enforced symmetrical GLCM (http://pyradiomics.readthedocs.io) [18, 32, 34].

### Tissue specimen and MRI concordance

Tissue specimen was analyzed in the Dr. Senckenberg Institute of Pathology (SIP), Goethe University Hospital, Frankfurt am Main. The index lesion (and benign VOI) was determined by correlating the mpMRI with the assessment of the highest GrG (and prostatic tissue without evidence of malignancy) in the pathological report. J.K. (uropathologist with 10 years of experience) annotated the RPX specimen to match the index lesion (and the benign VOI) of the MRI examination (highest PI-RADS or no sign of malignancy). If the pathologic assessment did not match the index lesion (highest GrG ≠ annotated mpMRI index lesion) or benign VOI, images were reexamined and reevaluated in direct correlation to the histopathologic slides. We considered the histopathological results as ground-truth. If a patient had bioptic and RPX tissues available, we assessed the RPX tissue. For this study, we defined GrG ≥ 3 as clinically significant PCa.

### Evaluation approach

We applied ADC-derived radiomic features and the PI-RADS categories in relation to the GrGs. We performed two-dimensional principal component (PC) analysis and univariate analysis (UA) to analyze benign versus malignant tissue as well as insignificant versus significant PCa. In our cohort, all PCa lesions with PI-RADS = 3 (*n* = 8) were clinically insignificant and were excluded for the following analysis. We computed significance values (*p* values) for all features by UA. We chose the top four features with the lowest *p* value of the two-tailed Student’s *t* test and Wilcoxon test as being the most stable for further evaluation steps [19]. We performed multivariate analysis of correlation to correct for collinearity and reduce features. Reduction of features is a common method and reduces the risk of overfitting [17, 35]. To assess the predictive power, we generated receiver operating characteristic (ROC) curves of support vector machines (SVM), neural networks (NNs), and random forest (RF) analysis for 15 combinations of the predictor subsets. We employed 100-fold cross-validation to evaluate the performance of the prediction [36]. In each run, we randomly drew 70% of the samples for training and validated the classifier with the remaining independent 30% of the sample data (modified as described previously [37]). We obtained the area under the curve (AUC) and assessed differences in the prediction power of the models by the application of a two-tailed Student’s *t* test of the 100 values of AUC. The machine learning algorithms were conducted in Python 3.7 using the open-source scikit-learn 0.21.3 packages SVC for SVM, MLPClassifier for NNs and the RandomForestClassifier for RF analysis with prior normalization of features using StandardScaler (https://scikit-learn.org/) [38]. We conducted further statistical analyses with Prism 6.0 (GraphPad software) and JMP 14 (SAS). We indicated the significant values as follows: *p < 0.05; **p < 0.01; ***p < 0.001. A flowchart of the methodologic study design is shown in Supplementary Document 1.

## Results

### Radiomic features differ in benign versus malignant prostate tissue

**Fig. 4:**
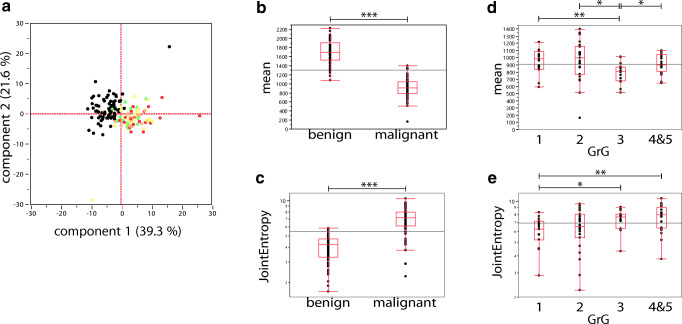
Clear clustering of benign versus malignant tissue with radiomic features revealing significant differences in different Gleason Grade Groups. In **a**, the principal component analysis of all radiomic features, except shape features, is shown. Index lesions are color-coded according to the respective Gleason Grade Group (GrG; 1/2/3/4&5, green/yellow/orange/red) and normal-appearing peripheral zone is depicted in black. Prostate tissue shows clear clustering in benign and malignant volumes of interest, whereas different GrGs do not reveal distinguishable subcluster (**a**). Box-Whisker plots for the features mean (**b**) and joint entropy (**c**) are shown with significant differences using two-tailed Student’s *t* test. In **d** and **e**, Box-Whisker plots reveal significant differences in specific GrGs, exemplarily shown for mean (**d**) and joint entropy (**e**) using nonparametric comparison for each pair/Wilcoxon method

PC analysis clustered benign peripheral zone (black) against the malignant index lesion (colors, Fig. 4a). Subclusters of variant GrGs were not visualized (green/yellow/orange/red, PI-RADS 1/2/3/4&5; Fig. 4a). Lower mean ADC values were revealed for malignant lesions (*p* < 0.001) which is in concordance with PI-RADS [15] (Fig. 4b). Various radiomic features differed comparing benign versus malignant prostate tissue, with JointEntropy (JE, *p* < 0.001) being depicted exemplarily (Fig. 4c; for all features, see Supplementary Document 2). We observed differences in a subset of radiomic features when comparing different GrGs with mean (p(GrG 1/3, 2/3, 3/4&5) = 0.007, 0.012, 0.016) and JE (p(GrG 1/3, 1/4&5) = 0.018, 0.008) being depicted exemplarily (Fig. 4d, e; for all features, see Supplementary Document 3).

### Clinically significant PCa lesions of high-risk patients revealed differences in radiomic features

**Fig. 5:**
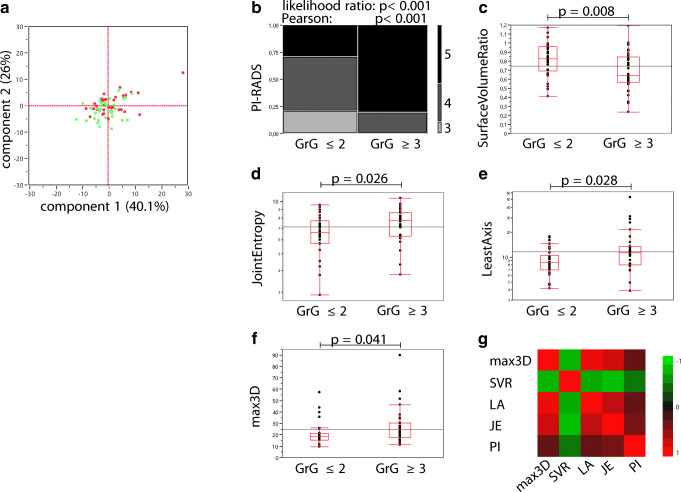
Radiomic features and the clinical assessment categories reveal significant differences in clinically significant versus insignificant prostate cancer. Two-dimensional principal component analysis of all radiomic features of the index lesions shows random distribution of significant (red) and insignificant (green) prostate cancer (PCa, **a**). In **b**, the contingency table of the Prostate Imaging Reporting and Data System (PI-RADS) categories related to clinically significant (GrG ≥ 3) and insignificant (GrG ≤ 2) PCa is shown. Box-Whisker plots for the top four radiomic features to differentiate clinically significant PCa are shown (**c**, SVR, surface to volume ratio; **d**, JE, joint entropy; **e**, LA, least axis; **f**, max3D, maximum 3D diameter). **g** The color map on correlation of max3D, SVR, LA, JE, and PI-RADS, starting at green for negative (− 1) correlation and moving to red as the correlation approaches 1. Detailed data of the multivariate correlation is depicted in Table 3. Statistical analysis was performed using two-tailed Student’s *t* test (**c**–**f**), likelihood ratio/Pearson test (**b**), or multivariate measurements of correlations (**g**). In **c**–**g**, the index lesions with PI-RADS = 3 (*n* = 8) were excluded. Clinically significant PCa was defined as GrG ≥ 3, with GrG ≤ 2 being considered as clinically insignificant PCa

PC analysis achieved no stratification of the index lesion according to its clinical significance (Fig. 5a). The samples were distributed randomly (Fig. 5a; dots: green, insignificant PCa; red, significant PCa). The PI-RADS assessment categories were differently distributed in clinically significant PCa (Fig. 5b, likelihood ratio and Pearson *p* < 0.001). In our cohort, all PI-RADS = 3 lesions were found to be clinically insignificant (Fig. 5b). To avoid overfitting and redundancy of the prediction models, PI-RADS = 3 lesions (*n* = 8) were excluded for further analysis. Next, the top four features of an iterative Wilcoxon and two-tailed Student’s *t* test were determined (Fig. 5 c–f depicts data of the iterative Student’s *t* test; for all data, see Supplementary Document 4). The prioritized features surface to volume ratio (SVR), JE, least axis (LA), and maximum 3D diameter (max3D) showed lowest *p* values (*p* = 0.008, *p* = 0.026, *p* = 0.028, *p* = 0.041) stratifying the clinical significance of the examined lesions (Fig. 5c–f). High correlation was revealed using multivariate testing (Fig. 5g; Table 3). Therefore, for the consecutive prediction models, internal combinations of the highly correlated shape features (max3D, SVR, LA) were excluded (Table 3). Though correlation was revealed for JE with the shape features (Fig. 5g; Table 3), we performed subsets of combinations for prediction model generation as JE represents a member of the different feature class, GLCM. We could identify PI-RADS as the most independent variable (Fig. 5g; Table 3; |correlation| < 0.47 for each feature).

**Table 3:** Matrix of correlations of the features used for classifier building

	max3D	SVR	LA	JE	PI
max3D	1.000	− 0.778	0.930	0.812	0.339
SVR	− 0.778	1.000	− 0.726	− 0.824	− 0.467
LA	0.930	− 0.726	1.000	0.740	0.334
JE	0.812	− 0.824	0.740	1.000	0.416
PI	0.339	− 0.467	0.334	0.416	1.000

Multivariate measurements of correlations of the selected radiomic features and the clinical assessment category PI-RADS (Prostate Imaging Reporting and Data System) used for the generation of the prediction models. *max3D*, maximum 3D diameter; *SVR*, surface to volume ratio; *LA*, least axis; *JE*, joint entropy; *PI*, PI-RADS

### The prediction performance of clinically significant PCa lesions depends on the selected feature subset and machine learning algorithm

**Fig. 6:**
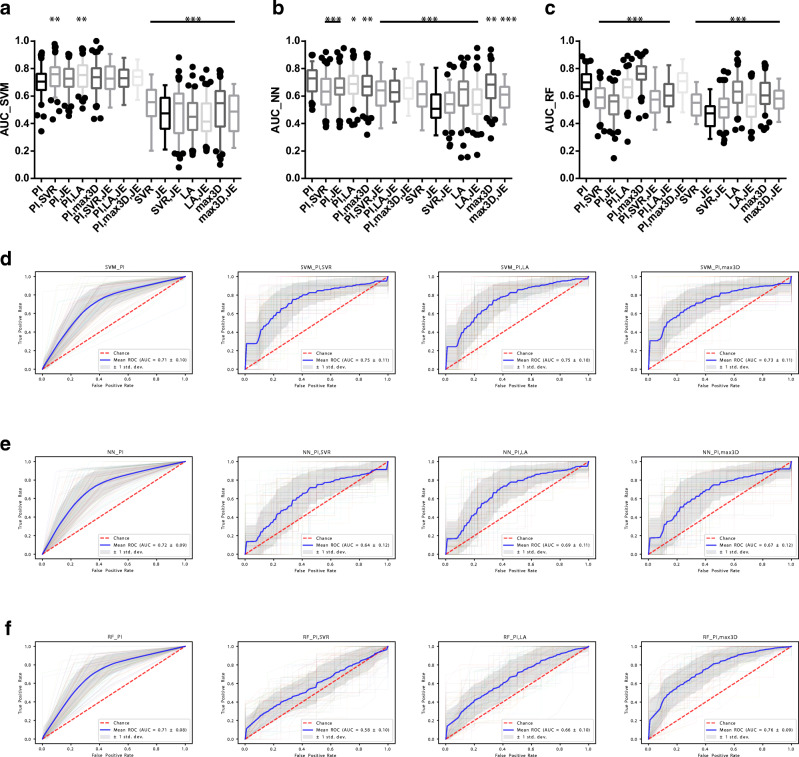
The addition of radiomic features to the clinical routine assessment categories using different machine learning algorithms has highly variable effect on the discriminative accuracy to predict significant versus insignificant PCa. Analysis of prediction performance for clinically significant prostate cancer (PCa) using 15 variant feature subsets with 3 different machine learning algorithms. The subsets were based on PI-RADS (PI) and the top four quantitative imaging features surface to volume ratio (SVR), joint entropy (JE), least axis (LA), or maximum 3D diameter (max3D). The prediction models were built using support vector machine (SVM, **a**, **d**), neural network (NN, **b**, **e**), or random forest (RF, **c**, **f**) algorithms. **a**–**c** The Box-Whisker plots with 5–95% percentile for each machine learning algorithm obtained by 100-fold cross-validation experiments as depicted in detail in the “Materials and methods” section using the respective area under the receiver operator characteristics (ROC) curve to predict significant PCa. Asterisks relate to the analysis of PI against the respective subset as indicated (**a**–**c**). Significant differences to PI are depicted using two-tailed, unpaired Student’s *t* test (**a**–**c**). The respective images of the 100-fold cross-validated (colors) ROC curve analyses with the mean ROC curve (blue) are shown for each prediction model for PI and its combination with SVR, LA, or max3D (**e**, **f**). The adjacent gray area depicts ± one standard deviation (**e**, **f**). Shown are the results of the validation cohort with 30% holdback proportion, drawn at random. Patients with PI-RADS = 3 (*n* = 8) were excluded due to training/validation redundancy, to avoid overfitting and bias as the respective lesions were always insignificant PCa in the studied cohort (Fig. 5b). SVM was adapted for rbf-Kernel, *C* = 1 with probability = true. The NN consisted of 1 layer and 3 hidden nodes, maximum iteration of 100, logistic activator, and the lbfgs solver. For RF analysis, 20 estimators with random_state = 0 were specified

We trained three machine learning algorithms with 15 subsets of radiomic features and the clinical assessment category PI-RADS to predict the clinical significance of PCa lesions (Fig. 6). SVM, NN, and RF showed variable prediction performance comparing PI-RADS against variant feature subsets (Fig. 6a–c). PI-RADS was superior to all subsets of radiomic features alone in the prediction of clinical significance (Fig. 6a–c, *p* = 0.003 for max3D using NN and *p* < 0.001 for all other models). Variant combinations of PI-RADS with radiomic features improved or weakened the prediction performance dependent on the employed machine learning algorithm (Fig. 6a–c). Using SVM, the combination of PI-RADS with SVR or LA improved the prediction performance (*p* = 0.008, *p* = 0.002, ΔAUC = + 0.04; Fig. 6a, d). Using NN, each combination of PI-RADS with radiomic features weakened the predictive performance (Fig. 6b, e; highest decrease of AUC by adding SVR with *p* < 0.001, ΔAUC = − 0.08). Using RF, the additional application of max3D outperformed PI-RADS alone (*p* < 0.001, ΔAUC = + 0.05; Fig. 6b, f) establishing the best working model (Fig. 6). The combination of PI-RADS with max3D and JE did not improve the performance to PI-RADS alone (*p* = 0.27), whereas all other combinations even weakened the prediction performance (Fig. 6c, f; highest decrease of AUC by adding JE with *p* < 0.001, ΔAUC = − 0.17). Figure 6 d–f depicts the representative 100-fold cross-validated ROC curves with their AUC values for PI-RADS and its combination with the shape features SVR, LA, and 3Dmax.

## Discussion

In this study, we analyzed the effect of different subsets of radiomic features and the clinical assessment category PI-RADS on the predictive performance of three machine learning algorithms to stratify PCa of the PZ according to its clinical significance. We first demonstrated adequate VOI placement in concordance with the PI-RADS [15] assessment. Our data demonstrates that the integration of radiomic features using machine learning algorithms can positively or negatively influence the prediction performance for clinically significant PCa. The results emphasize the need to be cautious using radiomic machine learning strategies but also the potential of the features SVR, LA, and max3D to improve PI-RADS assessment categories.

Gleason Grading suffers from interobserver variance with the differentiation between GrG = 2 and GrG = 3 being especially challenging [39]. New decision support tools are critical to reduce over- and undertreatment [10, 11, 14, 39]. Qualitative mean ADC is part of the PI-RADS [15], but further features inherit independent data though not being part of the current assessment categories [3, 22, 23, 25, 40]. Radiomics ability to decipher biologic and prognostic parameters has been shown in numerous studies [3, 9, 17, 18]. In PCa, the mere detection of malignancy has been augmented to the assessment of aggressiveness up to genomic risk stratification biomarkers [3, 35, 40, 41]. In this context, ADC analysis seems to effect differentiation of PCa aggressiveness in particular [3, 21, 40, 42]. Bonekamp et al. have been able to show that mean ADC performs equally as complex machine learning approaches to differentiate GrG ≤ 1 versus GrG ≥ 2 [21]. Therefore, we focused on ADC to examine our patient cohort. Previous studies have demonstrated the ability of mpMRI and radiomics to differentiate variant tissue types to aid PCa diagnosis and our data supported this finding [23, 35, 43]. Nevertheless, this finding should not be overstated as it seems to be a logic consequence to the applied methodology of supervised VOI definition [21, 35, 44], demonstrating an appropriate VOI placement [21, 35, 44]. Highest methodological transparency, open-source software, and standardization are necessary to obtain valid results and to promote interdisciplinary research [32, 33, 45, 46]. Our study design aims to propose a feasible and reproducible step-by-step approach and usage of open-source software. For image processing and radiomic feature definition and extraction, we applied open-source software [28, 34], whereas numerous studies have applied house-built software, making repeatability nearly impossible [33]. Image preprocessing may alter the extracted values and may reduce reproducibility across datasets [32, 33]. Currently, no consensus exists regarding variant preprocessing settings [32, 33]. We reduced image manipulation to the minimum and performed no additional preprocessing. ADC as a quantitative value requires no normalization [21]. Consistent with Aerts et al, we applied PyRadiomics with default settings [18]. To reduce variability with regard to VOI segmentation, we applied the grow from seeds algorithm within 3D Slicer [27–30]. Parmar et al. demonstrated that UA Wilcoxon test–based feature selection with RF had the highest performance and data stability of radiomic applications [19]. Therefore, we performed UA feature selection by the Wilcoxon method and further applied multivariate measurement of correlation to handle collinearity [46]. We tested and compared variant machine learning algorithms as the choice of classification method is known to be of major importance regarding performance variation [19, 20]. Consistent with Parmar et al., our best working model was found using RF classification method [19].

Our study has limitations that warrant discussion. We examined retrospective data with subsequent patient enrollment; a selection bias cannot be ruled out. We employed tissue specimens, which were obtained using three variant techniques: RPX, US-/MRI-fusion biopsy, and MRI-guided biopsy with biopsy techniques may inherit sampling bias [4, 5, 7]. We did not include cancers of the transition zone. We applied clinical routine protocols and an analysis of more homogeneous data would have been preferable. Around 40% of the patients had prostatic tissue changes due to prior biopsy. Not biopsy-naïve patients may not be regarded to be outliers but an issue of clinical routine. We did include those patients to reduce the selection bias. With 73 patients, our study population was limited and a larger cohort might improve the significance of statistical analysis. In our cohort, all patients with PI-RADS lesions equaling three (*n* = 8) were found to have clinically insignificant PCa. Since these patients would bias the predictive models, they were excluded in the machine learning analyses, though this might limit the generalizability of the obtained results. By restricting patient inclusion to examinations from the same 3-T scanner, we ruled out an interscanner variability. Nevertheless, intrascanner variability might have altered our results as shown in a phantom study by Baeßler et al. [45]. The fact that our predictive subsets of PI-RADS with only one radiomic feature showed better performance compared with two features may be explained by the observation that increased dimensionality may lead to reduced discriminative power [3]. We have limited the analysis to three machine learning algorithms and cannot exclude that an unapplied algorithm might have shown variant results. We performed annotation of index lesions with supervision of board-certified radiologists (T.J.V., B.B.) as well as in direct correlation with an uropathologist (J.K.) and the pathology reports. Nevertheless, even targeted biopsies and RPX specimen have a residual uncertainty [4, 47].

In conclusion, our study underlines the potential of ADC-derived radiomic features of prostate mpMRI examinations to aid in the stratification of prostate cancer lesions according to their clinical significance. We emphasized the need to be cautious prior to applying computer-aided diagnostics as the predictive performance highly depends on feature and machine learning algorithm selection at worst even reducing clinical assessment performance. Non-invasive prediction models may have the potential to be part of decision support tools to aid clinicians in the selection of an adequate therapy, but we need to be cautious before translation into clinical routine.

## Electronic supplementary material

ESM 1 (PDF 54 kb)

ESM 2 (DOC 3513 kb)

ESM 3 (DOC 5388 kb)

ESM 4 (DOC 4497 kb)

## Abbreviations

ADC: Apparent diffusion coefficient
AFS: Anterior fibromuscular stroma
AUC: Area under the curve
CI: Confidence interval
DCE: Dynamic contrast-enhanced
DWI: Diffusion-weighted imaging
FOV: Field of view
GrG: Gleason Grade Group
JE: Joint entropy
LA: Least axis
max3D: Maximum 3D diameter
mpMRI: Multiparametric MRI
MRI: Magnetic resonance imaging
*n*: Absolute number
NA: Not available
NN: Neural network
PC: Principal component
PCa: Prostate cancer
PI: PI-RADS
PSA: Prostate-specific antigen
PZ: Peripheral zone
ROC: Receiver operating characteristics
RPX: Radical prostatectomy
SD: Standard deviation
SVR: Surface to volume ratio
T1w: T1-weighted
T2w: T2-weighted
TE: Echo time
TR: Repetition time
TSE: Turbo-spin-echo
UA: Univariate analysis
US: Ultrasonography
VOI: Volume of interest
