# Supplementary figures and images for: Comparison of machine learning algorithms to predict clinically significant prostate cancer of the peripheral zone with multiparametric MRI using clinical assessment categories and radiomic features
Source: Eur Radiol. 2020 Jul 16;30(12):6757–69. doi: 10.1007/s00330-020-07064-5 (PMC7599168; doi:10.1007/s00330-020-07064-5)

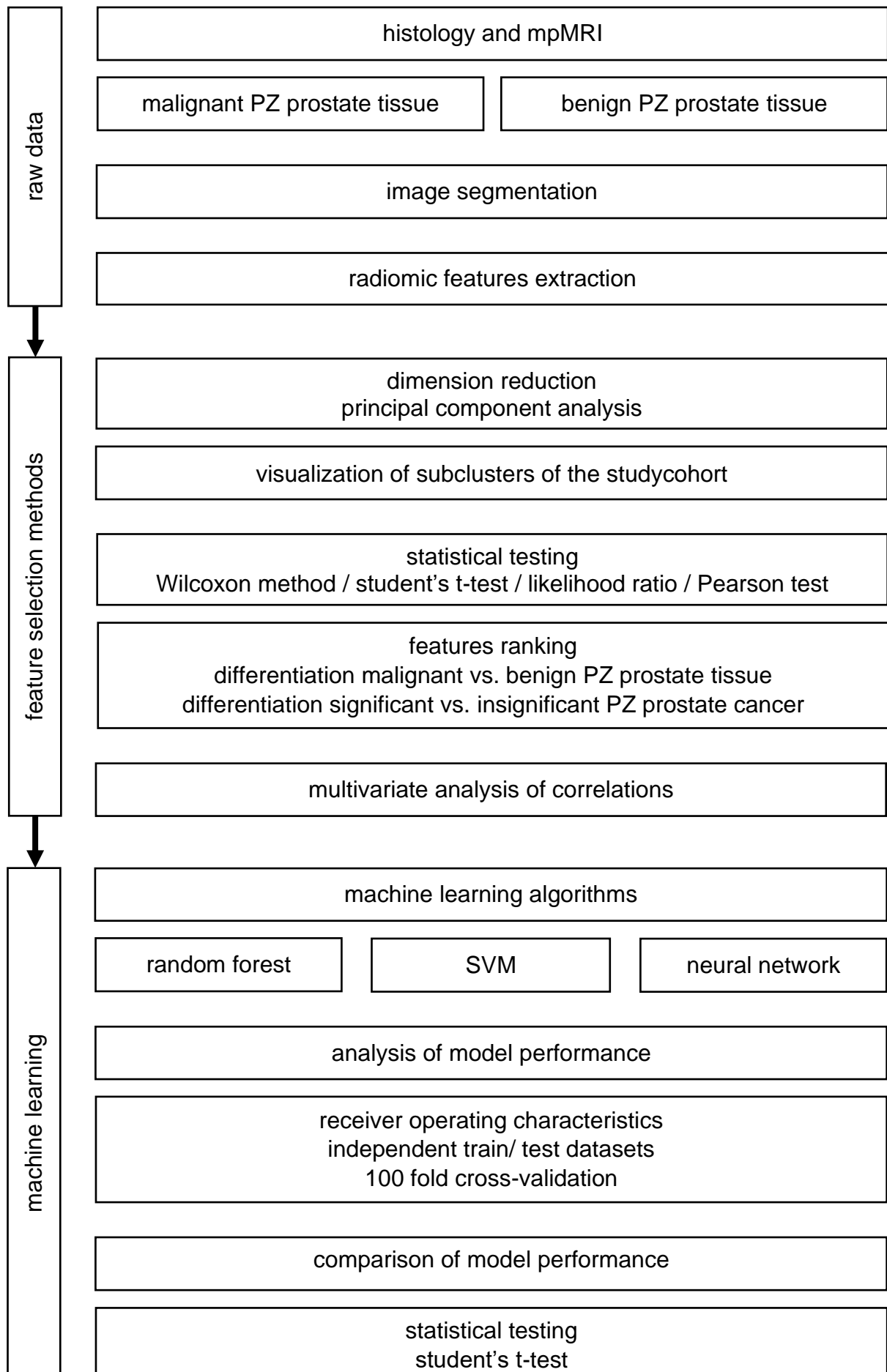

Supplement: Supplementary file 1 — (PDF 54 kb) [file 330_2020_7064_MOESM1_ESM.pdf]
