# Supplementary material for: Comparison of machine learning algorithms to predict clinically significant prostate cancer of the peripheral zone with multiparametric MRI using clinical assessment categories and radiomic features
Source: Eur Radiol. 2020 Jul 16;30(12):6757–69. doi: 10.1007/s00330-020-07064-5 (PMC7599168; doi:10.1007/s00330-020-07064-5)
Supplement: Supplementary file 2 — (DOC 3513 kb) [file 330_2020_7064_MOESM2_ESM.doc]

**Fit Group**

**Oneway Analysis of original-gldm-GrayLevelVariance By Type**

**Quantiles**

| **Level** | **Minimum** | **10%** | **25%** | **Median** | **75%** | **90%** | **Maximum** |
| --- | --- | --- | --- | --- | --- | --- | --- |
| benign | 3,668639 | 12,26111 | 16,38462 | 31,81 | 49,60754 | 75,22513 | 377,157 |
| malignant | 0,636487 | 26,38795 | 37,64416 | 52,39868 | 74,16914 | 120,3524 | 187,0951 |

**Oneway Anova**

**Summary of Fit**

| Rsquare | 0,056127 |
| --- | --- |
| Adj Rsquare | 0,049573 |
| Root Mean Square Error | 41,88374 |
| Mean of Response | 51,75296 |
| Observations (or Sum Wgts) | 146 |

**Pooled t Test**

malignant-benign

Assuming equal variances

| Difference | 20,2867 | t Ratio | 2,926255 |
| --- | --- | --- | --- |
| Std Err Dif | 6,9326 | DF | 144 |
| Upper CL Dif | 33,9896 | Prob > |t| | 0,0040* |
| Lower CL Dif | 6,5838 | Prob > t | 0,0020* |
| Confidence | 0,95 | Prob < t | 0,9980 |

**Analysis of Variance**

| **Source** | **DF** | **Sum of Squares** | **Mean Square** | **F Ratio** | **Prob > F** |
| --- | --- | --- | --- | --- | --- |
| Type | 1 | 15021,56 | 15021,6 | 8,5630 | 0,0040* |
| Error | 144 | 252611,63 | 1754,2 |  |  |
| C. Total | 145 | 267633,20 |  |  |  |

**Means for Oneway Anova**

| **Level** | **Number** | **Mean** | **Std Error** | **Lower 95%** | **Upper 95%** |
| --- | --- | --- | --- | --- | --- |
| benign | 73 | 41,6096 | 4,9021 | 31,920 | 51,299 |
| malignant | 73 | 61,8963 | 4,9021 | 52,207 | 71,586 |

Std Error uses a pooled estimate of error variance

**Analysis of original-gldm-HighGrayLevelEmphasis By Type**

**Quantiles**

| **Level** | **Minimum** | **10%** | **25%** | **Median** | **75%** | **90%** | **Maximum** |
| --- | --- | --- | --- | --- | --- | --- | --- |
| benign | 20,5 | 52,22222 | 97,2451 | 187,1818 | 290,3 | 459,0841 | 3234,545 |
| malignant | 4,468085 | 179,2681 | 257,1464 | 414,1875 | 702,2304 | 1013,389 | 2059,788 |

**Oneway Anova**

**Summary of Fit**

| Rsquare | 0,116872 |
| --- | --- |
| Adj Rsquare | 0,11074 |
| Root Mean Square Error | 367,3197 |
| Mean of Response | 383,3851 |
| Observations (or Sum Wgts) | 146 |

**Pooled t Test**

malignant-benign

Assuming equal variances

| Difference | 265,414 | t Ratio | 4,365415 |
| --- | --- | --- | --- |
| Std Err Dif | 60,799 | DF | 144 |
| Upper CL Dif | 385,588 | Prob > |t| | <,0001* |
| Lower CL Dif | 145,240 | Prob > t | <,0001* |
| Confidence | 0,95 | Prob < t | 1,0000 |

**Analysis of Variance**

| **Source** | **DF** | **Sum of Squares** | **Mean Square** | **F Ratio** | **Prob > F** |
| --- | --- | --- | --- | --- | --- |
| Type | 1 | 2571222 | 2571222 | 19,0569 | <,0001* |
| Error | 144 | 19429020 | 134924 |  |  |
| C. Total | 145 | 22000242 |  |  |  |

**Means for Oneway Anova**

| **Level** | **Number** | **Mean** | **Std Error** | **Lower 95%** | **Upper 95%** |
| --- | --- | --- | --- | --- | --- |
| benign | 73 | 250,678 | 42,992 | 165,70 | 335,65 |
| malignant | 73 | 516,092 | 42,992 | 431,12 | 601,07 |

Std Error uses a pooled estimate of error variance

**Oneway Analysis of original-gldm-DependenceEntropy By Type**

**Quantiles**

| **Level** | **Minimum** | **10%** | **25%** | **Median** | **75%** | **90%** | **Maximum** |
| --- | --- | --- | --- | --- | --- | --- | --- |
| benign | 2,251629 | 2,539164 | 3,028738 | 3,572431 | 3,851178 | 4,230442 | 4,678563 |
| malignant | 3,5 | 4,430275 | 5,201136 | 5,876638 | 6,311837 | 6,831185 | 7,573548 |

**Oneway Anova**

**Summary of Fit**

| Rsquare | 0,708217 |
| --- | --- |
| Adj Rsquare | 0,706191 |
| Root Mean Square Error | 0,733288 |
| Mean of Response | 4,605109 |
| Observations (or Sum Wgts) | 146 |

**Pooled t Test**

malignant-benign

Assuming equal variances

| Difference | 2,26915 | t Ratio | 18,69539 |
| --- | --- | --- | --- |
| Std Err Dif | 0,12137 | DF | 144 |
| Upper CL Dif | 2,50905 | Prob > |t| | <,0001* |
| Lower CL Dif | 2,02924 | Prob > t | <,0001* |
| Confidence | 0,95 | Prob < t | 1,0000 |

**Analysis of Variance**

| **Source** | **DF** | **Sum of Squares** | **Mean Square** | **F Ratio** | **Prob > F** |
| --- | --- | --- | --- | --- | --- |
| Type | 1 | 187,93973 | 187,940 | 349,5175 | <,0001* |
| Error | 144 | 77,43052 | 0,538 |  |  |
| C. Total | 145 | 265,37024 |  |  |  |

**Means for Oneway Anova**

| **Level** | **Number** | **Mean** | **Std Error** | **Lower 95%** | **Upper 95%** |
| --- | --- | --- | --- | --- | --- |
| benign | 73 | 3,47053 | 0,08582 | 3,3009 | 3,6402 |
| malignant | 73 | 5,73968 | 0,08582 | 5,5700 | 5,9093 |

Std Error uses a pooled estimate of error variance

**Oneway Analysis of original-gldm-DependenceNonUniformity By Type**

**Quantiles**

| **Level** | **Minimum** | **10%** | **25%** | **Median** | **75%** | **90%** | **Maximum** |
| --- | --- | --- | --- | --- | --- | --- | --- |
| benign | 3,333333 | 5,114286 | 7,863636 | 10,3 | 13,6 | 18,8087 | 30,28 |
| malignant | 6,914894 | 14,975 | 31,12158 | 58,27805 | 121,5465 | 382,9076 | 4518,518 |

**Oneway Anova**

**Summary of Fit**

| Rsquare | 0,052372 |
| --- | --- |
| Adj Rsquare | 0,045792 |
| Root Mean Square Error | 390,6332 |
| Mean of Response | 102,4597 |
| Observations (or Sum Wgts) | 146 |

**Pooled t Test**

malignant-benign

Assuming equal variances

| Difference | 182,405 | t Ratio | 2,821068 |
| --- | --- | --- | --- |
| Std Err Dif | 64,658 | DF | 144 |
| Upper CL Dif | 310,206 | Prob > |t| | 0,0055* |
| Lower CL Dif | 54,603 | Prob > t | 0,0027* |
| Confidence | 0,95 | Prob < t | 0,9973 |

**Analysis of Variance**

| **Source** | **DF** | **Sum of Squares** | **Mean Square** | **F Ratio** | **Prob > F** |
| --- | --- | --- | --- | --- | --- |
| Type | 1 | 1214410 | 1214410 | 7,9584 | 0,0055* |
| Error | 144 | 21973577 | 152594 |  |  |
| C. Total | 145 | 23187986 |  |  |  |

**Means for Oneway Anova**

| **Level** | **Number** | **Mean** | **Std Error** | **Lower 95%** | **Upper 95%** |
| --- | --- | --- | --- | --- | --- |
| benign | 73 | 11,257 | 45,720 | -79,1 | 101,63 |
| malignant | 73 | 193,662 | 45,720 | 103,3 | 284,03 |

Std Error uses a pooled estimate of error variance

**Oneway Analysis of original-gldm-GrayLevelNonUniformity By Type**

**Quantiles**

| **Level** | **Minimum** | **10%** | **25%** | **Median** | **75%** | **90%** | **Maximum** |
| --- | --- | --- | --- | --- | --- | --- | --- |
| benign | 1 | 1,208889 | 1,4 | 1,666667 | 2,20362 | 2,876842 | 3,888889 |
| malignant | 1,888889 | 2,952041 | 4,731061 | 7,895349 | 15,91416 | 59,18739 | 430,8082 |

**Oneway Anova**

**Summary of Fit**

| Rsquare | 0,075948 |
| --- | --- |
| Adj Rsquare | 0,069531 |
| Root Mean Square Error | 39,37342 |
| Mean of Response | 13,0583 |
| Observations (or Sum Wgts) | 146 |

**Pooled t Test**

malignant-benign

Assuming equal variances

| Difference | 22,4207 | t Ratio | 3,440264 |
| --- | --- | --- | --- |
| Std Err Dif | 6,5171 | DF | 144 |
| Upper CL Dif | 35,3023 | Prob > |t| | 0,0008* |
| Lower CL Dif | 9,5391 | Prob > t | 0,0004* |
| Confidence | 0,95 | Prob < t | 0,9996 |

**Analysis of Variance**

| **Source** | **DF** | **Sum of Squares** | **Mean Square** | **F Ratio** | **Prob > F** |
| --- | --- | --- | --- | --- | --- |
| Type | 1 | 18348,04 | 18348,0 | 11,8354 | 0,0008* |
| Error | 144 | 223238,28 | 1550,3 |  |  |
| C. Total | 145 | 241586,32 |  |  |  |

**Means for Oneway Anova**

| **Level** | **Number** | **Mean** | **Std Error** | **Lower 95%** | **Upper 95%** |
| --- | --- | --- | --- | --- | --- |
| benign | 73 | 1,8480 | 4,6083 | -7,26 | 10,957 |
| malignant | 73 | 24,2686 | 4,6083 | 15,16 | 33,377 |

Std Error uses a pooled estimate of error variance

**Oneway Analysis of original-gldm-SmallDependenceEmphasis By Type**

**Quantiles**

| **Level** | **Minimum** | **10%** | **25%** | **Median** | **75%** | **90%** | **Maximum** |
| --- | --- | --- | --- | --- | --- | --- | --- |
| benign | 0,495726 | 0,607143 | 0,734843 | 0,8125 | 0,888736 | 1 | 1 |
| malignant | 0,063013 | 0,439688 | 0,470547 | 0,525981 | 0,611688 | 0,674344 | 0,744856 |

**Oneway Anova**

**Summary of Fit**

| Rsquare | 0,573738 |
| --- | --- |
| Adj Rsquare | 0,570778 |
| Root Mean Square Error | 0,118171 |
| Mean of Response | 0,674022 |
| Observations (or Sum Wgts) | 146 |

**Pooled t Test**

malignant-benign

Assuming equal variances

| Difference | -0,27231 | t Ratio | -13,9219 |
| --- | --- | --- | --- |
| Std Err Dif | 0,01956 | DF | 144 |
| Upper CL Dif | -0,23365 | Prob > |t| | <,0001* |
| Lower CL Dif | -0,31097 | Prob > t | 1,0000 |
| Confidence | 0,95 | Prob < t | <,0001* |

**Analysis of Variance**

| **Source** | **DF** | **Sum of Squares** | **Mean Square** | **F Ratio** | **Prob > F** |
| --- | --- | --- | --- | --- | --- |
| Type | 1 | 2,7066033 | 2,70660 | 193,8203 | <,0001* |
| Error | 144 | 2,0108881 | 0,01396 |  |  |
| C. Total | 145 | 4,7174914 |  |  |  |

**Means for Oneway Anova**

| **Level** | **Number** | **Mean** | **Std Error** | **Lower 95%** | **Upper 95%** |
| --- | --- | --- | --- | --- | --- |
| benign | 73 | 0,810178 | 0,01383 | 0,78284 | 0,83752 |
| malignant | 73 | 0,537866 | 0,01383 | 0,51053 | 0,56520 |

Std Error uses a pooled estimate of error variance

**Oneway Analysis of original-gldm-SmallDependenceHighGrayLevelEmphasis By Type**

**Quantiles**

| **Level** | **Minimum** | **10%** | **25%** | **Median** | **75%** | **90%** | **Maximum** |
| --- | --- | --- | --- | --- | --- | --- | --- |
| benign | 17,83333 | 41,47021 | 78,46389 | 140,4706 | 256,9659 | 353,7075 | 2727,136 |
| malignant | 0,4792 | 95,52035 | 140,2437 | 235,2864 | 380,6886 | 556,1722 | 1097,426 |

**Oneway Anova**

**Summary of Fit**

| Rsquare | 0,022861 |
| --- | --- |
| Adj Rsquare | 0,016076 |
| Root Mean Square Error | 268,9079 |
| Mean of Response | 247,9929 |
| Observations (or Sum Wgts) | 146 |

**Pooled t Test**

malignant-benign

Assuming equal variances

| Difference | 81,70 | t Ratio | 1,835498 |
| --- | --- | --- | --- |
| Std Err Dif | 44,51 | DF | 144 |
| Upper CL Dif | 169,68 | Prob > |t| | 0,0685 |
| Lower CL Dif | -6,28 | Prob > t | 0,0342* |
| Confidence | 0,95 | Prob < t | 0,9658 |

**Analysis of Variance**

| **Source** | **DF** | **Sum of Squares** | **Mean Square** | **F Ratio** | **Prob > F** |
| --- | --- | --- | --- | --- | --- |
| Type | 1 | 243621 | 243621 | 3,3691 | 0,0685 |
| Error | 144 | 10412853 | 72311 |  |  |
| C. Total | 145 | 10656474 |  |  |  |

**Means for Oneway Anova**

| **Level** | **Number** | **Mean** | **Std Error** | **Lower 95%** | **Upper 95%** |
| --- | --- | --- | --- | --- | --- |
| benign | 73 | 207,144 | 31,473 | 144,93 | 269,35 |
| malignant | 73 | 288,842 | 31,473 | 226,63 | 351,05 |

Std Error uses a pooled estimate of error variance

**Oneway Analysis of original-gldm-DependenceNonUniformityNormalized By Type**

**Quantiles**

| **Level** | **Minimum** | **10%** | **25%** | **Median** | **75%** | **90%** | **Maximum** |
| --- | --- | --- | --- | --- | --- | --- | --- |
| benign | 0,3241 | 0,462735 | 0,528345 | 0,625 | 0,747374 | 1 | 1 |
| malignant | 0,147125 | 0,272033 | 0,287594 | 0,327744 | 0,390569 | 0,451978 | 0,533608 |

**Oneway Anova**

**Summary of Fit**

| Rsquare | 0,545705 |
| --- | --- |
| Adj Rsquare | 0,542551 |
| Root Mean Square Error | 0,146628 |
| Mean of Response | 0,505406 |
| Observations (or Sum Wgts) | 146 |

**Pooled t Test**

malignant-benign

Assuming equal variances

| Difference | -0,31920 | t Ratio | -13,152 |
| --- | --- | --- | --- |
| Std Err Dif | 0,02427 | DF | 144 |
| Upper CL Dif | -0,27123 | Prob > |t| | <,0001* |
| Lower CL Dif | -0,36717 | Prob > t | 1,0000 |
| Confidence | 0,95 | Prob < t | <,0001* |

**Analysis of Variance**

| **Source** | **DF** | **Sum of Squares** | **Mean Square** | **F Ratio** | **Prob > F** |
| --- | --- | --- | --- | --- | --- |
| Type | 1 | 3,7188982 | 3,71890 | 172,9749 | <,0001* |
| Error | 144 | 3,0959478 | 0,02150 |  |  |
| C. Total | 145 | 6,8148460 |  |  |  |

**Means for Oneway Anova**

| **Level** | **Number** | **Mean** | **Std Error** | **Lower 95%** | **Upper 95%** |
| --- | --- | --- | --- | --- | --- |
| benign | 73 | 0,665006 | 0,01716 | 0,63108 | 0,69893 |
| malignant | 73 | 0,345807 | 0,01716 | 0,31189 | 0,37973 |

Std Error uses a pooled estimate of error variance

**Oneway Analysis of original-gldm-LargeDependenceEmphasis By Type**

**Quantiles**

| **Level** | **Minimum** | **10%** | **25%** | **Median** | **75%** | **90%** | **Maximum** |
| --- | --- | --- | --- | --- | --- | --- | --- |
| benign | 1 | 1 | 1,445055 | 1,8 | 2,355882 | 3,429744 | 6 |
| malignant | 2,125 | 2,878431 | 3,682914 | 4,669903 | 5,693961 | 6,275691 | 39,46809 |

**Oneway Anova**

**Summary of Fit**

| Rsquare | 0,19949 |
| --- | --- |
| Adj Rsquare | 0,193931 |
| Root Mean Square Error | 3,098351 |
| Mean of Response | 3,550835 |
| Observations (or Sum Wgts) | 146 |

**Pooled t Test**

malignant-benign

Assuming equal variances

| Difference | 3,07215 | t Ratio | 5,99043 |
| --- | --- | --- | --- |
| Std Err Dif | 0,51284 | DF | 144 |
| Upper CL Dif | 4,08582 | Prob > |t| | <,0001* |
| Lower CL Dif | 2,05848 | Prob > t | <,0001* |
| Confidence | 0,95 | Prob < t | 1,0000 |

**Analysis of Variance**

| **Source** | **DF** | **Sum of Squares** | **Mean Square** | **F Ratio** | **Prob > F** |
| --- | --- | --- | --- | --- | --- |
| Type | 1 | 344,4905 | 344,491 | 35,8853 | <,0001* |
| Error | 144 | 1382,3681 | 9,600 |  |  |
| C. Total | 145 | 1726,8587 |  |  |  |

**Means for Oneway Anova**

| **Level** | **Number** | **Mean** | **Std Error** | **Lower 95%** | **Upper 95%** |
| --- | --- | --- | --- | --- | --- |
| benign | 73 | 2,01476 | 0,36263 | 1,2980 | 2,7315 |
| malignant | 73 | 5,08691 | 0,36263 | 4,3701 | 5,8037 |

Std Error uses a pooled estimate of error variance

**Oneway Analysis of original-gldm-LargeDependenceLowGrayLevelEmphasis By Type**

**Quantiles**

| **Level** | **Minimum** | **10%** | **25%** | **Median** | **75%** | **90%** | **Maximum** |
| --- | --- | --- | --- | --- | --- | --- | --- |
| benign | 0,029752 | 0,051853 | 0,067738 | 0,103673 | 0,17402 | 0,300208 | 2,79025 |
| malignant | 0,005259 | 0,009519 | 0,017183 | 0,031846 | 0,06865 | 0,157657 | 14,01079 |

**Oneway Anova**

**Summary of Fit**

| Rsquare | 0,001693 |
| --- | --- |
| Adj Rsquare | -0,00524 |
| Root Mean Square Error | 1,185749 |
| Mean of Response | 0,221849 |
| Observations (or Sum Wgts) | 146 |

**Pooled t Test**

malignant-benign

Assuming equal variances

| Difference | 0,09698 | t Ratio | 0,494138 |
| --- | --- | --- | --- |
| Std Err Dif | 0,19627 | DF | 144 |
| Upper CL Dif | 0,48492 | Prob > |t| | 0,6220 |
| Lower CL Dif | -0,29095 | Prob > t | 0,3110 |
| Confidence | 0,95 | Prob < t | 0,6890 |

**Analysis of Variance**

| **Source** | **DF** | **Sum of Squares** | **Mean Square** | **F Ratio** | **Prob > F** |
| --- | --- | --- | --- | --- | --- |
| Type | 1 | 0,34331 | 0,34331 | 0,2442 | 0,6220 |
| Error | 144 | 202,46407 | 1,40600 |  |  |
| C. Total | 145 | 202,80737 |  |  |  |

**Means for Oneway Anova**

| **Level** | **Number** | **Mean** | **Std Error** | **Lower 95%** | **Upper 95%** |
| --- | --- | --- | --- | --- | --- |
| benign | 73 | 0,173357 | 0,13878 | -0,1010 | 0,44767 |
| malignant | 73 | 0,270340 | 0,13878 | -0,0040 | 0,54465 |

Std Error uses a pooled estimate of error variance

**Oneway Analysis of original-gldm-DependenceVariance By Type**

**Quantiles**

| **Level** | **Minimum** | **10%** | **25%** | **Median** | **75%** | **90%** | **Maximum** |
| --- | --- | --- | --- | --- | --- | --- | --- |
| benign | 0 | 0 | 0,126313 | 0,195556 | 0,335533 | 0,664896 | 2,280612 |
| malignant | 0,234375 | 0,472716 | 0,684606 | 0,954633 | 1,291698 | 1,52244 | 4,229968 |

**Oneway Anova**

**Summary of Fit**

| Rsquare | 0,410234 |
| --- | --- |
| Adj Rsquare | 0,406138 |
| Root Mean Square Error | 0,450778 |
| Mean of Response | 0,652674 |
| Observations (or Sum Wgts) | 146 |

**Pooled t Test**

malignant-benign

Assuming equal variances

| Difference | 0,746747 | t Ratio | 10,00822 |
| --- | --- | --- | --- |
| Std Err Dif | 0,074613 | DF | 144 |
| Upper CL Dif | 0,894226 | Prob > |t| | <,0001* |
| Lower CL Dif | 0,599268 | Prob > t | <,0001* |
| Confidence | 0,95 | Prob < t | 1,0000 |

**Analysis of Variance**

| **Source** | **DF** | **Sum of Squares** | **Mean Square** | **F Ratio** | **Prob > F** |
| --- | --- | --- | --- | --- | --- |
| Type | 1 | 20,353545 | 20,3535 | 100,1645 | <,0001* |
| Error | 144 | 29,260972 | 0,2032 |  |  |
| C. Total | 145 | 49,614517 |  |  |  |

**Means for Oneway Anova**

| **Level** | **Number** | **Mean** | **Std Error** | **Lower 95%** | **Upper 95%** |
| --- | --- | --- | --- | --- | --- |
| benign | 73 | 0,27930 | 0,05276 | 0,17502 | 0,3836 |
| malignant | 73 | 1,02605 | 0,05276 | 0,92176 | 1,1303 |

Std Error uses a pooled estimate of error variance

**Oneway Analysis of original-gldm-LargeDependenceHighGrayLevelEmphasis By Type**

**Quantiles**

| **Level** | **Minimum** | **10%** | **25%** | **Median** | **75%** | **90%** | **Maximum** |
| --- | --- | --- | --- | --- | --- | --- | --- |
| benign | 32,5 | 88,3119 | 158,756 | 307,7692 | 488,5426 | 986,0246 | 5264,182 |
| malignant | 197,234 | 724,184 | 1021,533 | 1584,214 | 3183,469 | 4949,97 | 10906,75 |

**Oneway Anova**

**Summary of Fit**

| Rsquare | 0,306398 |
| --- | --- |
| Adj Rsquare | 0,301581 |
| Root Mean Square Error | 1382,579 |
| Mean of Response | 1377,958 |
| Observations (or Sum Wgts) | 146 |

**Pooled t Test**

malignant-benign

Assuming equal variances

| Difference | 1825,21 | t Ratio | 7,975707 |
| --- | --- | --- | --- |
| Std Err Dif | 228,85 | DF | 144 |
| Upper CL Dif | 2277,54 | Prob > |t| | <,0001* |
| Lower CL Dif | 1372,88 | Prob > t | <,0001* |
| Confidence | 0,95 | Prob < t | 1,0000 |

**Analysis of Variance**

| **Source** | **DF** | **Sum of Squares** | **Mean Square** | **F Ratio** | **Prob > F** |
| --- | --- | --- | --- | --- | --- |
| Type | 1 | 121595742 | 121595742 | 63,6119 | <,0001* |
| Error | 144 | 275259607 | 1911525 |  |  |
| C. Total | 145 | 396855348 |  |  |  |

**Means for Oneway Anova**

| **Level** | **Number** | **Mean** | **Std Error** | **Lower 95%** | **Upper 95%** |
| --- | --- | --- | --- | --- | --- |
| benign | 73 | 465,35 | 161,82 | 145,5 | 785,2 |
| malignant | 73 | 2290,56 | 161,82 | 1970,7 | 2610,4 |

Std Error uses a pooled estimate of error variance

**Oneway Analysis of original-gldm-SmallDependenceLowGrayLevelEmphasis By Type**

**Quantiles**

| **Level** | **Minimum** | **10%** | **25%** | **Median** | **75%** | **90%** | **Maximum** |
| --- | --- | --- | --- | --- | --- | --- | --- |
| benign | 0,025668 | 0,041064 | 0,053541 | 0,073784 | 0,10625 | 0,159346 | 0,213955 |
| malignant | 0,000602 | 0,001758 | 0,005539 | 0,01147 | 0,020012 | 0,03758 | 0,088751 |

**Oneway Anova**

**Summary of Fit**

| Rsquare | 0,531212 |
| --- | --- |
| Adj Rsquare | 0,527957 |
| Root Mean Square Error | 0,033078 |
| Mean of Response | 0,05033 |
| Observations (or Sum Wgts) | 146 |

**Pooled t Test**

malignant-benign

Assuming equal variances

| Difference | -0,06994 | t Ratio | -12,774 |
| --- | --- | --- | --- |
| Std Err Dif | 0,00548 | DF | 144 |
| Upper CL Dif | -0,05912 | Prob > |t| | <,0001* |
| Lower CL Dif | -0,08076 | Prob > t | 1,0000 |
| Confidence | 0,95 | Prob < t | <,0001* |

**Analysis of Variance**

| **Source** | **DF** | **Sum of Squares** | **Mean Square** | **F Ratio** | **Prob > F** |
| --- | --- | --- | --- | --- | --- |
| Type | 1 | 0,17853925 | 0,178539 | 163,1751 | <,0001* |
| Error | 144 | 0,15755867 | 0,001094 |  |  |
| C. Total | 145 | 0,33609792 |  |  |  |

**Means for Oneway Anova**

| **Level** | **Number** | **Mean** | **Std Error** | **Lower 95%** | **Upper 95%** |
| --- | --- | --- | --- | --- | --- |
| benign | 73 | 0,085299 | 0,00387 | 0,07765 | 0,09295 |
| malignant | 73 | 0,015360 | 0,00387 | 0,00771 | 0,02301 |

Std Error uses a pooled estimate of error variance

**Oneway Analysis of original-gldm-LowGrayLevelEmphasis By Type**

**Quantiles**

| **Level** | **Minimum** | **10%** | **25%** | **Median** | **75%** | **90%** | **Maximum** |
| --- | --- | --- | --- | --- | --- | --- | --- |
| benign | 0,027437 | 0,047627 | 0,060091 | 0,088342 | 0,122743 | 0,173801 | 0,35275 |
| malignant | 0,000978 | 0,002774 | 0,007541 | 0,014663 | 0,031466 | 0,049859 | 0,452866 |

**Oneway Anova**

**Summary of Fit**

| Rsquare | 0,294592 |
| --- | --- |
| Adj Rsquare | 0,289694 |
| Root Mean Square Error | 0,055054 |
| Mean of Response | 0,063113 |
| Observations (or Sum Wgts) | 146 |

**Pooled t Test**

malignant-benign

Assuming equal variances

| Difference | -0,07067 | t Ratio | -7,75482 |
| --- | --- | --- | --- |
| Std Err Dif | 0,00911 | DF | 144 |
| Upper CL Dif | -0,05265 | Prob > |t| | <,0001* |
| Lower CL Dif | -0,08868 | Prob > t | 1,0000 |
| Confidence | 0,95 | Prob < t | <,0001* |

**Analysis of Variance**

| **Source** | **DF** | **Sum of Squares** | **Mean Square** | **F Ratio** | **Prob > F** |
| --- | --- | --- | --- | --- | --- |
| Type | 1 | 0,18226971 | 0,182270 | 60,1373 | <,0001* |
| Error | 144 | 0,43644889 | 0,003031 |  |  |
| C. Total | 145 | 0,61871859 |  |  |  |

**Means for Oneway Anova**

| **Level** | **Number** | **Mean** | **Std Error** | **Lower 95%** | **Upper 95%** |
| --- | --- | --- | --- | --- | --- |
| benign | 73 | 0,098446 | 0,00644 | 0,08571 | 0,11118 |
| malignant | 73 | 0,027780 | 0,00644 | 0,01504 | 0,04052 |

Std Error uses a pooled estimate of error variance

**Oneway Analysis of original-glcm-JointAverage By Type**

**Quantiles**

| **Level** | **Minimum** | **10%** | **25%** | **Median** | **75%** | **90%** | **Maximum** |
| --- | --- | --- | --- | --- | --- | --- | --- |
| benign | 3,682143 | 6,652073 | 8,752656 | 12,33604 | 16,09501 | 19,66979 | 54,66667 |
| malignant | 2,025363 | 12,12757 | 14,16021 | 18,15261 | 23,91121 | 29,83152 | 42,7696 |

**Oneway Anova**

**Summary of Fit**

| Rsquare | 0,16906 |
| --- | --- |
| Adj Rsquare | 0,16329 |
| Root Mean Square Error | 6,933574 |
| Mean of Response | 16,28806 |
| Observations (or Sum Wgts) | 146 |

**Pooled t Test**

malignant-benign

Assuming equal variances

| Difference | 6,21195 | t Ratio | 5,412743 |
| --- | --- | --- | --- |
| Std Err Dif | 1,14765 | DF | 144 |
| Upper CL Dif | 8,48038 | Prob > |t| | <,0001* |
| Lower CL Dif | 3,94353 | Prob > t | <,0001* |
| Confidence | 0,95 | Prob < t | 1,0000 |

**Analysis of Variance**

| **Source** | **DF** | **Sum of Squares** | **Mean Square** | **F Ratio** | **Prob > F** |
| --- | --- | --- | --- | --- | --- |
| Type | 1 | 1408,4751 | 1408,48 | 29,2978 | <,0001* |
| Error | 144 | 6922,7212 | 48,07 |  |  |
| C. Total | 145 | 8331,1963 |  |  |  |

**Means for Oneway Anova**

| **Level** | **Number** | **Mean** | **Std Error** | **Lower 95%** | **Upper 95%** |
| --- | --- | --- | --- | --- | --- |
| benign | 73 | 13,1821 | 0,81151 | 11,578 | 14,786 |
| malignant | 73 | 19,3940 | 0,81151 | 17,790 | 20,998 |

Std Error uses a pooled estimate of error variance

**Oneway Analysis of original-glcm-SumAverage By Type**

**Quantiles**

| **Level** | **Minimum** | **10%** | **25%** | **Median** | **75%** | **90%** | **Maximum** |
| --- | --- | --- | --- | --- | --- | --- | --- |
| benign | 7,364286 | 13,30415 | 17,50531 | 24,67208 | 32,19003 | 39,33957 | 109,3333 |
| malignant | 4,050725 | 24,25514 | 28,32043 | 36,30522 | 47,82242 | 59,66305 | 85,53921 |

**Oneway Anova**

**Summary of Fit**

| Rsquare | 0,16906 |
| --- | --- |
| Adj Rsquare | 0,16329 |
| Root Mean Square Error | 13,86715 |
| Mean of Response | 32,57612 |
| Observations (or Sum Wgts) | 146 |

**Pooled t Test**

malignant-benign

Assuming equal variances

| Difference | 12,4239 | t Ratio | 5,412743 |
| --- | --- | --- | --- |
| Std Err Dif | 2,2953 | DF | 144 |
| Upper CL Dif | 16,9608 | Prob > |t| | <,0001* |
| Lower CL Dif | 7,8871 | Prob > t | <,0001* |
| Confidence | 0,95 | Prob < t | 1,0000 |

**Analysis of Variance**

| **Source** | **DF** | **Sum of Squares** | **Mean Square** | **F Ratio** | **Prob > F** |
| --- | --- | --- | --- | --- | --- |
| Type | 1 | 5633,900 | 5633,90 | 29,2978 | <,0001* |
| Error | 144 | 27690,885 | 192,30 |  |  |
| C. Total | 145 | 33324,785 |  |  |  |

**Means for Oneway Anova**

| **Level** | **Number** | **Mean** | **Std Error** | **Lower 95%** | **Upper 95%** |
| --- | --- | --- | --- | --- | --- |
| benign | 73 | 26,3642 | 1,6230 | 23,156 | 29,572 |
| malignant | 73 | 38,7881 | 1,6230 | 35,580 | 41,996 |

Std Error uses a pooled estimate of error variance

**Oneway Analysis of original-glcm-JointEntropy By Type**

**Quantiles**

| **Level** | **Minimum** | **10%** | **25%** | **Median** | **75%** | **90%** | **Maximum** |
| --- | --- | --- | --- | --- | --- | --- | --- |
| benign | 1,666667 | 2,741155 | 3,297598 | 4,212954 | 4,716578 | 5,193436 | 5,808142 |
| malignant | 2,245767 | 4,380355 | 6,067316 | 7,151312 | 8,035271 | 9,079324 | 10,45828 |

**Oneway Anova**

**Summary of Fit**

| Rsquare | 0,525078 |
| --- | --- |
| Adj Rsquare | 0,52178 |
| Root Mean Square Error | 1,396691 |
| Mean of Response | 5,469531 |
| Observations (or Sum Wgts) | 146 |

**Pooled t Test**

malignant-benign

Assuming equal variances

| Difference | 2,91699 | t Ratio | 12,61774 |
| --- | --- | --- | --- |
| Std Err Dif | 0,23118 | DF | 144 |
| Upper CL Dif | 3,37394 | Prob > |t| | <,0001* |
| Lower CL Dif | 2,46004 | Prob > t | <,0001* |
| Confidence | 0,95 | Prob < t | 1,0000 |

**Analysis of Variance**

| **Source** | **DF** | **Sum of Squares** | **Mean Square** | **F Ratio** | **Prob > F** |
| --- | --- | --- | --- | --- | --- |
| Type | 1 | 310,57299 | 310,573 | 159,2074 | <,0001* |
| Error | 144 | 280,90721 | 1,951 |  |  |
| C. Total | 145 | 591,48020 |  |  |  |

**Means for Oneway Anova**

| **Level** | **Number** | **Mean** | **Std Error** | **Lower 95%** | **Upper 95%** |
| --- | --- | --- | --- | --- | --- |
| benign | 73 | 4,01103 | 0,16347 | 3,6879 | 4,3341 |
| malignant | 73 | 6,92803 | 0,16347 | 6,6049 | 7,2511 |

Std Error uses a pooled estimate of error variance

**Oneway Analysis of original-glcm-ClusterShade By Type**

**Quantiles**

| **Level** | **Minimum** | **10%** | **25%** | **Median** | **75%** | **90%** | **Maximum** |
| --- | --- | --- | --- | --- | --- | --- | --- |
| benign | -22015,1 | -809,361 | -339,165 | -32,141 | 46,34969 | 218,71 | 700,194 |
| malignant | -2455,52 | -187,006 | -34,4733 | 295,7238 | 1056,714 | 2412,784 | 8848,825 |

**Oneway Anova**

**Summary of Fit**

| Rsquare | 0,079958 |
| --- | --- |
| Adj Rsquare | 0,073569 |
| Root Mean Square Error | 2207,272 |
| Mean of Response | 179,4079 |
| Observations (or Sum Wgts) | 146 |

**Pooled t Test**

malignant-benign

Assuming equal variances

| Difference | 1292,47 | t Ratio | 3,537607 |
| --- | --- | --- | --- |
| Std Err Dif | 365,35 | DF | 144 |
| Upper CL Dif | 2014,61 | Prob > |t| | 0,0005* |
| Lower CL Dif | 570,32 | Prob > t | 0,0003* |
| Confidence | 0,95 | Prob < t | 0,9997 |

**Analysis of Variance**

| **Source** | **DF** | **Sum of Squares** | **Mean Square** | **F Ratio** | **Prob > F** |
| --- | --- | --- | --- | --- | --- |
| Type | 1 | 60972053 | 60972053 | 12,5147 | 0,0005* |
| Error | 144 | 701575213 | 4872050,1 |  |  |
| C. Total | 145 | 762547266 |  |  |  |

**Means for Oneway Anova**

| **Level** | **Number** | **Mean** | **Std Error** | **Lower 95%** | **Upper 95%** |
| --- | --- | --- | --- | --- | --- |
| benign | 73 | -466,82 | 258,34 | -977,5 | 43,8 |
| malignant | 73 | 825,64 | 258,34 | 315,0 | 1336,3 |

Std Error uses a pooled estimate of error variance

**Oneway Analysis of original-glcm-MaximumProbability By Type**

**Quantiles**

| **Level** | **Minimum** | **10%** | **25%** | **Median** | **75%** | **90%** | **Maximum** |
| --- | --- | --- | --- | --- | --- | --- | --- |
| benign | 0,030242 | 0,051562 | 0,06201 | 0,093403 | 0,133681 | 0,213631 | 0,333333 |
| malignant | 0,003615 | 0,007414 | 0,012225 | 0,020774 | 0,032387 | 0,086641 | 0,25873 |

**Oneway Anova**

**Summary of Fit**

| Rsquare | 0,290845 |
| --- | --- |
| Adj Rsquare | 0,28592 |
| Root Mean Square Error | 0,056959 |
| Mean of Response | 0,071882 |
| Observations (or Sum Wgts) | 146 |

**Pooled t Test**

malignant-benign

Assuming equal variances

| Difference | -0,07245 | t Ratio | -7,68496 |
| --- | --- | --- | --- |
| Std Err Dif | 0,00943 | DF | 144 |
| Upper CL Dif | -0,05382 | Prob > |t| | <,0001* |
| Lower CL Dif | -0,09109 | Prob > t | 1,0000 |
| Confidence | 0,95 | Prob < t | <,0001* |

**Analysis of Variance**

| **Source** | **DF** | **Sum of Squares** | **Mean Square** | **F Ratio** | **Prob > F** |
| --- | --- | --- | --- | --- | --- |
| Type | 1 | 0,19160740 | 0,191607 | 59,0586 | <,0001* |
| Error | 144 | 0,46718805 | 0,003244 |  |  |
| C. Total | 145 | 0,65879545 |  |  |  |

**Means for Oneway Anova**

| **Level** | **Number** | **Mean** | **Std Error** | **Lower 95%** | **Upper 95%** |
| --- | --- | --- | --- | --- | --- |
| benign | 73 | 0,108108 | 0,00667 | 0,09493 | 0,12129 |
| malignant | 73 | 0,035655 | 0,00667 | 0,02248 | 0,04883 |

Std Error uses a pooled estimate of error variance

**Oneway Analysis of original-glcm-Idmn By Type**

**Quantiles**

| **Level** | **Minimum** | **10%** | **25%** | **Median** | **75%** | **90%** | **Maximum** |
| --- | --- | --- | --- | --- | --- | --- | --- |
| benign | 0,78501 | 0,8629 | 0,892252 | 0,921619 | 0,9376 | 0,951755 | 0,968483 |
| malignant | 0,828559 | 0,910605 | 0,933225 | 0,957826 | 0,967791 | 0,981964 | 0,988362 |

**Oneway Anova**

**Summary of Fit**

| Rsquare | 0,212948 |
| --- | --- |
| Adj Rsquare | 0,207482 |
| Root Mean Square Error | 0,035209 |
| Mean of Response | 0,929953 |
| Observations (or Sum Wgts) | 146 |

**Pooled t Test**

malignant-benign

Assuming equal variances

| Difference | 0,036377 | t Ratio | 6,241894 |
| --- | --- | --- | --- |
| Std Err Dif | 0,005828 | DF | 144 |
| Upper CL Dif | 0,047896 | Prob > |t| | <,0001* |
| Lower CL Dif | 0,024858 | Prob > t | <,0001* |
| Confidence | 0,95 | Prob < t | 1,0000 |

**Analysis of Variance**

| **Source** | **DF** | **Sum of Squares** | **Mean Square** | **F Ratio** | **Prob > F** |
| --- | --- | --- | --- | --- | --- |
| Type | 1 | 0,04830017 | 0,048300 | 38,9612 | <,0001* |
| Error | 144 | 0,17851650 | 0,001240 |  |  |
| C. Total | 145 | 0,22681667 |  |  |  |

**Means for Oneway Anova**

| **Level** | **Number** | **Mean** | **Std Error** | **Lower 95%** | **Upper 95%** |
| --- | --- | --- | --- | --- | --- |
| benign | 73 | 0,911764 | 0,00412 | 0,90362 | 0,91991 |
| malignant | 73 | 0,948141 | 0,00412 | 0,94000 | 0,95629 |

Std Error uses a pooled estimate of error variance

**Oneway Analysis of original-glcm-JointEnergy By Type**

**Quantiles**

| **Level** | **Minimum** | **10%** | **25%** | **Median** | **75%** | **90%** | **Maximum** |
| --- | --- | --- | --- | --- | --- | --- | --- |
| benign | 0,018724 | 0,029112 | 0,038868 | 0,057588 | 0,108581 | 0,196714 | 0,333333 |
| malignant | 0,001129 | 0,002444 | 0,00433 | 0,007815 | 0,016751 | 0,058199 | 0,237168 |

**Oneway Anova**

**Summary of Fit**

| Rsquare | 0,238786 |
| --- | --- |
| Adj Rsquare | 0,2335 |
| Root Mean Square Error | 0,056054 |
| Mean of Response | 0,053358 |
| Observations (or Sum Wgts) | 146 |

**Pooled t Test**

malignant-benign

Assuming equal variances

| Difference | -0,06236 | t Ratio | -6,72097 |
| --- | --- | --- | --- |
| Std Err Dif | 0,00928 | DF | 144 |
| Upper CL Dif | -0,04402 | Prob > |t| | <,0001* |
| Lower CL Dif | -0,08070 | Prob > t | 1,0000 |
| Confidence | 0,95 | Prob < t | <,0001* |

**Analysis of Variance**

| **Source** | **DF** | **Sum of Squares** | **Mean Square** | **F Ratio** | **Prob > F** |
| --- | --- | --- | --- | --- | --- |
| Type | 1 | 0,14192924 | 0,141929 | 45,1715 | <,0001* |
| Error | 144 | 0,45244925 | 0,003142 |  |  |
| C. Total | 145 | 0,59437849 |  |  |  |

**Means for Oneway Anova**

| **Level** | **Number** | **Mean** | **Std Error** | **Lower 95%** | **Upper 95%** |
| --- | --- | --- | --- | --- | --- |
| benign | 73 | 0,084536 | 0,00656 | 0,07157 | 0,09750 |
| malignant | 73 | 0,022179 | 0,00656 | 0,00921 | 0,03515 |

Std Error uses a pooled estimate of error variance

**Oneway Analysis of original-glcm-Contrast By Type**

**Quantiles**

| **Level** | **Minimum** | **10%** | **25%** | **Median** | **75%** | **90%** | **Maximum** |
| --- | --- | --- | --- | --- | --- | --- | --- |
| benign | 6,905556 | 17,54044 | 28,70131 | 45,28125 | 72,57232 | 125,5519 | 520,8333 |
| malignant | 0,871287 | 42,43405 | 52,28222 | 71,13198 | 109,1279 | 143,1324 | 232,2285 |

**Oneway Anova**

**Summary of Fit**

| Rsquare | 0,038597 |
| --- | --- |
| Adj Rsquare | 0,031921 |
| Root Mean Square Error | 56,47765 |
| Mean of Response | 73,24366 |
| Observations (or Sum Wgts) | 146 |

**Pooled t Test**

malignant-benign

Assuming equal variances

| Difference | 22,4769 | t Ratio | 2,404396 |
| --- | --- | --- | --- |
| Std Err Dif | 9,3482 | DF | 144 |
| Upper CL Dif | 40,9544 | Prob > |t| | 0,0175* |
| Lower CL Dif | 3,9994 | Prob > t | 0,0087* |
| Confidence | 0,95 | Prob < t | 0,9913 |

**Analysis of Variance**

| **Source** | **DF** | **Sum of Squares** | **Mean Square** | **F Ratio** | **Prob > F** |
| --- | --- | --- | --- | --- | --- |
| Type | 1 | 18440,18 | 18440,2 | 5,7811 | 0,0175* |
| Error | 144 | 459320,38 | 3189,7 |  |  |
| C. Total | 145 | 477760,56 |  |  |  |

**Means for Oneway Anova**

| **Level** | **Number** | **Mean** | **Std Error** | **Lower 95%** | **Upper 95%** |
| --- | --- | --- | --- | --- | --- |
| benign | 73 | 62,0052 | 6,6102 | 48,940 | 75,071 |
| malignant | 73 | 84,4821 | 6,6102 | 71,417 | 97,548 |

Std Error uses a pooled estimate of error variance

**Oneway Analysis of original-glcm-DifferenceEntropy By Type**

**Quantiles**

| **Level** | **Minimum** | **10%** | **25%** | **Median** | **75%** | **90%** | **Maximum** |
| --- | --- | --- | --- | --- | --- | --- | --- |
| benign | 0,639432 | 1,464376 | 2,027026 | 2,703745 | 3,02599 | 3,203608 | 3,547771 |
| malignant | 1,179581 | 2,964249 | 3,589408 | 3,830527 | 4,081488 | 4,304399 | 4,719208 |

**Oneway Anova**

**Summary of Fit**

| Rsquare | 0,453109 |
| --- | --- |
| Adj Rsquare | 0,449311 |
| Root Mean Square Error | 0,670415 |
| Mean of Response | 3,105553 |
| Observations (or Sum Wgts) | 146 |

**Pooled t Test**

malignant-benign

Assuming equal variances

| Difference | 1,21207 | t Ratio | 10,92275 |
| --- | --- | --- | --- |
| Std Err Dif | 0,11097 | DF | 144 |
| Upper CL Dif | 1,43141 | Prob > |t| | <,0001* |
| Lower CL Dif | 0,99274 | Prob > t | <,0001* |
| Confidence | 0,95 | Prob < t | 1,0000 |

**Analysis of Variance**

| **Source** | **DF** | **Sum of Squares** | **Mean Square** | **F Ratio** | **Prob > F** |
| --- | --- | --- | --- | --- | --- |
| Type | 1 | 53,62306 | 53,6231 | 119,3066 | <,0001* |
| Error | 144 | 64,72168 | 0,4495 |  |  |
| C. Total | 145 | 118,34474 |  |  |  |

**Means for Oneway Anova**

| **Level** | **Number** | **Mean** | **Std Error** | **Lower 95%** | **Upper 95%** |
| --- | --- | --- | --- | --- | --- |
| benign | 73 | 2,49952 | 0,07847 | 2,3444 | 2,6546 |
| malignant | 73 | 3,71159 | 0,07847 | 3,5565 | 3,8667 |

Std Error uses a pooled estimate of error variance

**Oneway Analysis of original-glcm-InverseVariance By Type**

**Quantiles**

| **Level** | **Minimum** | **10%** | **25%** | **Median** | **75%** | **90%** | **Maximum** |
| --- | --- | --- | --- | --- | --- | --- | --- |
| benign | 0,017825 | 0,06593 | 0,128131 | 0,169892 | 0,214156 | 0,252811 | 0,355005 |
| malignant | 0,086917 | 0,118962 | 0,134097 | 0,157492 | 0,182622 | 0,194688 | 0,528035 |

**Oneway Anova**

**Summary of Fit**

| Rsquare | 0,003887 |
| --- | --- |
| Adj Rsquare | -0,00303 |
| Root Mean Square Error | 0,06154 |
| Mean of Response | 0,166336 |
| Observations (or Sum Wgts) | 146 |

**Pooled t Test**

malignant-benign

Assuming equal variances

| Difference | -0,00764 | t Ratio | -0,74957 |
| --- | --- | --- | --- |
| Std Err Dif | 0,01019 | DF | 144 |
| Upper CL Dif | 0,01250 | Prob > |t| | 0,4547 |
| Lower CL Dif | -0,02777 | Prob > t | 0,7726 |
| Confidence | 0,95 | Prob < t | 0,2274 |

**Analysis of Variance**

| **Source** | **DF** | **Sum of Squares** | **Mean Square** | **F Ratio** | **Prob > F** |
| --- | --- | --- | --- | --- | --- |
| Type | 1 | 0,00212784 | 0,002128 | 0,5619 | 0,4547 |
| Error | 144 | 0,54534984 | 0,003787 |  |  |
| C. Total | 145 | 0,54747768 |  |  |  |

**Means for Oneway Anova**

| **Level** | **Number** | **Mean** | **Std Error** | **Lower 95%** | **Upper 95%** |
| --- | --- | --- | --- | --- | --- |
| benign | 73 | 0,170153 | 0,00720 | 0,15592 | 0,18439 |
| malignant | 73 | 0,162518 | 0,00720 | 0,14828 | 0,17675 |

Std Error uses a pooled estimate of error variance

**Oneway Analysis of original-glcm-DifferenceVariance By Type**

**Quantiles**

| **Level** | **Minimum** | **10%** | **25%** | **Median** | **75%** | **90%** | **Maximum** |
| --- | --- | --- | --- | --- | --- | --- | --- |
| benign | 2,518519 | 4,813059 | 6,983909 | 13,11841 | 22,74738 | 31,29788 | 161,4965 |
| malignant | 0,335983 | 15,24185 | 19,82924 | 26,70544 | 39,79232 | 54,54745 | 104,0875 |

**Oneway Anova**

**Summary of Fit**

| Rsquare | 0,128805 |
| --- | --- |
| Adj Rsquare | 0,122755 |
| Root Mean Square Error | 18,64644 |
| Mean of Response | 24,40401 |
| Observations (or Sum Wgts) | 146 |

**Pooled t Test**

malignant-benign

Assuming equal variances

| Difference | 14,2409 | t Ratio | 4,614122 |
| --- | --- | --- | --- |
| Std Err Dif | 3,0864 | DF | 144 |
| Upper CL Dif | 20,3414 | Prob > |t| | <,0001* |
| Lower CL Dif | 8,1405 | Prob > t | <,0001* |
| Confidence | 0,95 | Prob < t | 1,0000 |

**Analysis of Variance**

| **Source** | **DF** | **Sum of Squares** | **Mean Square** | **F Ratio** | **Prob > F** |
| --- | --- | --- | --- | --- | --- |
| Type | 1 | 7402,356 | 7402,36 | 21,2901 | <,0001* |
| Error | 144 | 50067,310 | 347,69 |  |  |
| C. Total | 145 | 57469,666 |  |  |  |

**Means for Oneway Anova**

| **Level** | **Number** | **Mean** | **Std Error** | **Lower 95%** | **Upper 95%** |
| --- | --- | --- | --- | --- | --- |
| benign | 73 | 17,2835 | 2,1824 | 12,970 | 21,597 |
| malignant | 73 | 31,5245 | 2,1824 | 27,211 | 35,838 |

Std Error uses a pooled estimate of error variance

**Oneway Analysis of original-glcm-Idn By Type**

**Quantiles**

| **Level** | **Minimum** | **10%** | **25%** | **Median** | **75%** | **90%** | **Maximum** |
| --- | --- | --- | --- | --- | --- | --- | --- |
| benign | 0,687454 | 0,752681 | 0,780164 | 0,813849 | 0,837896 | 0,849698 | 0,881991 |
| malignant | 0,739904 | 0,809953 | 0,835418 | 0,865683 | 0,883962 | 0,909551 | 0,930662 |

**Oneway Anova**

**Summary of Fit**

| Rsquare | 0,285764 |
| --- | --- |
| Adj Rsquare | 0,280804 |
| Root Mean Square Error | 0,041061 |
| Mean of Response | 0,832894 |
| Observations (or Sum Wgts) | 146 |

**Pooled t Test**

malignant-benign

Assuming equal variances

| Difference | 0,051587 | t Ratio | 7,59039 |
| --- | --- | --- | --- |
| Std Err Dif | 0,006796 | DF | 144 |
| Upper CL Dif | 0,065021 | Prob > |t| | <,0001* |
| Lower CL Dif | 0,038154 | Prob > t | <,0001* |
| Confidence | 0,95 | Prob < t | 1,0000 |

**Analysis of Variance**

| **Source** | **DF** | **Sum of Squares** | **Mean Square** | **F Ratio** | **Prob > F** |
| --- | --- | --- | --- | --- | --- |
| Type | 1 | 0,09713524 | 0,097135 | 57,6140 | <,0001* |
| Error | 144 | 0,24277901 | 0,001686 |  |  |
| C. Total | 145 | 0,33991425 |  |  |  |

**Means for Oneway Anova**

| **Level** | **Number** | **Mean** | **Std Error** | **Lower 95%** | **Upper 95%** |
| --- | --- | --- | --- | --- | --- |
| benign | 73 | 0,807100 | 0,00481 | 0,79760 | 0,81660 |
| malignant | 73 | 0,858687 | 0,00481 | 0,84919 | 0,86819 |

Std Error uses a pooled estimate of error variance

**Oneway Analysis of original-glcm-Idm By Type**

**Quantiles**

| **Level** | **Minimum** | **10%** | **25%** | **Median** | **75%** | **90%** | **Maximum** |
| --- | --- | --- | --- | --- | --- | --- | --- |
| benign | 0,027941 | 0,068822 | 0,107513 | 0,156266 | 0,195307 | 0,248854 | 0,39947 |
| malignant | 0,079047 | 0,123152 | 0,145158 | 0,156047 | 0,183052 | 0,19563 | 0,674197 |

**Oneway Anova**

**Summary of Fit**

| Rsquare | 0,001563 |
| --- | --- |
| Adj Rsquare | -0,00537 |
| Root Mean Square Error | 0,070157 |
| Mean of Response | 0,163385 |
| Observations (or Sum Wgts) | 146 |

**Pooled t Test**

malignant-benign

Assuming equal variances

| Difference | 0,00551 | t Ratio | 0,474811 |
| --- | --- | --- | --- |
| Std Err Dif | 0,01161 | DF | 144 |
| Upper CL Dif | 0,02847 | Prob > |t| | 0,6356 |
| Lower CL Dif | -0,01744 | Prob > t | 0,3178 |
| Confidence | 0,95 | Prob < t | 0,6822 |

**Analysis of Variance**

| **Source** | **DF** | **Sum of Squares** | **Mean Square** | **F Ratio** | **Prob > F** |
| --- | --- | --- | --- | --- | --- |
| Type | 1 | 0,00110965 | 0,001110 | 0,2254 | 0,6356 |
| Error | 144 | 0,70877407 | 0,004922 |  |  |
| C. Total | 145 | 0,70988372 |  |  |  |

**Means for Oneway Anova**

| **Level** | **Number** | **Mean** | **Std Error** | **Lower 95%** | **Upper 95%** |
| --- | --- | --- | --- | --- | --- |
| benign | 73 | 0,160628 | 0,00821 | 0,14440 | 0,17686 |
| malignant | 73 | 0,166142 | 0,00821 | 0,14991 | 0,18237 |

Std Error uses a pooled estimate of error variance

**Oneway Analysis of original-glcm-Correlation By Type**

**Quantiles**

| **Level** | **Minimum** | **10%** | **25%** | **Median** | **75%** | **90%** | **Maximum** |
| --- | --- | --- | --- | --- | --- | --- | --- |
| benign | -0,41367 | -0,21766 | -0,04229 | 0,145402 | 0,308081 | 0,4826 | 0,691247 |
| malignant | -0,3856 | -0,02533 | 0,127932 | 0,24608 | 0,378413 | 0,495851 | 0,578163 |

**Oneway Anova**

**Summary of Fit**

| Rsquare | 0,046479 |
| --- | --- |
| Adj Rsquare | 0,039857 |
| Root Mean Square Error | 0,226935 |
| Mean of Response | 0,188803 |
| Observations (or Sum Wgts) | 146 |

**Pooled t Test**

malignant-benign

Assuming equal variances

| Difference | 0,099518 | t Ratio | 2,649385 |
| --- | --- | --- | --- |
| Std Err Dif | 0,037563 | DF | 144 |
| Upper CL Dif | 0,173763 | Prob > |t| | 0,0090* |
| Lower CL Dif | 0,025272 | Prob > t | 0,0045* |
| Confidence | 0,95 | Prob < t | 0,9955 |

**Analysis of Variance**

| **Source** | **DF** | **Sum of Squares** | **Mean Square** | **F Ratio** | **Prob > F** |
| --- | --- | --- | --- | --- | --- |
| Type | 1 | 0,3614868 | 0,361487 | 7,0192 | 0,0090* |
| Error | 144 | 7,4159162 | 0,051499 |  |  |
| C. Total | 145 | 7,7774030 |  |  |  |

**Means for Oneway Anova**

| **Level** | **Number** | **Mean** | **Std Error** | **Lower 95%** | **Upper 95%** |
| --- | --- | --- | --- | --- | --- |
| benign | 73 | 0,139044 | 0,02656 | 0,08655 | 0,19154 |
| malignant | 73 | 0,238562 | 0,02656 | 0,18606 | 0,29106 |

Std Error uses a pooled estimate of error variance

**Oneway Analysis of original-glcm-Autocorrelation By Type**

**Quantiles**

| **Level** | **Minimum** | **10%** | **25%** | **Median** | **75%** | **90%** | **Maximum** |
| --- | --- | --- | --- | --- | --- | --- | --- |
| benign | 14,175 | 44,40404 | 76,82697 | 158,2778 | 261,5845 | 395,234 | 3057,26 |
| malignant | 4,269287 | 148,3225 | 210,9895 | 336,303 | 579,4334 | 919,882 | 1915,536 |

**Oneway Anova**

**Summary of Fit**

| Rsquare | 0,089219 |
| --- | --- |
| Adj Rsquare | 0,082894 |
| Root Mean Square Error | 343,9199 |
| Mean of Response | 334,6966 |
| Observations (or Sum Wgts) | 146 |

**Pooled t Test**

malignant-benign

Assuming equal variances

| Difference | 213,803 | t Ratio | 3,755803 |
| --- | --- | --- | --- |
| Std Err Dif | 56,926 | DF | 144 |
| Upper CL Dif | 326,322 | Prob > |t| | 0,0003* |
| Lower CL Dif | 101,284 | Prob > t | 0,0001* |
| Confidence | 0,95 | Prob < t | 0,9999 |

**Analysis of Variance**

| **Source** | **DF** | **Sum of Squares** | **Mean Square** | **F Ratio** | **Prob > F** |
| --- | --- | --- | --- | --- | --- |
| Type | 1 | 1668477 | 1668477 | 14,1061 | 0,0003* |
| Error | 144 | 17032450 | 118281 |  |  |
| C. Total | 145 | 18700927 |  |  |  |

**Means for Oneway Anova**

| **Level** | **Number** | **Mean** | **Std Error** | **Lower 95%** | **Upper 95%** |
| --- | --- | --- | --- | --- | --- |
| benign | 73 | 227,795 | 40,253 | 148,23 | 307,36 |
| malignant | 73 | 441,598 | 40,253 | 362,04 | 521,16 |

Std Error uses a pooled estimate of error variance

**Oneway Analysis of original-glcm-SumEntropy By Type**

**Quantiles**

| **Level** | **Minimum** | **10%** | **25%** | **Median** | **75%** | **90%** | **Maximum** |
| --- | --- | --- | --- | --- | --- | --- | --- |
| benign | 0,666667 | 1,552583 | 2,289092 | 3,122394 | 3,457672 | 3,868834 | 4,398099 |
| malignant | 1,241601 | 3,22181 | 4,30557 | 4,943711 | 5,439369 | 5,775041 | 6,321469 |

**Oneway Anova**

**Summary of Fit**

| Rsquare | 0,503866 |
| --- | --- |
| Adj Rsquare | 0,500421 |
| Root Mean Square Error | 0,929901 |
| Mean of Response | 3,785448 |
| Observations (or Sum Wgts) | 146 |

**Pooled t Test**

malignant-benign

Assuming equal variances

| Difference | 1,86136 | t Ratio | 12,09315 |
| --- | --- | --- | --- |
| Std Err Dif | 0,15392 | DF | 144 |
| Upper CL Dif | 2,16559 | Prob > |t| | <,0001* |
| Lower CL Dif | 1,55713 | Prob > t | <,0001* |
| Confidence | 0,95 | Prob < t | 1,0000 |

**Analysis of Variance**

| **Source** | **DF** | **Sum of Squares** | **Mean Square** | **F Ratio** | **Prob > F** |
| --- | --- | --- | --- | --- | --- |
| Type | 1 | 126,45969 | 126,460 | 146,2444 | <,0001* |
| Error | 144 | 124,51897 | 0,865 |  |  |
| C. Total | 145 | 250,97866 |  |  |  |

**Means for Oneway Anova**

| **Level** | **Number** | **Mean** | **Std Error** | **Lower 95%** | **Upper 95%** |
| --- | --- | --- | --- | --- | --- |
| benign | 73 | 2,85477 | 0,10884 | 2,6396 | 3,0699 |
| malignant | 73 | 4,71613 | 0,10884 | 4,5010 | 4,9313 |

Std Error uses a pooled estimate of error variance

**Oneway Analysis of original-glcm-SumSquares By Type**

**Quantiles**

| **Level** | **Minimum** | **10%** | **25%** | **Median** | **75%** | **90%** | **Maximum** |
| --- | --- | --- | --- | --- | --- | --- | --- |
| benign | 2,907495 | 10,91951 | 16,44661 | 30,39891 | 44,66157 | 68,23396 | 315,4436 |
| malignant | 0,594845 | 26,63575 | 34,50958 | 50,64598 | 72,57086 | 113,1538 | 179,9478 |

**Oneway Anova**

**Summary of Fit**

| Rsquare | 0,077947 |
| --- | --- |
| Adj Rsquare | 0,071544 |
| Root Mean Square Error | 36,61276 |
| Mean of Response | 48,17062 |
| Observations (or Sum Wgts) | 146 |

**Pooled t Test**

malignant-benign

Assuming equal variances

| Difference | 21,1441 | t Ratio | 3,489019 |
| --- | --- | --- | --- |
| Std Err Dif | 6,0602 | DF | 144 |
| Upper CL Dif | 33,1225 | Prob > |t| | 0,0006* |
| Lower CL Dif | 9,1657 | Prob > t | 0,0003* |
| Confidence | 0,95 | Prob < t | 0,9997 |

**Analysis of Variance**

| **Source** | **DF** | **Sum of Squares** | **Mean Square** | **F Ratio** | **Prob > F** |
| --- | --- | --- | --- | --- | --- |
| Type | 1 | 16318,17 | 16318,2 | 12,1733 | 0,0006* |
| Error | 144 | 193031,13 | 1340,5 |  |  |
| C. Total | 145 | 209349,31 |  |  |  |

**Means for Oneway Anova**

| **Level** | **Number** | **Mean** | **Std Error** | **Lower 95%** | **Upper 95%** |
| --- | --- | --- | --- | --- | --- |
| benign | 73 | 37,5986 | 4,2852 | 29,129 | 46,069 |
| malignant | 73 | 58,7427 | 4,2852 | 50,273 | 67,213 |

Std Error uses a pooled estimate of error variance

**Oneway Analysis of original-glcm-ClusterProminence By Type**

**Quantiles**

| **Level** | **Minimum** | **10%** | **25%** | **Median** | **75%** | **90%** | **Maximum** |
| --- | --- | --- | --- | --- | --- | --- | --- |
| benign | 54,90171 | 1088,634 | 3190,658 | 13793,26 | 33376,01 | 75101,25 | 1829086 |
| malignant | 5,141301 | 10389,75 | 20355,51 | 43914,06 | 114158,3 | 268620,1 | 864685,9 |

**Oneway Anova**

**Summary of Fit**

| Rsquare | 0,022268 |
| --- | --- |
| Adj Rsquare | 0,015478 |
| Root Mean Square Error | 187940,6 |
| Mean of Response | 75813,57 |
| Observations (or Sum Wgts) | 146 |

**Pooled t Test**

malignant-benign

Assuming equal variances

| Difference | 56336 | t Ratio | 1,810961 |
| --- | --- | --- | --- |
| Std Err Dif | 31108 | DF | 144 |
| Upper CL Dif | 117823 | Prob > |t| | 0,0722 |
| Lower CL Dif | -5152 | Prob > t | 0,0361* |
| Confidence | 0,95 | Prob < t | 0,9639 |

**Analysis of Variance**

| **Source** | **DF** | **Sum of Squares** | **Mean Square** | **F Ratio** | **Prob > F** |
| --- | --- | --- | --- | --- | --- |
| Type | 1 | 1,1584e+11 | 1,158e+11 | 3,2796 | 0,0722 |
| Error | 144 | 5,0863e+12 | 3,532e+10 |  |  |
| C. Total | 145 | 5,2022e+12 |  |  |  |

**Means for Oneway Anova**

| **Level** | **Number** | **Mean** | **Std Error** | **Lower 95%** | **Upper 95%** |
| --- | --- | --- | --- | --- | --- |
| benign | 73 | 47646 | 21997 | 4167 | 91124 |
| malignant | 73 | 103981 | 21997 | 60503 | 147460 |

Std Error uses a pooled estimate of error variance

**Oneway Analysis of original-glcm-Imc2 By Type**

**Quantiles**

| **Level** | **Minimum** | **10%** | **25%** | **Median** | **75%** | **90%** | **Maximum** |
| --- | --- | --- | --- | --- | --- | --- | --- |
| benign | 0,92183 | 0,969443 | 0,986682 | 0,993916 | 0,996935 | 0,997844 | 0,998942 |
| malignant | 0,675327 | 0,845046 | 0,954689 | 0,98831 | 0,995996 | 0,998425 | 0,999087 |

**Oneway Anova**

**Summary of Fit**

| Rsquare | 0,097065 |
| --- | --- |
| Adj Rsquare | 0,090795 |
| Root Mean Square Error | 0,052181 |
| Mean of Response | 0,971081 |
| Observations (or Sum Wgts) | 146 |

**Pooled t Test**

malignant-benign

Assuming equal variances

| Difference | -0,03398 | t Ratio | -3,93446 |
| --- | --- | --- | --- |
| Std Err Dif | 0,00864 | DF | 144 |
| Upper CL Dif | -0,01691 | Prob > |t| | 0,0001* |
| Lower CL Dif | -0,05105 | Prob > t | 0,9999 |
| Confidence | 0,95 | Prob < t | <,0001* |

**Analysis of Variance**

| **Source** | **DF** | **Sum of Squares** | **Mean Square** | **F Ratio** | **Prob > F** |
| --- | --- | --- | --- | --- | --- |
| Type | 1 | 0,04215032 | 0,042150 | 15,4800 | 0,0001* |
| Error | 144 | 0,39209715 | 0,002723 |  |  |
| C. Total | 145 | 0,43424748 |  |  |  |

**Means for Oneway Anova**

| **Level** | **Number** | **Mean** | **Std Error** | **Lower 95%** | **Upper 95%** |
| --- | --- | --- | --- | --- | --- |
| benign | 73 | 0,988072 | 0,00611 | 0,97600 | 1,0001 |
| malignant | 73 | 0,954090 | 0,00611 | 0,94202 | 0,9662 |

Std Error uses a pooled estimate of error variance

**Oneway Analysis of original-glcm-Imc1 By Type**

**Quantiles**

| **Level** | **Minimum** | **10%** | **25%** | **Median** | **75%** | **90%** | **Maximum** |
| --- | --- | --- | --- | --- | --- | --- | --- |
| benign | -0,9537 | -0,8841 | -0,7908 | -0,72445 | -0,65813 | -0,571 | -0,42354 |
| malignant | -0,85465 | -0,72918 | -0,60556 | -0,45271 | -0,26947 | -0,13551 | -0,07315 |

**Oneway Anova**

**Summary of Fit**

| Rsquare | 0,408204 |
| --- | --- |
| Adj Rsquare | 0,404094 |
| Root Mean Square Error | 0,17182 |
| Mean of Response | -0,58028 |
| Observations (or Sum Wgts) | 146 |

**Pooled t Test**

malignant-benign

Assuming equal variances

| Difference | 0,283440 | t Ratio | 9,966295 |
| --- | --- | --- | --- |
| Std Err Dif | 0,028440 | DF | 144 |
| Upper CL Dif | 0,339653 | Prob > |t| | <,0001* |
| Lower CL Dif | 0,227226 | Prob > t | <,0001* |
| Confidence | 0,95 | Prob < t | 1,0000 |

**Analysis of Variance**

| **Source** | **DF** | **Sum of Squares** | **Mean Square** | **F Ratio** | **Prob > F** |
| --- | --- | --- | --- | --- | --- |
| Type | 1 | 2,9323379 | 2,93234 | 99,3270 | <,0001* |
| Error | 144 | 4,2511753 | 0,02952 |  |  |
| C. Total | 145 | 7,1835133 |  |  |  |

**Means for Oneway Anova**

| **Level** | **Number** | **Mean** | **Std Error** | **Lower 95%** | **Upper 95%** |
| --- | --- | --- | --- | --- | --- |
| benign | 73 | -0,72200 | 0,02011 | -0,7617 | -0,6822 |
| malignant | 73 | -0,43856 | 0,02011 | -0,4783 | -0,3988 |

Std Error uses a pooled estimate of error variance

**Oneway Analysis of original-glcm-DifferenceAverage By Type**

**Quantiles**

| **Level** | **Minimum** | **10%** | **25%** | **Median** | **75%** | **90%** | **Maximum** |
| --- | --- | --- | --- | --- | --- | --- | --- |
| benign | 1,950198 | 3,338891 | 4,41632 | 5,415476 | 6,993986 | 9,114934 | 16,91667 |
| malignant | 0,688219 | 5,080866 | 5,57287 | 6,590278 | 7,917903 | 9,218344 | 12,12089 |

**Oneway Anova**

**Summary of Fit**

| Rsquare | 0,043713 |
| --- | --- |
| Adj Rsquare | 0,037072 |
| Root Mean Square Error | 2,146739 |
| Mean of Response | 6,393795 |
| Observations (or Sum Wgts) | 146 |

**Pooled t Test**

malignant-benign

Assuming equal variances

| Difference | 0,91164 | t Ratio | 2,565612 |
| --- | --- | --- | --- |
| Std Err Dif | 0,35533 | DF | 144 |
| Upper CL Dif | 1,61398 | Prob > |t| | 0,0113* |
| Lower CL Dif | 0,20930 | Prob > t | 0,0057* |
| Confidence | 0,95 | Prob < t | 0,9943 |

**Analysis of Variance**

| **Source** | **DF** | **Sum of Squares** | **Mean Square** | **F Ratio** | **Prob > F** |
| --- | --- | --- | --- | --- | --- |
| Type | 1 | 30,33474 | 30,3347 | 6,5824 | 0,0113* |
| Error | 144 | 663,62210 | 4,6085 |  |  |
| C. Total | 145 | 693,95684 |  |  |  |

**Means for Oneway Anova**

| **Level** | **Number** | **Mean** | **Std Error** | **Lower 95%** | **Upper 95%** |
| --- | --- | --- | --- | --- | --- |
| benign | 73 | 5,93797 | 0,25126 | 5,4413 | 6,4346 |
| malignant | 73 | 6,84961 | 0,25126 | 6,3530 | 7,3462 |

Std Error uses a pooled estimate of error variance

**Oneway Analysis of original-glcm-Id By Type**

**Quantiles**

| **Level** | **Minimum** | **10%** | **25%** | **Median** | **75%** | **90%** | **Maximum** |
| --- | --- | --- | --- | --- | --- | --- | --- |
| benign | 0,113835 | 0,155432 | 0,200214 | 0,249698 | 0,285175 | 0,349063 | 0,472441 |
| malignant | 0,15493 | 0,203942 | 0,228053 | 0,244956 | 0,2687 | 0,286552 | 0,686402 |

**Oneway Anova**

**Summary of Fit**

| Rsquare | 0,000108 |
| --- | --- |
| Adj Rsquare | -0,00684 |
| Root Mean Square Error | 0,066702 |
| Mean of Response | 0,251898 |
| Observations (or Sum Wgts) | 146 |

**Pooled t Test**

malignant-benign

Assuming equal variances

| Difference | -0,00138 | t Ratio | -0,12479 |
| --- | --- | --- | --- |
| Std Err Dif | 0,01104 | DF | 144 |
| Upper CL Dif | 0,02044 | Prob > |t| | 0,9009 |
| Lower CL Dif | -0,02320 | Prob > t | 0,5496 |
| Confidence | 0,95 | Prob < t | 0,4504 |

**Analysis of Variance**

| **Source** | **DF** | **Sum of Squares** | **Mean Square** | **F Ratio** | **Prob > F** |
| --- | --- | --- | --- | --- | --- |
| Type | 1 | 0,00006929 | 0,000069 | 0,0156 | 0,9009 |
| Error | 144 | 0,64067995 | 0,004449 |  |  |
| C. Total | 145 | 0,64074924 |  |  |  |

**Means for Oneway Anova**

| **Level** | **Number** | **Mean** | **Std Error** | **Lower 95%** | **Upper 95%** |
| --- | --- | --- | --- | --- | --- |
| benign | 73 | 0,252587 | 0,00781 | 0,23716 | 0,26802 |
| malignant | 73 | 0,251209 | 0,00781 | 0,23578 | 0,26664 |

Std Error uses a pooled estimate of error variance

**Oneway Analysis of original-glcm-ClusterTendency By Type**

**Quantiles**

| **Level** | **Minimum** | **10%** | **25%** | **Median** | **75%** | **90%** | **Maximum** |
| --- | --- | --- | --- | --- | --- | --- | --- |
| benign | 4,724425 | 18,28551 | 38,86896 | 70,89797 | 108,3072 | 174,7814 | 740,941 |
| malignant | 1,508091 | 55,12253 | 81,96105 | 118,8427 | 198,9707 | 305,3623 | 487,5625 |

**Oneway Anova**

**Summary of Fit**

| Rsquare | 0,092363 |
| --- | --- |
| Adj Rsquare | 0,08606 |
| Root Mean Square Error | 98,00799 |
| Mean of Response | 119,4388 |
| Observations (or Sum Wgts) | 146 |

**Pooled t Test**

malignant-benign

Assuming equal variances

| Difference | 62,0995 | t Ratio | 3,828012 |
| --- | --- | --- | --- |
| Std Err Dif | 16,2224 | DF | 144 |
| Upper CL Dif | 94,1643 | Prob > |t| | 0,0002* |
| Lower CL Dif | 30,0348 | Prob > t | <,0001* |
| Confidence | 0,95 | Prob < t | 0,9999 |

**Analysis of Variance**

| **Source** | **DF** | **Sum of Squares** | **Mean Square** | **F Ratio** | **Prob > F** |
| --- | --- | --- | --- | --- | --- |
| Type | 1 | 140756,9 | 140757 | 14,6537 | 0,0002* |
| Error | 144 | 1383201,5 | 9606 |  |  |
| C. Total | 145 | 1523958,4 |  |  |  |

**Means for Oneway Anova**

| **Level** | **Number** | **Mean** | **Std Error** | **Lower 95%** | **Upper 95%** |
| --- | --- | --- | --- | --- | --- |
| benign | 73 | 88,389 | 11,471 | 65,72 | 111,06 |
| malignant | 73 | 150,489 | 11,471 | 127,82 | 173,16 |

Std Error uses a pooled estimate of error variance

**Oneway Analysis of original-firstorder-InterquartileRange By Type**

**Quantiles**

| **Level** | **Minimum** | **10%** | **25%** | **Median** | **75%** | **90%** | **Maximum** |
| --- | --- | --- | --- | --- | --- | --- | --- |
| benign | 38 | 82,35 | 134 | 184 | 242 | 329,85 | 481 |
| malignant | 29,5 | 172,25 | 197 | 246,5 | 301,375 | 401,5 | 508 |

**Oneway Anova**

**Summary of Fit**

| Rsquare | 0,123164 |
| --- | --- |
| Adj Rsquare | 0,117075 |
| Root Mean Square Error | 87,92356 |
| Mean of Response | 228,5377 |
| Observations (or Sum Wgts) | 146 |

**Pooled t Test**

malignant-benign

Assuming equal variances

| Difference | 65,4521 | t Ratio | 4,497431 |
| --- | --- | --- | --- |
| Std Err Dif | 14,5532 | DF | 144 |
| Upper CL Dif | 94,2176 | Prob > |t| | <,0001* |
| Lower CL Dif | 36,6865 | Prob > t | <,0001* |
| Confidence | 0,95 | Prob < t | 1,0000 |

**Analysis of Variance**

| **Source** | **DF** | **Sum of Squares** | **Mean Square** | **F Ratio** | **Prob > F** |
| --- | --- | --- | --- | --- | --- |
| Type | 1 | 156365,0 | 156365 | 20,2269 | <,0001* |
| Error | 144 | 1113199,5 | 7731 |  |  |
| C. Total | 145 | 1269564,4 |  |  |  |

**Means for Oneway Anova**

| **Level** | **Number** | **Mean** | **Std Error** | **Lower 95%** | **Upper 95%** |
| --- | --- | --- | --- | --- | --- |
| benign | 73 | 195,812 | 10,291 | 175,47 | 216,15 |
| malignant | 73 | 261,264 | 10,291 | 240,92 | 281,60 |

Std Error uses a pooled estimate of error variance

**Oneway Analysis of original-firstorder-Skewness By Type**

**Quantiles**

| **Level** | **Minimum** | **10%** | **25%** | **Median** | **75%** | **90%** | **Maximum** |
| --- | --- | --- | --- | --- | --- | --- | --- |
| benign | -1,67178 | -0,81453 | -0,50424 | -0,20779 | 0,211633 | 0,467691 | 0,996488 |
| malignant | -0,86303 | -0,51683 | -0,13156 | 0,162807 | 0,422845 | 0,624826 | 1,321986 |

**Oneway Anova**

**Summary of Fit**

| Rsquare | 0,090944 |
| --- | --- |
| Adj Rsquare | 0,084631 |
| Root Mean Square Error | 0,471262 |
| Mean of Response | -0,02374 |
| Observations (or Sum Wgts) | 146 |

**Pooled t Test**

malignant-benign

Assuming equal variances

| Difference | 0,296066 | t Ratio | 3,795532 |
| --- | --- | --- | --- |
| Std Err Dif | 0,078004 | DF | 144 |
| Upper CL Dif | 0,450247 | Prob > |t| | 0,0002* |
| Lower CL Dif | 0,141886 | Prob > t | 0,0001* |
| Confidence | 0,95 | Prob < t | 0,9999 |

**Analysis of Variance**

| **Source** | **DF** | **Sum of Squares** | **Mean Square** | **F Ratio** | **Prob > F** |
| --- | --- | --- | --- | --- | --- |
| Type | 1 | 3,199417 | 3,19942 | 14,4061 | 0,0002* |
| Error | 144 | 31,980702 | 0,22209 |  |  |
| C. Total | 145 | 35,180120 |  |  |  |

**Means for Oneway Anova**

| **Level** | **Number** | **Mean** | **Std Error** | **Lower 95%** | **Upper 95%** |
| --- | --- | --- | --- | --- | --- |
| benign | 73 | -0,17177 | 0,05516 | -0,2808 | -0,0627 |
| malignant | 73 | 0,12429 | 0,05516 | 0,0153 | 0,2333 |

Std Error uses a pooled estimate of error variance

**Oneway Analysis of original-firstorder-Uniformity By Type**

**Quantiles**

| **Level** | **Minimum** | **10%** | **25%** | **Median** | **75%** | **90%** | **Maximum** |
| --- | --- | --- | --- | --- | --- | --- | --- |
| benign | 0,058673 | 0,069728 | 0,082865 | 0,102041 | 0,140496 | 0,19216 | 0,242604 |
| malignant | 0,023847 | 0,033939 | 0,038894 | 0,049486 | 0,057554 | 0,072641 | 0,338162 |

**Oneway Anova**

**Summary of Fit**

| Rsquare | 0,357142 |
| --- | --- |
| Adj Rsquare | 0,352678 |
| Root Mean Square Error | 0,042128 |
| Mean of Response | 0,086768 |
| Observations (or Sum Wgts) | 146 |

**Pooled t Test**

malignant-benign

Assuming equal variances

| Difference | -0,06237 | t Ratio | -8,94426 |
| --- | --- | --- | --- |
| Std Err Dif | 0,00697 | DF | 144 |
| Upper CL Dif | -0,04859 | Prob > |t| | <,0001* |
| Lower CL Dif | -0,07615 | Prob > t | 1,0000 |
| Confidence | 0,95 | Prob < t | <,0001* |

**Analysis of Variance**

| **Source** | **DF** | **Sum of Squares** | **Mean Square** | **F Ratio** | **Prob > F** |
| --- | --- | --- | --- | --- | --- |
| Type | 1 | 0,14198093 | 0,141981 | 79,9999 | <,0001* |
| Error | 144 | 0,25556610 | 0,001775 |  |  |
| C. Total | 145 | 0,39754703 |  |  |  |

**Means for Oneway Anova**

| **Level** | **Number** | **Mean** | **Std Error** | **Lower 95%** | **Upper 95%** |
| --- | --- | --- | --- | --- | --- |
| benign | 73 | 0,117953 | 0,00493 | 0,10821 | 0,12770 |
| malignant | 73 | 0,055584 | 0,00493 | 0,04584 | 0,06533 |

Std Error uses a pooled estimate of error variance

**Oneway Analysis of original-firstorder-Median By Type**

**Quantiles**

| **Level** | **Minimum** | **10%** | **25%** | **Median** | **75%** | **90%** | **Maximum** |
| --- | --- | --- | --- | --- | --- | --- | --- |
| benign | 1053 | 1353,2 | 1536,5 | 1710 | 1908,25 | 2042,2 | 2216 |
| malignant | 158 | 627,3 | 789,5 | 913,5 | 1020,25 | 1167,8 | 1412 |

**Oneway Anova**

**Summary of Fit**

| Rsquare | 0,742063 |
| --- | --- |
| Adj Rsquare | 0,740271 |
| Root Mean Square Error | 238,1094 |
| Mean of Response | 1304,373 |
| Observations (or Sum Wgts) | 146 |

**Pooled t Test**

malignant-benign

Assuming equal variances

| Difference | -802,18 | t Ratio | -20,3538 |
| --- | --- | --- | --- |
| Std Err Dif | 39,41 | DF | 144 |
| Upper CL Dif | -724,28 | Prob > |t| | <,0001* |
| Lower CL Dif | -880,09 | Prob > t | 1,0000 |
| Confidence | 0,95 | Prob < t | <,0001* |

**Analysis of Variance**

| **Source** | **DF** | **Sum of Squares** | **Mean Square** | **F Ratio** | **Prob > F** |
| --- | --- | --- | --- | --- | --- |
| Type | 1 | 23487774 | 23487774 | 414,2752 | <,0001* |
| Error | 144 | 8164234 | 56696,067 |  |  |
| C. Total | 145 | 31652008 |  |  |  |

**Means for Oneway Anova**

| **Level** | **Number** | **Mean** | **Std Error** | **Lower 95%** | **Upper 95%** |
| --- | --- | --- | --- | --- | --- |
| benign | 73 | 1705,47 | 27,869 | 1650,4 | 1760,6 |
| malignant | 73 | 903,28 | 27,869 | 848,2 | 958,4 |

Std Error uses a pooled estimate of error variance

**Oneway Analysis of original-firstorder-Energy By Type**

**Quantiles**

| **Level** | **Minimum** | **10%** | **25%** | **Median** | **75%** | **90%** | **Maximum** |
| --- | --- | --- | --- | --- | --- | --- | --- |
| benign | 8805034 | 20275071 | 28284354 | 44304131 | 72214793 | 1,035e+8 | 1,596e+8 |
| malignant | 1236184 | 37099385 | 74228842 | 1,405e+8 | 3,876e+8 | 1,347e+9 | 2,1e+10 |

**Oneway Anova**

**Summary of Fit**

| Rsquare | 0,031629 |
| --- | --- |
| Adj Rsquare | 0,024904 |
| Root Mean Square Error | 1,798e+9 |
| Mean of Response | 3,762e+8 |
| Observations (or Sum Wgts) | 146 |

**Pooled t Test**

malignant-benign

Assuming equal variances

| Difference | 645405588 | t Ratio | 2,16871 |
| --- | --- | --- | --- |
| Std Err Dif | 297598855 | DF | 144 |
| Upper CL Dif | 1,2336e+9 | Prob > |t| | 0,0317* |
| Lower CL Dif | 57179111 | Prob > t | 0,0159* |
| Confidence | 0,95 | Prob < t | 0,9841 |

**Analysis of Variance**

| **Source** | **DF** | **Sum of Squares** | **Mean Square** | **F Ratio** | **Prob > F** |
| --- | --- | --- | --- | --- | --- |
| Type | 1 | 1,5204e+19 | 1,52e+19 | 4,7033 | 0,0317* |
| Error | 144 | 4,655e+20 | 3,233e+18 |  |  |
| C. Total | 145 | 4,807e+20 |  |  |  |

**Means for Oneway Anova**

| **Level** | **Number** | **Mean** | **Std Error** | **Lower 95%** | **Upper 95%** |
| --- | --- | --- | --- | --- | --- |
| benign | 73 | 53500617 | 210434169 | -3,624e+8 | 469439548 |
| malignant | 73 | 698906205 | 210434169 | 282967274 | 1,1148e+9 |

Std Error uses a pooled estimate of error variance

**Oneway Analysis of original-firstorder-RobustMeanAbsoluteDeviation By Type**

**Quantiles**

| **Level** | **Minimum** | **10%** | **25%** | **Median** | **75%** | **90%** | **Maximum** |
| --- | --- | --- | --- | --- | --- | --- | --- |
| benign | 19,03704 | 35,50667 | 58,35012 | 74,84688 | 96,64178 | 126,0331 | 198,7407 |
| malignant | 13,57618 | 73,69214 | 86,11172 | 101,7934 | 126,6437 | 168,2042 | 201,0735 |

**Oneway Anova**

**Summary of Fit**

| Rsquare | 0,127793 |
| --- | --- |
| Adj Rsquare | 0,121736 |
| Root Mean Square Error | 35,8446 |
| Mean of Response | 95,28464 |
| Observations (or Sum Wgts) | 146 |

**Pooled t Test**

malignant-benign

Assuming equal variances

| Difference | 27,2522 | t Ratio | 4,593301 |
| --- | --- | --- | --- |
| Std Err Dif | 5,9330 | DF | 144 |
| Upper CL Dif | 38,9793 | Prob > |t| | <,0001* |
| Lower CL Dif | 15,5251 | Prob > t | <,0001* |
| Confidence | 0,95 | Prob < t | 1,0000 |

**Analysis of Variance**

| **Source** | **DF** | **Sum of Squares** | **Mean Square** | **F Ratio** | **Prob > F** |
| --- | --- | --- | --- | --- | --- |
| Type | 1 | 27108,00 | 27108,0 | 21,0984 | <,0001* |
| Error | 144 | 185016,32 | 1284,8 |  |  |
| C. Total | 145 | 212124,32 |  |  |  |

**Means for Oneway Anova**

| **Level** | **Number** | **Mean** | **Std Error** | **Lower 95%** | **Upper 95%** |
| --- | --- | --- | --- | --- | --- |
| benign | 73 | 81,659 | 4,1953 | 73,37 | 89,95 |
| malignant | 73 | 108,911 | 4,1953 | 100,62 | 117,20 |

Std Error uses a pooled estimate of error variance

**Oneway Analysis of original-firstorder-MeanAbsoluteDeviation By Type**

**Quantiles**

| **Level** | **Minimum** | **10%** | **25%** | **Median** | **75%** | **90%** | **Maximum** |
| --- | --- | --- | --- | --- | --- | --- | --- |
| benign | 37,37278 | 65,66864 | 83,15067 | 116,3269 | 142,1529 | 181,5111 | 358,7769 |
| malignant | 17,37347 | 106,1864 | 121,2304 | 145,1578 | 173,9551 | 228,1611 | 277,1082 |

**Oneway Anova**

**Summary of Fit**

| Rsquare | 0,098804 |
| --- | --- |
| Adj Rsquare | 0,092545 |
| Root Mean Square Error | 48,42613 |
| Mean of Response | 136,762 |
| Observations (or Sum Wgts) | 146 |

**Pooled t Test**

malignant-benign

Assuming equal variances

| Difference | 31,8486 | t Ratio | 3,973358 |
| --- | --- | --- | --- |
| Std Err Dif | 8,0155 | DF | 144 |
| Upper CL Dif | 47,6920 | Prob > |t| | 0,0001* |
| Lower CL Dif | 16,0053 | Prob > t | <,0001* |
| Confidence | 0,95 | Prob < t | 0,9999 |

**Analysis of Variance**

| **Source** | **DF** | **Sum of Squares** | **Mean Square** | **F Ratio** | **Prob > F** |
| --- | --- | --- | --- | --- | --- |
| Type | 1 | 37023,28 | 37023,3 | 15,7876 | 0,0001* |
| Error | 144 | 337692,96 | 2345,1 |  |  |
| C. Total | 145 | 374716,24 |  |  |  |

**Means for Oneway Anova**

| **Level** | **Number** | **Mean** | **Std Error** | **Lower 95%** | **Upper 95%** |
| --- | --- | --- | --- | --- | --- |
| benign | 73 | 120,838 | 5,6678 | 109,63 | 132,04 |
| malignant | 73 | 152,686 | 5,6678 | 141,48 | 163,89 |

Std Error uses a pooled estimate of error variance

**Oneway Analysis of original-firstorder-TotalEnergy By Type**

**Quantiles**

| **Level** | **Minimum** | **10%** | **25%** | **Median** | **75%** | **90%** | **Maximum** |
| --- | --- | --- | --- | --- | --- | --- | --- |
| benign | 87322573 | 1,298e+8 | 2,057e+8 | 2,698e+8 | 4,656e+8 | 6,639e+8 | 1,05e+9 |
| malignant | 7934058 | 2,374e+8 | 5,174e+8 | 8,683e+8 | 2,374e+9 | 8,574e+9 | 1,8e+11 |

**Oneway Anova**

**Summary of Fit**

| Rsquare | 0,025181 |
| --- | --- |
| Adj Rsquare | 0,018412 |
| Root Mean Square Error | 1,51e+10 |
| Mean of Response | 2,765e+9 |
| Observations (or Sum Wgts) | 146 |

**Pooled t Test**

malignant-benign

Assuming equal variances

| Difference | 4,8322e+9 | t Ratio | 1,928673 |
| --- | --- | --- | --- |
| Std Err Dif | 2,5055e+9 | DF | 144 |
| Upper CL Dif | 9,7844e+9 | Prob > |t| | 0,0557 |
| Lower CL Dif | -1,2e+8 | Prob > t | 0,0279* |
| Confidence | 0,95 | Prob < t | 0,9721 |

**Analysis of Variance**

| **Source** | **DF** | **Sum of Squares** | **Mean Square** | **F Ratio** | **Prob > F** |
| --- | --- | --- | --- | --- | --- |
| Type | 1 | 8,5228e+20 | 8,523e+20 | 3,7198 | 0,0557 |
| Error | 144 | 3,2994e+22 | 2,291e+20 |  |  |
| C. Total | 145 | 3,3846e+22 |  |  |  |

**Means for Oneway Anova**

| **Level** | **Number** | **Mean** | **Std Error** | **Lower 95%** | **Upper 95%** |
| --- | --- | --- | --- | --- | --- |
| benign | 73 | 349351958 | 1,7716e+9 | -3,152e+9 | 3,8511e+9 |
| malignant | 73 | 5,1816e+9 | 1,7716e+9 | 1,6798e+9 | 8,6833e+9 |

Std Error uses a pooled estimate of error variance

**Oneway Analysis of original-firstorder-Maximum By Type**

**Quantiles**

| **Level** | **Minimum** | **10%** | **25%** | **Median** | **75%** | **90%** | **Maximum** |
| --- | --- | --- | --- | --- | --- | --- | --- |
| benign | 1362 | 1578,4 | 1750,5 | 1968 | 2151 | 2277,4 | 2512 |
| malignant | 206 | 1065,6 | 1168,5 | 1389 | 1592,5 | 1948,6 | 2492 |

**Oneway Anova**

**Summary of Fit**

| Rsquare | 0,414628 |
| --- | --- |
| Adj Rsquare | 0,410563 |
| Root Mean Square Error | 311,8216 |
| Mean of Response | 1688,205 |
| Observations (or Sum Wgts) | 146 |

**Pooled t Test**

malignant-benign

Assuming equal variances

| Difference | -521,26 | t Ratio | -10,0994 |
| --- | --- | --- | --- |
| Std Err Dif | 51,61 | DF | 144 |
| Upper CL Dif | -419,24 | Prob > |t| | <,0001* |
| Lower CL Dif | -623,28 | Prob > t | 1,0000 |
| Confidence | 0,95 | Prob < t | <,0001* |

**Analysis of Variance**

| **Source** | **DF** | **Sum of Squares** | **Mean Square** | **F Ratio** | **Prob > F** |
| --- | --- | --- | --- | --- | --- |
| Type | 1 | 9917498 | 9917498 | 101,9976 | <,0001* |
| Error | 144 | 14001506 | 97233 |  |  |
| C. Total | 145 | 23919004 |  |  |  |

**Means for Oneway Anova**

| **Level** | **Number** | **Mean** | **Std Error** | **Lower 95%** | **Upper 95%** |
| --- | --- | --- | --- | --- | --- |
| benign | 73 | 1948,84 | 36,496 | 1876,7 | 2021,0 |
| malignant | 73 | 1427,58 | 36,496 | 1355,4 | 1499,7 |

Std Error uses a pooled estimate of error variance

**Oneway Analysis of original-firstorder-RootMeanSquared By Type**

**Quantiles**

| **Level** | **Minimum** | **10%** | **25%** | **Median** | **75%** | **90%** | **Maximum** |
| --- | --- | --- | --- | --- | --- | --- | --- |
| benign | 1084,08 | 1335,449 | 1530,601 | 1712,801 | 1912,956 | 2036,328 | 2229,811 |
| malignant | 162,1783 | 667,6896 | 801,6688 | 925,0001 | 1072,847 | 1179,382 | 1410,001 |

**Oneway Anova**

**Summary of Fit**

| Rsquare | 0,735896 |
| --- | --- |
| Adj Rsquare | 0,734062 |
| Root Mean Square Error | 233,1881 |
| Mean of Response | 1317,673 |
| Observations (or Sum Wgts) | 146 |

**Pooled t Test**

malignant-benign

Assuming equal variances

| Difference | -773,15 | t Ratio | -20,031 |
| --- | --- | --- | --- |
| Std Err Dif | 38,60 | DF | 144 |
| Upper CL Dif | -696,86 | Prob > |t| | <,0001* |
| Lower CL Dif | -849,44 | Prob > t | 1,0000 |
| Confidence | 0,95 | Prob < t | <,0001* |

**Analysis of Variance**

| **Source** | **DF** | **Sum of Squares** | **Mean Square** | **F Ratio** | **Prob > F** |
| --- | --- | --- | --- | --- | --- |
| Type | 1 | 21818111 | 21818111 | 401,2403 | <,0001* |
| Error | 144 | 7830240 | 54376,67 |  |  |
| C. Total | 145 | 29648352 |  |  |  |

**Means for Oneway Anova**

| **Level** | **Number** | **Mean** | **Std Error** | **Lower 95%** | **Upper 95%** |
| --- | --- | --- | --- | --- | --- |
| benign | 73 | 1704,25 | 27,293 | 1650,3 | 1758,2 |
| malignant | 73 | 931,10 | 27,293 | 877,2 | 985,0 |

Std Error uses a pooled estimate of error variance

**Oneway Analysis of original-firstorder-90Percentile By Type**

**Quantiles**

| **Level** | **Minimum** | **10%** | **25%** | **Median** | **75%** | **90%** | **Maximum** |
| --- | --- | --- | --- | --- | --- | --- | --- |
| benign | 1266,8 | 1502,48 | 1670,7 | 1890,4 | 2084,3 | 2190,2 | 2435 |
| malignant | 193 | 871,92 | 995,6 | 1150,9 | 1315,85 | 1460,6 | 1699,2 |

**Oneway Anova**

**Summary of Fit**

| Rsquare | 0,661302 |
| --- | --- |
| Adj Rsquare | 0,65895 |
| Root Mean Square Error | 257,2602 |
| Mean of Response | 1510,425 |
| Observations (or Sum Wgts) | 146 |

**Pooled t Test**

malignant-benign

Assuming equal variances

| Difference | -714,00 | t Ratio | -16,7677 |
| --- | --- | --- | --- |
| Std Err Dif | 42,58 | DF | 144 |
| Upper CL Dif | -629,84 | Prob > |t| | <,0001* |
| Lower CL Dif | -798,17 | Prob > t | 1,0000 |
| Confidence | 0,95 | Prob < t | <,0001* |

**Analysis of Variance**

| **Source** | **DF** | **Sum of Squares** | **Mean Square** | **F Ratio** | **Prob > F** |
| --- | --- | --- | --- | --- | --- |
| Type | 1 | 18607768 | 18607768 | 281,1571 | <,0001* |
| Error | 144 | 9530325 | 66182,815 |  |  |
| C. Total | 145 | 28138094 |  |  |  |

**Means for Oneway Anova**

| **Level** | **Number** | **Mean** | **Std Error** | **Lower 95%** | **Upper 95%** |
| --- | --- | --- | --- | --- | --- |
| benign | 73 | 1867,43 | 30,110 | 1807,9 | 1926,9 |
| malignant | 73 | 1153,42 | 30,110 | 1093,9 | 1212,9 |

Std Error uses a pooled estimate of error variance

**Oneway Analysis of original-firstorder-Minimum By Type**

**Quantiles**

| **Level** | **Minimum** | **10%** | **25%** | **Median** | **75%** | **90%** | **Maximum** |
| --- | --- | --- | --- | --- | --- | --- | --- |
| benign | 352 | 1054,2 | 1203,5 | 1402 | 1617,5 | 1801,4 | 1977 |
| malignant | 0 | 24,6 | 286,5 | 441 | 612 | 745,6 | 995 |

**Oneway Anova**

**Summary of Fit**

| Rsquare | 0,763378 |
| --- | --- |
| Adj Rsquare | 0,761734 |
| Root Mean Square Error | 271,6607 |
| Mean of Response | 915,274 |
| Observations (or Sum Wgts) | 146 |

**Pooled t Test**

malignant-benign

Assuming equal variances

| Difference | -969,2 | t Ratio | -21,5538 |
| --- | --- | --- | --- |
| Std Err Dif | 45,0 | DF | 144 |
| Upper CL Dif | -880,3 | Prob > |t| | <,0001* |
| Lower CL Dif | -1058,1 | Prob > t | 1,0000 |
| Confidence | 0,95 | Prob < t | <,0001* |

**Analysis of Variance**

| **Source** | **DF** | **Sum of Squares** | **Mean Square** | **F Ratio** | **Prob > F** |
| --- | --- | --- | --- | --- | --- |
| Type | 1 | 34284675 | 34284675 | 464,5648 | <,0001* |
| Error | 144 | 10627136 | 73799,558 |  |  |
| C. Total | 145 | 44911811 |  |  |  |

**Means for Oneway Anova**

| **Level** | **Number** | **Mean** | **Std Error** | **Lower 95%** | **Upper 95%** |
| --- | --- | --- | --- | --- | --- |
| benign | 73 | 1399,86 | 31,795 | 1337,0 | 1462,7 |
| malignant | 73 | 430,68 | 31,795 | 367,8 | 493,5 |

Std Error uses a pooled estimate of error variance

**Oneway Analysis of original-firstorder-Entropy By Type**

**Quantiles**

| **Level** | **Minimum** | **10%** | **25%** | **Median** | **75%** | **90%** | **Maximum** |
| --- | --- | --- | --- | --- | --- | --- | --- |
| benign | 2,170951 | 2,508656 | 2,926569 | 3,419382 | 3,720611 | 3,997876 | 4,23327 |
| malignant | 1,658936 | 3,965215 | 4,339824 | 4,586009 | 4,922842 | 5,125856 | 5,585106 |

**Oneway Anova**

**Summary of Fit**

| Rsquare | 0,555598 |
| --- | --- |
| Adj Rsquare | 0,552512 |
| Root Mean Square Error | 0,553221 |
| Mean of Response | 3,931144 |
| Observations (or Sum Wgts) | 146 |

**Pooled t Test**

malignant-benign

Assuming equal variances

| Difference | 1,22864 | t Ratio | 13,41755 |
| --- | --- | --- | --- |
| Std Err Dif | 0,09157 | DF | 144 |
| Upper CL Dif | 1,40964 | Prob > |t| | <,0001* |
| Lower CL Dif | 1,04765 | Prob > t | <,0001* |
| Confidence | 0,95 | Prob < t | 1,0000 |

**Analysis of Variance**

| **Source** | **DF** | **Sum of Squares** | **Mean Square** | **F Ratio** | **Prob > F** |
| --- | --- | --- | --- | --- | --- |
| Type | 1 | 55,099030 | 55,0990 | 180,0308 | <,0001* |
| Error | 144 | 44,071689 | 0,3061 |  |  |
| C. Total | 145 | 99,170720 |  |  |  |

**Means for Oneway Anova**

| **Level** | **Number** | **Mean** | **Std Error** | **Lower 95%** | **Upper 95%** |
| --- | --- | --- | --- | --- | --- |
| benign | 73 | 3,31682 | 0,06475 | 3,1888 | 3,4448 |
| malignant | 73 | 4,54546 | 0,06475 | 4,4175 | 4,6734 |

Std Error uses a pooled estimate of error variance

**Oneway Analysis of original-firstorder-Range By Type**

**Quantiles**

| **Level** | **Minimum** | **10%** | **25%** | **Median** | **75%** | **90%** | **Maximum** |
| --- | --- | --- | --- | --- | --- | --- | --- |
| benign | 191 | 292,4 | 379,5 | 539 | 674 | 805,4 | 1829 |
| malignant | 76 | 553,2 | 724 | 889 | 1221,5 | 1549,6 | 2492 |

**Oneway Anova**

**Summary of Fit**

| Rsquare | 0,314275 |
| --- | --- |
| Adj Rsquare | 0,309513 |
| Root Mean Square Error | 333,1071 |
| Mean of Response | 772,9315 |
| Observations (or Sum Wgts) | 146 |

**Pooled t Test**

malignant-benign

Assuming equal variances

| Difference | 447,918 | t Ratio | 8,123831 |
| --- | --- | --- | --- |
| Std Err Dif | 55,136 | DF | 144 |
| Upper CL Dif | 556,899 | Prob > |t| | <,0001* |
| Lower CL Dif | 338,937 | Prob > t | <,0001* |
| Confidence | 0,95 | Prob < t | 1,0000 |

**Analysis of Variance**

| **Source** | **DF** | **Sum of Squares** | **Mean Square** | **F Ratio** | **Prob > F** |
| --- | --- | --- | --- | --- | --- |
| Type | 1 | 7323008 | 7323008 | 65,9966 | <,0001* |
| Error | 144 | 15978289 | 110960 |  |  |
| C. Total | 145 | 23301297 |  |  |  |

**Means for Oneway Anova**

| **Level** | **Number** | **Mean** | **Std Error** | **Lower 95%** | **Upper 95%** |
| --- | --- | --- | --- | --- | --- |
| benign | 73 | 548,973 | 38,987 | 471,91 | 626,0 |
| malignant | 73 | 996,890 | 38,987 | 919,83 | 1074,0 |

Std Error uses a pooled estimate of error variance

**Oneway Analysis of original-firstorder-Variance By Type**

**Quantiles**

| **Level** | **Minimum** | **10%** | **25%** | **Median** | **75%** | **90%** | **Maximum** |
| --- | --- | --- | --- | --- | --- | --- | --- |
| benign | 2144,13 | 7693,344 | 10310,43 | 20152,91 | 30976,2 | 46599,99 | 239185,9 |
| malignant | 428,7225 | 16402,44 | 23447,26 | 32581,3 | 46179,03 | 75265,68 | 116634,2 |

**Oneway Anova**

**Summary of Fit**

| Rsquare | 0,054648 |
| --- | --- |
| Adj Rsquare | 0,048083 |
| Root Mean Square Error | 26434,59 |
| Mean of Response | 32369,26 |
| Observations (or Sum Wgts) | 146 |

**Pooled t Test**

malignant-benign

Assuming equal variances

| Difference | 12624,1 | t Ratio | 2,885181 |
| --- | --- | --- | --- |
| Std Err Dif | 4375,5 | DF | 144 |
| Upper CL Dif | 21272,5 | Prob > |t| | 0,0045* |
| Lower CL Dif | 3975,6 | Prob > t | 0,0023* |
| Confidence | 0,95 | Prob < t | 0,9977 |

**Analysis of Variance**

| **Source** | **DF** | **Sum of Squares** | **Mean Square** | **F Ratio** | **Prob > F** |
| --- | --- | --- | --- | --- | --- |
| Type | 1 | 5816894254 | 5,8169e+9 | 8,3243 | 0,0045* |
| Error | 144 | 1,0063e+11 | 698787293 |  |  |
| C. Total | 145 | 1,0644e+11 |  |  |  |

**Means for Oneway Anova**

| **Level** | **Number** | **Mean** | **Std Error** | **Lower 95%** | **Upper 95%** |
| --- | --- | --- | --- | --- | --- |
| benign | 73 | 26057,2 | 3093,9 | 19942 | 32173 |
| malignant | 73 | 38681,3 | 3093,9 | 32566 | 44797 |

Std Error uses a pooled estimate of error variance

**Oneway Analysis of original-firstorder-10Percentile By Type**

**Quantiles**

| **Level** | **Minimum** | **10%** | **25%** | **Median** | **75%** | **90%** | **Maximum** |
| --- | --- | --- | --- | --- | --- | --- | --- |
| benign | 947,6 | 1187,58 | 1319,65 | 1509,6 | 1708,5 | 1874,08 | 2075,5 |
| malignant | 1,8 | 462,2 | 576,2 | 680,1 | 802,05 | 901,84 | 1143,2 |

**Oneway Anova**

**Summary of Fit**

| Rsquare | 0,77477 |
| --- | --- |
| Adj Rsquare | 0,773206 |
| Root Mean Square Error | 229,7127 |
| Mean of Response | 1096,053 |
| Observations (or Sum Wgts) | 146 |

**Pooled t Test**

malignant-benign

Assuming equal variances

| Difference | -846,24 | t Ratio | -22,2564 |
| --- | --- | --- | --- |
| Std Err Dif | 38,02 | DF | 144 |
| Upper CL Dif | -771,08 | Prob > |t| | <,0001* |
| Lower CL Dif | -921,39 | Prob > t | 1,0000 |
| Confidence | 0,95 | Prob < t | <,0001* |

**Analysis of Variance**

| **Source** | **DF** | **Sum of Squares** | **Mean Square** | **F Ratio** | **Prob > F** |
| --- | --- | --- | --- | --- | --- |
| Type | 1 | 26138356 | 26138356 | 495,3455 | <,0001* |
| Error | 144 | 7598582 | 52767,927 |  |  |
| C. Total | 145 | 33736938 |  |  |  |

**Means for Oneway Anova**

| **Level** | **Number** | **Mean** | **Std Error** | **Lower 95%** | **Upper 95%** |
| --- | --- | --- | --- | --- | --- |
| benign | 73 | 1519,17 | 26,886 | 1466,0 | 1572,3 |
| malignant | 73 | 672,93 | 26,886 | 619,8 | 726,1 |

Std Error uses a pooled estimate of error variance

**Oneway Analysis of original-firstorder-Kurtosis By Type**

**Quantiles**

| **Level** | **Minimum** | **10%** | **25%** | **Median** | **75%** | **90%** | **Maximum** |
| --- | --- | --- | --- | --- | --- | --- | --- |
| benign | 1,317372 | 1,783919 | 2,12007 | 2,516943 | 3,050287 | 3,659619 | 5,068563 |
| malignant | 1,46581 | 2,096691 | 2,410484 | 2,735136 | 3,504402 | 4,026172 | 5,654035 |

**Oneway Anova**

**Summary of Fit**

| Rsquare | 0,045253 |
| --- | --- |
| Adj Rsquare | 0,038623 |
| Root Mean Square Error | 0,762206 |
| Mean of Response | 2,794404 |
| Observations (or Sum Wgts) | 146 |

**Pooled t Test**

malignant-benign

Assuming equal variances

| Difference | 0,329599 | t Ratio | 2,612523 |
| --- | --- | --- | --- |
| Std Err Dif | 0,126161 | DF | 144 |
| Upper CL Dif | 0,578966 | Prob > |t| | 0,0099* |
| Lower CL Dif | 0,080232 | Prob > t | 0,0050* |
| Confidence | 0,95 | Prob < t | 0,9950 |

**Analysis of Variance**

| **Source** | **DF** | **Sum of Squares** | **Mean Square** | **F Ratio** | **Prob > F** |
| --- | --- | --- | --- | --- | --- |
| Type | 1 | 3,965195 | 3,96520 | 6,8253 | 0,0099* |
| Error | 144 | 83,657875 | 0,58096 |  |  |
| C. Total | 145 | 87,623070 |  |  |  |

**Means for Oneway Anova**

| **Level** | **Number** | **Mean** | **Std Error** | **Lower 95%** | **Upper 95%** |
| --- | --- | --- | --- | --- | --- |
| benign | 73 | 2,62960 | 0,08921 | 2,4533 | 2,8059 |
| malignant | 73 | 2,95920 | 0,08921 | 2,7829 | 3,1355 |

Std Error uses a pooled estimate of error variance

**Oneway Analysis of original-firstorder-Mean By Type**

**Quantiles**

| **Level** | **Minimum** | **10%** | **25%** | **Median** | **75%** | **90%** | **Maximum** |
| --- | --- | --- | --- | --- | --- | --- | --- |
| benign | 1075,611 | 1330,413 | 1525,087 | 1696,333 | 1906,711 | 2031,786 | 2226,167 |
| malignant | 160,8511 | 651,4815 | 788,3167 | 908,0622 | 1046,749 | 1157,313 | 1397,189 |

**Oneway Anova**

**Summary of Fit**

| Rsquare | 0,741331 |
| --- | --- |
| Adj Rsquare | 0,739535 |
| Root Mean Square Error | 233,9283 |
| Mean of Response | 1303,191 |
| Observations (or Sum Wgts) | 146 |

**Pooled t Test**

malignant-benign

Assuming equal variances

| Difference | -786,60 | t Ratio | -20,3149 |
| --- | --- | --- | --- |
| Std Err Dif | 38,72 | DF | 144 |
| Upper CL Dif | -710,06 | Prob > |t| | <,0001* |
| Lower CL Dif | -863,13 | Prob > t | 1,0000 |
| Confidence | 0,95 | Prob < t | <,0001* |

**Analysis of Variance**

| **Source** | **DF** | **Sum of Squares** | **Mean Square** | **F Ratio** | **Prob > F** |
| --- | --- | --- | --- | --- | --- |
| Type | 1 | 22583799 | 22583799 | 412,6970 | <,0001* |
| Error | 144 | 7880036 | 54722,47 |  |  |
| C. Total | 145 | 30463835 |  |  |  |

**Means for Oneway Anova**

| **Level** | **Number** | **Mean** | **Std Error** | **Lower 95%** | **Upper 95%** |
| --- | --- | --- | --- | --- | --- |
| benign | 73 | 1696,49 | 27,379 | 1642,4 | 1750,6 |
| malignant | 73 | 909,89 | 27,379 | 855,8 | 964,0 |

Std Error uses a pooled estimate of error variance

**Oneway Analysis of original-glrlm-ShortRunLowGrayLevelEmphasis By Type**

**Quantiles**

| **Level** | **Minimum** | **10%** | **25%** | **Median** | **75%** | **90%** | **Maximum** |
| --- | --- | --- | --- | --- | --- | --- | --- |
| benign | 0,028315 | 0,049962 | 0,060713 | 0,085627 | 0,120058 | 0,176339 | 0,243073 |
| malignant | 0,000964 | 0,00274 | 0,007531 | 0,014922 | 0,030779 | 0,050347 | 0,399814 |

**Oneway Anova**

**Summary of Fit**

| Rsquare | 0,346684 |
| --- | --- |
| Adj Rsquare | 0,342147 |
| Root Mean Square Error | 0,048621 |
| Mean of Response | 0,061962 |
| Observations (or Sum Wgts) | 146 |

**Pooled t Test**

malignant-benign

Assuming equal variances

| Difference | -0,07035 | t Ratio | -8,74152 |
| --- | --- | --- | --- |
| Std Err Dif | 0,00805 | DF | 144 |
| Upper CL Dif | -0,05444 | Prob > |t| | <,0001* |
| Lower CL Dif | -0,08626 | Prob > t | 1,0000 |
| Confidence | 0,95 | Prob < t | <,0001* |

**Analysis of Variance**

| **Source** | **DF** | **Sum of Squares** | **Mean Square** | **F Ratio** | **Prob > F** |
| --- | --- | --- | --- | --- | --- |
| Type | 1 | 0,18064155 | 0,180642 | 76,4141 | <,0001* |
| Error | 144 | 0,34041321 | 0,002364 |  |  |
| C. Total | 145 | 0,52105476 |  |  |  |

**Means for Oneway Anova**

| **Level** | **Number** | **Mean** | **Std Error** | **Lower 95%** | **Upper 95%** |
| --- | --- | --- | --- | --- | --- |
| benign | 73 | 0,097137 | 0,00569 | 0,08589 | 0,10838 |
| malignant | 73 | 0,026787 | 0,00569 | 0,01554 | 0,03803 |

Std Error uses a pooled estimate of error variance

**Oneway Analysis of original-glrlm-GrayLevelVariance By Type**

**Quantiles**

| **Level** | **Minimum** | **10%** | **25%** | **Median** | **75%** | **90%** | **Maximum** |
| --- | --- | --- | --- | --- | --- | --- | --- |
| benign | 4,020392 | 12,35681 | 17,2581 | 31,83633 | 49,90255 | 75,73577 | 385,0203 |
| malignant | 0,661157 | 26,68951 | 38,01009 | 52,79693 | 74,90802 | 120,7085 | 187,5598 |

**Oneway Anova**

**Summary of Fit**

| Rsquare | 0,05493 |
| --- | --- |
| Adj Rsquare | 0,048367 |
| Root Mean Square Error | 42,29531 |
| Mean of Response | 52,20775 |
| Observations (or Sum Wgts) | 146 |

**Pooled t Test**

malignant-benign

Assuming equal variances

| Difference | 20,2534 | t Ratio | 2,893028 |
| --- | --- | --- | --- |
| Std Err Dif | 7,0008 | DF | 144 |
| Upper CL Dif | 34,0910 | Prob > |t| | 0,0044* |
| Lower CL Dif | 6,4159 | Prob > t | 0,0022* |
| Confidence | 0,95 | Prob < t | 0,9978 |

**Analysis of Variance**

| **Source** | **DF** | **Sum of Squares** | **Mean Square** | **F Ratio** | **Prob > F** |
| --- | --- | --- | --- | --- | --- |
| Type | 1 | 14972,35 | 14972,3 | 8,3696 | 0,0044* |
| Error | 144 | 257600,63 | 1788,9 |  |  |
| C. Total | 145 | 272572,98 |  |  |  |

**Means for Oneway Anova**

| **Level** | **Number** | **Mean** | **Std Error** | **Lower 95%** | **Upper 95%** |
| --- | --- | --- | --- | --- | --- |
| benign | 73 | 42,0810 | 4,9503 | 32,296 | 51,866 |
| malignant | 73 | 62,3345 | 4,9503 | 52,550 | 72,119 |

Std Error uses a pooled estimate of error variance

**Oneway Analysis of original-glrlm-LowGrayLevelRunEmphasis By Type**

**Quantiles**

| **Level** | **Minimum** | **10%** | **25%** | **Median** | **75%** | **90%** | **Maximum** |
| --- | --- | --- | --- | --- | --- | --- | --- |
| benign | 0,028366 | 0,050149 | 0,060975 | 0,08831 | 0,123643 | 0,178933 | 0,306875 |
| malignant | 0,00099 | 0,002811 | 0,007643 | 0,015016 | 0,031605 | 0,050737 | 0,46662 |

**Oneway Anova**

**Summary of Fit**

| Rsquare | 0,304503 |
| --- | --- |
| Adj Rsquare | 0,299673 |
| Root Mean Square Error | 0,054265 |
| Mean of Response | 0,063754 |
| Observations (or Sum Wgts) | 146 |

**Pooled t Test**

malignant-benign

Assuming equal variances

| Difference | -0,07132 | t Ratio | -7,94016 |
| --- | --- | --- | --- |
| Std Err Dif | 0,00898 | DF | 144 |
| Upper CL Dif | -0,05357 | Prob > |t| | <,0001* |
| Lower CL Dif | -0,08907 | Prob > t | 1,0000 |
| Confidence | 0,95 | Prob < t | <,0001* |

**Analysis of Variance**

| **Source** | **DF** | **Sum of Squares** | **Mean Square** | **F Ratio** | **Prob > F** |
| --- | --- | --- | --- | --- | --- |
| Type | 1 | 0,18565433 | 0,185654 | 63,0461 | <,0001* |
| Error | 144 | 0,42404260 | 0,002945 |  |  |
| C. Total | 145 | 0,60969693 |  |  |  |

**Means for Oneway Anova**

| **Level** | **Number** | **Mean** | **Std Error** | **Lower 95%** | **Upper 95%** |
| --- | --- | --- | --- | --- | --- |
| benign | 73 | 0,099413 | 0,00635 | 0,08686 | 0,11197 |
| malignant | 73 | 0,028094 | 0,00635 | 0,01554 | 0,04065 |

Std Error uses a pooled estimate of error variance

**Oneway Analysis of original-glrlm-GrayLevelNonUniformityNormalized By Type**

**Quantiles**

| **Level** | **Minimum** | **10%** | **25%** | **Median** | **75%** | **90%** | **Maximum** |
| --- | --- | --- | --- | --- | --- | --- | --- |
| benign | 0,058143 | 0,069364 | 0,081187 | 0,1 | 0,138534 | 0,18682 | 0,234568 |
| malignant | 0,023779 | 0,03361 | 0,038556 | 0,048312 | 0,057224 | 0,072286 | 0,336305 |

**Oneway Anova**

**Summary of Fit**

| Rsquare | 0,353426 |
| --- | --- |
| Adj Rsquare | 0,348936 |
| Root Mean Square Error | 0,040935 |
| Mean of Response | 0,085076 |
| Observations (or Sum Wgts) | 146 |

**Pooled t Test**

malignant-benign

Assuming equal variances

| Difference | -0,06011 | t Ratio | -8,872 |
| --- | --- | --- | --- |
| Std Err Dif | 0,00678 | DF | 144 |
| Upper CL Dif | -0,04672 | Prob > |t| | <,0001* |
| Lower CL Dif | -0,07351 | Prob > t | 1,0000 |
| Confidence | 0,95 | Prob < t | <,0001* |

**Analysis of Variance**

| **Source** | **DF** | **Sum of Squares** | **Mean Square** | **F Ratio** | **Prob > F** |
| --- | --- | --- | --- | --- | --- |
| Type | 1 | 0,13189565 | 0,131896 | 78,7124 | <,0001* |
| Error | 144 | 0,24129594 | 0,001676 |  |  |
| C. Total | 145 | 0,37319159 |  |  |  |

**Means for Oneway Anova**

| **Level** | **Number** | **Mean** | **Std Error** | **Lower 95%** | **Upper 95%** |
| --- | --- | --- | --- | --- | --- |
| benign | 73 | 0,115132 | 0,00479 | 0,10566 | 0,12460 |
| malignant | 73 | 0,055019 | 0,00479 | 0,04555 | 0,06449 |

Std Error uses a pooled estimate of error variance

**Oneway Analysis of original-glrlm-RunVariance By Type**

**Quantiles**

| **Level** | **Minimum** | **10%** | **25%** | **Median** | **75%** | **90%** | **Maximum** |
| --- | --- | --- | --- | --- | --- | --- | --- |
| benign | 0 | 0 | 0,018465 | 0,031111 | 0,054337 | 0,086184 | 0,159377 |
| malignant | 0,01402 | 0,022752 | 0,027906 | 0,039822 | 0,045491 | 0,050616 | 0,262458 |

**Oneway Anova**

**Summary of Fit**

| Rsquare | 0,001276 |
| --- | --- |
| Adj Rsquare | -0,00566 |
| Root Mean Square Error | 0,030328 |
| Mean of Response | 0,039087 |
| Observations (or Sum Wgts) | 146 |

**Pooled t Test**

malignant-benign

Assuming equal variances

| Difference | 0,00215 | t Ratio | 0,428851 |
| --- | --- | --- | --- |
| Std Err Dif | 0,00502 | DF | 144 |
| Upper CL Dif | 0,01208 | Prob > |t| | 0,6687 |
| Lower CL Dif | -0,00777 | Prob > t | 0,3343 |
| Confidence | 0,95 | Prob < t | 0,6657 |

**Analysis of Variance**

| **Source** | **DF** | **Sum of Squares** | **Mean Square** | **F Ratio** | **Prob > F** |
| --- | --- | --- | --- | --- | --- |
| Type | 1 | 0,00016916 | 0,000169 | 0,1839 | 0,6687 |
| Error | 144 | 0,13244988 | 0,000920 |  |  |
| C. Total | 145 | 0,13261904 |  |  |  |

**Means for Oneway Anova**

| **Level** | **Number** | **Mean** | **Std Error** | **Lower 95%** | **Upper 95%** |
| --- | --- | --- | --- | --- | --- |
| benign | 73 | 0,038010 | 0,00355 | 0,03099 | 0,04503 |
| malignant | 73 | 0,040163 | 0,00355 | 0,03315 | 0,04718 |

Std Error uses a pooled estimate of error variance

**Oneway Analysis of original-glrlm-GrayLevelNonUniformity By Type**

**Quantiles**

| **Level** | **Minimum** | **10%** | **25%** | **Median** | **75%** | **90%** | **Maximum** |
| --- | --- | --- | --- | --- | --- | --- | --- |
| benign | 1 | 1,205714 | 1,36978 | 1,6 | 2,014793 | 2,499878 | 3,723529 |
| malignant | 1,852781 | 2,858113 | 4,570033 | 7,43442 | 14,94735 | 55,83482 | 405,8616 |

**Oneway Anova**

**Summary of Fit**

| Rsquare | 0,076646 |
| --- | --- |
| Adj Rsquare | 0,070234 |
| Root Mean Square Error | 37,10221 |
| Mean of Response | 12,34537 |
| Observations (or Sum Wgts) | 146 |

**Pooled t Test**

malignant-benign

Assuming equal variances

| Difference | 21,2322 | t Ratio | 3,457337 |
| --- | --- | --- | --- |
| Std Err Dif | 6,1412 | DF | 144 |
| Upper CL Dif | 33,3707 | Prob > |t| | 0,0007* |
| Lower CL Dif | 9,0937 | Prob > t | 0,0004* |
| Confidence | 0,95 | Prob < t | 0,9996 |

**Analysis of Variance**

| **Source** | **DF** | **Sum of Squares** | **Mean Square** | **F Ratio** | **Prob > F** |
| --- | --- | --- | --- | --- | --- |
| Type | 1 | 16454,43 | 16454,4 | 11,9532 | 0,0007* |
| Error | 144 | 198226,62 | 1376,6 |  |  |
| C. Total | 145 | 214681,05 |  |  |  |

**Means for Oneway Anova**

| **Level** | **Number** | **Mean** | **Std Error** | **Lower 95%** | **Upper 95%** |
| --- | --- | --- | --- | --- | --- |
| benign | 73 | 1,7293 | 4,3425 | -6,85 | 10,313 |
| malignant | 73 | 22,9615 | 4,3425 | 14,38 | 31,545 |

Std Error uses a pooled estimate of error variance

**Oneway Analysis of original-glrlm-LongRunEmphasis By Type**

**Quantiles**

| **Level** | **Minimum** | **10%** | **25%** | **Median** | **75%** | **90%** | **Maximum** |
| --- | --- | --- | --- | --- | --- | --- | --- |
| benign | 1 | 1 | 1,059028 | 1,107143 | 1,173906 | 1,251696 | 1,50386 |
| malignant | 1,044389 | 1,070129 | 1,085139 | 1,116642 | 1,137356 | 1,149361 | 1,936815 |

**Oneway Anova**

**Summary of Fit**

| Rsquare | 7,425e-5 |
| --- | --- |
| Adj Rsquare | -0,00687 |
| Root Mean Square Error | 0,103115 |
| Mean of Response | 1,12288 |
| Observations (or Sum Wgts) | 146 |

**Pooled t Test**

malignant-benign

Assuming equal variances

| Difference | -0,00176 | t Ratio | -0,10341 |
| --- | --- | --- | --- |
| Std Err Dif | 0,01707 | DF | 144 |
| Upper CL Dif | 0,03197 | Prob > |t| | 0,9178 |
| Lower CL Dif | -0,03550 | Prob > t | 0,5411 |
| Confidence | 0,95 | Prob < t | 0,4589 |

**Analysis of Variance**

| **Source** | **DF** | **Sum of Squares** | **Mean Square** | **F Ratio** | **Prob > F** |
| --- | --- | --- | --- | --- | --- |
| Type | 1 | 0,0001137 | 0,000114 | 0,0107 | 0,9178 |
| Error | 144 | 1,5310998 | 0,010633 |  |  |
| C. Total | 145 | 1,5312135 |  |  |  |

**Means for Oneway Anova**

| **Level** | **Number** | **Mean** | **Std Error** | **Lower 95%** | **Upper 95%** |
| --- | --- | --- | --- | --- | --- |
| benign | 73 | 1,12376 | 0,01207 | 1,0999 | 1,1476 |
| malignant | 73 | 1,12200 | 0,01207 | 1,0981 | 1,1459 |

Std Error uses a pooled estimate of error variance

**Oneway Analysis of original-glrlm-ShortRunHighGrayLevelEmphasis By Type**

**Quantiles**

| **Level** | **Minimum** | **10%** | **25%** | **Median** | **75%** | **90%** | **Maximum** |
| --- | --- | --- | --- | --- | --- | --- | --- |
| benign | 21,85417 | 51,42449 | 97,95654 | 187,1818 | 288,6707 | 432,5687 | 3152,615 |
| malignant | 3,600202 | 175,8904 | 251,8647 | 405,6935 | 684,1099 | 984,348 | 2018,638 |

**Oneway Anova**

**Summary of Fit**

| Rsquare | 0,118128 |
| --- | --- |
| Adj Rsquare | 0,112003 |
| Root Mean Square Error | 358,4491 |
| Mean of Response | 374,226 |
| Observations (or Sum Wgts) | 146 |

**Pooled t Test**

malignant-benign

Assuming equal variances

| Difference | 260,576 | t Ratio | 4,391915 |
| --- | --- | --- | --- |
| Std Err Dif | 59,331 | DF | 144 |
| Upper CL Dif | 377,848 | Prob > |t| | <,0001* |
| Lower CL Dif | 143,304 | Prob > t | <,0001* |
| Confidence | 0,95 | Prob < t | 1,0000 |

**Analysis of Variance**

| **Source** | **DF** | **Sum of Squares** | **Mean Square** | **F Ratio** | **Prob > F** |
| --- | --- | --- | --- | --- | --- |
| Type | 1 | 2478352 | 2478352 | 19,2889 | <,0001* |
| Error | 144 | 18501954 | 128486 |  |  |
| C. Total | 145 | 20980306 |  |  |  |

**Means for Oneway Anova**

| **Level** | **Number** | **Mean** | **Std Error** | **Lower 95%** | **Upper 95%** |
| --- | --- | --- | --- | --- | --- |
| benign | 73 | 243,938 | 41,953 | 161,01 | 326,86 |
| malignant | 73 | 504,514 | 41,953 | 421,59 | 587,44 |

Std Error uses a pooled estimate of error variance

**Oneway Analysis of original-glrlm-RunLengthNonUniformity By Type**

**Quantiles**

| **Level** | **Minimum** | **10%** | **25%** | **Median** | **75%** | **90%** | **Maximum** |
| --- | --- | --- | --- | --- | --- | --- | --- |
| benign | 5 | 7,533333 | 10,125 | 14,56667 | 20,26151 | 26,3199 | 45,92626 |
| malignant | 15,28333 | 32,25365 | 78,15822 | 164,5484 | 354,214 | 1267,698 | 14563,78 |

**Oneway Anova**

**Summary of Fit**

| Rsquare | 0,050423 |
| --- | --- |
| Adj Rsquare | 0,043829 |
| Root Mean Square Error | 1261,315 |
| Mean of Response | 304,7075 |
| Observations (or Sum Wgts) | 146 |

**Pooled t Test**

malignant-benign

Assuming equal variances

| Difference | 577,311 | t Ratio | 2,765237 |
| --- | --- | --- | --- |
| Std Err Dif | 208,774 | DF | 144 |
| Upper CL Dif | 989,969 | Prob > |t| | 0,0064* |
| Lower CL Dif | 164,653 | Prob > t | 0,0032* |
| Confidence | 0,95 | Prob < t | 0,9968 |

**Analysis of Variance**

| **Source** | **DF** | **Sum of Squares** | **Mean Square** | **F Ratio** | **Prob > F** |
| --- | --- | --- | --- | --- | --- |
| Type | 1 | 12165004 | 12165004 | 7,6465 | 0,0064* |
| Error | 144 | 229091974 | 1590916,5 |  |  |
| C. Total | 145 | 241256977 |  |  |  |

**Means for Oneway Anova**

| **Level** | **Number** | **Mean** | **Std Error** | **Lower 95%** | **Upper 95%** |
| --- | --- | --- | --- | --- | --- |
| benign | 73 | 16,052 | 147,63 | -275,7 | 307,85 |
| malignant | 73 | 593,363 | 147,63 | 301,6 | 885,16 |

Std Error uses a pooled estimate of error variance

**Oneway Analysis of original-glrlm-ShortRunEmphasis By Type**

**Quantiles**

| **Level** | **Minimum** | **10%** | **25%** | **Median** | **75%** | **90%** | **Maximum** |
| --- | --- | --- | --- | --- | --- | --- | --- |
| benign | 0,892553 | 0,937076 | 0,961218 | 0,974802 | 0,985243 | 1 | 1 |
| malignant | 0,827472 | 0,965425 | 0,967972 | 0,97305 | 0,979992 | 0,983907 | 0,988903 |

**Oneway Anova**

**Summary of Fit**

| Rsquare | 0,000741 |
| --- | --- |
| Adj Rsquare | -0,0062 |
| Root Mean Square Error | 0,021598 |
| Mean of Response | 0,971341 |
| Observations (or Sum Wgts) | 146 |

**Pooled t Test**

malignant-benign

Assuming equal variances

| Difference | 0,00117 | t Ratio | 0,326839 |
| --- | --- | --- | --- |
| Std Err Dif | 0,00357 | DF | 144 |
| Upper CL Dif | 0,00823 | Prob > |t| | 0,7443 |
| Lower CL Dif | -0,00590 | Prob > t | 0,3721 |
| Confidence | 0,95 | Prob < t | 0,6279 |

**Analysis of Variance**

| **Source** | **DF** | **Sum of Squares** | **Mean Square** | **F Ratio** | **Prob > F** |
| --- | --- | --- | --- | --- | --- |
| Type | 1 | 0,00004983 | 0,000050 | 0,1068 | 0,7443 |
| Error | 144 | 0,06717495 | 0,000466 |  |  |
| C. Total | 145 | 0,06722478 |  |  |  |

**Means for Oneway Anova**

| **Level** | **Number** | **Mean** | **Std Error** | **Lower 95%** | **Upper 95%** |
| --- | --- | --- | --- | --- | --- |
| benign | 73 | 0,970757 | 0,00253 | 0,96576 | 0,97575 |
| malignant | 73 | 0,971926 | 0,00253 | 0,96693 | 0,97692 |

Std Error uses a pooled estimate of error variance

**Oneway Analysis of original-glrlm-LongRunHighGrayLevelEmphasis By Type**

**Quantiles**

| **Level** | **Minimum** | **10%** | **25%** | **Median** | **75%** | **90%** | **Maximum** |
| --- | --- | --- | --- | --- | --- | --- | --- |
| benign | 23,83333 | 63,1125 | 105,4406 | 203,3159 | 328,3742 | 478,2749 | 3501,459 |
| malignant | 8,631241 | 196,4564 | 285,8686 | 452,8838 | 772,3725 | 1146,009 | 2335,755 |

**Oneway Anova**

**Summary of Fit**

| Rsquare | 0,119981 |
| --- | --- |
| Adj Rsquare | 0,11387 |
| Root Mean Square Error | 403,9452 |
| Mean of Response | 423,9991 |
| Observations (or Sum Wgts) | 146 |

**Pooled t Test**

malignant-benign

Assuming equal variances

| Difference | 296,256 | t Ratio | 4,430892 |
| --- | --- | --- | --- |
| Std Err Dif | 66,861 | DF | 144 |
| Upper CL Dif | 428,413 | Prob > |t| | <,0001* |
| Lower CL Dif | 164,099 | Prob > t | <,0001* |
| Confidence | 0,95 | Prob < t | 1,0000 |

**Analysis of Variance**

| **Source** | **DF** | **Sum of Squares** | **Mean Square** | **F Ratio** | **Prob > F** |
| --- | --- | --- | --- | --- | --- |
| Type | 1 | 3203517 | 3203517 | 19,6328 | <,0001* |
| Error | 144 | 23496724 | 163172 |  |  |
| C. Total | 145 | 26700242 |  |  |  |

**Means for Oneway Anova**

| **Level** | **Number** | **Mean** | **Std Error** | **Lower 95%** | **Upper 95%** |
| --- | --- | --- | --- | --- | --- |
| benign | 73 | 275,871 | 47,278 | 182,42 | 369,32 |
| malignant | 73 | 572,127 | 47,278 | 478,68 | 665,58 |

Std Error uses a pooled estimate of error variance

**Oneway Analysis of original-glrlm-RunPercentage By Type**

**Quantiles**

| **Level** | **Minimum** | **10%** | **25%** | **Median** | **75%** | **90%** | **Maximum** |
| --- | --- | --- | --- | --- | --- | --- | --- |
| benign | 0,868421 | 0,926573 | 0,950806 | 0,96875 | 0,981456 | 1 | 1 |
| malignant | 0,810147 | 0,954305 | 0,957606 | 0,963801 | 0,97297 | 0,978408 | 0,985871 |

**Oneway Anova**

**Summary of Fit**

| Rsquare | 0,000143 |
| --- | --- |
| Adj Rsquare | -0,0068 |
| Root Mean Square Error | 0,024828 |
| Mean of Response | 0,963952 |
| Observations (or Sum Wgts) | 146 |

**Pooled t Test**

malignant-benign

Assuming equal variances

| Difference | -0,00059 | t Ratio | -0,14352 |
| --- | --- | --- | --- |
| Std Err Dif | 0,00411 | DF | 144 |
| Upper CL Dif | 0,00753 | Prob > |t| | 0,8861 |
| Lower CL Dif | -0,00871 | Prob > t | 0,5570 |
| Confidence | 0,95 | Prob < t | 0,4430 |

**Analysis of Variance**

| **Source** | **DF** | **Sum of Squares** | **Mean Square** | **F Ratio** | **Prob > F** |
| --- | --- | --- | --- | --- | --- |
| Type | 1 | 0,00001270 | 0,000013 | 0,0206 | 0,8861 |
| Error | 144 | 0,08876316 | 0,000616 |  |  |
| C. Total | 145 | 0,08877586 |  |  |  |

**Means for Oneway Anova**

| **Level** | **Number** | **Mean** | **Std Error** | **Lower 95%** | **Upper 95%** |
| --- | --- | --- | --- | --- | --- |
| benign | 73 | 0,964247 | 0,00291 | 0,95850 | 0,96999 |
| malignant | 73 | 0,963657 | 0,00291 | 0,95791 | 0,96940 |

Std Error uses a pooled estimate of error variance

**Oneway Analysis of original-glrlm-LongRunLowGrayLevelEmphasis By Type**

**Quantiles**

| **Level** | **Minimum** | **10%** | **25%** | **Median** | **75%** | **90%** | **Maximum** |
| --- | --- | --- | --- | --- | --- | --- | --- |
| benign | 0,028572 | 0,050371 | 0,063282 | 0,094712 | 0,12754 | 0,180206 | 0,562083 |
| malignant | 0,001105 | 0,003118 | 0,008099 | 0,015727 | 0,03357 | 0,05235 | 0,856265 |

**Oneway Anova**

**Summary of Fit**

| Rsquare | 0,148736 |
| --- | --- |
| Adj Rsquare | 0,142825 |
| Root Mean Square Error | 0,08854 |
| Mean of Response | 0,07189 |
| Observations (or Sum Wgts) | 146 |

**Pooled t Test**

malignant-benign

Assuming equal variances

| Difference | -0,07351 | t Ratio | -5,016 |
| --- | --- | --- | --- |
| Std Err Dif | 0,01466 | DF | 144 |
| Upper CL Dif | -0,04454 | Prob > |t| | <,0001* |
| Lower CL Dif | -0,10248 | Prob > t | 1,0000 |
| Confidence | 0,95 | Prob < t | <,0001* |

**Analysis of Variance**

| **Source** | **DF** | **Sum of Squares** | **Mean Square** | **F Ratio** | **Prob > F** |
| --- | --- | --- | --- | --- | --- |
| Type | 1 | 0,1972396 | 0,197240 | 25,1603 | <,0001* |
| Error | 144 | 1,1288622 | 0,007839 |  |  |
| C. Total | 145 | 1,3261017 |  |  |  |

**Means for Oneway Anova**

| **Level** | **Number** | **Mean** | **Std Error** | **Lower 95%** | **Upper 95%** |
| --- | --- | --- | --- | --- | --- |
| benign | 73 | 0,108645 | 0,01036 | 0,08816 | 0,12913 |
| malignant | 73 | 0,035135 | 0,01036 | 0,01465 | 0,05562 |

Std Error uses a pooled estimate of error variance

**Oneway Analysis of original-glrlm-RunEntropy By Type**

**Quantiles**

| **Level** | **Minimum** | **10%** | **25%** | **Median** | **75%** | **90%** | **Maximum** |
| --- | --- | --- | --- | --- | --- | --- | --- |
| benign | 2,251629 | 2,55847 | 3,000905 | 3,459432 | 3,738423 | 4,055803 | 4,310358 |
| malignant | 2,390519 | 4,00466 | 4,46811 | 4,748365 | 5,09795 | 5,386266 | 5,843222 |

**Oneway Anova**

**Summary of Fit**

| Rsquare | 0,59882 |
| --- | --- |
| Adj Rsquare | 0,596034 |
| Root Mean Square Error | 0,546083 |
| Mean of Response | 4,043851 |
| Observations (or Sum Wgts) | 146 |

**Pooled t Test**

malignant-benign

Assuming equal variances

| Difference | 1,32517 | t Ratio | 14,66086 |
| --- | --- | --- | --- |
| Std Err Dif | 0,09039 | DF | 144 |
| Upper CL Dif | 1,50383 | Prob > |t| | <,0001* |
| Lower CL Dif | 1,14651 | Prob > t | <,0001* |
| Confidence | 0,95 | Prob < t | 1,0000 |

**Analysis of Variance**

| **Source** | **DF** | **Sum of Squares** | **Mean Square** | **F Ratio** | **Prob > F** |
| --- | --- | --- | --- | --- | --- |
| Type | 1 | 64,09689 | 64,0969 | 214,9408 | <,0001* |
| Error | 144 | 42,94183 | 0,2982 |  |  |
| C. Total | 145 | 107,03872 |  |  |  |

**Means for Oneway Anova**

| **Level** | **Number** | **Mean** | **Std Error** | **Lower 95%** | **Upper 95%** |
| --- | --- | --- | --- | --- | --- |
| benign | 73 | 3,38126 | 0,06391 | 3,2549 | 3,5076 |
| malignant | 73 | 4,70644 | 0,06391 | 4,5801 | 4,8328 |

Std Error uses a pooled estimate of error variance

**Oneway Analysis of original-glrlm-HighGrayLevelRunEmphasis By Type**

**Quantiles**

| **Level** | **Minimum** | **10%** | **25%** | **Median** | **75%** | **90%** | **Maximum** |
| --- | --- | --- | --- | --- | --- | --- | --- |
| benign | 22,25 | 51,58813 | 99,47271 | 187,1818 | 291,3658 | 451,6281 | 3222,384 |
| malignant | 4,424969 | 179,7434 | 258,4643 | 415,1315 | 702,0604 | 1014,033 | 2077,596 |

**Oneway Anova**

**Summary of Fit**

| Rsquare | 0,118381 |
| --- | --- |
| Adj Rsquare | 0,112259 |
| Root Mean Square Error | 367,1475 |
| Mean of Response | 383,745 |
| Observations (or Sum Wgts) | 146 |

**Pooled t Test**

malignant-benign

Assuming equal variances

| Difference | 267,224 | t Ratio | 4,397255 |
| --- | --- | --- | --- |
| Std Err Dif | 60,771 | DF | 144 |
| Upper CL Dif | 387,342 | Prob > |t| | <,0001* |
| Lower CL Dif | 147,106 | Prob > t | <,0001* |
| Confidence | 0,95 | Prob < t | 1,0000 |

**Analysis of Variance**

| **Source** | **DF** | **Sum of Squares** | **Mean Square** | **F Ratio** | **Prob > F** |
| --- | --- | --- | --- | --- | --- |
| Type | 1 | 2606420 | 2606420 | 19,3359 | <,0001* |
| Error | 144 | 19410806 | 134797 |  |  |
| C. Total | 145 | 22017227 |  |  |  |

**Means for Oneway Anova**

| **Level** | **Number** | **Mean** | **Std Error** | **Lower 95%** | **Upper 95%** |
| --- | --- | --- | --- | --- | --- |
| benign | 73 | 250,133 | 42,971 | 165,20 | 335,07 |
| malignant | 73 | 517,357 | 42,971 | 432,42 | 602,29 |

Std Error uses a pooled estimate of error variance

**Oneway Analysis of original-glrlm-RunLengthNonUniformityNormalized By Type**

**Quantiles**

| **Level** | **Minimum** | **10%** | **25%** | **Median** | **75%** | **90%** | **Maximum** |
| --- | --- | --- | --- | --- | --- | --- | --- |
| benign | 0,761246 | 0,856052 | 0,908403 | 0,937884 | 0,96307 | 1 | 1 |
| malignant | 0,678678 | 0,912995 | 0,919019 | 0,932171 | 0,948544 | 0,959557 | 0,971959 |

**Oneway Anova**

**Summary of Fit**

| Rsquare | 0,000323 |
| --- | --- |
| Adj Rsquare | -0,00662 |
| Root Mean Square Error | 0,044937 |
| Mean of Response | 0,931424 |
| Observations (or Sum Wgts) | 146 |

**Pooled t Test**

malignant-benign

Assuming equal variances

| Difference | -0,00160 | t Ratio | -0,21559 |
| --- | --- | --- | --- |
| Std Err Dif | 0,00744 | DF | 144 |
| Upper CL Dif | 0,01310 | Prob > |t| | 0,8296 |
| Lower CL Dif | -0,01631 | Prob > t | 0,5852 |
| Confidence | 0,95 | Prob < t | 0,4148 |

**Analysis of Variance**

| **Source** | **DF** | **Sum of Squares** | **Mean Square** | **F Ratio** | **Prob > F** |
| --- | --- | --- | --- | --- | --- |
| Type | 1 | 0,00009386 | 0,000094 | 0,0465 | 0,8296 |
| Error | 144 | 0,29078923 | 0,002019 |  |  |
| C. Total | 145 | 0,29088308 |  |  |  |

**Means for Oneway Anova**

| **Level** | **Number** | **Mean** | **Std Error** | **Lower 95%** | **Upper 95%** |
| --- | --- | --- | --- | --- | --- |
| benign | 73 | 0,932226 | 0,00526 | 0,92183 | 0,94262 |
| malignant | 73 | 0,930623 | 0,00526 | 0,92023 | 0,94102 |

Std Error uses a pooled estimate of error variance

**Oneway Analysis of original-glszm-GrayLevelVariance By Type**

**Quantiles**

| **Level** | **Minimum** | **10%** | **25%** | **Median** | **75%** | **90%** | **Maximum** |
| --- | --- | --- | --- | --- | --- | --- | --- |
| benign | 5,234375 | 12,60408 | 19,33058 | 34,64 | 52,0727 | 77,68551 | 408,61 |
| malignant | 1,25 | 29,62343 | 42,53969 | 59,17562 | 87,67461 | 124,8344 | 193,0242 |

**Oneway Anova**

**Summary of Fit**

| Rsquare | 0,078384 |
| --- | --- |
| Adj Rsquare | 0,071984 |
| Root Mean Square Error | 44,14115 |
| Mean of Response | 56,31 |
| Observations (or Sum Wgts) | 146 |

**Pooled t Test**

malignant-benign

Assuming equal variances

| Difference | 25,5692 | t Ratio | 3,49961 |
| --- | --- | --- | --- |
| Std Err Dif | 7,3063 | DF | 144 |
| Upper CL Dif | 40,0106 | Prob > |t| | 0,0006* |
| Lower CL Dif | 11,1277 | Prob > t | 0,0003* |
| Confidence | 0,95 | Prob < t | 0,9997 |

**Analysis of Variance**

| **Source** | **DF** | **Sum of Squares** | **Mean Square** | **F Ratio** | **Prob > F** |
| --- | --- | --- | --- | --- | --- |
| Type | 1 | 23863,08 | 23863,1 | 12,2473 | 0,0006* |
| Error | 144 | 280575,52 | 1948,4 |  |  |
| C. Total | 145 | 304438,60 |  |  |  |

**Means for Oneway Anova**

| **Level** | **Number** | **Mean** | **Std Error** | **Lower 95%** | **Upper 95%** |
| --- | --- | --- | --- | --- | --- |
| benign | 73 | 43,5254 | 5,1663 | 33,314 | 53,737 |
| malignant | 73 | 69,0946 | 5,1663 | 58,883 | 79,306 |

Std Error uses a pooled estimate of error variance

**Oneway Analysis of original-glszm-ZoneVariance By Type**

**Quantiles**

| **Level** | **Minimum** | **10%** | **25%** | **Median** | **75%** | **90%** | **Maximum** |
| --- | --- | --- | --- | --- | --- | --- | --- |
| benign | 0 | 0 | 0,073697 | 0,130178 | 0,290801 | 0,59 | 1,909722 |
| malignant | 0,177515 | 0,482602 | 0,804509 | 1,495169 | 2,696691 | 3,523793 | 48,6875 |

**Oneway Anova**

**Summary of Fit**

| Rsquare | 0,075715 |
| --- | --- |
| Adj Rsquare | 0,069297 |
| Root Mean Square Error | 4,006787 |
| Mean of Response | 1,363399 |
| Observations (or Sum Wgts) | 146 |

**Pooled t Test**

malignant-benign

Assuming equal variances

| Difference | 2,27783 | t Ratio | 3,434557 |
| --- | --- | --- | --- |
| Std Err Dif | 0,66321 | DF | 144 |
| Upper CL Dif | 3,58871 | Prob > |t| | 0,0008* |
| Lower CL Dif | 0,96695 | Prob > t | 0,0004* |
| Confidence | 0,95 | Prob < t | 0,9996 |

**Analysis of Variance**

| **Source** | **DF** | **Sum of Squares** | **Mean Square** | **F Ratio** | **Prob > F** |
| --- | --- | --- | --- | --- | --- |
| Type | 1 | 189,3800 | 189,380 | 11,7962 | 0,0008* |
| Error | 144 | 2311,8255 | 16,054 |  |  |
| C. Total | 145 | 2501,2055 |  |  |  |

**Means for Oneway Anova**

| **Level** | **Number** | **Mean** | **Std Error** | **Lower 95%** | **Upper 95%** |
| --- | --- | --- | --- | --- | --- |
| benign | 73 | 0,22449 | 0,46896 | -0,702 | 1,1514 |
| malignant | 73 | 2,50231 | 0,46896 | 1,575 | 3,4292 |

Std Error uses a pooled estimate of error variance

**Oneway Analysis of original-glszm-GrayLevelNonUniformityNormalized By Type**

**Quantiles**

| **Level** | **Minimum** | **10%** | **25%** | **Median** | **75%** | **90%** | **Maximum** |
| --- | --- | --- | --- | --- | --- | --- | --- |
| benign | 0,056213 | 0,065664 | 0,08 | 0,09375 | 0,135802 | 0,170799 | 0,22449 |
| malignant | 0,022073 | 0,030716 | 0,03494 | 0,044126 | 0,052334 | 0,069977 | 0,25 |

**Oneway Anova**

**Summary of Fit**

| Rsquare | 0,424274 |
| --- | --- |
| Adj Rsquare | 0,420276 |
| Root Mean Square Error | 0,035234 |
| Mean of Response | 0,0787 |
| Observations (or Sum Wgts) | 146 |

**Pooled t Test**

malignant-benign

Assuming equal variances

| Difference | -0,06008 | t Ratio | -10,3014 |
| --- | --- | --- | --- |
| Std Err Dif | 0,00583 | DF | 144 |
| Upper CL Dif | -0,04855 | Prob > |t| | <,0001* |
| Lower CL Dif | -0,07161 | Prob > t | 1,0000 |
| Confidence | 0,95 | Prob < t | <,0001* |

**Analysis of Variance**

| **Source** | **DF** | **Sum of Squares** | **Mean Square** | **F Ratio** | **Prob > F** |
| --- | --- | --- | --- | --- | --- |
| Type | 1 | 0,13174231 | 0,131742 | 106,1188 | <,0001* |
| Error | 144 | 0,17877030 | 0,001241 |  |  |
| C. Total | 145 | 0,31051261 |  |  |  |

**Means for Oneway Anova**

| **Level** | **Number** | **Mean** | **Std Error** | **Lower 95%** | **Upper 95%** |
| --- | --- | --- | --- | --- | --- |
| benign | 73 | 0,108739 | 0,00412 | 0,10059 | 0,11689 |
| malignant | 73 | 0,048661 | 0,00412 | 0,04051 | 0,05681 |

Std Error uses a pooled estimate of error variance

**Oneway Analysis of original-glszm-SizeZoneNonUniformityNormalized By Type**

**Quantiles**

| **Level** | **Minimum** | **10%** | **25%** | **Median** | **75%** | **90%** | **Maximum** |
| --- | --- | --- | --- | --- | --- | --- | --- |
| benign | 0,450617 | 0,555556 | 0,686648 | 0,768889 | 0,862668 | 1 | 1 |
| malignant | 0,25 | 0,423078 | 0,461443 | 0,500918 | 0,569504 | 0,660817 | 0,693762 |

**Oneway Anova**

**Summary of Fit**

| Rsquare | 0,539033 |
| --- | --- |
| Adj Rsquare | 0,535832 |
| Root Mean Square Error | 0,120764 |
| Mean of Response | 0,648402 |
| Observations (or Sum Wgts) | 146 |

**Pooled t Test**

malignant-benign

Assuming equal variances

| Difference | -0,25939 | t Ratio | -12,9764 |
| --- | --- | --- | --- |
| Std Err Dif | 0,01999 | DF | 144 |
| Upper CL Dif | -0,21988 | Prob > |t| | <,0001* |
| Lower CL Dif | -0,29890 | Prob > t | 1,0000 |
| Confidence | 0,95 | Prob < t | <,0001* |

**Analysis of Variance**

| **Source** | **DF** | **Sum of Squares** | **Mean Square** | **F Ratio** | **Prob > F** |
| --- | --- | --- | --- | --- | --- |
| Type | 1 | 2,4557492 | 2,45575 | 168,3868 | <,0001* |
| Error | 144 | 2,1000928 | 0,01458 |  |  |
| C. Total | 145 | 4,5558420 |  |  |  |

**Means for Oneway Anova**

| **Level** | **Number** | **Mean** | **Std Error** | **Lower 95%** | **Upper 95%** |
| --- | --- | --- | --- | --- | --- |
| benign | 73 | 0,778095 | 0,01413 | 0,75016 | 0,80603 |
| malignant | 73 | 0,518710 | 0,01413 | 0,49077 | 0,54665 |

Std Error uses a pooled estimate of error variance

**Oneway Analysis of original-glszm-SizeZoneNonUniformity By Type**

**Quantiles**

| **Level** | **Minimum** | **10%** | **25%** | **Median** | **75%** | **90%** | **Maximum** |
| --- | --- | --- | --- | --- | --- | --- | --- |
| benign | 3,333333 | 5 | 7,863636 | 10,875 | 15,01053 | 20,05455 | 32,44186 |
| malignant | 1 | 17,25963 | 28,97279 | 51 | 117,9687 | 332,9909 | 4397,37 |

**Oneway Anova**

**Summary of Fit**

| Rsquare | 0,048705 |
| --- | --- |
| Adj Rsquare | 0,042099 |
| Root Mean Square Error | 377,2801 |
| Mean of Response | 96,50794 |
| Observations (or Sum Wgts) | 146 |

**Pooled t Test**

malignant-benign

Assuming equal variances

| Difference | 169,562 | t Ratio | 2,71526 |
| --- | --- | --- | --- |
| Std Err Dif | 62,448 | DF | 144 |
| Upper CL Dif | 292,995 | Prob > |t| | 0,0074* |
| Lower CL Dif | 46,129 | Prob > t | 0,0037* |
| Confidence | 0,95 | Prob < t | 0,9963 |

**Analysis of Variance**

| **Source** | **DF** | **Sum of Squares** | **Mean Square** | **F Ratio** | **Prob > F** |
| --- | --- | --- | --- | --- | --- |
| Type | 1 | 1049423 | 1049423 | 7,3726 | 0,0074* |
| Error | 144 | 20497005 | 142340 |  |  |
| C. Total | 145 | 21546428 |  |  |  |

**Means for Oneway Anova**

| **Level** | **Number** | **Mean** | **Std Error** | **Lower 95%** | **Upper 95%** |
| --- | --- | --- | --- | --- | --- |
| benign | 73 | 11,727 | 44,157 | -75,55 | 99,01 |
| malignant | 73 | 181,289 | 44,157 | 94,01 | 268,57 |

Std Error uses a pooled estimate of error variance

**Oneway Analysis of original-glszm-GrayLevelNonUniformity By Type**

**Quantiles**

| **Level** | **Minimum** | **10%** | **25%** | **Median** | **75%** | **90%** | **Maximum** |
| --- | --- | --- | --- | --- | --- | --- | --- |
| benign | 1 | 1 | 1,183333 | 1,4 | 1,571429 | 1,845455 | 3,2 |
| malignant | 1 | 1,679503 | 2,855666 | 4,311111 | 9,131208 | 26,69414 | 199,0071 |

**Oneway Anova**

**Summary of Fit**

| Rsquare | 0,076742 |
| --- | --- |
| Adj Rsquare | 0,07033 |
| Root Mean Square Error | 18,0832 |
| Mean of Response | 6,614893 |
| Observations (or Sum Wgts) | 146 |

**Pooled t Test**

malignant-benign

Assuming equal variances

| Difference | 10,3553 | t Ratio | 3,459678 |
| --- | --- | --- | --- |
| Std Err Dif | 2,9932 | DF | 144 |
| Upper CL Dif | 16,2715 | Prob > |t| | 0,0007* |
| Lower CL Dif | 4,4392 | Prob > t | 0,0004* |
| Confidence | 0,95 | Prob < t | 0,9996 |

**Analysis of Variance**

| **Source** | **DF** | **Sum of Squares** | **Mean Square** | **F Ratio** | **Prob > F** |
| --- | --- | --- | --- | --- | --- |
| Type | 1 | 3914,010 | 3914,01 | 11,9694 | 0,0007* |
| Error | 144 | 47088,295 | 327,00 |  |  |
| C. Total | 145 | 51002,305 |  |  |  |

**Means for Oneway Anova**

| **Level** | **Number** | **Mean** | **Std Error** | **Lower 95%** | **Upper 95%** |
| --- | --- | --- | --- | --- | --- |
| benign | 73 | 1,4372 | 2,1165 | -2,746 | 5,621 |
| malignant | 73 | 11,7926 | 2,1165 | 7,609 | 15,976 |

Std Error uses a pooled estimate of error variance

**Oneway Analysis of original-glszm-LargeAreaEmphasis By Type**

**Quantiles**

| **Level** | **Minimum** | **10%** | **25%** | **Median** | **75%** | **90%** | **Maximum** |
| --- | --- | --- | --- | --- | --- | --- | --- |
| benign | 1 | 1 | 1,240385 | 1,461538 | 1,8775 | 2,4 | 4,416667 |
| malignant | 1,692308 | 2,356122 | 2,926314 | 4,232932 | 6,016519 | 7,005635 | 186,75 |

**Oneway Anova**

**Summary of Fit**

| Rsquare | 0,031176 |
| --- | --- |
| Adj Rsquare | 0,024448 |
| Root Mean Square Error | 15,15541 |
| Mean of Response | 4,324196 |
| Observations (or Sum Wgts) | 146 |

**Pooled t Test**

malignant-benign

Assuming equal variances

| Difference | 5,4000 | t Ratio | 2,152631 |
| --- | --- | --- | --- |
| Std Err Dif | 2,5085 | DF | 144 |
| Upper CL Dif | 10,3583 | Prob > |t| | 0,0330* |
| Lower CL Dif | 0,4416 | Prob > t | 0,0165* |
| Confidence | 0,95 | Prob < t | 0,9835 |

**Analysis of Variance**

| **Source** | **DF** | **Sum of Squares** | **Mean Square** | **F Ratio** | **Prob > F** |
| --- | --- | --- | --- | --- | --- |
| Type | 1 | 1064,326 | 1064,33 | 4,6338 | 0,0330* |
| Error | 144 | 33074,846 | 229,69 |  |  |
| C. Total | 145 | 34139,172 |  |  |  |

**Means for Oneway Anova**

| **Level** | **Number** | **Mean** | **Std Error** | **Lower 95%** | **Upper 95%** |
| --- | --- | --- | --- | --- | --- |
| benign | 73 | 1,62421 | 1,7738 | -1,882 | 5,130 |
| malignant | 73 | 7,02418 | 1,7738 | 3,518 | 10,530 |

Std Error uses a pooled estimate of error variance

**Oneway Analysis of original-glszm-SmallAreaHighGrayLevelEmphasis By Type**

**Quantiles**

| **Level** | **Minimum** | **10%** | **25%** | **Median** | **75%** | **90%** | **Maximum** |
| --- | --- | --- | --- | --- | --- | --- | --- |
| benign | 23,70625 | 49,03462 | 87,12865 | 162,0769 | 278,0322 | 382,4834 | 2906,825 |
| malignant | 4,022206 | 131,1341 | 198,2362 | 310,1828 | 576,2683 | 757,6555 | 1766,947 |

**Oneway Anova**

**Summary of Fit**

| Rsquare | 0,075195 |
| --- | --- |
| Adj Rsquare | 0,068772 |
| Root Mean Square Error | 317,5715 |
| Mean of Response | 314,0471 |
| Observations (or Sum Wgts) | 146 |

**Pooled t Test**

malignant-benign

Assuming equal variances

| Difference | 179,864 | t Ratio | 3,421758 |
| --- | --- | --- | --- |
| Std Err Dif | 52,565 | DF | 144 |
| Upper CL Dif | 283,762 | Prob > |t| | 0,0008* |
| Lower CL Dif | 75,966 | Prob > t | 0,0004* |
| Confidence | 0,95 | Prob < t | 0,9996 |

**Analysis of Variance**

| **Source** | **DF** | **Sum of Squares** | **Mean Square** | **F Ratio** | **Prob > F** |
| --- | --- | --- | --- | --- | --- |
| Type | 1 | 1180815 | 1180815 | 11,7084 | 0,0008* |
| Error | 144 | 14522642 | 100852 |  |  |
| C. Total | 145 | 15703457 |  |  |  |

**Means for Oneway Anova**

| **Level** | **Number** | **Mean** | **Std Error** | **Lower 95%** | **Upper 95%** |
| --- | --- | --- | --- | --- | --- |
| benign | 73 | 224,115 | 37,169 | 150,65 | 297,58 |
| malignant | 73 | 403,979 | 37,169 | 330,51 | 477,45 |

Std Error uses a pooled estimate of error variance

**Oneway Analysis of original-glszm-ZonePercentage By Type**

**Quantiles**

| **Level** | **Minimum** | **10%** | **25%** | **Median** | **75%** | **90%** | **Maximum** |
| --- | --- | --- | --- | --- | --- | --- | --- |
| benign | 0,615385 | 0,736364 | 0,807988 | 0,866667 | 0,925824 | 1 | 1 |
| malignant | 0,085106 | 0,523973 | 0,557762 | 0,617647 | 0,701225 | 0,74847 | 0,816327 |

**Oneway Anova**

**Summary of Fit**

| Rsquare | 0,58573 |
| --- | --- |
| Adj Rsquare | 0,582853 |
| Root Mean Square Error | 0,10268 |
| Mean of Response | 0,742613 |
| Observations (or Sum Wgts) | 146 |

**Pooled t Test**

malignant-benign

Assuming equal variances

| Difference | -0,24251 | t Ratio | -14,2688 |
| --- | --- | --- | --- |
| Std Err Dif | 0,01700 | DF | 144 |
| Upper CL Dif | -0,20891 | Prob > |t| | <,0001* |
| Lower CL Dif | -0,27610 | Prob > t | 1,0000 |
| Confidence | 0,95 | Prob < t | <,0001* |

**Analysis of Variance**

| **Source** | **DF** | **Sum of Squares** | **Mean Square** | **F Ratio** | **Prob > F** |
| --- | --- | --- | --- | --- | --- |
| Type | 1 | 2,1465703 | 2,14657 | 203,5995 | <,0001* |
| Error | 144 | 1,5182065 | 0,01054 |  |  |
| C. Total | 145 | 3,6647768 |  |  |  |

**Means for Oneway Anova**

| **Level** | **Number** | **Mean** | **Std Error** | **Lower 95%** | **Upper 95%** |
| --- | --- | --- | --- | --- | --- |
| benign | 73 | 0,863867 | 0,01202 | 0,84011 | 0,88762 |
| malignant | 73 | 0,621359 | 0,01202 | 0,59761 | 0,64511 |

Std Error uses a pooled estimate of error variance

**Oneway Analysis of original-glszm-LargeAreaLowGrayLevelEmphasis By Type**

**Quantiles**

| **Level** | **Minimum** | **10%** | **25%** | **Median** | **75%** | **90%** | **Maximum** |
| --- | --- | --- | --- | --- | --- | --- | --- |
| benign | 0,033069 | 0,056538 | 0,074704 | 0,10776 | 0,173719 | 0,237577 | 1,378929 |
| malignant | 0,00856 | 0,010946 | 0,019252 | 0,03519 | 0,077391 | 0,128378 | 84,62674 |

**Oneway Anova**

**Summary of Fit**

| Rsquare | 0,006143 |
| --- | --- |
| Adj Rsquare | -0,00076 |
| Root Mean Square Error | 7,000128 |
| Mean of Response | 0,692532 |
| Observations (or Sum Wgts) | 146 |

**Pooled t Test**

malignant-benign

Assuming equal variances

| Difference | 1,0931 | t Ratio | 0,943394 |
| --- | --- | --- | --- |
| Std Err Dif | 1,1587 | DF | 144 |
| Upper CL Dif | 3,3833 | Prob > |t| | 0,3471 |
| Lower CL Dif | -1,1971 | Prob > t | 0,1735 |
| Confidence | 0,95 | Prob < t | 0,8265 |

**Analysis of Variance**

| **Source** | **DF** | **Sum of Squares** | **Mean Square** | **F Ratio** | **Prob > F** |
| --- | --- | --- | --- | --- | --- |
| Type | 1 | 43,6112 | 43,6112 | 0,8900 | 0,3471 |
| Error | 144 | 7056,2580 | 49,0018 |  |  |
| C. Total | 145 | 7099,8692 |  |  |  |

**Means for Oneway Anova**

| **Level** | **Number** | **Mean** | **Std Error** | **Lower 95%** | **Upper 95%** |
| --- | --- | --- | --- | --- | --- |
| benign | 73 | 0,14599 | 0,81930 | -1,473 | 1,7654 |
| malignant | 73 | 1,23907 | 0,81930 | -0,380 | 2,8585 |

Std Error uses a pooled estimate of error variance

**Oneway Analysis of original-glszm-LargeAreaHighGrayLevelEmphasis By Type**

**Quantiles**

| **Level** | **Minimum** | **10%** | **25%** | **Median** | **75%** | **90%** | **Maximum** |
| --- | --- | --- | --- | --- | --- | --- | --- |
| benign | 34,71429 | 81,68611 | 141,0196 | 264,8571 | 473,9038 | 720,9524 | 4302,2 |
| malignant | 252,8421 | 622,8347 | 1007,59 | 1558,138 | 2795,615 | 5489,759 | 15257,9 |

**Oneway Anova**

**Summary of Fit**

| Rsquare | 0,256687 |
| --- | --- |
| Adj Rsquare | 0,251525 |
| Root Mean Square Error | 1679,077 |
| Mean of Response | 1351,256 |
| Observations (or Sum Wgts) | 146 |

**Pooled t Test**

malignant-benign

Assuming equal variances

| Difference | 1959,85 | t Ratio | 7,051762 |
| --- | --- | --- | --- |
| Std Err Dif | 277,92 | DF | 144 |
| Upper CL Dif | 2509,18 | Prob > |t| | <,0001* |
| Lower CL Dif | 1410,51 | Prob > t | <,0001* |
| Confidence | 0,95 | Prob < t | 1,0000 |

**Analysis of Variance**

| **Source** | **DF** | **Sum of Squares** | **Mean Square** | **F Ratio** | **Prob > F** |
| --- | --- | --- | --- | --- | --- |
| Type | 1 | 140196350 | 140196350 | 49,7273 | <,0001* |
| Error | 144 | 405979325 | 2819300,9 |  |  |
| C. Total | 145 | 546175675 |  |  |  |

**Means for Oneway Anova**

| **Level** | **Number** | **Mean** | **Std Error** | **Lower 95%** | **Upper 95%** |
| --- | --- | --- | --- | --- | --- |
| benign | 73 | 371,33 | 196,52 | -17 | 759,8 |
| malignant | 73 | 2331,18 | 196,52 | 1943 | 2719,6 |

Std Error uses a pooled estimate of error variance

**Oneway Analysis of original-glszm-HighGrayLevelZoneEmphasis By Type**

**Quantiles**

| **Level** | **Minimum** | **10%** | **25%** | **Median** | **75%** | **90%** | **Maximum** |
| --- | --- | --- | --- | --- | --- | --- | --- |
| benign | 26,71429 | 52,67179 | 104,9364 | 183,5714 | 296,1571 | 425,0154 | 3185,9 |
| malignant | 7,5 | 178,4603 | 275,0079 | 413,5098 | 725,8434 | 1022,976 | 2311,652 |

**Oneway Anova**

**Summary of Fit**

| Rsquare | 0,126968 |
| --- | --- |
| Adj Rsquare | 0,120905 |
| Root Mean Square Error | 375,48 |
| Mean of Response | 390,5379 |
| Observations (or Sum Wgts) | 146 |

**Pooled t Test**

malignant-benign

Assuming equal variances

| Difference | 284,416 | t Ratio | 4,576285 |
| --- | --- | --- | --- |
| Std Err Dif | 62,150 | DF | 144 |
| Upper CL Dif | 407,260 | Prob > |t| | <,0001* |
| Lower CL Dif | 161,572 | Prob > t | <,0001* |
| Confidence | 0,95 | Prob < t | 1,0000 |

**Analysis of Variance**

| **Source** | **DF** | **Sum of Squares** | **Mean Square** | **F Ratio** | **Prob > F** |
| --- | --- | --- | --- | --- | --- |
| Type | 1 | 2952568 | 2952568 | 20,9424 | <,0001* |
| Error | 144 | 20301877 | 140985 |  |  |
| C. Total | 145 | 23254445 |  |  |  |

**Means for Oneway Anova**

| **Level** | **Number** | **Mean** | **Std Error** | **Lower 95%** | **Upper 95%** |
| --- | --- | --- | --- | --- | --- |
| benign | 73 | 248,330 | 43,947 | 161,47 | 335,19 |
| malignant | 73 | 532,746 | 43,947 | 445,88 | 619,61 |

Std Error uses a pooled estimate of error variance

**Oneway Analysis of original-glszm-SmallAreaEmphasis By Type**

**Quantiles**

| **Level** | **Minimum** | **10%** | **25%** | **Median** | **75%** | **90%** | **Maximum** |
| --- | --- | --- | --- | --- | --- | --- | --- |
| benign | 0,677469 | 0,763016 | 0,855061 | 0,9 | 0,939904 | 1 | 1 |
| malignant | 0,253802 | 0,673453 | 0,704375 | 0,734623 | 0,782922 | 0,83682 | 0,857323 |

**Oneway Anova**

**Summary of Fit**

| Rsquare | 0,482672 |
| --- | --- |
| Adj Rsquare | 0,47908 |
| Root Mean Square Error | 0,080434 |
| Mean of Response | 0,81608 |
| Observations (or Sum Wgts) | 146 |

**Pooled t Test**

malignant-benign

Assuming equal variances

| Difference | -0,15432 | t Ratio | -11,5911 |
| --- | --- | --- | --- |
| Std Err Dif | 0,01331 | DF | 144 |
| Upper CL Dif | -0,12800 | Prob > |t| | <,0001* |
| Lower CL Dif | -0,18063 | Prob > t | 1,0000 |
| Confidence | 0,95 | Prob < t | <,0001* |

**Analysis of Variance**

| **Source** | **DF** | **Sum of Squares** | **Mean Square** | **F Ratio** | **Prob > F** |
| --- | --- | --- | --- | --- | --- |
| Type | 1 | 0,8692082 | 0,869208 | 134,3536 | <,0001* |
| Error | 144 | 0,9316163 | 0,006470 |  |  |
| C. Total | 145 | 1,8008245 |  |  |  |

**Means for Oneway Anova**

| **Level** | **Number** | **Mean** | **Std Error** | **Lower 95%** | **Upper 95%** |
| --- | --- | --- | --- | --- | --- |
| benign | 73 | 0,893239 | 0,00941 | 0,87463 | 0,91185 |
| malignant | 73 | 0,738921 | 0,00941 | 0,72031 | 0,75753 |

Std Error uses a pooled estimate of error variance

**Oneway Analysis of original-glszm-LowGrayLevelZoneEmphasis By Type**

**Quantiles**

| **Level** | **Minimum** | **10%** | **25%** | **Median** | **75%** | **90%** | **Maximum** |
| --- | --- | --- | --- | --- | --- | --- | --- |
| benign | 0,031742 | 0,053411 | 0,066511 | 0,084244 | 0,1282 | 0,190098 | 0,233297 |
| malignant | 0,001198 | 0,003501 | 0,009318 | 0,019264 | 0,034214 | 0,055236 | 0,355903 |

**Oneway Anova**

**Summary of Fit**

| Rsquare | 0,390923 |
| --- | --- |
| Adj Rsquare | 0,386693 |
| Root Mean Square Error | 0,046471 |
| Mean of Response | 0,066193 |
| Observations (or Sum Wgts) | 146 |

**Pooled t Test**

malignant-benign

Assuming equal variances

| Difference | -0,07395 | t Ratio | -9,61371 |
| --- | --- | --- | --- |
| Std Err Dif | 0,00769 | DF | 144 |
| Upper CL Dif | -0,05874 | Prob > |t| | <,0001* |
| Lower CL Dif | -0,08915 | Prob > t | 1,0000 |
| Confidence | 0,95 | Prob < t | <,0001* |

**Analysis of Variance**

| **Source** | **DF** | **Sum of Squares** | **Mean Square** | **F Ratio** | **Prob > F** |
| --- | --- | --- | --- | --- | --- |
| Type | 1 | 0,19959040 | 0,199590 | 92,4234 | <,0001* |
| Error | 144 | 0,31097132 | 0,002160 |  |  |
| C. Total | 145 | 0,51056172 |  |  |  |

**Means for Oneway Anova**

| **Level** | **Number** | **Mean** | **Std Error** | **Lower 95%** | **Upper 95%** |
| --- | --- | --- | --- | --- | --- |
| benign | 73 | 0,103167 | 0,00544 | 0,09242 | 0,11392 |
| malignant | 73 | 0,029219 | 0,00544 | 0,01847 | 0,03997 |

Std Error uses a pooled estimate of error variance

**Oneway Analysis of original-glszm-ZoneEntropy By Type**

**Quantiles**

| **Level** | **Minimum** | **10%** | **25%** | **Median** | **75%** | **90%** | **Maximum** |
| --- | --- | --- | --- | --- | --- | --- | --- |
| benign | 2,251629 | 2,616391 | 3 | 3,57782 | 3,836592 | 4,188955 | 4,582901 |
| malignant | 2 | 4,244047 | 4,940451 | 5,589063 | 6,084355 | 6,5884 | 7,305317 |

**Oneway Anova**

**Summary of Fit**

| Rsquare | 0,651229 |
| --- | --- |
| Adj Rsquare | 0,648807 |
| Root Mean Square Error | 0,745978 |
| Mean of Response | 4,472933 |
| Observations (or Sum Wgts) | 146 |

**Pooled t Test**

malignant-benign

Assuming equal variances

| Difference | 2,02468 | t Ratio | 16,3975 |
| --- | --- | --- | --- |
| Std Err Dif | 0,12348 | DF | 144 |
| Upper CL Dif | 2,26874 | Prob > |t| | <,0001* |
| Lower CL Dif | 1,78063 | Prob > t | <,0001* |
| Confidence | 0,95 | Prob < t | 1,0000 |

**Analysis of Variance**

| **Source** | **DF** | **Sum of Squares** | **Mean Square** | **F Ratio** | **Prob > F** |
| --- | --- | --- | --- | --- | --- |
| Type | 1 | 149,62604 | 149,626 | 268,8780 | <,0001* |
| Error | 144 | 80,13355 | 0,556 |  |  |
| C. Total | 145 | 229,75960 |  |  |  |

**Means for Oneway Anova**

| **Level** | **Number** | **Mean** | **Std Error** | **Lower 95%** | **Upper 95%** |
| --- | --- | --- | --- | --- | --- |
| benign | 73 | 3,46059 | 0,08731 | 3,2880 | 3,6332 |
| malignant | 73 | 5,48528 | 0,08731 | 5,3127 | 5,6579 |

Std Error uses a pooled estimate of error variance

**Oneway Analysis of original-glszm-SmallAreaLowGrayLevelEmphasis By Type**

**Quantiles**

| **Level** | **Minimum** | **10%** | **25%** | **Median** | **75%** | **90%** | **Maximum** |
| --- | --- | --- | --- | --- | --- | --- | --- |
| benign | 0,023301 | 0,040477 | 0,059777 | 0,080392 | 0,12309 | 0,177675 | 0,230746 |
| malignant | 0,001018 | 0,002902 | 0,008261 | 0,016752 | 0,027004 | 0,047371 | 0,108879 |

**Oneway Anova**

**Summary of Fit**

| Rsquare | 0,506028 |
| --- | --- |
| Adj Rsquare | 0,502598 |
| Root Mean Square Error | 0,036948 |
| Mean of Response | 0,058419 |
| Observations (or Sum Wgts) | 146 |

**Pooled t Test**

malignant-benign

Assuming equal variances

| Difference | -0,07428 | t Ratio | -12,1456 |
| --- | --- | --- | --- |
| Std Err Dif | 0,00612 | DF | 144 |
| Upper CL Dif | -0,06219 | Prob > |t| | <,0001* |
| Lower CL Dif | -0,08637 | Prob > t | 1,0000 |
| Confidence | 0,95 | Prob < t | <,0001* |

**Analysis of Variance**

| **Source** | **DF** | **Sum of Squares** | **Mean Square** | **F Ratio** | **Prob > F** |
| --- | --- | --- | --- | --- | --- |
| Type | 1 | 0,20137654 | 0,201377 | 147,5146 | <,0001* |
| Error | 144 | 0,19657867 | 0,001365 |  |  |
| C. Total | 145 | 0,39795521 |  |  |  |

**Means for Oneway Anova**

| **Level** | **Number** | **Mean** | **Std Error** | **Lower 95%** | **Upper 95%** |
| --- | --- | --- | --- | --- | --- |
| benign | 73 | 0,095558 | 0,00432 | 0,08701 | 0,10411 |
| malignant | 73 | 0,021281 | 0,00432 | 0,01273 | 0,02983 |

Std Error uses a pooled estimate of error variance

**Oneway Analysis of original-ngtdm-Coarseness By Type**

**Quantiles**

| **Level** | **Minimum** | **10%** | **25%** | **Median** | **75%** | **90%** | **Maximum** |
| --- | --- | --- | --- | --- | --- | --- | --- |
| benign | 0,071109 | 0,101377 | 0,11835 | 0,156692 | 0,206443 | 0,256718 | 0,385604 |
| malignant | 0,00052 | 0,005435 | 0,016601 | 0,030862 | 0,053842 | 0,083059 | 0,14397 |

**Oneway Anova**

**Summary of Fit**

| Rsquare | 0,612901 |
| --- | --- |
| Adj Rsquare | 0,610213 |
| Root Mean Square Error | 0,052715 |
| Mean of Response | 0,105007 |
| Observations (or Sum Wgts) | 146 |

**Pooled t Test**

malignant-benign

Assuming equal variances

| Difference | -0,13175 | t Ratio | -15,0996 |
| --- | --- | --- | --- |
| Std Err Dif | 0,00873 | DF | 144 |
| Upper CL Dif | -0,11450 | Prob > |t| | <,0001* |
| Lower CL Dif | -0,14900 | Prob > t | 1,0000 |
| Confidence | 0,95 | Prob < t | <,0001* |

**Analysis of Variance**

| **Source** | **DF** | **Sum of Squares** | **Mean Square** | **F Ratio** | **Prob > F** |
| --- | --- | --- | --- | --- | --- |
| Type | 1 | 0,6335670 | 0,633567 | 227,9979 | <,0001* |
| Error | 144 | 0,4001513 | 0,002779 |  |  |
| C. Total | 145 | 1,0337183 |  |  |  |

**Means for Oneway Anova**

| **Level** | **Number** | **Mean** | **Std Error** | **Lower 95%** | **Upper 95%** |
| --- | --- | --- | --- | --- | --- |
| benign | 73 | 0,170882 | 0,00617 | 0,15869 | 0,18308 |
| malignant | 73 | 0,039133 | 0,00617 | 0,02694 | 0,05133 |

Std Error uses a pooled estimate of error variance

**Oneway Analysis of original-ngtdm-Complexity By Type**

**Quantiles**

| **Level** | **Minimum** | **10%** | **25%** | **Median** | **75%** | **90%** | **Maximum** |
| --- | --- | --- | --- | --- | --- | --- | --- |
| benign | 32,93552 | 102,3458 | 186,4033 | 384,5423 | 692,4101 | 1013,182 | 2805,706 |
| malignant | 3,066231 | 607,9684 | 1075,173 | 1726,089 | 3771,284 | 5913,432 | 21094,04 |

**Oneway Anova**

**Summary of Fit**

| Rsquare | 0,223436 |
| --- | --- |
| Adj Rsquare | 0,218044 |
| Root Mean Square Error | 2245,442 |
| Mean of Response | 1669,695 |
| Observations (or Sum Wgts) | 146 |

**Pooled t Test**

malignant-benign

Assuming equal variances

| Difference | 2392,35 | t Ratio | 6,436794 |
| --- | --- | --- | --- |
| Std Err Dif | 371,67 | DF | 144 |
| Upper CL Dif | 3126,98 | Prob > |t| | <,0001* |
| Lower CL Dif | 1657,72 | Prob > t | <,0001* |
| Confidence | 0,95 | Prob < t | 1,0000 |

**Analysis of Variance**

| **Source** | **DF** | **Sum of Squares** | **Mean Square** | **F Ratio** | **Prob > F** |
| --- | --- | --- | --- | --- | --- |
| Type | 1 | 208902112 | 208902112 | 41,4323 | <,0001* |
| Error | 144 | 726049179 | 5042008,2 |  |  |
| C. Total | 145 | 934951291 |  |  |  |

**Means for Oneway Anova**

| **Level** | **Number** | **Mean** | **Std Error** | **Lower 95%** | **Upper 95%** |
| --- | --- | --- | --- | --- | --- |
| benign | 73 | 473,52 | 262,81 | -46 | 993,0 |
| malignant | 73 | 2865,87 | 262,81 | 2346 | 3385,3 |

Std Error uses a pooled estimate of error variance

**Oneway Analysis of original-ngtdm-Strength By Type**

**Quantiles**

| **Level** | **Minimum** | **10%** | **25%** | **Median** | **75%** | **90%** | **Maximum** |
| --- | --- | --- | --- | --- | --- | --- | --- |
| benign | 5,096017 | 11,64566 | 16,24132 | 22,52092 | 34,3996 | 48,35869 | 141,1547 |
| malignant | 0,789187 | 3,826236 | 7,17065 | 11,38472 | 19,55028 | 28,53433 | 38,38237 |

**Oneway Anova**

**Summary of Fit**

| Rsquare | 0,170688 |
| --- | --- |
| Adj Rsquare | 0,164929 |
| Root Mean Square Error | 16,24975 |
| Mean of Response | 21,30882 |
| Observations (or Sum Wgts) | 146 |

**Pooled t Test**

malignant-benign

Assuming equal variances

| Difference | -14,643 | t Ratio | -5,44406 |
| --- | --- | --- | --- |
| Std Err Dif | 2,690 | DF | 144 |
| Upper CL Dif | -9,326 | Prob > |t| | <,0001* |
| Lower CL Dif | -19,959 | Prob > t | 1,0000 |
| Confidence | 0,95 | Prob < t | <,0001* |

**Analysis of Variance**

| **Source** | **DF** | **Sum of Squares** | **Mean Square** | **F Ratio** | **Prob > F** |
| --- | --- | --- | --- | --- | --- |
| Type | 1 | 7825,997 | 7826,00 | 29,6378 | <,0001* |
| Error | 144 | 38023,812 | 264,05 |  |  |
| C. Total | 145 | 45849,808 |  |  |  |

**Means for Oneway Anova**

| **Level** | **Number** | **Mean** | **Std Error** | **Lower 95%** | **Upper 95%** |
| --- | --- | --- | --- | --- | --- |
| benign | 73 | 28,6302 | 1,9019 | 24,871 | 32,389 |
| malignant | 73 | 13,9874 | 1,9019 | 10,228 | 17,747 |

Std Error uses a pooled estimate of error variance

**Oneway Analysis of original-ngtdm-Contrast By Type**

**Quantiles**

| **Level** | **Minimum** | **10%** | **25%** | **Median** | **75%** | **90%** | **Maximum** |
| --- | --- | --- | --- | --- | --- | --- | --- |
| benign | 0,365143 | 0,696442 | 1,037203 | 1,792772 | 4,122019 | 9,683724 | 100,2555 |
| malignant | 0,049897 | 0,206265 | 0,272584 | 0,44951 | 0,724701 | 1,677791 | 2,530431 |

**Oneway Anova**

**Summary of Fit**

| Rsquare | 0,055185 |
| --- | --- |
| Adj Rsquare | 0,048623 |
| Root Mean Square Error | 10,13069 |
| Mean of Response | 3,080717 |
| Observations (or Sum Wgts) | 146 |

**Pooled t Test**

malignant-benign

Assuming equal variances

| Difference | -4,8631 | t Ratio | -2,90013 |
| --- | --- | --- | --- |
| Std Err Dif | 1,6768 | DF | 144 |
| Upper CL Dif | -1,5487 | Prob > |t| | 0,0043* |
| Lower CL Dif | -8,1775 | Prob > t | 0,9978 |
| Confidence | 0,95 | Prob < t | 0,0022* |

**Analysis of Variance**

| **Source** | **DF** | **Sum of Squares** | **Mean Square** | **F Ratio** | **Prob > F** |
| --- | --- | --- | --- | --- | --- |
| Type | 1 | 863,202 | 863,202 | 8,4107 | 0,0043* |
| Error | 144 | 14778,849 | 102,631 |  |  |
| C. Total | 145 | 15642,050 |  |  |  |

**Means for Oneway Anova**

| **Level** | **Number** | **Mean** | **Std Error** | **Lower 95%** | **Upper 95%** |
| --- | --- | --- | --- | --- | --- |
| benign | 73 | 5,51225 | 1,1857 | 3,169 | 7,8559 |
| malignant | 73 | 0,64919 | 1,1857 | -1,694 | 2,9928 |

Std Error uses a pooled estimate of error variance

**Oneway Analysis of original-ngtdm-Busyness By Type**

**Quantiles**

| **Level** | **Minimum** | **10%** | **25%** | **Median** | **75%** | **90%** | **Maximum** |
| --- | --- | --- | --- | --- | --- | --- | --- |
| benign | 0,025629 | 0,031483 | 0,041422 | 0,067302 | 0,103086 | 0,145806 | 0,416898 |
| malignant | 0,023412 | 0,035615 | 0,045848 | 0,069182 | 0,105001 | 0,179004 | 1,295467 |

**Oneway Anova**

**Summary of Fit**

| Rsquare | 0,009205 |
| --- | --- |
| Adj Rsquare | 0,002324 |
| Root Mean Square Error | 0,120329 |
| Mean of Response | 0,095219 |
| Observations (or Sum Wgts) | 146 |

**Pooled t Test**

malignant-benign

Assuming equal variances

| Difference | 0,02304 | t Ratio | 1,156623 |
| --- | --- | --- | --- |
| Std Err Dif | 0,01992 | DF | 144 |
| Upper CL Dif | 0,06240 | Prob > |t| | 0,2493 |
| Lower CL Dif | -0,01633 | Prob > t | 0,1247 |
| Confidence | 0,95 | Prob < t | 0,8753 |

**Analysis of Variance**

| **Source** | **DF** | **Sum of Squares** | **Mean Square** | **F Ratio** | **Prob > F** |
| --- | --- | --- | --- | --- | --- |
| Type | 1 | 0,0193699 | 0,019370 | 1,3378 | 0,2493 |
| Error | 144 | 2,0850002 | 0,014479 |  |  |
| C. Total | 145 | 2,1043701 |  |  |  |

**Means for Oneway Anova**

| **Level** | **Number** | **Mean** | **Std Error** | **Lower 95%** | **Upper 95%** |
| --- | --- | --- | --- | --- | --- |
| benign | 73 | 0,083701 | 0,01408 | 0,05586 | 0,11154 |
| malignant | 73 | 0,106737 | 0,01408 | 0,07890 | 0,13457 |

Std Error uses a pooled estimate of error variance
