# Supplementary material for: Comparison of machine learning algorithms to predict clinically significant prostate cancer of the peripheral zone with multiparametric MRI using clinical assessment categories and radiomic features
Source: Eur Radiol. 2020 Jul 16;30(12):6757–69. doi: 10.1007/s00330-020-07064-5 (PMC7599168; doi:10.1007/s00330-020-07064-5)
Supplement: Supplementary file 3 — (DOC 5388 kb) [file 330_2020_7064_MOESM3_ESM.doc]

**Fit Group**

**Oneway Analysis of original-shape-Maximum3DDiameter By GrG**

**Quantiles**

| **Level** | **Minimum** | **10%** | **25%** | **Median** | **75%** | **90%** | **Maximum** |
| --- | --- | --- | --- | --- | --- | --- | --- |
| 1 | 9,16903 | 9,452558 | 15,23796 | 17,75137 | 20,18404 | 25,71816 | 25,96578 |
| 2 | 8,564527 | 9,746004 | 12,08562 | 18,74459 | 21,67435 | 46,79036 | 57,28075 |
| 3 | 12,79444 | 13,81389 | 17,74587 | 24,17124 | 25,82849 | 35,65911 | 37,70306 |
| 4&5 | 11,33647 | 12,52576 | 16,55224 | 25,4865 | 43,56057 | 57,47545 | 89,77997 |

**Nonparametric Comparisons For Each Pair Using Wilcoxon Method**

| **q*** | **Alpha** |
| --- | --- |
| 1,95996 | 0,05 |

| **Level** | **- Level** | **Score Mean Difference** | **Std Err Dif** | **Z** | **p-Value** | **Hodges-Lehmann** | **Lower CL** | **Upper CL** | **Difference Plot** |
| --- | --- | --- | --- | --- | --- | --- | --- | --- | --- |
| 4&5 | 1 | 8,808333 | 3,500000 | 2,516667 | 0,0118* | 8,510838 | 2,16744 | 17,70954 |  |
| 4&5 | 2 | 8,179348 | 3,839063 | 2,130558 | 0,0331* | 6,882739 | 0,45074 | 14,81103 |  |
| 3 | 1 | 6,666667 | 3,214550 | 2,073903 | 0,0381* | 5,039907 | 0,02600 | 9,15183 |  |
| 3 | 2 | 4,956522 | 3,688211 | 1,343882 | 0,1790 | 4,140845 | -2,51656 | 8,35734 |  |
| 4&5 | 3 | 4,258333 | 3,500000 | 1,216667 | 0,2237 | 3,827778 | -2,63277 | 12,78271 |  |
| 2 | 1 | 2,313043 | 3,688211 | 0,627145 | 0,5306 | 0,881452 | -3,83727 | 6,35494 |  |

**Oneway Analysis of original-shape-Maximum2DDiameterSlice By GrG**

**Quantiles**

| **Level** | **Minimum** | **10%** | **25%** | **Median** | **75%** | **90%** | **Maximum** |
| --- | --- | --- | --- | --- | --- | --- | --- |
| 1 | 7,18022 | 8,238651 | 9,614804 | 12,64911 | 17,93817 | 21,7782 | 23,85139 |
| 2 | 6,666667 | 7,509841 | 9,575404 | 13,92041 | 20,82733 | 36,41574 | 56,01587 |
| 3 | 7,333333 | 10,02812 | 13,33333 | 19,00292 | 22,70585 | 30,72765 | 34,06401 |
| 4&5 | 10,74968 | 11,42056 | 12,25882 | 23,25903 | 37,32487 | 56,21711 | 72,60242 |

**Nonparametric Comparisons For Each Pair Using Wilcoxon Method**

| **q*** | **Alpha** |
| --- | --- |
| 1,95996 | 0,05 |

| **Level** | **- Level** | **Score Mean Difference** | **Std Err Dif** | **Z** | **p-Value** | **Hodges-Lehmann** | **Lower CL** | **Upper CL** | **Difference Plot** |
| --- | --- | --- | --- | --- | --- | --- | --- | --- | --- |
| 4&5 | 1 | 10,26667 | 3,499510 | 2,933744 | 0,0033* | 9,839010 | 2,45904 | 18,03119 |  |
| 4&5 | 2 | 7,99239 | 3,838338 | 2,082253 | 0,0373* | 7,184636 | 0,25452 | 14,55632 |  |
| 3 | 1 | 6,66667 | 3,213477 | 2,074596 | 0,0380* | 4,216370 | 0,63302 | 9,66706 |  |
| 4&5 | 3 | 4,60833 | 3,498774 | 1,317128 | 0,1878 | 4,794491 | -1,25949 | 14,00472 |  |
| 3 | 2 | 4,24058 | 3,686394 | 1,150333 | 0,2500 | 3,616853 | -2,88737 | 7,89573 |  |
| 2 | 1 | 1,21159 | 3,687404 | 0,328577 | 0,7425 | 0,930130 | -3,02406 | 6,60590 |  |

**Oneway Analysis of original-shape-Sphericity By GrG**

**Quantiles**

| **Level** | **Minimum** | **10%** | **25%** | **Median** | **75%** | **90%** | **Maximum** |
| --- | --- | --- | --- | --- | --- | --- | --- |
| 1 | 0,471726 | 0,495463 | 0,52914 | 0,63333 | 0,693988 | 0,825501 | 0,873928 |
| 2 | 0,416992 | 0,4447 | 0,476345 | 0,609375 | 0,675789 | 0,810019 | 0,890704 |
| 3 | 0,502732 | 0,519986 | 0,592926 | 0,636532 | 0,656436 | 0,712828 | 0,753439 |
| 4&5 | 0,367849 | 0,394929 | 0,44372 | 0,542058 | 0,698314 | 0,735435 | 0,738174 |

**Nonparametric Comparisons For Each Pair Using Wilcoxon Method**

| **q*** | **Alpha** |
| --- | --- |
| 1,95996 | 0,05 |

| **Level** | **- Level** | **Score Mean Difference** | **Std Err Dif** | **Z** | **p-Value** | **Hodges-Lehmann** | **Lower CL** | **Upper CL** | **Difference Plot** |
| --- | --- | --- | --- | --- | --- | --- | --- | --- | --- |
| 3 | 2 | 2,09275 | 3,688211 | 0,56742 | 0,5704 | 0,021420 | -0,049047 | 0,1005613 |  |
| 3 | 1 | 0,66667 | 3,214550 | 0,20739 | 0,8357 | 0,005029 | -0,062883 | 0,0769184 |  |
| 2 | 1 | -1,98261 | 3,688211 | -0,53755 | 0,5909 | -0,021814 | -0,106695 | 0,0621924 |  |
| 4&5 | 2 | -4,34674 | 3,839063 | -1,13224 | 0,2575 | -0,044443 | -0,136172 | 0,0379219 |  |
| 4&5 | 1 | -5,07500 | 3,500000 | -1,45000 | 0,1471 | -0,075581 | -0,160731 | 0,0250977 |  |
| 4&5 | 3 | -5,54167 | 3,500000 | -1,58333 | 0,1133 | -0,078702 | -0,163639 | 0,0333315 |  |

**Oneway Analysis of original-shape-MinorAxis By GrG**

**Quantiles**

| **Level** | **Minimum** | **10%** | **25%** | **Median** | **75%** | **90%** | **Maximum** |
| --- | --- | --- | --- | --- | --- | --- | --- |
| 1 | 6,590699 | 7,007812 | 7,954068 | 10,93495 | 13,25952 | 17,44397 | 18,16972 |
| 2 | 6,48417 | 6,935256 | 8,231931 | 12,30077 | 14,38594 | 29,74722 | 33,44799 |
| 3 | 6,463433 | 8,808441 | 10,67523 | 13,73165 | 15,89676 | 24,92239 | 27,14634 |
| 4&5 | 7,10876 | 8,04347 | 10,62654 | 16,28056 | 31,29091 | 37,32846 | 60,20007 |

**Nonparametric Comparisons For Each Pair Using Wilcoxon Method**

| **q*** | **Alpha** |
| --- | --- |
| 1,95996 | 0,05 |

| **Level** | **- Level** | **Score Mean Difference** | **Std Err Dif** | **Z** | **p-Value** | **Hodges-Lehmann** | **Lower CL** | **Upper CL** | **Difference Plot** |
| --- | --- | --- | --- | --- | --- | --- | --- | --- | --- |
| 4&5 | 1 | 8,458333 | 3,500000 | 2,416667 | 0,0157* | 5,027385 | 1,05265 | 10,01212 |  |
| 4&5 | 2 | 7,711957 | 3,839063 | 2,008812 | 0,0446* | 3,677400 | 0,10721 | 8,52400 |  |
| 3 | 1 | 5,333333 | 3,214550 | 1,659123 | 0,0971 | 2,957733 | -0,48115 | 5,68008 |  |
| 4&5 | 3 | 4,375000 | 3,500000 | 1,250000 | 0,2113 | 2,404467 | -1,53852 | 7,44621 |  |
| 3 | 2 | 3,524638 | 3,688211 | 0,955650 | 0,3392 | 1,523539 | -1,96320 | 4,19081 |  |
| 2 | 1 | 3,084058 | 3,688211 | 0,836194 | 0,4030 | 1,137141 | -1,56636 | 4,82543 |  |

**Oneway Analysis of original-shape-Elongation By GrG**

**Quantiles**

| **Level** | **Minimum** | **10%** | **25%** | **Median** | **75%** | **90%** | **Maximum** |
| --- | --- | --- | --- | --- | --- | --- | --- |
| 1 | 0,311951 | 0,434095 | 0,555655 | 0,645088 | 0,839714 | 0,881768 | 0,883093 |
| 2 | 0,417204 | 0,47509 | 0,628634 | 0,701265 | 0,824134 | 0,845274 | 0,901339 |
| 3 | 0,445431 | 0,525589 | 0,604995 | 0,710618 | 0,770283 | 0,841249 | 0,871909 |
| 4&5 | 0,384147 | 0,5247 | 0,650911 | 0,796145 | 0,880484 | 0,904015 | 0,908154 |

**Nonparametric Comparisons For Each Pair Using Wilcoxon Method**

| **q*** | **Alpha** |
| --- | --- |
| 1,95996 | 0,05 |

| **Level** | **- Level** | **Score Mean Difference** | **Std Err Dif** | **Z** | **p-Value** | **Hodges-Lehmann** | **Lower CL** | **Upper CL** | **Difference Plot** |
| --- | --- | --- | --- | --- | --- | --- | --- | --- | --- |
| 4&5 | 1 | 5,77500 | 3,500000 | 1,65000 | 0,0989 | 0,060129 | -0,022699 | 0,2073330 |  |
| 4&5 | 2 | 5,37500 | 3,839063 | 1,40008 | 0,1615 | 0,060535 | -0,025234 | 0,1558290 |  |
| 4&5 | 3 | 4,72500 | 3,500000 | 1,35000 | 0,1770 | 0,072171 | -0,031031 | 0,1550169 |  |
| 2 | 1 | 1,76232 | 3,688211 | 0,47782 | 0,6328 | 0,018246 | -0,097995 | 0,1314686 |  |
| 3 | 1 | 1,46667 | 3,214550 | 0,45626 | 0,6482 | 0,025524 | -0,084264 | 0,1321256 |  |
| 3 | 2 | -0,44058 | 3,688211 | -0,11946 | 0,9049 | -0,008518 | -0,087993 | 0,0824872 |  |

**Oneway Analysis of original-shape-SurfaceVolumeRatio By GrG**

**Quantiles**

| **Level** | **Minimum** | **10%** | **25%** | **Median** | **75%** | **90%** | **Maximum** |
| --- | --- | --- | --- | --- | --- | --- | --- |
| 1 | 0,649806 | 0,6576 | 0,791759 | 0,851549 | 0,993273 | 1,092231 | 1,094571 |
| 2 | 0,410717 | 0,500846 | 0,697247 | 0,81989 | 0,965203 | 1,181421 | 1,435417 |
| 3 | 0,464691 | 0,467926 | 0,568309 | 0,623898 | 0,857735 | 0,949019 | 1,001432 |
| 4&5 | 0,236315 | 0,335665 | 0,553363 | 0,678691 | 0,841901 | 0,973968 | 1,192864 |

**Nonparametric Comparisons For Each Pair Using Wilcoxon Method**

| **q*** | **Alpha** |
| --- | --- |
| 1,95996 | 0,05 |

| **Level** | **- Level** | **Score Mean Difference** | **Std Err Dif** | **Z** | **p-Value** | **Hodges-Lehmann** | **Lower CL** | **Upper CL** | **Difference Plot** |
| --- | --- | --- | --- | --- | --- | --- | --- | --- | --- |
| 4&5 | 3 | -0,29167 | 3,500000 | -0,08333 | 0,9336 | -0,003001 | -0,156706 | 0,160452 |  |
| 2 | 1 | -2,53333 | 3,688211 | -0,68687 | 0,4922 | -0,037516 | -0,178496 | 0,105631 |  |
| 4&5 | 2 | -7,52500 | 3,839063 | -1,96011 | 0,0500* | -0,155149 | -0,321310 | 0,002475 |  |
| 3 | 2 | -7,71014 | 3,688211 | -2,09048 | 0,0366* | -0,167343 | -0,310643 | -0,016040 |  |
| 3 | 1 | -9,06667 | 3,214550 | -2,82051 | 0,0048* | -0,206763 | -0,338017 | -0,072242 |  |
| 4&5 | 1 | -9,50833 | 3,500000 | -2,71667 | 0,0066* | -0,209739 | -0,344610 | -0,059130 |  |

**Oneway Analysis of original-shape-Volume By GrG**

**Quantiles**

| **Level** | **Minimum** | **10%** | **25%** | **Median** | **75%** | **90%** | **Maximum** |
| --- | --- | --- | --- | --- | --- | --- | --- |
| 1 | 172,8 | 257,2799 | 339,2001 | 774,4005 | 1312,002 | 2344,961 | 2636,799 |
| 2 | 123,904 | 209,9202 | 345,5991 | 805,3757 | 1685,334 | 9260,798 | 18630,39 |
| 3 | 263,2959 | 485,479 | 735,6805 | 1875,199 | 2508,799 | 7931,834 | 8568,79 |
| 4&5 | 179,1999 | 375,6799 | 757,7168 | 2246,4 | 11201,07 | 26505,59 | 142600,5 |

**Nonparametric Comparisons For Each Pair Using Wilcoxon Method**

| **q*** | **Alpha** |
| --- | --- |
| 1,95996 | 0,05 |

| **Level** | **- Level** | **Score Mean Difference** | **Std Err Dif** | **Z** | **p-Value** | **Hodges-Lehmann** | **Lower CL** | **Upper CL** | **Difference Plot** |
| --- | --- | --- | --- | --- | --- | --- | --- | --- | --- |
| 4&5 | 1 | 9,741667 | 3,500000 | 2,783333 | 0,0054* | 1417,214 | 254,934 | 2643,199 |  |
| 4&5 | 2 | 8,179348 | 3,838918 | 2,130639 | 0,0331* | 1231,468 | 70,400 | 2205,698 |  |
| 3 | 1 | 7,066667 | 3,214550 | 2,198338 | 0,0279* | 697,604 | 44,802 | 1593,601 |  |
| 3 | 2 | 5,507246 | 3,688009 | 1,493284 | 0,1354 | 537,601 | -179,200 | 1388,801 |  |
| 4&5 | 3 | 3,558333 | 3,500000 | 1,016667 | 0,3093 | 494,401 | -467,198 | 2066,306 |  |
| 2 | 1 | 1,762319 | 3,688009 | 0,477851 | 0,6328 | 101,631 | -320,003 | 652,800 |  |

**Oneway Analysis of original-shape-MajorAxis By GrG**

**Quantiles**

| **Level** | **Minimum** | **10%** | **25%** | **Median** | **75%** | **90%** | **Maximum** |
| --- | --- | --- | --- | --- | --- | --- | --- |
| 1 | 9,100898 | 9,838509 | 15,4291 | 17,02723 | 18,79017 | 24,26853 | 25,63755 |
| 2 | 9,399655 | 10,92082 | 12,26455 | 16,90858 | 22,28438 | 42,02452 | 53,20742 |
| 3 | 13,4649 | 14,09227 | 17,64516 | 20,47631 | 21,95763 | 30,05934 | 31,13438 |
| 4&5 | 11,25113 | 11,50448 | 15,9552 | 21,16472 | 36,67611 | 51,26666 | 70,62249 |

**Nonparametric Comparisons For Each Pair Using Wilcoxon Method**

| **q*** | **Alpha** |
| --- | --- |
| 1,95996 | 0,05 |

| **Level** | **- Level** | **Score Mean Difference** | **Std Err Dif** | **Z** | **p-Value** | **Hodges-Lehmann** | **Lower CL** | **Upper CL** | **Difference Plot** |
| --- | --- | --- | --- | --- | --- | --- | --- | --- | --- |
| 4&5 | 1 | 7,408333 | 3,500000 | 2,116667 | 0,0343* | 5,126623 | 0,52266 | 14,22865 |  |
| 4&5 | 2 | 6,216304 | 3,839063 | 1,619224 | 0,1054 | 3,978004 | -0,52963 | 9,86225 |  |
| 3 | 1 | 5,333333 | 3,214550 | 1,659123 | 0,0971 | 3,045097 | -0,48327 | 6,04696 |  |
| 3 | 2 | 3,744928 | 3,688211 | 1,015378 | 0,3099 | 2,585782 | -1,80807 | 6,08163 |  |
| 4&5 | 3 | 3,208333 | 3,500000 | 0,916667 | 0,3593 | 1,896803 | -2,67220 | 10,37061 |  |
| 2 | 1 | 0,881159 | 3,688211 | 0,238912 | 0,8112 | 0,232252 | -3,24821 | 5,32425 |  |

**Oneway Analysis of original-shape-SurfaceArea By GrG**

**Quantiles**

| **Level** | **Minimum** | **10%** | **25%** | **Median** | **75%** | **90%** | **Maximum** |
| --- | --- | --- | --- | --- | --- | --- | --- |
| 1 | 189,142 | 237,1412 | 342,0344 | 685,0611 | 921,9437 | 1630,159 | 1747,658 |
| 2 | 177,8539 | 221,697 | 307,0848 | 733,2125 | 1223,994 | 4678,348 | 7651,818 |
| 3 | 263,6729 | 431,5464 | 650,2735 | 1138,258 | 1565,234 | 3704,331 | 4028,037 |
| 4&5 | 213,7611 | 341,7467 | 576,9201 | 1633,066 | 6074,257 | 8933,813 | 33698,62 |

**Nonparametric Comparisons For Each Pair Using Wilcoxon Method**

| **q*** | **Alpha** |
| --- | --- |
| 1,95996 | 0,05 |

| **Level** | **- Level** | **Score Mean Difference** | **Std Err Dif** | **Z** | **p-Value** | **Hodges-Lehmann** | **Lower CL** | **Upper CL** | **Difference Plot** |
| --- | --- | --- | --- | --- | --- | --- | --- | --- | --- |
| 4&5 | 1 | 8,691667 | 3,500000 | 2,483333 | 0,0130* | 888,5272 | 142,589 | 1503,035 |  |
| 4&5 | 2 | 7,898913 | 3,839063 | 2,057511 | 0,0396* | 822,0948 | 24,646 | 1399,120 |  |
| 3 | 1 | 5,733333 | 3,214550 | 1,783557 | 0,0745 | 315,8081 | -100,634 | 796,223 |  |
| 4&5 | 3 | 4,841667 | 3,500000 | 1,383333 | 0,1666 | 543,2351 | -207,400 | 1223,469 |  |
| 3 | 2 | 4,185507 | 3,688211 | 1,134834 | 0,2564 | 304,2617 | -179,435 | 673,193 |  |
| 2 | 1 | 0,991304 | 3,688211 | 0,268776 | 0,7881 | 48,1514 | -232,860 | 489,332 |  |

**Oneway Analysis of original-shape-Flatness By GrG**

**Quantiles**

| **Level** | **Minimum** | **10%** | **25%** | **Median** | **75%** | **90%** | **Maximum** |
| --- | --- | --- | --- | --- | --- | --- | --- |
| 1 | 0,21389 | 0,269206 | 0,380375 | 0,477257 | 0,62814 | 0,74513 | 0,770513 |
| 2 | 0,266319 | 0,310648 | 0,35668 | 0,453133 | 0,550275 | 0,689977 | 0,72534 |
| 3 | 0,336179 | 0,360767 | 0,429818 | 0,525712 | 0,59647 | 0,63265 | 0,671851 |
| 4&5 | 0,306632 | 0,325125 | 0,486336 | 0,556191 | 0,681125 | 0,751339 | 0,78413 |

**Nonparametric Comparisons For Each Pair Using Wilcoxon Method**

| **q*** | **Alpha** |
| --- | --- |
| 1,95996 | 0,05 |

| **Level** | **- Level** | **Score Mean Difference** | **Std Err Dif** | **Z** | **p-Value** | **Hodges-Lehmann** | **Lower CL** | **Upper CL** | **Difference Plot** |
| --- | --- | --- | --- | --- | --- | --- | --- | --- | --- |
| 4&5 | 2 | 8,74022 | 3,839063 | 2,27665 | 0,0228* | 0,109645 | 0,013466 | 0,1951365 |  |
| 3 | 2 | 4,95652 | 3,688211 | 1,34388 | 0,1790 | 0,061494 | -0,036818 | 0,1433377 |  |
| 4&5 | 1 | 4,37500 | 3,500000 | 1,25000 | 0,2113 | 0,065138 | -0,048492 | 0,1707077 |  |
| 4&5 | 3 | 3,90833 | 3,500000 | 1,11667 | 0,2641 | 0,044819 | -0,051067 | 0,1397374 |  |
| 3 | 1 | 0,40000 | 3,214550 | 0,12443 | 0,9010 | 0,011753 | -0,098041 | 0,1261215 |  |
| 2 | 1 | -2,86377 | 3,688211 | -0,77647 | 0,4375 | -0,039764 | -0,143799 | 0,0662067 |  |

**Oneway Analysis of original-shape-LeastAxis By GrG**

**Quantiles**

| **Level** | **Minimum** | **10%** | **25%** | **Median** | **75%** | **90%** | **Maximum** |
| --- | --- | --- | --- | --- | --- | --- | --- |
| 1 | 4,383515 | 4,586959 | 6,476729 | 8,064879 | 9,918665 | 12,84816 | 13,28034 |
| 2 | 3,803878 | 4,322994 | 6,12478 | 8,387491 | 11,32187 | 16,78654 | 17,74243 |
| 3 | 4,878124 | 6,067796 | 7,868633 | 10,32212 | 12,35497 | 17,72568 | 17,7968 |
| 4&5 | 3,889939 | 7,062318 | 8,108619 | 11,68793 | 19,9476 | 30,90745 | 53,30986 |

**Nonparametric Comparisons For Each Pair Using Wilcoxon Method**

| **q*** | **Alpha** |
| --- | --- |
| 1,95996 | 0,05 |

| **Level** | **- Level** | **Score Mean Difference** | **Std Err Dif** | **Z** | **p-Value** | **Hodges-Lehmann** | **Lower CL** | **Upper CL** | **Difference Plot** |
| --- | --- | --- | --- | --- | --- | --- | --- | --- | --- |
| 4&5 | 1 | 9,391667 | 3,500000 | 2,683333 | 0,0073* | 3,728401 | 1,09296 | 7,289716 |  |
| 4&5 | 2 | 8,927174 | 3,839063 | 2,325352 | 0,0201* | 3,500919 | 0,67359 | 6,822267 |  |
| 3 | 1 | 5,333333 | 3,214550 | 1,659123 | 0,0971 | 2,175813 | -0,23264 | 4,552535 |  |
| 3 | 2 | 4,956522 | 3,688211 | 1,343882 | 0,1790 | 1,752680 | -0,67525 | 3,967477 |  |
| 4&5 | 3 | 4,141667 | 3,500000 | 1,183333 | 0,2367 | 1,949628 | -1,02962 | 5,775552 |  |
| 2 | 1 | 1,321739 | 3,688211 | 0,358369 | 0,7201 | 0,300333 | -1,74561 | 2,825793 |  |

**Oneway Analysis of original-shape-Maximum2DDiameterColumn By GrG**

**Quantiles**

| **Level** | **Minimum** | **10%** | **25%** | **Median** | **75%** | **90%** | **Maximum** |
| --- | --- | --- | --- | --- | --- | --- | --- |
| 1 | 6,434629 | 7,119795 | 11,25779 | 15,15314 | 17,92031 | 24,82252 | 25,93153 |
| 2 | 6,434628 | 6,979273 | 10,7629 | 16,08657 | 21,6333 | 44,06076 | 56,46096 |
| 3 | 10,27705 | 11,83238 | 15,05723 | 16,33864 | 22,72972 | 30,69856 | 32,41628 |
| 4&5 | 8,772684 | 11,25779 | 13,96456 | 20,90531 | 39,60015 | 52,26084 | 83,19999 |

**Nonparametric Comparisons For Each Pair Using Wilcoxon Method**

| **q*** | **Alpha** |
| --- | --- |
| 1,95996 | 0,05 |

| **Level** | **- Level** | **Score Mean Difference** | **Std Err Dif** | **Z** | **p-Value** | **Hodges-Lehmann** | **Lower CL** | **Upper CL** | **Difference Plot** |
| --- | --- | --- | --- | --- | --- | --- | --- | --- | --- |
| 4&5 | 1 | 7,933333 | 3,499755 | 2,266825 | 0,0234* | 6,521816 | 1,25289 | 17,14986 |  |
| 4&5 | 2 | 7,244565 | 3,839063 | 1,887066 | 0,0592 | 5,255400 | -0,25915 | 13,19030 |  |
| 4&5 | 3 | 4,141667 | 3,499755 | 1,183416 | 0,2366 | 4,215460 | -2,71665 | 13,26900 |  |
| 3 | 1 | 4,133333 | 3,214193 | 1,285963 | 0,1985 | 2,198878 | -1,36460 | 7,69376 |  |
| 3 | 2 | 2,423188 | 3,687605 | 0,657117 | 0,5111 | 2,106367 | -3,12280 | 6,70345 |  |
| 2 | 1 | 1,982609 | 3,688211 | 0,537553 | 0,5909 | 0,933429 | -3,70895 | 6,86847 |  |

**Oneway Analysis of original-shape-Maximum2DDiameterRow By GrG**

**Quantiles**

| **Level** | **Minimum** | **10%** | **25%** | **Median** | **75%** | **90%** | **Maximum** |
| --- | --- | --- | --- | --- | --- | --- | --- |
| 1 | 5,333333 | 6,933334 | 10,7629 | 15,15315 | 16,47302 | 21,17175 | 22,51557 |
| 2 | 7,774603 | 8,847676 | 10,10468 | 14,94523 | 19,69771 | 36,50119 | 37 |
| 3 | 7,7746 | 9,111973 | 13,99429 | 16,473 | 19,1949 | 33,86743 | 37,70306 |
| 4&5 | 4,480078 | 10,77878 | 14,09241 | 18,9232 | 39,61533 | 50,26776 | 81,27035 |

**Nonparametric Comparisons For Each Pair Using Wilcoxon Method**

| **q*** | **Alpha** |
| --- | --- |
| 1,95996 | 0,05 |

| **Level** | **- Level** | **Score Mean Difference** | **Std Err Dif** | **Z** | **p-Value** | **Hodges-Lehmann** | **Lower CL** | **Upper CL** | **Difference Plot** |
| --- | --- | --- | --- | --- | --- | --- | --- | --- | --- |
| 4&5 | 2 | 6,917391 | 3,838773 | 1,801980 | 0,0715 | 4,544708 | -0,43480 | 10,77951 |  |
| 4&5 | 1 | 6,358333 | 3,499265 | 1,817048 | 0,0692 | 5,099141 | -0,41047 | 13,14842 |  |
| 3 | 1 | 4,000000 | 3,212762 | 1,245035 | 0,2131 | 2,005468 | -0,92380 | 7,73616 |  |
| 4&5 | 3 | 3,091667 | 3,499755 | 0,883395 | 0,3770 | 2,848491 | -2,81393 | 10,06469 |  |
| 3 | 2 | 2,973913 | 3,688211 | 0,806329 | 0,4201 | 1,460011 | -3,12826 | 6,05589 |  |
| 2 | 1 | 1,321739 | 3,687202 | 0,358467 | 0,7200 | 0,841248 | -3,34119 | 6,09706 |  |

**Oneway Analysis of original-gldm-GrayLevelVariance By GrG**

**Quantiles**

| **Level** | **Minimum** | **10%** | **25%** | **Median** | **75%** | **90%** | **Maximum** |
| --- | --- | --- | --- | --- | --- | --- | --- |
| 1 | 28,77323 | 29,61117 | 31,51157 | 39,88664 | 72,85183 | 102,7814 | 123,2355 |
| 2 | 0,636487 | 21,89323 | 40,58629 | 58,49817 | 76,78824 | 135,0887 | 168,7429 |
| 3 | 24,79956 | 24,89283 | 28,49379 | 50,62708 | 60,08548 | 87,92077 | 94,35235 |
| 4&5 | 23,35417 | 28,04449 | 39,82375 | 55,55548 | 111,2119 | 160,2063 | 187,0951 |

**Nonparametric Comparisons For Each Pair Using Wilcoxon Method**

| **q*** | **Alpha** |
| --- | --- |
| 1,95996 | 0,05 |

| **Level** | **- Level** | **Score Mean Difference** | **Std Err Dif** | **Z** | **p-Value** | **Hodges-Lehmann** | **Lower CL** | **Upper CL** | **Difference Plot** |
| --- | --- | --- | --- | --- | --- | --- | --- | --- | --- |
| 4&5 | 3 | 4,37500 | 3,500000 | 1,25000 | 0,2113 | 13,3151 | -7,7276 | 43,05814 |  |
| 4&5 | 1 | 4,02500 | 3,500000 | 1,15000 | 0,2501 | 8,1804 | -8,0667 | 37,93022 |  |
| 2 | 1 | 3,63478 | 3,688211 | 0,98551 | 0,3244 | 8,9519 | -10,9398 | 28,32838 |  |
| 4&5 | 2 | 0,70109 | 3,839063 | 0,18262 | 0,8551 | 1,1784 | -18,7601 | 28,58738 |  |
| 3 | 1 | -1,46667 | 3,214550 | -0,45626 | 0,6482 | -2,9485 | -19,4397 | 16,46655 |  |
| 3 | 2 | -4,29565 | 3,688211 | -1,16470 | 0,2441 | -11,8767 | -27,5991 | 7,68680 |  |

**Oneway Analysis of original-gldm-HighGrayLevelEmphasis By GrG**

**Quantiles**

| **Level** | **Minimum** | **10%** | **25%** | **Median** | **75%** | **90%** | **Maximum** |
| --- | --- | --- | --- | --- | --- | --- | --- |
| 1 | 127,7736 | 157,6011 | 215,0293 | 297,9184 | 592,0534 | 940,2942 | 1115,486 |
| 2 | 4,468085 | 145,5574 | 308,9063 | 484,4757 | 864,8565 | 1000,341 | 1045,125 |
| 3 | 146,2632 | 167,67 | 245,7617 | 349,3232 | 526,9011 | 953,2922 | 1107,002 |
| 4&5 | 169,3611 | 230,9003 | 309,8215 | 504,3615 | 789,2305 | 1337,513 | 2059,788 |

**Nonparametric Comparisons For Each Pair Using Wilcoxon Method**

| **q*** | **Alpha** |
| --- | --- |
| 1,95996 | 0,05 |

| **Level** | **- Level** | **Score Mean Difference** | **Std Err Dif** | **Z** | **p-Value** | **Hodges-Lehmann** | **Lower CL** | **Upper CL** | **Difference Plot** |
| --- | --- | --- | --- | --- | --- | --- | --- | --- | --- |
| 4&5 | 1 | 6,94167 | 3,500000 | 1,98333 | 0,0473* | 161,384 | -0,841 | 426,3992 |  |
| 4&5 | 3 | 6,00833 | 3,500000 | 1,71667 | 0,0860 | 138,498 | -28,451 | 389,0571 |  |
| 2 | 1 | 5,39710 | 3,688211 | 1,46334 | 0,1434 | 132,943 | -57,545 | 346,0300 |  |
| 4&5 | 2 | 1,35543 | 3,839063 | 0,35306 | 0,7240 | 42,413 | -180,966 | 258,3829 |  |
| 3 | 1 | 1,06667 | 3,214550 | 0,33182 | 0,7400 | 27,319 | -135,018 | 164,1184 |  |
| 3 | 2 | -5,17681 | 3,688211 | -1,40361 | 0,1604 | -126,965 | -338,213 | 56,6584 |  |

**Oneway Analysis of original-gldm-DependenceEntropy By GrG**

**Quantiles**

| **Level** | **Minimum** | **10%** | **25%** | **Median** | **75%** | **90%** | **Maximum** |
| --- | --- | --- | --- | --- | --- | --- | --- |
| 1 | 4,060262 | 4,384165 | 4,734035 | 5,495845 | 5,914728 | 6,169626 | 6,364449 |
| 2 | 3,5 | 4,081086 | 4,96313 | 5,755198 | 6,317308 | 6,921043 | 7,016533 |
| 3 | 4,454822 | 4,87387 | 5,41811 | 6,049609 | 6,263235 | 6,599341 | 6,624392 |
| 4&5 | 4,164498 | 4,909155 | 5,276421 | 6,244046 | 6,80245 | 7,081889 | 7,573548 |

**Nonparametric Comparisons For Each Pair Using Wilcoxon Method**

| **q*** | **Alpha** |
| --- | --- |
| 1,95996 | 0,05 |

| **Level** | **- Level** | **Score Mean Difference** | **Std Err Dif** | **Z** | **p-Value** | **Hodges-Lehmann** | **Lower CL** | **Upper CL** | **Difference Plot** |
| --- | --- | --- | --- | --- | --- | --- | --- | --- | --- |
| 4&5 | 1 | 8,808333 | 3,500000 | 2,516667 | 0,0118* | 0,7108157 | 0,189376 | 1,291149 |  |
| 3 | 1 | 7,600000 | 3,214550 | 2,364250 | 0,0181* | 0,4981086 | 0,076611 | 1,086796 |  |
| 4&5 | 2 | 6,309783 | 3,839063 | 1,643574 | 0,1003 | 0,5094964 | -0,109915 | 1,076710 |  |
| 3 | 2 | 3,744928 | 3,688211 | 1,015378 | 0,3099 | 0,2962040 | -0,342310 | 0,869194 |  |
| 4&5 | 3 | 2,975000 | 3,500000 | 0,850000 | 0,3953 | 0,2012700 | -0,290325 | 0,773450 |  |
| 2 | 1 | 2,643478 | 3,688211 | 0,716737 | 0,4735 | 0,2702503 | -0,408598 | 0,902868 |  |

**Oneway Analysis of original-gldm-DependenceNonUniformity By GrG**

**Quantiles**

| **Level** | **Minimum** | **10%** | **25%** | **Median** | **75%** | **90%** | **Maximum** |
| --- | --- | --- | --- | --- | --- | --- | --- |
| 1 | 12,62963 | 17,33487 | 26,14286 | 38,31783 | 62,13043 | 117,0927 | 138,8835 |
| 2 | 6,914894 | 10,32353 | 28,81481 | 40,968 | 107,3966 | 382,9076 | 788,9354 |
| 3 | 11 | 23,03789 | 41,26263 | 83,00683 | 113,6327 | 302,4106 | 355,8627 |
| 4&5 | 11,14286 | 23,8746 | 33,73076 | 119,8113 | 507,425 | 961,0899 | 4518,518 |

**Nonparametric Comparisons For Each Pair Using Wilcoxon Method**

| **q*** | **Alpha** |
| --- | --- |
| 1,95996 | 0,05 |

| **Level** | **- Level** | **Score Mean Difference** | **Std Err Dif** | **Z** | **p-Value** | **Hodges-Lehmann** | **Lower CL** | **Upper CL** | **Difference Plot** |
| --- | --- | --- | --- | --- | --- | --- | --- | --- | --- |
| 4&5 | 1 | 8,925000 | 3,500000 | 2,550000 | 0,0108* | 78,08837 | 7,2952 | 133,5793 |  |
| 4&5 | 2 | 7,244565 | 3,839063 | 1,887066 | 0,0592 | 66,37709 | -1,4786 | 114,6463 |  |
| 3 | 1 | 6,666667 | 3,214550 | 2,073903 | 0,0381* | 27,95853 | 0,7723 | 70,3772 |  |
| 3 | 2 | 4,736232 | 3,688211 | 1,284154 | 0,1991 | 22,05621 | -14,5761 | 56,0635 |  |
| 4&5 | 3 | 4,258333 | 3,500000 | 1,216667 | 0,2237 | 35,75082 | -22,8250 | 109,3789 |  |
| 2 | 1 | 1,982609 | 3,688211 | 0,537553 | 0,5909 | 5,38725 | -17,3719 | 34,6040 |  |

**Oneway Analysis of original-gldm-GrayLevelNonUniformity By GrG**

**Quantiles**

| **Level** | **Minimum** | **10%** | **25%** | **Median** | **75%** | **90%** | **Maximum** |
| --- | --- | --- | --- | --- | --- | --- | --- |
| 1 | 1,888889 | 2,237188 | 3,382353 | 5,797101 | 11,56585 | 16,68225 | 17,80357 |
| 2 | 2 | 2,577578 | 4,027778 | 5,893204 | 15,89362 | 59,18739 | 104,2085 |
| 3 | 3,235294 | 4,088057 | 6,837838 | 10,82569 | 15,34899 | 42,48558 | 47,17732 |
| 4&5 | 2,5 | 3,519706 | 5,330357 | 13,72208 | 64,23045 | 142,4787 | 430,8082 |

**Nonparametric Comparisons For Each Pair Using Wilcoxon Method**

| **q*** | **Alpha** |
| --- | --- |
| 1,95996 | 0,05 |

| **Level** | **- Level** | **Score Mean Difference** | **Std Err Dif** | **Z** | **p-Value** | **Hodges-Lehmann** | **Lower CL** | **Upper CL** | **Difference Plot** |
| --- | --- | --- | --- | --- | --- | --- | --- | --- | --- |
| 4&5 | 1 | 7,933333 | 3,499755 | 2,266825 | 0,0234* | 7,058324 | 0,61111 | 20,89160 |  |
| 4&5 | 2 | 6,496739 | 3,839063 | 1,692272 | 0,0906 | 5,074744 | -0,62500 | 16,89587 |  |
| 3 | 1 | 6,133333 | 3,214550 | 1,907991 | 0,0564 | 4,014018 | -0,14706 | 9,40264 |  |
| 3 | 2 | 5,066667 | 3,688211 | 1,373747 | 0,1695 | 2,769737 | -1,79671 | 7,72618 |  |
| 2 | 1 | 3,084058 | 3,688211 | 0,836194 | 0,4030 | 1,161994 | -1,86792 | 4,74683 |  |
| 4&5 | 3 | 2,625000 | 3,500000 | 0,750000 | 0,4533 | 2,625251 | -3,92093 | 18,12392 |  |

**Oneway Analysis of original-gldm-SmallDependenceEmphasis By GrG**

**Quantiles**

| **Level** | **Minimum** | **10%** | **25%** | **Median** | **75%** | **90%** | **Maximum** |
| --- | --- | --- | --- | --- | --- | --- | --- |
| 1 | 0,429843 | 0,437325 | 0,486001 | 0,607442 | 0,665162 | 0,723781 | 0,736961 |
| 2 | 0,063013 | 0,43155 | 0,453151 | 0,514667 | 0,625 | 0,705724 | 0,744856 |
| 3 | 0,432436 | 0,4446 | 0,470807 | 0,501287 | 0,561963 | 0,61698 | 0,627525 |
| 4&5 | 0,423587 | 0,428986 | 0,470462 | 0,525905 | 0,601973 | 0,643722 | 0,663294 |

**Nonparametric Comparisons For Each Pair Using Wilcoxon Method**

| **q*** | **Alpha** |
| --- | --- |
| 1,95996 | 0,05 |

| **Level** | **- Level** | **Score Mean Difference** | **Std Err Dif** | **Z** | **p-Value** | **Hodges-Lehmann** | **Lower CL** | **Upper CL** | **Difference Plot** |
| --- | --- | --- | --- | --- | --- | --- | --- | --- | --- |
| 4&5 | 3 | 1,10833 | 3,500000 | 0,31667 | 0,7515 | 0,011409 | -0,032877 | 0,0584048 |  |
| 4&5 | 2 | -0,51413 | 3,839063 | -0,13392 | 0,8935 | -0,003135 | -0,059276 | 0,0524208 |  |
| 3 | 2 | -1,10145 | 3,688211 | -0,29864 | 0,7652 | -0,007146 | -0,073102 | 0,0466553 |  |
| 2 | 1 | -3,85507 | 3,688211 | -1,04524 | 0,2959 | -0,032850 | -0,129739 | 0,0298419 |  |
| 4&5 | 1 | -4,95833 | 3,500000 | -1,41667 | 0,1566 | -0,040918 | -0,128301 | 0,0210410 |  |
| 3 | 1 | -5,20000 | 3,214550 | -1,61764 | 0,1057 | -0,057481 | -0,147116 | 0,0220125 |  |

**Oneway Analysis of original-gldm-SmallDependenceHighGrayLevelEmphasis By GrG**

**Quantiles**

| **Level** | **Minimum** | **10%** | **25%** | **Median** | **75%** | **90%** | **Maximum** |
| --- | --- | --- | --- | --- | --- | --- | --- |
| 1 | 92,1043 | 97,22838 | 119,036 | 165,9863 | 309,2223 | 624,177 | 762,8201 |
| 2 | 0,4792 | 71,78431 | 165,1742 | 276,5495 | 449,4826 | 531,2484 | 618,6696 |
| 3 | 63,74049 | 79,12328 | 121,3447 | 170,5808 | 290,9266 | 553,232 | 562,0526 |
| 4&5 | 86,39699 | 112,0486 | 176,6017 | 247,8968 | 441,987 | 855,874 | 1097,426 |

**Nonparametric Comparisons For Each Pair Using Wilcoxon Method**

| **q*** | **Alpha** |
| --- | --- |
| 1,95996 | 0,05 |

| **Level** | **- Level** | **Score Mean Difference** | **Std Err Dif** | **Z** | **p-Value** | **Hodges-Lehmann** | **Lower CL** | **Upper CL** | **Difference Plot** |
| --- | --- | --- | --- | --- | --- | --- | --- | --- | --- |
| 4&5 | 1 | 6,12500 | 3,500000 | 1,75000 | 0,0801 | 82,3734 | -15,149 | 224,3902 |  |
| 4&5 | 3 | 5,89167 | 3,500000 | 1,68333 | 0,0923 | 78,5729 | -19,138 | 215,4082 |  |
| 2 | 1 | 4,40580 | 3,688211 | 1,19456 | 0,2323 | 79,2889 | -42,438 | 184,4452 |  |
| 4&5 | 2 | 1,07500 | 3,839063 | 0,28002 | 0,7795 | 15,4288 | -95,132 | 138,2771 |  |
| 3 | 1 | 0,00000 | 3,214550 | 0,00000 | 1,0000 | -2,1995 | -91,378 | 76,7017 |  |
| 3 | 2 | -4,62609 | 3,688211 | -1,25429 | 0,2097 | -79,1707 | -186,342 | 30,9826 |  |

**Oneway Analysis of original-gldm-DependenceNonUniformityNormalized By GrG**

**Quantiles**

| **Level** | **Minimum** | **10%** | **25%** | **Median** | **75%** | **90%** | **Maximum** |
| --- | --- | --- | --- | --- | --- | --- | --- |
| 1 | 0,284283 | 0,286146 | 0,297037 | 0,386258 | 0,450221 | 0,511037 | 0,533528 |
| 2 | 0,147125 | 0,260102 | 0,281352 | 0,327744 | 0,425781 | 0,502534 | 0,533608 |
| 3 | 0,281571 | 0,282608 | 0,287474 | 0,323529 | 0,35838 | 0,391369 | 0,416794 |
| 4&5 | 0,250873 | 0,264151 | 0,280686 | 0,323619 | 0,385446 | 0,410953 | 0,422832 |

**Nonparametric Comparisons For Each Pair Using Wilcoxon Method**

| **q*** | **Alpha** |
| --- | --- |
| 1,95996 | 0,05 |

| **Level** | **- Level** | **Score Mean Difference** | **Std Err Dif** | **Z** | **p-Value** | **Hodges-Lehmann** | **Lower CL** | **Upper CL** | **Difference Plot** |
| --- | --- | --- | --- | --- | --- | --- | --- | --- | --- |
| 4&5 | 3 | 0,17500 | 3,500000 | 0,05000 | 0,9601 | 0,001856 | -0,031194 | 0,0428221 |  |
| 4&5 | 2 | -2,29022 | 3,839063 | -0,59656 | 0,5508 | -0,013370 | -0,060560 | 0,0283517 |  |
| 3 | 2 | -2,53333 | 3,688211 | -0,68687 | 0,4922 | -0,014138 | -0,072176 | 0,0262723 |  |
| 2 | 1 | -3,85507 | 3,688211 | -1,04524 | 0,2959 | -0,024439 | -0,103189 | 0,0254630 |  |
| 3 | 1 | -5,73333 | 3,214550 | -1,78356 | 0,0745 | -0,046713 | -0,119475 | 0,0052907 |  |
| 4&5 | 1 | -6,47500 | 3,500000 | -1,85000 | 0,0643 | -0,038812 | -0,110946 | 0,0032481 |  |

**Oneway Analysis of original-gldm-LargeDependenceEmphasis By GrG**

**Quantiles**

| **Level** | **Minimum** | **10%** | **25%** | **Median** | **75%** | **90%** | **Maximum** |
| --- | --- | --- | --- | --- | --- | --- | --- |
| 1 | 2,142857 | 2,387755 | 2,852941 | 3,754717 | 5,607143 | 6,088002 | 6,300493 |
| 2 | 2,125 | 2,443601 | 3,375 | 4,411765 | 5,988 | 6,580761 | 39,46809 |
| 3 | 3,121212 | 3,523092 | 4,045045 | 5,168421 | 5,637931 | 5,910072 | 6,046512 |
| 4&5 | 3,072289 | 3,362614 | 3,818452 | 4,713095 | 5,9568 | 6,6728 | 7,104848 |

**Nonparametric Comparisons For Each Pair Using Wilcoxon Method**

| **q*** | **Alpha** |
| --- | --- |
| 1,95996 | 0,05 |

| **Level** | **- Level** | **Score Mean Difference** | **Std Err Dif** | **Z** | **p-Value** | **Hodges-Lehmann** | **Lower CL** | **Upper CL** | **Difference Plot** |
| --- | --- | --- | --- | --- | --- | --- | --- | --- | --- |
| 4&5 | 1 | 5,65833 | 3,500000 | 1,61667 | 0,1060 | 0,733961 | -0,257064 | 1,730341 |  |
| 3 | 1 | 4,13333 | 3,214550 | 1,28582 | 0,1985 | 0,622690 | -0,410162 | 1,852941 |  |
| 2 | 1 | 3,08406 | 3,688211 | 0,83619 | 0,4030 | 0,391015 | -0,688140 | 1,509142 |  |
| 4&5 | 2 | 2,85109 | 3,839063 | 0,74265 | 0,4577 | 0,297528 | -0,612836 | 1,248069 |  |
| 3 | 2 | 2,20290 | 3,688211 | 0,59728 | 0,5503 | 0,289988 | -0,624858 | 1,149210 |  |
| 4&5 | 3 | -0,05833 | 3,500000 | -0,01667 | 0,9867 | -0,002061 | -0,848739 | 0,916860 |  |

**Oneway Analysis of original-gldm-LargeDependenceLowGrayLevelEmphasis By GrG**

**Quantiles**

| **Level** | **Minimum** | **10%** | **25%** | **Median** | **75%** | **90%** | **Maximum** |
| --- | --- | --- | --- | --- | --- | --- | --- |
| 1 | 0,014239 | 0,014386 | 0,019954 | 0,053959 | 0,131663 | 0,351402 | 0,603608 |
| 2 | 0,008983 | 0,009545 | 0,013263 | 0,031201 | 0,068024 | 1,067401 | 14,01079 |
| 3 | 0,007141 | 0,012359 | 0,027379 | 0,038506 | 0,083911 | 0,120682 | 0,166498 |
| 4&5 | 0,005259 | 0,008313 | 0,010921 | 0,024144 | 0,043066 | 0,136884 | 0,243848 |

**Nonparametric Comparisons For Each Pair Using Wilcoxon Method**

| **q*** | **Alpha** |
| --- | --- |
| 1,95996 | 0,05 |

| **Level** | **- Level** | **Score Mean Difference** | **Std Err Dif** | **Z** | **p-Value** | **Hodges-Lehmann** | **Lower CL** | **Upper CL** | **Difference Plot** |
| --- | --- | --- | --- | --- | --- | --- | --- | --- | --- |
| 3 | 2 | 3,41449 | 3,688211 | 0,92579 | 0,3546 | 0,007429 | -0,014327 | 0,0295930 |  |
| 3 | 1 | -1,06667 | 3,214550 | -0,33182 | 0,7400 | -0,007342 | -0,051504 | 0,0226677 |  |
| 4&5 | 2 | -3,50543 | 3,839063 | -0,91310 | 0,3612 | -0,005672 | -0,023028 | 0,0079339 |  |
| 2 | 1 | -4,07536 | 3,688211 | -1,10497 | 0,2692 | -0,010971 | -0,048304 | 0,0109289 |  |
| 4&5 | 3 | -5,54167 | 3,500000 | -1,58333 | 0,1133 | -0,016907 | -0,041381 | 0,0026067 |  |
| 4&5 | 1 | -6,47500 | 3,500000 | -1,85000 | 0,0643 | -0,018600 | -0,056592 | 0,0025328 |  |

**Oneway Analysis of original-gldm-DependenceVariance By GrG**

**Quantiles**

| **Level** | **Minimum** | **10%** | **25%** | **Median** | **75%** | **90%** | **Maximum** |
| --- | --- | --- | --- | --- | --- | --- | --- |
| 1 | 0,273219 | 0,303526 | 0,451479 | 0,833333 | 1,276526 | 1,546706 | 1,708656 |
| 2 | 0,234375 | 0,348251 | 0,673484 | 0,822115 | 1,319543 | 1,525905 | 4,229968 |
| 3 | 0,541781 | 0,614681 | 0,830939 | 1,041108 | 1,204283 | 1,382537 | 1,518524 |
| 4&5 | 0,504573 | 0,590452 | 0,726292 | 1,098211 | 1,424431 | 1,658783 | 1,685829 |

**Nonparametric Comparisons For Each Pair Using Wilcoxon Method**

| **q*** | **Alpha** |
| --- | --- |
| 1,95996 | 0,05 |

| **Level** | **- Level** | **Score Mean Difference** | **Std Err Dif** | **Z** | **p-Value** | **Hodges-Lehmann** | **Lower CL** | **Upper CL** | **Difference Plot** |
| --- | --- | --- | --- | --- | --- | --- | --- | --- | --- |
| 4&5 | 1 | 5,075000 | 3,500000 | 1,450000 | 0,1471 | 0,2198496 | -0,105769 | 0,5351077 |  |
| 4&5 | 2 | 4,253261 | 3,839063 | 1,107890 | 0,2679 | 0,1564935 | -0,139026 | 0,4398142 |  |
| 3 | 1 | 2,933333 | 3,214550 | 0,912517 | 0,3615 | 0,1608016 | -0,170053 | 0,4377929 |  |
| 3 | 2 | 2,643478 | 3,688211 | 0,716737 | 0,4735 | 0,0915907 | -0,184609 | 0,3310390 |  |
| 4&5 | 3 | 2,275000 | 3,500000 | 0,650000 | 0,5157 | 0,0837356 | -0,180904 | 0,3632968 |  |
| 2 | 1 | 1,211594 | 3,688211 | 0,328505 | 0,7425 | 0,0595863 | -0,234353 | 0,3703791 |  |

**Oneway Analysis of original-gldm-LargeDependenceHighGrayLevelEmphasis By GrG**

**Quantiles**

| **Level** | **Minimum** | **10%** | **25%** | **Median** | **75%** | **90%** | **Maximum** |
| --- | --- | --- | --- | --- | --- | --- | --- |
| 1 | 296,0377 | 388,7068 | 725,2653 | 1243,611 | 2491,233 | 3329,105 | 3397,123 |
| 2 | 197,234 | 515,1765 | 1160,108 | 1584,214 | 3723,554 | 5656,012 | 6274,986 |
| 3 | 723,4632 | 879,258 | 1006,896 | 1422,972 | 2038,952 | 4215,767 | 5989,101 |
| 4&5 | 717,0278 | 810,5373 | 1287,09 | 2745,189 | 3698,009 | 6334,607 | 10906,75 |

**Nonparametric Comparisons For Each Pair Using Wilcoxon Method**

| **q*** | **Alpha** |
| --- | --- |
| 1,95996 | 0,05 |

| **Level** | **- Level** | **Score Mean Difference** | **Std Err Dif** | **Z** | **p-Value** | **Hodges-Lehmann** | **Lower CL** | **Upper CL** | **Difference Plot** |
| --- | --- | --- | --- | --- | --- | --- | --- | --- | --- |
| 4&5 | 1 | 8,57500 | 3,500000 | 2,45000 | 0,0143* | 970,864 | 178,60 | 2283,729 |  |
| 4&5 | 3 | 6,59167 | 3,500000 | 1,88333 | 0,0597 | 827,597 | -97,96 | 2083,492 |  |
| 2 | 1 | 5,61739 | 3,688211 | 1,52307 | 0,1277 | 565,515 | -246,77 | 1464,939 |  |
| 4&5 | 2 | 3,13152 | 3,839063 | 0,81570 | 0,4147 | 492,111 | -537,91 | 1692,130 |  |
| 3 | 1 | 2,40000 | 3,214550 | 0,74661 | 0,4553 | 258,123 | -462,45 | 912,879 |  |
| 3 | 2 | -2,42319 | 3,688211 | -0,65701 | 0,5112 | -251,622 | -1170,30 | 514,742 |  |

**Oneway Analysis of original-gldm-SmallDependenceLowGrayLevelEmphasis By GrG**

**Quantiles**

| **Level** | **Minimum** | **10%** | **25%** | **Median** | **75%** | **90%** | **Maximum** |
| --- | --- | --- | --- | --- | --- | --- | --- |
| 1 | 0,004145 | 0,004175 | 0,010273 | 0,012251 | 0,023855 | 0,039779 | 0,040641 |
| 2 | 0,001223 | 0,001443 | 0,008057 | 0,012474 | 0,026624 | 0,03758 | 0,088751 |
| 3 | 0,001675 | 0,003633 | 0,006851 | 0,011261 | 0,020269 | 0,030857 | 0,042502 |
| 4&5 | 0,000602 | 0,000932 | 0,002349 | 0,006314 | 0,016055 | 0,037228 | 0,051497 |

**Nonparametric Comparisons For Each Pair Using Wilcoxon Method**

| **q*** | **Alpha** |
| --- | --- |
| 1,95996 | 0,05 |

| **Level** | **- Level** | **Score Mean Difference** | **Std Err Dif** | **Z** | **p-Value** | **Hodges-Lehmann** | **Lower CL** | **Upper CL** | **Difference Plot** |
| --- | --- | --- | --- | --- | --- | --- | --- | --- | --- |
| 2 | 1 | -1,32174 | 3,688211 | -0,35837 | 0,7201 | -0,001849 | -0,009050 | 0,006532 |  |
| 3 | 2 | -1,43188 | 3,688211 | -0,38823 | 0,6978 | -0,001444 | -0,009470 | 0,005627 |  |
| 3 | 1 | -2,66667 | 3,214550 | -0,82956 | 0,4068 | -0,002592 | -0,010575 | 0,003736 |  |
| 4&5 | 3 | -5,30833 | 3,500000 | -1,51667 | 0,1294 | -0,003967 | -0,009411 | 0,001498 |  |
| 4&5 | 2 | -5,93587 | 3,839063 | -1,54618 | 0,1221 | -0,005865 | -0,011670 | 0,000850 |  |
| 4&5 | 1 | -7,64167 | 3,500000 | -2,18333 | 0,0290* | -0,006454 | -0,013953 | -0,000612 |  |

**Oneway Analysis of original-gldm-LowGrayLevelEmphasis By GrG**

**Quantiles**

| **Level** | **Minimum** | **10%** | **25%** | **Median** | **75%** | **90%** | **Maximum** |
| --- | --- | --- | --- | --- | --- | --- | --- |
| 1 | 0,005164 | 0,005522 | 0,01117 | 0,019301 | 0,043674 | 0,061966 | 0,079946 |
| 2 | 0,002012 | 0,002346 | 0,008747 | 0,014663 | 0,034003 | 0,1184 | 0,452866 |
| 3 | 0,002219 | 0,004617 | 0,00999 | 0,016348 | 0,02861 | 0,040217 | 0,049678 |
| 4&5 | 0,000978 | 0,002028 | 0,003955 | 0,008338 | 0,019806 | 0,051 | 0,072861 |

**Nonparametric Comparisons For Each Pair Using Wilcoxon Method**

| **q*** | **Alpha** |
| --- | --- |
| 1,95996 | 0,05 |

| **Level** | **- Level** | **Score Mean Difference** | **Std Err Dif** | **Z** | **p-Value** | **Hodges-Lehmann** | **Lower CL** | **Upper CL** | **Difference Plot** |
| --- | --- | --- | --- | --- | --- | --- | --- | --- | --- |
| 3 | 2 | -0,55072 | 3,688211 | -0,14932 | 0,8813 | -0,000138 | -0,012120 | 0,008692 |  |
| 3 | 1 | -3,06667 | 3,214550 | -0,95400 | 0,3401 | -0,005482 | -0,021197 | 0,004826 |  |
| 2 | 1 | -3,41449 | 3,688211 | -0,92579 | 0,3546 | -0,004101 | -0,016943 | 0,007717 |  |
| 4&5 | 2 | -5,28152 | 3,839063 | -1,37573 | 0,1689 | -0,006393 | -0,015227 | 0,001673 |  |
| 4&5 | 3 | -5,54167 | 3,500000 | -1,58333 | 0,1133 | -0,006052 | -0,012664 | 0,002163 |  |
| 4&5 | 1 | -7,40833 | 3,500000 | -2,11667 | 0,0343* | -0,010373 | -0,027453 | -0,000781 |  |

**Oneway Analysis of original-glcm-JointAverage By GrG**

**Quantiles**

| **Level** | **Minimum** | **10%** | **25%** | **Median** | **75%** | **90%** | **Maximum** |
| --- | --- | --- | --- | --- | --- | --- | --- |
| 1 | 8,918399 | 10,63279 | 13,23162 | 15,54547 | 21,17405 | 28,75556 | 29,99173 |
| 2 | 2,025363 | 10,89545 | 13,90874 | 19,72339 | 25,18418 | 29,92082 | 30,2646 |
| 3 | 10,8119 | 11,85099 | 13,90384 | 16,03776 | 20,59049 | 28,32246 | 31,73925 |
| 4&5 | 11,32393 | 13,36505 | 14,67957 | 20,58688 | 26,62703 | 33,31254 | 42,7696 |

**Nonparametric Comparisons For Each Pair Using Wilcoxon Method**

| **q*** | **Alpha** |
| --- | --- |
| 1,95996 | 0,05 |

| **Level** | **- Level** | **Score Mean Difference** | **Std Err Dif** | **Z** | **p-Value** | **Hodges-Lehmann** | **Lower CL** | **Upper CL** | **Difference Plot** |
| --- | --- | --- | --- | --- | --- | --- | --- | --- | --- |
| 4&5 | 1 | 5,77500 | 3,500000 | 1,65000 | 0,0989 | 3,34977 | -0,61184 | 8,655249 |  |
| 4&5 | 3 | 5,30833 | 3,500000 | 1,51667 | 0,1294 | 3,34790 | -0,79375 | 8,251866 |  |
| 2 | 1 | 4,40580 | 3,688211 | 1,19456 | 0,2323 | 2,81958 | -1,83167 | 7,548026 |  |
| 4&5 | 2 | 1,63587 | 3,839063 | 0,42611 | 0,6700 | 0,98753 | -3,67630 | 5,901200 |  |
| 3 | 1 | 0,53333 | 3,214550 | 0,16591 | 0,8682 | 0,45204 | -3,93507 | 3,807808 |  |
| 3 | 2 | -4,18551 | 3,688211 | -1,13483 | 0,2564 | -2,73515 | -7,25983 | 1,965361 |  |

**Oneway Analysis of original-glcm-SumAverage By GrG**

**Quantiles**

| **Level** | **Minimum** | **10%** | **25%** | **Median** | **75%** | **90%** | **Maximum** |
| --- | --- | --- | --- | --- | --- | --- | --- |
| 1 | 17,8368 | 21,26557 | 26,46323 | 31,09093 | 42,34809 | 57,51111 | 59,98346 |
| 2 | 4,050725 | 21,79091 | 27,81749 | 39,44677 | 50,36836 | 59,84164 | 60,52921 |
| 3 | 21,62381 | 23,70197 | 27,80768 | 32,07551 | 41,18097 | 56,64491 | 63,47849 |
| 4&5 | 22,64786 | 26,7301 | 29,35914 | 41,17376 | 53,25407 | 66,62507 | 85,53921 |

**Nonparametric Comparisons For Each Pair Using Wilcoxon Method**

| **q*** | **Alpha** |
| --- | --- |
| 1,95996 | 0,05 |

| **Level** | **- Level** | **Score Mean Difference** | **Std Err Dif** | **Z** | **p-Value** | **Hodges-Lehmann** | **Lower CL** | **Upper CL** | **Difference Plot** |
| --- | --- | --- | --- | --- | --- | --- | --- | --- | --- |
| 4&5 | 1 | 5,77500 | 3,500000 | 1,65000 | 0,0989 | 6,69954 | -1,2237 | 17,31050 |  |
| 4&5 | 3 | 5,30833 | 3,500000 | 1,51667 | 0,1294 | 6,69581 | -1,5875 | 16,50373 |  |
| 2 | 1 | 4,40580 | 3,688211 | 1,19456 | 0,2323 | 5,63915 | -3,6633 | 15,09605 |  |
| 4&5 | 2 | 1,63587 | 3,839063 | 0,42611 | 0,6700 | 1,97506 | -7,3526 | 11,80240 |  |
| 3 | 1 | 0,53333 | 3,214550 | 0,16591 | 0,8682 | 0,90408 | -7,8701 | 7,61562 |  |
| 3 | 2 | -4,18551 | 3,688211 | -1,13483 | 0,2564 | -5,47030 | -14,5197 | 3,93072 |  |

**Oneway Analysis of original-glcm-JointEntropy By GrG**

**Quantiles**

| **Level** | **Minimum** | **10%** | **25%** | **Median** | **75%** | **90%** | **Maximum** |
| --- | --- | --- | --- | --- | --- | --- | --- |
| 1 | 2,870959 | 3,831703 | 5,244744 | 6,286042 | 7,151312 | 7,971786 | 8,287549 |
| 2 | 2,245767 | 3,235791 | 5,421242 | 6,566782 | 8,019047 | 9,155877 | 9,545729 |
| 3 | 4,319126 | 5,404439 | 6,305322 | 7,668048 | 8,023858 | 8,88094 | 9,051453 |
| 4&5 | 3,783291 | 5,269031 | 6,339743 | 7,978127 | 8,988595 | 9,677728 | 10,45828 |

**Nonparametric Comparisons For Each Pair Using Wilcoxon Method**

| **q*** | **Alpha** |
| --- | --- |
| 1,95996 | 0,05 |

| **Level** | **- Level** | **Score Mean Difference** | **Std Err Dif** | **Z** | **p-Value** | **Hodges-Lehmann** | **Lower CL** | **Upper CL** | **Difference Plot** |
| --- | --- | --- | --- | --- | --- | --- | --- | --- | --- |
| 4&5 | 1 | 9,275000 | 3,500000 | 2,650000 | 0,0080* | 1,564861 | 0,535927 | 2,616062 |  |
| 3 | 1 | 7,600000 | 3,214550 | 2,364250 | 0,0181* | 1,161552 | 0,221132 | 2,112419 |  |
| 4&5 | 2 | 7,244565 | 3,839063 | 1,887066 | 0,0592 | 1,113853 | -0,033193 | 2,259543 |  |
| 3 | 2 | 4,736232 | 3,688211 | 1,284154 | 0,1991 | 0,725056 | -0,365093 | 1,827798 |  |
| 4&5 | 3 | 3,558333 | 3,500000 | 1,016667 | 0,3093 | 0,354034 | -0,746187 | 1,522376 |  |
| 2 | 1 | 2,973913 | 3,688211 | 0,806329 | 0,4201 | 0,418271 | -0,760467 | 1,716034 |  |

**Oneway Analysis of original-glcm-ClusterShade By GrG**

**Quantiles**

| **Level** | **Minimum** | **10%** | **25%** | **Median** | **75%** | **90%** | **Maximum** |
| --- | --- | --- | --- | --- | --- | --- | --- |
| 1 | -2455,52 | -1013,66 | -34,8433 | 295,7238 | 452,8007 | 898,0011 | 1222,965 |
| 2 | -276,708 | -116,408 | -45,6317 | 247,1643 | 1145,11 | 2412,784 | 4973,719 |
| 3 | -240,644 | -211,287 | 79,36372 | 244,386 | 599,6119 | 1534,475 | 1641,939 |
| 4&5 | -488,485 | -437,751 | -69,3975 | 420,2916 | 2662,822 | 7689,665 | 8848,825 |

**Nonparametric Comparisons For Each Pair Using Wilcoxon Method**

| **q*** | **Alpha** |
| --- | --- |
| 1,95996 | 0,05 |

| **Level** | **- Level** | **Score Mean Difference** | **Std Err Dif** | **Z** | **p-Value** | **Hodges-Lehmann** | **Lower CL** | **Upper CL** | **Difference Plot** |
| --- | --- | --- | --- | --- | --- | --- | --- | --- | --- |
| 4&5 | 1 | 3,441667 | 3,500000 | 0,9833333 | 0,3254 | 260,443 | -239,134 | 1805,105 |  |
| 4&5 | 3 | 2,158333 | 3,500000 | 0,6166667 | 0,5375 | 160,553 | -316,791 | 1345,792 |  |
| 4&5 | 2 | 1,822826 | 3,839063 | 0,4748101 | 0,6349 | 131,819 | -361,032 | 1103,271 |  |
| 3 | 1 | 1,600000 | 3,214550 | 0,4977368 | 0,6187 | 63,826 | -214,261 | 500,571 |  |
| 2 | 1 | 1,542029 | 3,688211 | 0,4180968 | 0,6759 | 51,501 | -215,414 | 784,780 |  |
| 3 | 2 | 0,000000 | 3,688211 | 0,0000000 | 1,0000 | -2,778 | -479,434 | 296,279 |  |

**Oneway Analysis of original-glcm-MaximumProbability By GrG**

**Quantiles**

| **Level** | **Minimum** | **10%** | **25%** | **Median** | **75%** | **90%** | **Maximum** |
| --- | --- | --- | --- | --- | --- | --- | --- |
| 1 | 0,011138 | 0,014218 | 0,021833 | 0,030846 | 0,049826 | 0,141862 | 0,227922 |
| 2 | 0,005748 | 0,007383 | 0,012492 | 0,02511 | 0,040324 | 0,179075 | 0,25873 |
| 3 | 0,00767 | 0,008435 | 0,011806 | 0,016234 | 0,026253 | 0,054838 | 0,088076 |
| 4&5 | 0,003615 | 0,00488 | 0,0074 | 0,012776 | 0,029595 | 0,051069 | 0,101805 |

**Nonparametric Comparisons For Each Pair Using Wilcoxon Method**

| **q*** | **Alpha** |
| --- | --- |
| 1,95996 | 0,05 |

| **Level** | **- Level** | **Score Mean Difference** | **Std Err Dif** | **Z** | **p-Value** | **Hodges-Lehmann** | **Lower CL** | **Upper CL** | **Difference Plot** |
| --- | --- | --- | --- | --- | --- | --- | --- | --- | --- |
| 4&5 | 3 | -3,09167 | 3,500000 | -0,88333 | 0,3771 | -0,002726 | -0,008991 | 0,005052 |  |
| 2 | 1 | -3,96522 | 3,688211 | -1,07511 | 0,2823 | -0,005859 | -0,018636 | 0,008230 |  |
| 3 | 2 | -4,62609 | 3,688211 | -1,25429 | 0,2097 | -0,006005 | -0,019148 | 0,003198 |  |
| 4&5 | 2 | -7,24457 | 3,839063 | -1,88707 | 0,0592 | -0,008031 | -0,020311 | 0,000516 |  |
| 3 | 1 | -8,00000 | 3,214550 | -2,48868 | 0,0128* | -0,012596 | -0,024425 | -0,002784 |  |
| 4&5 | 1 | -9,50833 | 3,500000 | -2,71667 | 0,0066* | -0,015722 | -0,025862 | -0,004617 |  |

**Oneway Analysis of original-glcm-Idmn By GrG**

**Quantiles**

| **Level** | **Minimum** | **10%** | **25%** | **Median** | **75%** | **90%** | **Maximum** |
| --- | --- | --- | --- | --- | --- | --- | --- |
| 1 | 0,830264 | 0,838287 | 0,922002 | 0,932812 | 0,961218 | 0,974336 | 0,980382 |
| 2 | 0,828559 | 0,902423 | 0,938004 | 0,952161 | 0,978214 | 0,983851 | 0,985385 |
| 3 | 0,921731 | 0,928876 | 0,94025 | 0,958026 | 0,967272 | 0,976695 | 0,981635 |
| 4&5 | 0,901195 | 0,907314 | 0,943064 | 0,963227 | 0,979573 | 0,985826 | 0,988362 |

**Nonparametric Comparisons For Each Pair Using Wilcoxon Method**

| **q*** | **Alpha** |
| --- | --- |
| 1,95996 | 0,05 |

| **Level** | **- Level** | **Score Mean Difference** | **Std Err Dif** | **Z** | **p-Value** | **Hodges-Lehmann** | **Lower CL** | **Upper CL** | **Difference Plot** |
| --- | --- | --- | --- | --- | --- | --- | --- | --- | --- |
| 4&5 | 1 | 6,94167 | 3,500000 | 1,98333 | 0,0473* | 0,019818 | -0,001009 | 0,0428499 |  |
| 3 | 1 | 4,66667 | 3,214550 | 1,45173 | 0,1466 | 0,012137 | -0,004611 | 0,0360238 |  |
| 2 | 1 | 4,18551 | 3,688211 | 1,13483 | 0,2564 | 0,015080 | -0,008879 | 0,0345999 |  |
| 4&5 | 2 | 3,59891 | 3,839063 | 0,93745 | 0,3485 | 0,006391 | -0,007510 | 0,0222996 |  |
| 4&5 | 3 | 3,09167 | 3,500000 | 0,88333 | 0,3771 | 0,006861 | -0,008089 | 0,0213533 |  |
| 3 | 2 | -0,22029 | 3,688211 | -0,05973 | 0,9524 | -0,000531 | -0,016273 | 0,0164445 |  |

**Oneway Analysis of original-glcm-JointEnergy By GrG**

**Quantiles**

| **Level** | **Minimum** | **10%** | **25%** | **Median** | **75%** | **90%** | **Maximum** |
| --- | --- | --- | --- | --- | --- | --- | --- |
| 1 | 0,003662 | 0,004756 | 0,008169 | 0,014064 | 0,028908 | 0,118275 | 0,206056 |
| 2 | 0,001829 | 0,002224 | 0,004405 | 0,011423 | 0,025054 | 0,134217 | 0,237168 |
| 3 | 0,002401 | 0,00272 | 0,004325 | 0,005819 | 0,013607 | 0,031986 | 0,055866 |
| 4&5 | 0,001129 | 0,001652 | 0,002562 | 0,00447 | 0,013194 | 0,028386 | 0,07976 |

**Nonparametric Comparisons For Each Pair Using Wilcoxon Method**

| **q*** | **Alpha** |
| --- | --- |
| 1,95996 | 0,05 |

| **Level** | **- Level** | **Score Mean Difference** | **Std Err Dif** | **Z** | **p-Value** | **Hodges-Lehmann** | **Lower CL** | **Upper CL** | **Difference Plot** |
| --- | --- | --- | --- | --- | --- | --- | --- | --- | --- |
| 2 | 1 | -3,19420 | 3,688211 | -0,86606 | 0,3865 | -0,002866 | -0,010737 | 0,005629 |  |
| 4&5 | 3 | -3,44167 | 3,500000 | -0,98333 | 0,3254 | -0,001178 | -0,003670 | 0,002521 |  |
| 3 | 2 | -4,62609 | 3,688211 | -1,25429 | 0,2097 | -0,003830 | -0,011564 | 0,001251 |  |
| 4&5 | 2 | -7,33804 | 3,839063 | -1,91142 | 0,0560 | -0,004360 | -0,011466 | 0,000154 |  |
| 3 | 1 | -7,73333 | 3,214550 | -2,40573 | 0,0161* | -0,006429 | -0,015771 | -0,001159 |  |
| 4&5 | 1 | -9,27500 | 3,500000 | -2,65000 | 0,0080* | -0,007190 | -0,016356 | -0,002023 |  |

**Oneway Analysis of original-glcm-Contrast By GrG**

**Quantiles**

| **Level** | **Minimum** | **10%** | **25%** | **Median** | **75%** | **90%** | **Maximum** |
| --- | --- | --- | --- | --- | --- | --- | --- |
| 1 | 42,39294 | 43,20306 | 50,23806 | 61,74931 | 142,2085 | 210,697 | 215,688 |
| 2 | 0,871287 | 29,03682 | 57,13946 | 76,44745 | 99,11218 | 139,0037 | 201,974 |
| 3 | 37,57956 | 38,12338 | 47,6985 | 65,78068 | 96,60433 | 138,3431 | 142,7395 |
| 4&5 | 42,49571 | 50,91187 | 52,40836 | 71,30743 | 112,4208 | 144,9611 | 232,2285 |

**Nonparametric Comparisons For Each Pair Using Wilcoxon Method**

| **q*** | **Alpha** |
| --- | --- |
| 1,95996 | 0,05 |

| **Level** | **- Level** | **Score Mean Difference** | **Std Err Dif** | **Z** | **p-Value** | **Hodges-Lehmann** | **Lower CL** | **Upper CL** | **Difference Plot** |
| --- | --- | --- | --- | --- | --- | --- | --- | --- | --- |
| 4&5 | 3 | 3,32500 | 3,500000 | 0,950000 | 0,3421 | 7,47865 | -14,0564 | 31,52649 |  |
| 4&5 | 1 | 0,17500 | 3,500000 | 0,050000 | 0,9601 | 0,27099 | -42,5944 | 20,18253 |  |
| 4&5 | 2 | -0,32717 | 3,839063 | -0,085222 | 0,9321 | -0,54311 | -20,1903 | 25,02734 |  |
| 2 | 1 | -0,55072 | 3,688211 | -0,149320 | 0,8813 | -1,16483 | -49,3911 | 21,78710 |  |
| 3 | 2 | -2,64348 | 3,688211 | -0,716737 | 0,4735 | -6,77909 | -30,0526 | 16,67026 |  |
| 3 | 1 | -2,66667 | 3,214550 | -0,829561 | 0,4068 | -7,24924 | -61,5755 | 16,35821 |  |

**Oneway Analysis of original-glcm-DifferenceEntropy By GrG**

**Quantiles**

| **Level** | **Minimum** | **10%** | **25%** | **Median** | **75%** | **90%** | **Maximum** |
| --- | --- | --- | --- | --- | --- | --- | --- |
| 1 | 1,657997 | 2,458864 | 3,243098 | 3,630652 | 3,888919 | 4,199051 | 4,307989 |
| 2 | 1,179581 | 1,615783 | 3,55088 | 3,918498 | 4,020277 | 4,179893 | 4,314705 |
| 3 | 2,743771 | 3,172629 | 3,654926 | 3,8376 | 4,21046 | 4,450108 | 4,523562 |
| 4&5 | 2,501643 | 3,456061 | 3,664262 | 3,944533 | 4,257183 | 4,466647 | 4,719208 |

**Nonparametric Comparisons For Each Pair Using Wilcoxon Method**

| **q*** | **Alpha** |
| --- | --- |
| 1,95996 | 0,05 |

| **Level** | **- Level** | **Score Mean Difference** | **Std Err Dif** | **Z** | **p-Value** | **Hodges-Lehmann** | **Lower CL** | **Upper CL** | **Difference Plot** |
| --- | --- | --- | --- | --- | --- | --- | --- | --- | --- |
| 4&5 | 1 | 7,641667 | 3,500000 | 2,183333 | 0,0290* | 0,3368379 | 0,034116 | 0,6713776 |  |
| 3 | 1 | 5,733333 | 3,214550 | 1,783557 | 0,0745 | 0,2747134 | -0,046173 | 0,6138627 |  |
| 4&5 | 2 | 5,094565 | 3,839063 | 1,327033 | 0,1845 | 0,1745059 | -0,081091 | 0,4681074 |  |
| 2 | 1 | 4,405797 | 3,688211 | 1,194562 | 0,2323 | 0,1652619 | -0,149908 | 0,4148858 |  |
| 3 | 2 | 3,524638 | 3,688211 | 0,955650 | 0,3392 | 0,1003134 | -0,155967 | 0,4469773 |  |
| 4&5 | 3 | 1,341667 | 3,500000 | 0,383333 | 0,7015 | 0,0573046 | -0,242127 | 0,3512959 |  |

**Oneway Analysis of original-glcm-InverseVariance By GrG**

**Quantiles**

| **Level** | **Minimum** | **10%** | **25%** | **Median** | **75%** | **90%** | **Maximum** |
| --- | --- | --- | --- | --- | --- | --- | --- |
| 1 | 0,090816 | 0,097017 | 0,11764 | 0,157492 | 0,186268 | 0,199477 | 0,205455 |
| 2 | 0,086917 | 0,119144 | 0,136664 | 0,158419 | 0,184154 | 0,233741 | 0,528035 |
| 3 | 0,118181 | 0,121546 | 0,13359 | 0,157672 | 0,189696 | 0,194814 | 0,195496 |
| 4&5 | 0,120134 | 0,126489 | 0,139608 | 0,15494 | 0,177201 | 0,186333 | 0,191452 |

**Nonparametric Comparisons For Each Pair Using Wilcoxon Method**

| **q*** | **Alpha** |
| --- | --- |
| 1,95996 | 0,05 |

| **Level** | **- Level** | **Score Mean Difference** | **Std Err Dif** | **Z** | **p-Value** | **Hodges-Lehmann** | **Lower CL** | **Upper CL** | **Difference Plot** |
| --- | --- | --- | --- | --- | --- | --- | --- | --- | --- |
| 2 | 1 | 2,42319 | 3,688211 | 0,657009 | 0,5112 | 0,008413 | -0,018251 | 0,0390578 |  |
| 3 | 1 | 2,00000 | 3,214550 | 0,622171 | 0,5338 | 0,008289 | -0,018293 | 0,0366978 |  |
| 4&5 | 1 | 0,75833 | 3,500000 | 0,216667 | 0,8285 | 0,003275 | -0,021618 | 0,0308110 |  |
| 3 | 2 | -0,55072 | 3,688211 | -0,149320 | 0,8813 | -0,000794 | -0,024083 | 0,0198038 |  |
| 4&5 | 3 | -1,10833 | 3,500000 | -0,316667 | 0,7515 | -0,002997 | -0,022543 | 0,0155952 |  |
| 4&5 | 2 | -2,19674 | 3,839063 | -0,572207 | 0,5672 | -0,004643 | -0,022049 | 0,0134017 |  |

**Oneway Analysis of original-glcm-DifferenceVariance By GrG**

**Quantiles**

| **Level** | **Minimum** | **10%** | **25%** | **Median** | **75%** | **90%** | **Maximum** |
| --- | --- | --- | --- | --- | --- | --- | --- |
| 1 | 16,39635 | 17,02555 | 20,61467 | 23,15029 | 44,44584 | 69,11821 | 90,11708 |
| 2 | 0,335983 | 9,445313 | 19,3318 | 29,91821 | 37,31601 | 51,13364 | 60,70977 |
| 3 | 13,88514 | 13,97679 | 17,78515 | 25,97503 | 33,66821 | 53,54201 | 58,20929 |
| 4&5 | 15,82018 | 16,59563 | 20,45238 | 27,88705 | 46,26405 | 58,31594 | 104,0875 |

**Nonparametric Comparisons For Each Pair Using Wilcoxon Method**

| **q*** | **Alpha** |
| --- | --- |
| 1,95996 | 0,05 |

| **Level** | **- Level** | **Score Mean Difference** | **Std Err Dif** | **Z** | **p-Value** | **Hodges-Lehmann** | **Lower CL** | **Upper CL** | **Difference Plot** |
| --- | --- | --- | --- | --- | --- | --- | --- | --- | --- |
| 4&5 | 3 | 3,55833 | 3,500000 | 1,01667 | 0,3093 | 3,62015 | -5,2267 | 14,63825 |  |
| 4&5 | 2 | 1,54239 | 3,839063 | 0,40176 | 0,6879 | 2,05299 | -6,7518 | 12,95697 |  |
| 4&5 | 1 | 1,45833 | 3,500000 | 0,41667 | 0,6769 | 2,03453 | -5,8209 | 12,30860 |  |
| 2 | 1 | 0,55072 | 3,688211 | 0,14932 | 0,8813 | 0,77009 | -11,7965 | 8,99535 |  |
| 3 | 1 | -0,80000 | 3,214550 | -0,24887 | 0,8035 | -2,35846 | -11,6166 | 7,40435 |  |
| 3 | 2 | -1,98261 | 3,688211 | -0,53755 | 0,5909 | -2,39245 | -11,2409 | 6,98383 |  |

**Oneway Analysis of original-glcm-Idn By GrG**

**Quantiles**

| **Level** | **Minimum** | **10%** | **25%** | **Median** | **75%** | **90%** | **Maximum** |
| --- | --- | --- | --- | --- | --- | --- | --- |
| 1 | 0,740211 | 0,743674 | 0,821345 | 0,841824 | 0,869891 | 0,892801 | 0,90487 |
| 2 | 0,739904 | 0,802793 | 0,837091 | 0,864869 | 0,901421 | 0,914622 | 0,921439 |
| 3 | 0,824974 | 0,828418 | 0,844477 | 0,862986 | 0,879606 | 0,899682 | 0,907317 |
| 4&5 | 0,791767 | 0,810432 | 0,849713 | 0,875535 | 0,903378 | 0,920471 | 0,930662 |

**Nonparametric Comparisons For Each Pair Using Wilcoxon Method**

| **q*** | **Alpha** |
| --- | --- |
| 1,95996 | 0,05 |

| **Level** | **- Level** | **Score Mean Difference** | **Std Err Dif** | **Z** | **p-Value** | **Hodges-Lehmann** | **Lower CL** | **Upper CL** | **Difference Plot** |
| --- | --- | --- | --- | --- | --- | --- | --- | --- | --- |
| 4&5 | 1 | 6,59167 | 3,500000 | 1,88333 | 0,0597 | 0,030221 | -0,001205 | 0,0585289 |  |
| 4&5 | 3 | 4,02500 | 3,500000 | 1,15000 | 0,2501 | 0,013264 | -0,013766 | 0,0359962 |  |
| 3 | 1 | 4,00000 | 3,214550 | 1,24434 | 0,2134 | 0,015154 | -0,009417 | 0,0446195 |  |
| 2 | 1 | 3,96522 | 3,688211 | 1,07511 | 0,2823 | 0,019997 | -0,012211 | 0,0467718 |  |
| 4&5 | 2 | 3,78587 | 3,839063 | 0,98614 | 0,3241 | 0,010921 | -0,014297 | 0,0375388 |  |
| 3 | 2 | -0,22029 | 3,688211 | -0,05973 | 0,9524 | -0,001240 | -0,024268 | 0,0246226 |  |

**Oneway Analysis of original-glcm-Idm By GrG**

**Quantiles**

| **Level** | **Minimum** | **10%** | **25%** | **Median** | **75%** | **90%** | **Maximum** |
| --- | --- | --- | --- | --- | --- | --- | --- |
| 1 | 0,092392 | 0,110258 | 0,124333 | 0,15331 | 0,193564 | 0,198584 | 0,20056 |
| 2 | 0,079047 | 0,116243 | 0,146652 | 0,159734 | 0,185865 | 0,223931 | 0,674197 |
| 3 | 0,117649 | 0,121476 | 0,134679 | 0,156707 | 0,182583 | 0,203392 | 0,214419 |
| 4&5 | 0,114081 | 0,127159 | 0,145435 | 0,153969 | 0,171902 | 0,185482 | 0,20835 |

**Nonparametric Comparisons For Each Pair Using Wilcoxon Method**

| **q*** | **Alpha** |
| --- | --- |
| 1,95996 | 0,05 |

| **Level** | **- Level** | **Score Mean Difference** | **Std Err Dif** | **Z** | **p-Value** | **Hodges-Lehmann** | **Lower CL** | **Upper CL** | **Difference Plot** |
| --- | --- | --- | --- | --- | --- | --- | --- | --- | --- |
| 2 | 1 | 1,21159 | 3,688211 | 0,328505 | 0,7425 | 0,006107 | -0,017039 | 0,0324287 |  |
| 3 | 1 | 0,53333 | 3,214550 | 0,165912 | 0,8682 | 0,001859 | -0,019403 | 0,0316355 |  |
| 4&5 | 1 | -0,05833 | 3,500000 | -0,016667 | 0,9867 | -0,000272 | -0,025493 | 0,0235778 |  |
| 3 | 2 | -1,43188 | 3,688211 | -0,388233 | 0,6978 | -0,004036 | -0,026813 | 0,0216915 |  |
| 4&5 | 3 | -1,69167 | 3,500000 | -0,483333 | 0,6289 | -0,004229 | -0,025732 | 0,0164112 |  |
| 4&5 | 2 | -3,59891 | 3,839063 | -0,937446 | 0,3485 | -0,005764 | -0,022717 | 0,0082501 |  |

**Oneway Analysis of original-glcm-Correlation By GrG**

**Quantiles**

| **Level** | **Minimum** | **10%** | **25%** | **Median** | **75%** | **90%** | **Maximum** |
| --- | --- | --- | --- | --- | --- | --- | --- |
| 1 | -0,3856 | -0,25208 | 0,065359 | 0,189735 | 0,234238 | 0,43351 | 0,475314 |
| 2 | -0,17588 | -0,03443 | 0,106306 | 0,282147 | 0,456173 | 0,53683 | 0,565814 |
| 3 | -0,07444 | -0,019 | 0,16558 | 0,24608 | 0,318701 | 0,346674 | 0,358479 |
| 4&5 | 0,034558 | 0,042553 | 0,163439 | 0,333731 | 0,439784 | 0,529944 | 0,578163 |

**Nonparametric Comparisons For Each Pair Using Wilcoxon Method**

| **q*** | **Alpha** |
| --- | --- |
| 1,95996 | 0,05 |

| **Level** | **- Level** | **Score Mean Difference** | **Std Err Dif** | **Z** | **p-Value** | **Hodges-Lehmann** | **Lower CL** | **Upper CL** | **Difference Plot** |
| --- | --- | --- | --- | --- | --- | --- | --- | --- | --- |
| 4&5 | 1 | 7,52500 | 3,500000 | 2,15000 | 0,0316* | 0,159393 | 0,015701 | 0,2918682 |  |
| 4&5 | 3 | 6,70833 | 3,500000 | 1,91667 | 0,0553 | 0,101130 | -0,004809 | 0,2064420 |  |
| 2 | 1 | 4,73623 | 3,688211 | 1,28415 | 0,1991 | 0,105120 | -0,044614 | 0,2765217 |  |
| 3 | 1 | 3,86667 | 3,214550 | 1,20286 | 0,2290 | 0,059930 | -0,068659 | 0,1620351 |  |
| 4&5 | 2 | 2,19674 | 3,839063 | 0,57221 | 0,5672 | 0,045189 | -0,087088 | 0,1790256 |  |
| 3 | 2 | -2,53333 | 3,688211 | -0,68687 | 0,4922 | -0,065348 | -0,195731 | 0,0910218 |  |

**Oneway Analysis of original-glcm-Autocorrelation By GrG**

**Quantiles**

| **Level** | **Minimum** | **10%** | **25%** | **Median** | **75%** | **90%** | **Maximum** |
| --- | --- | --- | --- | --- | --- | --- | --- |
| 1 | 84,12808 | 120,1204 | 186,6235 | 230,6776 | 474,4853 | 843,3475 | 930,5767 |
| 2 | 4,269287 | 117,9136 | 210,876 | 451,7566 | 655,1374 | 928,9448 | 951,8811 |
| 3 | 121,1122 | 143,5115 | 193,8087 | 259,8119 | 442,8191 | 825,917 | 1023,082 |
| 4&5 | 129,1099 | 187,8707 | 232,763 | 459,5255 | 724,6566 | 1156,826 | 1915,536 |

**Nonparametric Comparisons For Each Pair Using Wilcoxon Method**

| **q*** | **Alpha** |
| --- | --- |
| 1,95996 | 0,05 |

| **Level** | **- Level** | **Score Mean Difference** | **Std Err Dif** | **Z** | **p-Value** | **Hodges-Lehmann** | **Lower CL** | **Upper CL** | **Difference Plot** |
| --- | --- | --- | --- | --- | --- | --- | --- | --- | --- |
| 4&5 | 1 | 6,35833 | 3,500000 | 1,81667 | 0,0693 | 126,166 | -15,005 | 350,8096 |  |
| 4&5 | 3 | 5,42500 | 3,500000 | 1,55000 | 0,1211 | 122,330 | -24,763 | 333,4372 |  |
| 2 | 1 | 4,62609 | 3,688211 | 1,25429 | 0,2097 | 107,599 | -58,317 | 287,2487 |  |
| 4&5 | 2 | 1,72935 | 3,839063 | 0,45046 | 0,6524 | 33,174 | -151,014 | 239,6736 |  |
| 3 | 1 | 0,93333 | 3,214550 | 0,29035 | 0,7716 | 16,936 | -121,236 | 118,7009 |  |
| 3 | 2 | -4,51594 | 3,688211 | -1,22443 | 0,2208 | -96,254 | -272,492 | 61,3621 |  |

**Oneway Analysis of original-glcm-SumEntropy By GrG**

**Quantiles**

| **Level** | **Minimum** | **10%** | **25%** | **Median** | **75%** | **90%** | **Maximum** |
| --- | --- | --- | --- | --- | --- | --- | --- |
| 1 | 1,855226 | 2,690995 | 3,866431 | 4,599127 | 4,860864 | 5,292016 | 5,571419 |
| 2 | 1,241601 | 2,322719 | 4,17202 | 4,826377 | 5,347535 | 5,775041 | 5,816552 |
| 3 | 3,099641 | 3,794909 | 4,484801 | 5,045946 | 5,434744 | 5,576033 | 5,690574 |
| 4&5 | 2,71124 | 3,932649 | 4,595883 | 5,131694 | 5,773754 | 6,054478 | 6,321469 |

**Nonparametric Comparisons For Each Pair Using Wilcoxon Method**

| **q*** | **Alpha** |
| --- | --- |
| 1,95996 | 0,05 |

| **Level** | **- Level** | **Score Mean Difference** | **Std Err Dif** | **Z** | **p-Value** | **Hodges-Lehmann** | **Lower CL** | **Upper CL** | **Difference Plot** |
| --- | --- | --- | --- | --- | --- | --- | --- | --- | --- |
| 4&5 | 1 | 8,925000 | 3,500000 | 2,550000 | 0,0108* | 0,7644884 | 0,162337 | 1,274898 |  |
| 3 | 1 | 6,933333 | 3,214550 | 2,156860 | 0,0310* | 0,5498438 | 0,046744 | 1,010247 |  |
| 4&5 | 2 | 6,496739 | 3,839063 | 1,692272 | 0,0906 | 0,4499280 | -0,093084 | 1,025963 |  |
| 2 | 1 | 3,744928 | 3,688211 | 1,015378 | 0,3099 | 0,2747573 | -0,303579 | 0,901364 |  |
| 4&5 | 3 | 3,558333 | 3,500000 | 1,016667 | 0,3093 | 0,2072503 | -0,331038 | 0,699849 |  |
| 3 | 2 | 2,863768 | 3,688211 | 0,776465 | 0,4375 | 0,1830795 | -0,292002 | 0,804668 |  |

**Oneway Analysis of original-glcm-SumSquares By GrG**

**Quantiles**

| **Level** | **Minimum** | **10%** | **25%** | **Median** | **75%** | **90%** | **Maximum** |
| --- | --- | --- | --- | --- | --- | --- | --- |
| 1 | 26,90888 | 27,23225 | 33,89716 | 42,44853 | 76,39363 | 110,0679 | 133,8859 |
| 2 | 0,594845 | 17,60005 | 39,95529 | 55,34653 | 70,25984 | 121,9736 | 141,4891 |
| 3 | 20,76083 | 22,3038 | 29,07934 | 48,5816 | 61,5499 | 86,91121 | 87,45765 |
| 4&5 | 26,27716 | 26,76765 | 37,76305 | 54,32814 | 106,1977 | 144,8534 | 179,9478 |

**Nonparametric Comparisons For Each Pair Using Wilcoxon Method**

| **q*** | **Alpha** |
| --- | --- |
| 1,95996 | 0,05 |

| **Level** | **- Level** | **Score Mean Difference** | **Std Err Dif** | **Z** | **p-Value** | **Hodges-Lehmann** | **Lower CL** | **Upper CL** | **Difference Plot** |
| --- | --- | --- | --- | --- | --- | --- | --- | --- | --- |
| 4&5 | 3 | 4,84167 | 3,500000 | 1,38333 | 0,1666 | 12,3721 | -6,9028 | 38,76622 |  |
| 4&5 | 1 | 3,09167 | 3,500000 | 0,88333 | 0,3771 | 7,2864 | -9,3367 | 33,21858 |  |
| 2 | 1 | 2,42319 | 3,688211 | 0,65701 | 0,5112 | 5,5243 | -14,9286 | 23,34597 |  |
| 4&5 | 2 | 0,98152 | 3,839063 | 0,25567 | 0,7982 | 1,4863 | -14,2408 | 28,60475 |  |
| 3 | 1 | -2,13333 | 3,214550 | -0,66365 | 0,5069 | -3,5764 | -24,3496 | 14,49387 |  |
| 3 | 2 | -4,07536 | 3,688211 | -1,10497 | 0,2692 | -9,7473 | -27,1746 | 8,63486 |  |

**Oneway Analysis of original-glcm-ClusterProminence By GrG**

**Quantiles**

| **Level** | **Minimum** | **10%** | **25%** | **Median** | **75%** | **90%** | **Maximum** |
| --- | --- | --- | --- | --- | --- | --- | --- |
| 1 | 6063,032 | 10157,34 | 14141,68 | 34456,48 | 75366,82 | 155884,2 | 249281,2 |
| 2 | 5,141301 | 3067,117 | 21967,25 | 44975,15 | 144656 | 291167,4 | 551451,9 |
| 3 | 6306,547 | 9223,321 | 13155,09 | 43254,25 | 87968,34 | 138838,4 | 165734,9 |
| 4&5 | 7300,586 | 10053,44 | 27048,43 | 45713,39 | 267344,7 | 725253,3 | 864685,9 |

**Nonparametric Comparisons For Each Pair Using Wilcoxon Method**

| **q*** | **Alpha** |
| --- | --- |
| 1,95996 | 0,05 |

| **Level** | **- Level** | **Score Mean Difference** | **Std Err Dif** | **Z** | **p-Value** | **Hodges-Lehmann** | **Lower CL** | **Upper CL** | **Difference Plot** |
| --- | --- | --- | --- | --- | --- | --- | --- | --- | --- |
| 4&5 | 1 | 5,19167 | 3,500000 | 1,48333 | 0,1380 | 21314,1 | -6597,7 | 149473,8 |  |
| 2 | 1 | 4,62609 | 3,688211 | 1,25429 | 0,2097 | 15478,8 | -13900,1 | 73263,9 |  |
| 4&5 | 3 | 4,02500 | 3,500000 | 1,15000 | 0,2501 | 21139,6 | -14799,9 | 153902,1 |  |
| 4&5 | 2 | 2,29022 | 3,839063 | 0,59656 | 0,5508 | 8878,9 | -28299,4 | 80943,4 |  |
| 3 | 1 | 0,53333 | 3,214550 | 0,16591 | 0,8682 | 2350,1 | -24490,2 | 41673,0 |  |
| 3 | 2 | -2,75362 | 3,688211 | -0,74660 | 0,4553 | -15210,6 | -64466,4 | 16879,1 |  |

**Oneway Analysis of original-glcm-Imc2 By GrG**

**Quantiles**

| **Level** | **Minimum** | **10%** | **25%** | **Median** | **75%** | **90%** | **Maximum** |
| --- | --- | --- | --- | --- | --- | --- | --- |
| 1 | 0,943215 | 0,949877 | 0,972442 | 0,993384 | 0,998566 | 0,998812 | 0,99897 |
| 2 | 0,675327 | 0,813433 | 0,956094 | 0,992041 | 0,997287 | 0,998455 | 0,999087 |
| 3 | 0,856252 | 0,874344 | 0,955059 | 0,987237 | 0,992231 | 0,995623 | 0,99748 |
| 4&5 | 0,695685 | 0,770606 | 0,851637 | 0,981227 | 0,996179 | 0,997959 | 0,998683 |

**Nonparametric Comparisons For Each Pair Using Wilcoxon Method**

| **q*** | **Alpha** |
| --- | --- |
| 1,95996 | 0,05 |

| **Level** | **- Level** | **Score Mean Difference** | **Std Err Dif** | **Z** | **p-Value** | **Hodges-Lehmann** | **Lower CL** | **Upper CL** | **Difference Plot** |
| --- | --- | --- | --- | --- | --- | --- | --- | --- | --- |
| 4&5 | 3 | -0,17500 | 3,500000 | -0,05000 | 0,9601 | -0,000639 | -0,072738 | 0,0095912 |  |
| 2 | 1 | -2,86377 | 3,688211 | -0,77647 | 0,4375 | -0,001369 | -0,018454 | 0,0046897 |  |
| 4&5 | 2 | -3,69239 | 3,839063 | -0,96179 | 0,3362 | -0,003040 | -0,060460 | 0,0042519 |  |
| 3 | 2 | -3,85507 | 3,688211 | -1,04524 | 0,2959 | -0,004357 | -0,019056 | 0,0099879 |  |
| 4&5 | 1 | -5,42500 | 3,500000 | -1,55000 | 0,1211 | -0,010530 | -0,097073 | 0,0018821 |  |
| 3 | 1 | -5,60000 | 3,214550 | -1,74208 | 0,0815 | -0,006475 | -0,026219 | 0,0025606 |  |

**Oneway Analysis of original-glcm-Imc1 By GrG**

**Quantiles**

| **Level** | **Minimum** | **10%** | **25%** | **Median** | **75%** | **90%** | **Maximum** |
| --- | --- | --- | --- | --- | --- | --- | --- |
| 1 | -0,83747 | -0,83614 | -0,69102 | -0,59903 | -0,3416 | -0,26905 | -0,2504 |
| 2 | -0,80317 | -0,73926 | -0,64388 | -0,51103 | -0,26374 | -0,13388 | -0,10946 |
| 3 | -0,68137 | -0,63683 | -0,48336 | -0,38276 | -0,27521 | -0,15166 | -0,13643 |
| 4&5 | -0,85465 | -0,69994 | -0,52059 | -0,35834 | -0,13602 | -0,09404 | -0,07315 |

**Nonparametric Comparisons For Each Pair Using Wilcoxon Method**

| **q*** | **Alpha** |
| --- | --- |
| 1,95996 | 0,05 |

| **Level** | **- Level** | **Score Mean Difference** | **Std Err Dif** | **Z** | **p-Value** | **Hodges-Lehmann** | **Lower CL** | **Upper CL** | **Difference Plot** |
| --- | --- | --- | --- | --- | --- | --- | --- | --- | --- |
| 4&5 | 1 | 7,875000 | 3,500000 | 2,250000 | 0,0244* | 0,1839291 | 0,017859 | 0,3685858 |  |
| 3 | 1 | 6,800000 | 3,214550 | 2,115381 | 0,0344* | 0,1701791 | 0,008952 | 0,3270192 |  |
| 4&5 | 2 | 6,590217 | 3,839063 | 1,716621 | 0,0860 | 0,1129813 | -0,018733 | 0,2737936 |  |
| 3 | 2 | 5,286957 | 3,688211 | 1,433475 | 0,1517 | 0,1079176 | -0,046367 | 0,2491823 |  |
| 2 | 1 | 3,744928 | 3,688211 | 1,015378 | 0,3099 | 0,0776045 | -0,091306 | 0,2106599 |  |
| 4&5 | 3 | 1,341667 | 3,500000 | 0,383333 | 0,7015 | 0,0308459 | -0,137317 | 0,1818035 |  |

**Oneway Analysis of original-glcm-DifferenceAverage By GrG**

**Quantiles**

| **Level** | **Minimum** | **10%** | **25%** | **Median** | **75%** | **90%** | **Maximum** |
| --- | --- | --- | --- | --- | --- | --- | --- |
| 1 | 5,063421 | 5,075698 | 5,36897 | 6,294512 | 9,286386 | 11,20248 | 12,12089 |
| 2 | 0,688219 | 4,110587 | 5,809638 | 6,689605 | 7,720163 | 9,057416 | 11,51059 |
| 3 | 4,836442 | 4,842281 | 5,425748 | 6,299376 | 7,567884 | 9,094302 | 9,157399 |
| 4&5 | 5,078854 | 5,485687 | 5,826544 | 6,426963 | 7,837113 | 9,225524 | 11,00236 |

**Nonparametric Comparisons For Each Pair Using Wilcoxon Method**

| **q*** | **Alpha** |
| --- | --- |
| 1,95996 | 0,05 |

| **Level** | **- Level** | **Score Mean Difference** | **Std Err Dif** | **Z** | **p-Value** | **Hodges-Lehmann** | **Lower CL** | **Upper CL** | **Difference Plot** |
| --- | --- | --- | --- | --- | --- | --- | --- | --- | --- |
| 4&5 | 3 | 3,79167 | 3,500000 | 1,08333 | 0,2787 | 0,353618 | -0,77085 | 1,290906 |  |
| 4&5 | 2 | 0,00000 | 3,839063 | 0,00000 | 1,0000 | -0,004199 | -0,89409 | 0,907016 |  |
| 4&5 | 1 | -0,05833 | 3,500000 | -0,01667 | 0,9867 | -0,006676 | -1,79010 | 0,890220 |  |
| 2 | 1 | -0,33043 | 3,688211 | -0,08959 | 0,9286 | -0,138020 | -2,12523 | 1,147598 |  |
| 3 | 2 | -2,53333 | 3,688211 | -0,68687 | 0,4922 | -0,352218 | -1,45215 | 0,717590 |  |
| 3 | 1 | -2,66667 | 3,214550 | -0,82956 | 0,4068 | -0,405278 | -2,31500 | 0,710128 |  |

**Oneway Analysis of original-glcm-Id By GrG**

**Quantiles**

| **Level** | **Minimum** | **10%** | **25%** | **Median** | **75%** | **90%** | **Maximum** |
| --- | --- | --- | --- | --- | --- | --- | --- |
| 1 | 0,163151 | 0,186595 | 0,207877 | 0,242305 | 0,284283 | 0,287893 | 0,289233 |
| 2 | 0,15493 | 0,200923 | 0,228551 | 0,244956 | 0,273868 | 0,315465 | 0,686402 |
| 3 | 0,197881 | 0,202617 | 0,222318 | 0,246474 | 0,274043 | 0,293807 | 0,3039 |
| 4&5 | 0,190995 | 0,206516 | 0,232359 | 0,243817 | 0,259921 | 0,277278 | 0,291926 |

**Nonparametric Comparisons For Each Pair Using Wilcoxon Method**

| **q*** | **Alpha** |
| --- | --- |
| 1,95996 | 0,05 |

| **Level** | **- Level** | **Score Mean Difference** | **Std Err Dif** | **Z** | **p-Value** | **Hodges-Lehmann** | **Lower CL** | **Upper CL** | **Difference Plot** |
| --- | --- | --- | --- | --- | --- | --- | --- | --- | --- |
| 3 | 1 | 1,20000 | 3,214550 | 0,373303 | 0,7089 | 0,004847 | -0,019242 | 0,0352357 |  |
| 2 | 1 | 0,66087 | 3,688211 | 0,179184 | 0,8578 | 0,003553 | -0,023284 | 0,0369344 |  |
| 3 | 2 | 0,66087 | 3,688211 | 0,179184 | 0,8578 | 0,001778 | -0,024429 | 0,0264234 |  |
| 4&5 | 1 | -0,29167 | 3,500000 | -0,083333 | 0,9336 | -0,000862 | -0,026144 | 0,0298670 |  |
| 4&5 | 2 | -0,88804 | 3,839063 | -0,231318 | 0,8171 | -0,002381 | -0,021011 | 0,0134549 |  |
| 4&5 | 3 | -2,15833 | 3,500000 | -0,616667 | 0,5375 | -0,005967 | -0,027768 | 0,0181864 |  |

**Oneway Analysis of original-glcm-ClusterTendency By GrG**

**Quantiles**

| **Level** | **Minimum** | **10%** | **25%** | **Median** | **75%** | **90%** | **Maximum** |
| --- | --- | --- | --- | --- | --- | --- | --- |
| 1 | 45,5032 | 57,34683 | 67,53362 | 90,75983 | 161,0689 | 245,5518 | 328,1737 |
| 2 | 1,508091 | 31,76868 | 94,43991 | 128,5294 | 204,5919 | 325,711 | 441,1564 |
| 3 | 42,48593 | 49,90069 | 64,45936 | 117,2402 | 161,6471 | 208,5648 | 210,7755 |
| 4&5 | 54,16693 | 56,04832 | 91,91178 | 124,2582 | 293,5068 | 456,1505 | 487,5625 |

**Nonparametric Comparisons For Each Pair Using Wilcoxon Method**

| **q*** | **Alpha** |
| --- | --- |
| 1,95996 | 0,05 |

| **Level** | **- Level** | **Score Mean Difference** | **Std Err Dif** | **Z** | **p-Value** | **Hodges-Lehmann** | **Lower CL** | **Upper CL** | **Difference Plot** |
| --- | --- | --- | --- | --- | --- | --- | --- | --- | --- |
| 4&5 | 1 | 5,42500 | 3,500000 | 1,55000 | 0,1211 | 35,1389 | -11,9931 | 126,1959 |  |
| 2 | 1 | 4,73623 | 3,688211 | 1,28415 | 0,1991 | 29,1973 | -26,4624 | 76,8206 |  |
| 4&5 | 3 | 4,72500 | 3,500000 | 1,35000 | 0,1770 | 39,4684 | -11,1556 | 129,6514 |  |
| 4&5 | 2 | 1,91630 | 3,839063 | 0,49916 | 0,6177 | 12,1746 | -42,0258 | 93,4153 |  |
| 3 | 1 | 0,13333 | 3,214550 | 0,04148 | 0,9669 | 1,3835 | -41,2911 | 45,4963 |  |
| 3 | 2 | -3,52464 | 3,688211 | -0,95565 | 0,3392 | -27,1140 | -77,4922 | 17,2929 |  |

**Oneway Analysis of original-firstorder-InterquartileRange By GrG**

**Quantiles**

| **Level** | **Minimum** | **10%** | **25%** | **Median** | **75%** | **90%** | **Maximum** |
| --- | --- | --- | --- | --- | --- | --- | --- |
| 1 | 171 | 171,75 | 189 | 244 | 293,75 | 404,75 | 425 |
| 2 | 29,5 | 174,55 | 219 | 269 | 309 | 406,55 | 508 |
| 3 | 155 | 162,8 | 176,25 | 224,75 | 300 | 312,95 | 314 |
| 4&5 | 160,75 | 172,975 | 190,25 | 242,125 | 373,875 | 461,45 | 502 |

**Nonparametric Comparisons For Each Pair Using Wilcoxon Method**

| **q*** | **Alpha** |
| --- | --- |
| 1,95996 | 0,05 |

| **Level** | **- Level** | **Score Mean Difference** | **Std Err Dif** | **Z** | **p-Value** | **Hodges-Lehmann** | **Lower CL** | **Upper CL** | **Difference Plot** |
| --- | --- | --- | --- | --- | --- | --- | --- | --- | --- |
| 2 | 1 | 3,30435 | 3,688009 | 0,89597 | 0,3703 | 23,5000 | -27,5000 | 66,75000 |  |
| 4&5 | 3 | 3,09167 | 3,500000 | 0,88333 | 0,3771 | 20,3750 | -28,5000 | 89,50000 |  |
| 4&5 | 1 | 1,45833 | 3,499510 | 0,41673 | 0,6769 | 8,2500 | -42,0000 | 68,75000 |  |
| 3 | 1 | -1,60000 | 3,214550 | -0,49774 | 0,6187 | -16,0000 | -70,2500 | 44,00000 |  |
| 4&5 | 2 | -1,96304 | 3,838773 | -0,51137 | 0,6091 | -10,6250 | -58,2500 | 52,25000 |  |
| 3 | 2 | -4,95652 | 3,688009 | -1,34396 | 0,1790 | -35,7500 | -82,0000 | 17,50000 |  |

**Oneway Analysis of original-firstorder-Skewness By GrG**

**Quantiles**

| **Level** | **Minimum** | **10%** | **25%** | **Median** | **75%** | **90%** | **Maximum** |
| --- | --- | --- | --- | --- | --- | --- | --- |
| 1 | -0,84298 | -0,54597 | 0,091139 | 0,232528 | 0,399728 | 0,769249 | 0,973451 |
| 2 | -0,56721 | -0,51683 | -0,34085 | 0,034296 | 0,316028 | 0,542785 | 0,637177 |
| 3 | -0,86303 | -0,4435 | 0,017943 | 0,161139 | 0,423791 | 0,857216 | 1,321986 |
| 4&5 | -0,81795 | -0,80856 | -0,26063 | 0,291911 | 0,488828 | 0,821298 | 0,938316 |

**Nonparametric Comparisons For Each Pair Using Wilcoxon Method**

| **q*** | **Alpha** |
| --- | --- |
| 1,95996 | 0,05 |

| **Level** | **- Level** | **Score Mean Difference** | **Std Err Dif** | **Z** | **p-Value** | **Hodges-Lehmann** | **Lower CL** | **Upper CL** | **Difference Plot** |
| --- | --- | --- | --- | --- | --- | --- | --- | --- | --- |
| 4&5 | 2 | 3,87935 | 3,839063 | 1,01049 | 0,3123 | 0,152932 | -0,171257 | 0,4460109 |  |
| 3 | 2 | 2,97391 | 3,688211 | 0,80633 | 0,4201 | 0,112312 | -0,144864 | 0,4077413 |  |
| 4&5 | 3 | 0,87500 | 3,500000 | 0,25000 | 0,8026 | 0,045913 | -0,369488 | 0,3491599 |  |
| 4&5 | 1 | 0,64167 | 3,500000 | 0,18333 | 0,8545 | 0,023600 | -0,373636 | 0,3052010 |  |
| 3 | 1 | -2,00000 | 3,214550 | -0,62217 | 0,5338 | -0,055530 | -0,275811 | 0,2309888 |  |
| 2 | 1 | -4,29565 | 3,688211 | -1,16470 | 0,2441 | -0,159355 | -0,424875 | 0,1175081 |  |

**Oneway Analysis of original-firstorder-Uniformity By GrG**

**Quantiles**

| **Level** | **Minimum** | **10%** | **25%** | **Median** | **75%** | **90%** | **Maximum** |
| --- | --- | --- | --- | --- | --- | --- | --- |
| 1 | 0,036714 | 0,03989 | 0,04974 | 0,054758 | 0,0598 | 0,071167 | 0,07298 |
| 2 | 0,024622 | 0,034832 | 0,039007 | 0,048011 | 0,057216 | 0,118599 | 0,338162 |
| 3 | 0,033981 | 0,034945 | 0,038781 | 0,047036 | 0,056044 | 0,081342 | 0,095156 |
| 4&5 | 0,023847 | 0,026126 | 0,033554 | 0,044556 | 0,061184 | 0,066942 | 0,089286 |

**Nonparametric Comparisons For Each Pair Using Wilcoxon Method**

| **q*** | **Alpha** |
| --- | --- |
| 1,95996 | 0,05 |

| **Level** | **- Level** | **Score Mean Difference** | **Std Err Dif** | **Z** | **p-Value** | **Hodges-Lehmann** | **Lower CL** | **Upper CL** | **Difference Plot** |
| --- | --- | --- | --- | --- | --- | --- | --- | --- | --- |
| 3 | 2 | -1,65217 | 3,688211 | -0,44796 | 0,6542 | -0,001497 | -0,011312 | 0,0075193 |  |
| 4&5 | 3 | -2,39167 | 3,500000 | -0,68333 | 0,4944 | -0,003720 | -0,013601 | 0,0084444 |  |
| 4&5 | 2 | -4,44022 | 3,839063 | -1,15659 | 0,2474 | -0,005539 | -0,016432 | 0,0057501 |  |
| 2 | 1 | -4,51594 | 3,688211 | -1,22443 | 0,2208 | -0,005372 | -0,014017 | 0,0041108 |  |
| 3 | 1 | -5,46667 | 3,214550 | -1,70060 | 0,0890 | -0,007345 | -0,015618 | 0,0017662 |  |
| 4&5 | 1 | -6,53333 | 3,499755 | -1,86680 | 0,0619 | -0,009484 | -0,020194 | 0,0014849 |  |

**Oneway Analysis of original-firstorder-Median By GrG**

**Quantiles**

| **Level** | **Minimum** | **10%** | **25%** | **Median** | **75%** | **90%** | **Maximum** |
| --- | --- | --- | --- | --- | --- | --- | --- |
| 1 | 591,5 | 606,2 | 819 | 989,5 | 1097 | 1205,3 | 1229 |
| 2 | 158 | 603,6 | 752 | 995 | 1157 | 1345,4 | 1412 |
| 3 | 538 | 549,4 | 660 | 799 | 896 | 1008 | 1014 |
| 4&5 | 626,5 | 660,35 | 818,875 | 925,25 | 1000,125 | 1080,45 | 1092 |

**Nonparametric Comparisons For Each Pair Using Wilcoxon Method**

| **q*** | **Alpha** |
| --- | --- |
| 1,95996 | 0,05 |

| **Level** | **- Level** | **Score Mean Difference** | **Std Err Dif** | **Z** | **p-Value** | **Hodges-Lehmann** | **Lower CL** | **Upper CL** | **Difference Plot** |
| --- | --- | --- | --- | --- | --- | --- | --- | --- | --- |
| 4&5 | 3 | 8,34167 | 3,500000 | 2,38333 | 0,0172* | 131,000 | 17,500 | 231,500 |  |
| 2 | 1 | 1,15652 | 3,688009 | 0,31359 | 0,7538 | 26,000 | -132,000 | 175,000 |  |
| 4&5 | 1 | -2,97500 | 3,500000 | -0,85000 | 0,3953 | -43,750 | -169,000 | 66,500 |  |
| 4&5 | 2 | -4,53370 | 3,839063 | -1,18094 | 0,2376 | -69,750 | -192,500 | 61,500 |  |
| 3 | 1 | -8,00000 | 3,214550 | -2,48868 | 0,0128* | -183,000 | -308,000 | -32,000 |  |
| 3 | 2 | -9,47246 | 3,688211 | -2,56831 | 0,0102* | -193,500 | -343,000 | -47,000 |  |

**Oneway Analysis of original-firstorder-Energy By GrG**

**Quantiles**

| **Level** | **Minimum** | **10%** | **25%** | **Median** | **75%** | **90%** | **Maximum** |
| --- | --- | --- | --- | --- | --- | --- | --- |
| 1 | 32567831 | 35745327 | 40486614 | 1,066e+8 | 1,632e+8 | 4,125e+8 | 4,356e+8 |
| 2 | 1236184 | 15753948 | 73559774 | 98671271 | 3,789e+8 | 1,347e+9 | 3,998e+9 |
| 3 | 26441659 | 62688674 | 1,015e+8 | 1,304e+8 | 1,978e+8 | 4,95e+8 | 6,228e+8 |
| 4&5 | 25692711 | 47405962 | 74630262 | 3,343e+8 | 1,877e+9 | 3,884e+9 | 2,1e+10 |

**Nonparametric Comparisons For Each Pair Using Wilcoxon Method**

| **q*** | **Alpha** |
| --- | --- |
| 1,95996 | 0,05 |

| **Level** | **- Level** | **Score Mean Difference** | **Std Err Dif** | **Z** | **p-Value** | **Hodges-Lehmann** | **Lower CL** | **Upper CL** | **Difference Plot** |
| --- | --- | --- | --- | --- | --- | --- | --- | --- | --- |
| 4&5 | 1 | 7,758333 | 3,500000 | 2,216667 | 0,0266* | 204688479 | 10423073 | 377496066 |  |
| 4&5 | 2 | 5,935870 | 3,839063 | 1,546177 | 0,1221 | 104951467 | -26322029 | 342240177 |  |
| 4&5 | 3 | 5,075000 | 3,500000 | 1,450000 | 0,1471 | 156484496 | -39784859 | 340864747 |  |
| 3 | 1 | 4,266667 | 3,214550 | 1,327298 | 0,1844 | 39994796 | -17467360 | 97042748 |  |
| 2 | 1 | 2,533333 | 3,688211 | 0,686873 | 0,4922 | 33496462 | -37716310 | 165599861 |  |
| 3 | 2 | 1,652174 | 3,688211 | 0,447961 | 0,6542 | 15798133 | -1,353e+8 | 85000047 |  |

**Oneway Analysis of original-firstorder-RobustMeanAbsoluteDeviation By GrG**

**Quantiles**

| **Level** | **Minimum** | **10%** | **25%** | **Median** | **75%** | **90%** | **Maximum** |
| --- | --- | --- | --- | --- | --- | --- | --- |
| 1 | 72,12731 | 73,74058 | 79,23054 | 98,92563 | 126,8596 | 169,5167 | 169,9093 |
| 2 | 13,57618 | 71,39014 | 90,93503 | 107,8697 | 132,8252 | 169,7197 | 201,0735 |
| 3 | 63,00053 | 64,86234 | 73,64546 | 94,77605 | 126,4278 | 130,7897 | 135,5653 |
| 4&5 | 73,76216 | 74,37462 | 80,8275 | 107,011 | 149,0379 | 190,8551 | 200,1718 |

**Nonparametric Comparisons For Each Pair Using Wilcoxon Method**

| **q*** | **Alpha** |
| --- | --- |
| 1,95996 | 0,05 |

| **Level** | **- Level** | **Score Mean Difference** | **Std Err Dif** | **Z** | **p-Value** | **Hodges-Lehmann** | **Lower CL** | **Upper CL** | **Difference Plot** |
| --- | --- | --- | --- | --- | --- | --- | --- | --- | --- |
| 4&5 | 3 | 4,14167 | 3,500000 | 1,18333 | 0,2367 | 12,6682 | -9,1170 | 38,82511 |  |
| 2 | 1 | 3,63478 | 3,688211 | 0,98551 | 0,3244 | 9,6713 | -10,7214 | 27,99574 |  |
| 4&5 | 1 | 1,80833 | 3,500000 | 0,51667 | 0,6054 | 5,8548 | -13,2886 | 30,26255 |  |
| 4&5 | 2 | -0,79457 | 3,839063 | -0,20697 | 0,8360 | -1,7672 | -20,5966 | 23,95083 |  |
| 3 | 1 | -2,00000 | 3,214550 | -0,62217 | 0,5338 | -4,8913 | -27,9962 | 16,12889 |  |
| 3 | 2 | -5,06667 | 3,688211 | -1,37375 | 0,1695 | -15,0303 | -35,3571 | 6,53312 |  |

**Oneway Analysis of original-firstorder-MeanAbsoluteDeviation By GrG**

**Quantiles**

| **Level** | **Minimum** | **10%** | **25%** | **Median** | **75%** | **90%** | **Maximum** |
| --- | --- | --- | --- | --- | --- | --- | --- |
| 1 | 106,1502 | 108,9202 | 118,1994 | 129,49 | 171,5249 | 218,033 | 232,9351 |
| 2 | 17,37347 | 97,03313 | 128,2496 | 159,5524 | 173,4491 | 237,5509 | 269,5574 |
| 3 | 94,40554 | 97,82035 | 106,2407 | 141,8625 | 165,1065 | 188,4655 | 188,4726 |
| 4&5 | 101,434 | 106,5492 | 120,6273 | 153,7895 | 211,009 | 252,7456 | 277,1082 |

**Nonparametric Comparisons For Each Pair Using Wilcoxon Method**

| **q*** | **Alpha** |
| --- | --- |
| 1,95996 | 0,05 |

| **Level** | **- Level** | **Score Mean Difference** | **Std Err Dif** | **Z** | **p-Value** | **Hodges-Lehmann** | **Lower CL** | **Upper CL** | **Difference Plot** |
| --- | --- | --- | --- | --- | --- | --- | --- | --- | --- |
| 4&5 | 3 | 4,49167 | 3,500000 | 1,28333 | 0,1994 | 18,7753 | -13,9998 | 54,87299 |  |
| 2 | 1 | 4,07536 | 3,688211 | 1,10497 | 0,2692 | 14,8285 | -16,0408 | 42,07606 |  |
| 4&5 | 1 | 2,97500 | 3,500000 | 0,85000 | 0,3953 | 10,3718 | -14,0118 | 47,77668 |  |
| 4&5 | 2 | -0,14022 | 3,839063 | -0,03652 | 0,9709 | -0,7098 | -27,2166 | 36,27204 |  |
| 3 | 1 | -0,66667 | 3,214550 | -0,20739 | 0,8357 | -4,3059 | -29,3931 | 23,20911 |  |
| 3 | 2 | -4,73623 | 3,688211 | -1,28415 | 0,1991 | -19,7516 | -42,7758 | 11,16712 |  |

**Oneway Analysis of original-firstorder-TotalEnergy By GrG**

**Quantiles**

| **Level** | **Minimum** | **10%** | **25%** | **Median** | **75%** | **90%** | **Maximum** |
| --- | --- | --- | --- | --- | --- | --- | --- |
| 1 | 2,084e+8 | 2,288e+8 | 2,591e+8 | 6,821e+8 | 1,045e+9 | 2,64e+9 | 2,788e+9 |
| 2 | 7934058 | 1,119e+8 | 4,708e+8 | 7,088e+8 | 2,197e+9 | 8,557e+9 | 2,56e+10 |
| 3 | 2,048e+8 | 4,154e+8 | 6,888e+8 | 8,99e+8 | 1,266e+9 | 3,874e+9 | 5,751e+9 |
| 4&5 | 1,644e+8 | 3,034e+8 | 6,138e+8 | 1,967e+9 | 1,2e+10 | 2,97e+10 | 1,8e+11 |

**Nonparametric Comparisons For Each Pair Using Wilcoxon Method**

| **q*** | **Alpha** |
| --- | --- |
| 1,95996 | 0,05 |

| **Level** | **- Level** | **Score Mean Difference** | **Std Err Dif** | **Z** | **p-Value** | **Hodges-Lehmann** | **Lower CL** | **Upper CL** | **Difference Plot** |
| --- | --- | --- | --- | --- | --- | --- | --- | --- | --- |
| 4&5 | 1 | 8,225000 | 3,500000 | 2,350000 | 0,0188* | 1,1581e+9 | 85382249 | 2,4053e+9 |  |
| 4&5 | 2 | 6,777174 | 3,839063 | 1,765320 | 0,0775 | 742870974 | -1,468e+8 | 2,1986e+9 |  |
| 4&5 | 3 | 5,308333 | 3,500000 | 1,516667 | 0,1294 | 864281544 | -1,89e+8 | 2,1255e+9 |  |
| 3 | 1 | 4,666667 | 3,214550 | 1,451732 | 0,1466 | 279834556 | -98032092 | 642859891 |  |
| 2 | 1 | 2,202899 | 3,688211 | 0,597281 | 0,5503 | 186193449 | -2,687e+8 | 1,0068e+9 |  |
| 3 | 2 | 2,202899 | 3,688211 | 0,597281 | 0,5503 | 143752936 | -5,74e+8 | 556660976 |  |

**Oneway Analysis of original-firstorder-Maximum By GrG**

**Quantiles**

| **Level** | **Minimum** | **10%** | **25%** | **Median** | **75%** | **90%** | **Maximum** |
| --- | --- | --- | --- | --- | --- | --- | --- |
| 1 | 1156 | 1176,4 | 1286 | 1389 | 1543 | 1558,8 | 1569 |
| 2 | 206 | 966,2 | 1146 | 1464 | 1793 | 2005,2 | 2143 |
| 3 | 1032 | 1053,6 | 1151 | 1323 | 1424 | 1496,4 | 1578 |
| 4&5 | 1042 | 1062,2 | 1149 | 1511,5 | 1783,75 | 2113 | 2492 |

**Nonparametric Comparisons For Each Pair Using Wilcoxon Method**

| **q*** | **Alpha** |
| --- | --- |
| 1,95996 | 0,05 |

| **Level** | **- Level** | **Score Mean Difference** | **Std Err Dif** | **Z** | **p-Value** | **Hodges-Lehmann** | **Lower CL** | **Upper CL** | **Difference Plot** |
| --- | --- | --- | --- | --- | --- | --- | --- | --- | --- |
| 4&5 | 3 | 5,89167 | 3,500000 | 1,68333 | 0,0923 | 201,000 | -26,000 | 468,0000 |  |
| 4&5 | 1 | 2,33333 | 3,499755 | 0,66671 | 0,5050 | 139,500 | -147,000 | 388,0000 |  |
| 2 | 1 | 2,31304 | 3,688211 | 0,62715 | 0,5306 | 60,000 | -144,000 | 329,0000 |  |
| 4&5 | 2 | 0,65435 | 3,838918 | 0,17045 | 0,8647 | 23,000 | -220,000 | 321,0000 |  |
| 3 | 1 | -5,33333 | 3,214550 | -1,65912 | 0,0971 | -110,000 | -220,000 | 29,0000 |  |
| 3 | 2 | -5,50725 | 3,688211 | -1,49320 | 0,1354 | -169,000 | -431,000 | 45,0000 |  |

**Oneway Analysis of original-firstorder-RootMeanSquared By GrG**

**Quantiles**

| **Level** | **Minimum** | **10%** | **25%** | **Median** | **75%** | **90%** | **Maximum** |
| --- | --- | --- | --- | --- | --- | --- | --- |
| 1 | 610,1811 | 640,857 | 852,5243 | 997,1622 | 1098,279 | 1170,202 | 1223,521 |
| 2 | 162,1783 | 639,1115 | 788,2813 | 1013,678 | 1184,365 | 1358,035 | 1410,001 |
| 3 | 561,8323 | 579,3801 | 716,3731 | 821,6394 | 881,8707 | 1017,419 | 1020,287 |
| 4&5 | 659,243 | 687,4946 | 824,2079 | 929,5518 | 1069,425 | 1119,038 | 1122,147 |

**Nonparametric Comparisons For Each Pair Using Wilcoxon Method**

| **q*** | **Alpha** |
| --- | --- |
| 1,95996 | 0,05 |

| **Level** | **- Level** | **Score Mean Difference** | **Std Err Dif** | **Z** | **p-Value** | **Hodges-Lehmann** | **Lower CL** | **Upper CL** | **Difference Plot** |
| --- | --- | --- | --- | --- | --- | --- | --- | --- | --- |
| 4&5 | 3 | 8,80833 | 3,500000 | 2,51667 | 0,0118* | 121,617 | 37,286 | 239,864 |  |
| 2 | 1 | 1,65217 | 3,688211 | 0,44796 | 0,6542 | 34,504 | -127,433 | 186,479 |  |
| 4&5 | 1 | -1,92500 | 3,500000 | -0,55000 | 0,5823 | -28,833 | -149,630 | 70,843 |  |
| 4&5 | 2 | -3,59891 | 3,839063 | -0,93745 | 0,3485 | -59,520 | -201,346 | 67,267 |  |
| 3 | 1 | -8,80000 | 3,214550 | -2,73755 | 0,0062* | -175,013 | -284,055 | -41,644 |  |
| 3 | 2 | -9,47246 | 3,688211 | -2,56831 | 0,0102* | -195,511 | -343,308 | -51,642 |  |

**Oneway Analysis of original-firstorder-90Percentile By GrG**

**Quantiles**

| **Level** | **Minimum** | **10%** | **25%** | **Median** | **75%** | **90%** | **Maximum** |
| --- | --- | --- | --- | --- | --- | --- | --- |
| 1 | 779,5 | 807,16 | 1061,4 | 1236 | 1288,7 | 1395,84 | 1398,6 |
| 2 | 193 | 879,64 | 1001 | 1204,7 | 1431 | 1620,48 | 1699,2 |
| 3 | 793,8 | 799,08 | 934,2 | 1026,3 | 1082,5 | 1178,96 | 1189,4 |
| 4&5 | 837,1 | 878,7 | 989,225 | 1165,35 | 1368,075 | 1507,88 | 1537 |

**Nonparametric Comparisons For Each Pair Using Wilcoxon Method**

| **q*** | **Alpha** |
| --- | --- |
| 1,95996 | 0,05 |

| **Level** | **- Level** | **Score Mean Difference** | **Std Err Dif** | **Z** | **p-Value** | **Hodges-Lehmann** | **Lower CL** | **Upper CL** | **Difference Plot** |
| --- | --- | --- | --- | --- | --- | --- | --- | --- | --- |
| 4&5 | 3 | 8,22500 | 3,500000 | 2,35000 | 0,0188* | 161,550 | 34,500 | 320,800 |  |
| 2 | 1 | 1,65217 | 3,688211 | 0,44796 | 0,6542 | 39,900 | -140,900 | 215,900 |  |
| 4&5 | 1 | 0,29167 | 3,500000 | 0,08333 | 0,9336 | 6,800 | -157,800 | 148,100 |  |
| 4&5 | 2 | -1,63587 | 3,839063 | -0,42611 | 0,6700 | -40,850 | -205,900 | 130,300 |  |
| 3 | 1 | -9,06667 | 3,214550 | -2,82051 | 0,0048* | -208,000 | -302,600 | -63,400 |  |
| 3 | 2 | -9,25217 | 3,688211 | -2,50858 | 0,0121* | -208,600 | -386,500 | -47,400 |  |

**Oneway Analysis of original-firstorder-Minimum By GrG**

**Quantiles**

| **Level** | **Minimum** | **10%** | **25%** | **Median** | **75%** | **90%** | **Maximum** |
| --- | --- | --- | --- | --- | --- | --- | --- |
| 1 | 0 | 199,8 | 368 | 531 | 758 | 773,8 | 778 |
| 2 | 0 | 116,8 | 291 | 459 | 680 | 814,6 | 995 |
| 3 | 0 | 0 | 13 | 387 | 511 | 711,2 | 752 |
| 4&5 | 0 | 53,5 | 243,75 | 391,5 | 520,5 | 647,5 | 680 |

**Nonparametric Comparisons For Each Pair Using Wilcoxon Method**

| **q*** | **Alpha** |
| --- | --- |
| 1,95996 | 0,05 |

| **Level** | **- Level** | **Score Mean Difference** | **Std Err Dif** | **Z** | **p-Value** | **Hodges-Lehmann** | **Lower CL** | **Upper CL** | **Difference Plot** |
| --- | --- | --- | --- | --- | --- | --- | --- | --- | --- |
| 4&5 | 3 | 0,40833 | 3,497303 | 0,11676 | 0,9071 | 2,500 | -146,000 | 184,000 |  |
| 2 | 1 | -3,19420 | 3,687807 | -0,86615 | 0,3864 | -65,000 | -236,000 | 107,000 |  |
| 3 | 2 | -4,90145 | 3,686192 | -1,32968 | 0,1836 | -120,000 | -301,000 | 70,000 |  |
| 4&5 | 2 | -5,28152 | 3,838483 | -1,37594 | 0,1688 | -98,500 | -252,000 | 52,000 |  |
| 3 | 1 | -6,60000 | 3,210973 | -2,05545 | 0,0398* | -177,000 | -368,000 | 0,000 |  |
| 4&5 | 1 | -7,46667 | 3,499755 | -2,13348 | 0,0329* | -157,500 | -324,000 | -23,000 |  |

**Oneway Analysis of original-firstorder-Entropy By GrG**

**Quantiles**

| **Level** | **Minimum** | **10%** | **25%** | **Median** | **75%** | **90%** | **Maximum** |
| --- | --- | --- | --- | --- | --- | --- | --- |
| 1 | 3,958229 | 3,968708 | 4,305573 | 4,413679 | 4,558817 | 4,943269 | 4,959687 |
| 2 | 1,658936 | 3,284005 | 4,316825 | 4,668088 | 4,91336 | 5,071722 | 5,497533 |
| 3 | 3,679395 | 3,888965 | 4,3242 | 4,591196 | 4,90703 | 5,067323 | 5,076317 |
| 4&5 | 3,682006 | 4,067198 | 4,388348 | 4,692089 | 5,204987 | 5,532977 | 5,585106 |

**Nonparametric Comparisons For Each Pair Using Wilcoxon Method**

| **q*** | **Alpha** |
| --- | --- |
| 1,95996 | 0,05 |

| **Level** | **- Level** | **Score Mean Difference** | **Std Err Dif** | **Z** | **p-Value** | **Hodges-Lehmann** | **Lower CL** | **Upper CL** | **Difference Plot** |
| --- | --- | --- | --- | --- | --- | --- | --- | --- | --- |
| 4&5 | 1 | 6,941667 | 3,500000 | 1,983333 | 0,0473* | 0,2794007 | -0,008126 | 0,6433301 |  |
| 3 | 1 | 5,333333 | 3,214550 | 1,659123 | 0,0971 | 0,2119271 | -0,083689 | 0,4740434 |  |
| 4&5 | 2 | 4,533696 | 3,839063 | 1,180938 | 0,2376 | 0,1889640 | -0,180484 | 0,5632998 |  |
| 2 | 1 | 4,295652 | 3,688211 | 1,164698 | 0,2441 | 0,1723308 | -0,146200 | 0,4376186 |  |
| 4&5 | 3 | 2,508333 | 3,500000 | 0,716667 | 0,4736 | 0,1449371 | -0,216275 | 0,4945502 |  |
| 3 | 2 | 0,881159 | 3,688211 | 0,238912 | 0,8112 | 0,0298659 | -0,261809 | 0,3447410 |  |

**Oneway Analysis of original-firstorder-Range By GrG**

**Quantiles**

| **Level** | **Minimum** | **10%** | **25%** | **Median** | **75%** | **90%** | **Maximum** |
| --- | --- | --- | --- | --- | --- | --- | --- |
| 1 | 520 | 530,8 | 596 | 849 | 1029 | 1245,6 | 1290 |
| 2 | 76 | 407,2 | 680 | 1001 | 1284 | 1536,8 | 1702 |
| 3 | 487 | 588,4 | 688 | 845 | 1147 | 1488,6 | 1578 |
| 4&5 | 488 | 609,8 | 769,75 | 988,5 | 1486,25 | 1943,8 | 2492 |

**Nonparametric Comparisons For Each Pair Using Wilcoxon Method**

| **q*** | **Alpha** |
| --- | --- |
| 1,95996 | 0,05 |

| **Level** | **- Level** | **Score Mean Difference** | **Std Err Dif** | **Z** | **p-Value** | **Hodges-Lehmann** | **Lower CL** | **Upper CL** | **Difference Plot** |
| --- | --- | --- | --- | --- | --- | --- | --- | --- | --- |
| 4&5 | 1 | 6,12500 | 3,500000 | 1,75000 | 0,0801 | 206,000 | -32,000 | 527,0000 |  |
| 4&5 | 3 | 4,31667 | 3,499755 | 1,23342 | 0,2174 | 157,000 | -119,000 | 486,0000 |  |
| 2 | 1 | 3,74493 | 3,688211 | 1,01538 | 0,3099 | 142,000 | -111,000 | 378,0000 |  |
| 4&5 | 2 | 3,22500 | 3,839063 | 0,84005 | 0,4009 | 114,500 | -171,000 | 442,0000 |  |
| 3 | 1 | 1,73333 | 3,214550 | 0,53921 | 0,5897 | 73,000 | -138,000 | 277,0000 |  |
| 3 | 2 | -1,43188 | 3,688211 | -0,38823 | 0,6978 | -53,000 | -334,000 | 186,0000 |  |

**Oneway Analysis of original-firstorder-Variance By GrG**

**Quantiles**

| **Level** | **Minimum** | **10%** | **25%** | **Median** | **75%** | **90%** | **Maximum** |
| --- | --- | --- | --- | --- | --- | --- | --- |
| 1 | 17709,3 | 18473,83 | 19513,37 | 24750,35 | 45110,1 | 64643,36 | 77505,88 |
| 2 | 428,7225 | 13530,65 | 25269,64 | 35964,37 | 48148,36 | 85369,79 | 105212,5 |
| 3 | 15435,14 | 15544,98 | 17769,28 | 31969,9 | 37681,46 | 54855,17 | 58702,06 |
| 4&5 | 14562,47 | 17704,63 | 25020,3 | 34403,18 | 69472,36 | 100149,3 | 116634,2 |

**Nonparametric Comparisons For Each Pair Using Wilcoxon Method**

| **q*** | **Alpha** |
| --- | --- |
| 1,95996 | 0,05 |

| **Level** | **- Level** | **Score Mean Difference** | **Std Err Dif** | **Z** | **p-Value** | **Hodges-Lehmann** | **Lower CL** | **Upper CL** | **Difference Plot** |
| --- | --- | --- | --- | --- | --- | --- | --- | --- | --- |
| 4&5 | 3 | 4,37500 | 3,500000 | 1,25000 | 0,2113 | 8233,04 | -5095,2 | 26835,67 |  |
| 4&5 | 1 | 4,02500 | 3,500000 | 1,15000 | 0,2501 | 5546,48 | -4950,9 | 23802,42 |  |
| 2 | 1 | 3,52464 | 3,688211 | 0,95565 | 0,3392 | 5666,13 | -6844,5 | 17506,11 |  |
| 4&5 | 2 | 0,79457 | 3,839063 | 0,20697 | 0,8360 | 864,13 | -11836,8 | 17955,11 |  |
| 3 | 1 | -1,06667 | 3,214550 | -0,33182 | 0,7400 | -1744,09 | -11718,0 | 10356,83 |  |
| 3 | 2 | -3,96522 | 3,688211 | -1,07511 | 0,2823 | -7580,16 | -17146,2 | 4788,37 |  |

**Oneway Analysis of original-firstorder-10Percentile By GrG**

**Quantiles**

| **Level** | **Minimum** | **10%** | **25%** | **Median** | **75%** | **90%** | **Maximum** |
| --- | --- | --- | --- | --- | --- | --- | --- |
| 1 | 399,5 | 441,8 | 661 | 695,1 | 819,3 | 957,76 | 1030 |
| 2 | 1,8 | 261,76 | 600,7 | 762 | 866,6 | 1016,64 | 1143,2 |
| 3 | 211,8 | 284,52 | 483,7 | 585,4 | 685,3 | 854,32 | 860,8 |
| 4&5 | 485,6 | 500,66 | 581,325 | 678,6 | 730,5 | 824,94 | 880,4 |

**Nonparametric Comparisons For Each Pair Using Wilcoxon Method**

| **q*** | **Alpha** |
| --- | --- |
| 1,95996 | 0,05 |

| **Level** | **- Level** | **Score Mean Difference** | **Std Err Dif** | **Z** | **p-Value** | **Hodges-Lehmann** | **Lower CL** | **Upper CL** | **Difference Plot** |
| --- | --- | --- | --- | --- | --- | --- | --- | --- | --- |
| 4&5 | 3 | 6,24167 | 3,500000 | 1,78333 | 0,0745 | 89,200 | -8,100 | 187,900 |  |
| 2 | 1 | 1,32174 | 3,688211 | 0,35837 | 0,7201 | 27,600 | -111,400 | 146,200 |  |
| 4&5 | 1 | -4,37500 | 3,500000 | -1,25000 | 0,2113 | -72,600 | -167,000 | 34,300 |  |
| 4&5 | 2 | -6,30978 | 3,839063 | -1,64357 | 0,1003 | -85,200 | -186,200 | 21,200 |  |
| 3 | 1 | -7,20000 | 3,214550 | -2,23982 | 0,0251* | -149,600 | -280,600 | -18,400 |  |
| 3 | 2 | -8,48116 | 3,688211 | -2,29953 | 0,0215* | -176,600 | -296,000 | -36,400 |  |

**Oneway Analysis of original-firstorder-Kurtosis By GrG**

**Quantiles**

| **Level** | **Minimum** | **10%** | **25%** | **Median** | **75%** | **90%** | **Maximum** |
| --- | --- | --- | --- | --- | --- | --- | --- |
| 1 | 1,704959 | 1,906554 | 2,173563 | 2,564532 | 3,720642 | 4,534278 | 4,832345 |
| 2 | 1,46581 | 1,8733 | 2,259067 | 2,696254 | 3,40795 | 3,850169 | 3,879078 |
| 3 | 2,062616 | 2,202371 | 2,526301 | 2,904763 | 3,647208 | 4,638206 | 5,654035 |
| 4&5 | 1,859402 | 2,316412 | 2,598297 | 2,874176 | 3,646391 | 4,224267 | 4,611982 |

**Nonparametric Comparisons For Each Pair Using Wilcoxon Method**

| **q*** | **Alpha** |
| --- | --- |
| 1,95996 | 0,05 |

| **Level** | **- Level** | **Score Mean Difference** | **Std Err Dif** | **Z** | **p-Value** | **Hodges-Lehmann** | **Lower CL** | **Upper CL** | **Difference Plot** |
| --- | --- | --- | --- | --- | --- | --- | --- | --- | --- |
| 4&5 | 2 | 5,46848 | 3,839063 | 1,42443 | 0,1543 | 0,337972 | -0,093110 | 0,7525024 |  |
| 4&5 | 1 | 4,60833 | 3,500000 | 1,31667 | 0,1880 | 0,308036 | -0,288401 | 0,8012258 |  |
| 3 | 2 | 4,40580 | 3,688211 | 1,19456 | 0,2323 | 0,234721 | -0,204515 | 0,7670292 |  |
| 3 | 1 | 2,40000 | 3,214550 | 0,74661 | 0,4553 | 0,225356 | -0,374582 | 0,8400243 |  |
| 4&5 | 3 | 1,45833 | 3,500000 | 0,41667 | 0,6769 | 0,074703 | -0,426297 | 0,5405283 |  |
| 2 | 1 | -0,44058 | 3,688211 | -0,11946 | 0,9049 | -0,027061 | -0,628687 | 0,4441181 |  |

**Oneway Analysis of original-firstorder-Mean By GrG**

**Quantiles**

| **Level** | **Minimum** | **10%** | **25%** | **Median** | **75%** | **90%** | **Maximum** |
| --- | --- | --- | --- | --- | --- | --- | --- |
| 1 | 590,003 | 621,3944 | 842,0739 | 985,875 | 1089,519 | 1152,244 | 1215,739 |
| 2 | 160,8511 | 579,2458 | 769,2839 | 997,75 | 1160,328 | 1337,322 | 1397,189 |
| 3 | 513,191 | 541,6334 | 674,1575 | 806,8089 | 872,9706 | 1009,11 | 1012,695 |
| 4&5 | 644,4341 | 672,1762 | 810,4063 | 902,761 | 1042,829 | 1097,041 | 1100,615 |

**Nonparametric Comparisons For Each Pair Using Wilcoxon Method**

| **q*** | **Alpha** |
| --- | --- |
| 1,95996 | 0,05 |

| **Level** | **- Level** | **Score Mean Difference** | **Std Err Dif** | **Z** | **p-Value** | **Hodges-Lehmann** | **Lower CL** | **Upper CL** | **Difference Plot** |
| --- | --- | --- | --- | --- | --- | --- | --- | --- | --- |
| 4&5 | 3 | 8,45833 | 3,500000 | 2,41667 | 0,0157* | 123,429 | 30,411 | 231,905 |  |
| 2 | 1 | 1,54203 | 3,688211 | 0,41810 | 0,6759 | 37,881 | -131,312 | 181,450 |  |
| 4&5 | 1 | -2,27500 | 3,500000 | -0,65000 | 0,5157 | -41,312 | -159,221 | 62,123 |  |
| 4&5 | 2 | -4,15978 | 3,839063 | -1,08354 | 0,2786 | -61,994 | -201,974 | 64,318 |  |
| 3 | 1 | -8,66667 | 3,214550 | -2,69607 | 0,0070* | -172,529 | -291,119 | -38,779 |  |
| 3 | 2 | -9,25217 | 3,688211 | -2,50858 | 0,0121* | -199,023 | -349,131 | -50,208 |  |

**Oneway Analysis of original-glrlm-ShortRunLowGrayLevelEmphasis By GrG**

**Quantiles**

| **Level** | **Minimum** | **10%** | **25%** | **Median** | **75%** | **90%** | **Maximum** |
| --- | --- | --- | --- | --- | --- | --- | --- |
| 1 | 0,005238 | 0,005547 | 0,011366 | 0,019591 | 0,042075 | 0,059046 | 0,073114 |
| 2 | 0,001993 | 0,00234 | 0,008955 | 0,014922 | 0,034498 | 0,108923 | 0,399814 |
| 3 | 0,002236 | 0,004671 | 0,010179 | 0,016152 | 0,028822 | 0,039831 | 0,0508 |
| 4&5 | 0,000964 | 0,00198 | 0,003957 | 0,008427 | 0,020049 | 0,05219 | 0,071207 |

**Nonparametric Comparisons For Each Pair Using Wilcoxon Method**

| **q*** | **Alpha** |
| --- | --- |
| 1,95996 | 0,05 |

| **Level** | **- Level** | **Score Mean Difference** | **Std Err Dif** | **Z** | **p-Value** | **Hodges-Lehmann** | **Lower CL** | **Upper CL** | **Difference Plot** |
| --- | --- | --- | --- | --- | --- | --- | --- | --- | --- |
| 3 | 2 | -0,66087 | 3,688211 | -0,17918 | 0,8578 | -0,000373 | -0,012426 | 0,008792 |  |
| 3 | 1 | -2,93333 | 3,214550 | -0,91252 | 0,3615 | -0,005565 | -0,020844 | 0,004787 |  |
| 2 | 1 | -3,30435 | 3,688211 | -0,89592 | 0,3703 | -0,003880 | -0,017227 | 0,007852 |  |
| 4&5 | 2 | -5,28152 | 3,839063 | -1,37573 | 0,1689 | -0,006545 | -0,015253 | 0,001653 |  |
| 4&5 | 3 | -5,65833 | 3,500000 | -1,61667 | 0,1060 | -0,006104 | -0,012436 | 0,002020 |  |
| 4&5 | 1 | -7,40833 | 3,500000 | -2,11667 | 0,0343* | -0,010769 | -0,026550 | -0,000807 |  |

**Oneway Analysis of original-glrlm-GrayLevelVariance By GrG**

**Quantiles**

| **Level** | **Minimum** | **10%** | **25%** | **Median** | **75%** | **90%** | **Maximum** |
| --- | --- | --- | --- | --- | --- | --- | --- |
| 1 | 29,4121 | 29,87477 | 31,50573 | 40,53267 | 73,12833 | 102,9689 | 124,2176 |
| 2 | 0,661157 | 22,09564 | 41,01385 | 58,81886 | 78,09252 | 134,2721 | 169,6237 |
| 3 | 25,16583 | 25,18692 | 28,71207 | 50,74785 | 60,20597 | 88,41265 | 95,21441 |
| 4&5 | 23,34965 | 28,65012 | 40,49747 | 56,1905 | 112,1513 | 161,7546 | 187,5598 |

**Nonparametric Comparisons For Each Pair Using Wilcoxon Method**

| **q*** | **Alpha** |
| --- | --- |
| 1,95996 | 0,05 |

| **Level** | **- Level** | **Score Mean Difference** | **Std Err Dif** | **Z** | **p-Value** | **Hodges-Lehmann** | **Lower CL** | **Upper CL** | **Difference Plot** |
| --- | --- | --- | --- | --- | --- | --- | --- | --- | --- |
| 4&5 | 3 | 4,37500 | 3,500000 | 1,25000 | 0,2113 | 13,5631 | -8,0732 | 42,79753 |  |
| 4&5 | 1 | 4,14167 | 3,500000 | 1,18333 | 0,2367 | 9,0861 | -7,7958 | 38,31479 |  |
| 2 | 1 | 3,52464 | 3,688211 | 0,95565 | 0,3392 | 9,5081 | -10,7106 | 28,50750 |  |
| 4&5 | 2 | 0,79457 | 3,839063 | 0,20697 | 0,8360 | 1,4586 | -18,6749 | 29,19060 |  |
| 3 | 1 | -1,33333 | 3,214550 | -0,41478 | 0,6783 | -2,9063 | -18,7981 | 16,57147 |  |
| 3 | 2 | -4,29565 | 3,688211 | -1,16470 | 0,2441 | -12,3018 | -27,6479 | 7,52245 |  |

**Oneway Analysis of original-glrlm-LowGrayLevelRunEmphasis By GrG**

**Quantiles**

| **Level** | **Minimum** | **10%** | **25%** | **Median** | **75%** | **90%** | **Maximum** |
| --- | --- | --- | --- | --- | --- | --- | --- |
| 1 | 0,005298 | 0,005627 | 0,011388 | 0,019651 | 0,043256 | 0,060886 | 0,077025 |
| 2 | 0,002042 | 0,002389 | 0,008991 | 0,015016 | 0,034639 | 0,114009 | 0,46662 |
| 3 | 0,00227 | 0,004728 | 0,010223 | 0,016565 | 0,029243 | 0,040592 | 0,051144 |
| 4&5 | 0,00099 | 0,002038 | 0,004044 | 0,008539 | 0,020177 | 0,05233 | 0,072734 |

**Nonparametric Comparisons For Each Pair Using Wilcoxon Method**

| **q*** | **Alpha** |
| --- | --- |
| 1,95996 | 0,05 |

| **Level** | **- Level** | **Score Mean Difference** | **Std Err Dif** | **Z** | **p-Value** | **Hodges-Lehmann** | **Lower CL** | **Upper CL** | **Difference Plot** |
| --- | --- | --- | --- | --- | --- | --- | --- | --- | --- |
| 3 | 2 | -0,55072 | 3,688211 | -0,14932 | 0,8813 | -0,000309 | -0,012352 | 0,008821 |  |
| 3 | 1 | -2,93333 | 3,214550 | -0,91252 | 0,3615 | -0,005638 | -0,021166 | 0,004925 |  |
| 2 | 1 | -3,30435 | 3,688211 | -0,89592 | 0,3703 | -0,004146 | -0,017266 | 0,007807 |  |
| 4&5 | 2 | -5,28152 | 3,839063 | -1,37573 | 0,1689 | -0,006614 | -0,015116 | 0,001686 |  |
| 4&5 | 3 | -5,65833 | 3,500000 | -1,61667 | 0,1060 | -0,006170 | -0,012831 | 0,002137 |  |
| 4&5 | 1 | -7,40833 | 3,500000 | -2,11667 | 0,0343* | -0,010507 | -0,027094 | -0,000837 |  |

**Oneway Analysis of original-glrlm-GrayLevelNonUniformityNormalized By GrG**

**Quantiles**

| **Level** | **Minimum** | **10%** | **25%** | **Median** | **75%** | **90%** | **Maximum** |
| --- | --- | --- | --- | --- | --- | --- | --- |
| 1 | 0,036636 | 0,03965 | 0,049779 | 0,054225 | 0,058773 | 0,071168 | 0,072892 |
| 2 | 0,024548 | 0,034658 | 0,038427 | 0,04764 | 0,056693 | 0,117559 | 0,336305 |
| 3 | 0,033789 | 0,034705 | 0,038686 | 0,046729 | 0,055853 | 0,080133 | 0,093269 |
| 4&5 | 0,023779 | 0,025823 | 0,033274 | 0,04435 | 0,059966 | 0,066305 | 0,087708 |

**Nonparametric Comparisons For Each Pair Using Wilcoxon Method**

| **q*** | **Alpha** |
| --- | --- |
| 1,95996 | 0,05 |

| **Level** | **- Level** | **Score Mean Difference** | **Std Err Dif** | **Z** | **p-Value** | **Hodges-Lehmann** | **Lower CL** | **Upper CL** | **Difference Plot** |
| --- | --- | --- | --- | --- | --- | --- | --- | --- | --- |
| 3 | 2 | -1,54203 | 3,688211 | -0,41810 | 0,6759 | -0,001395 | -0,011423 | 0,0077482 |  |
| 4&5 | 3 | -2,39167 | 3,500000 | -0,68333 | 0,4944 | -0,003896 | -0,013527 | 0,0082139 |  |
| 4&5 | 2 | -4,25326 | 3,839063 | -1,10789 | 0,2679 | -0,005239 | -0,016196 | 0,0055773 |  |
| 2 | 1 | -4,51594 | 3,688211 | -1,22443 | 0,2208 | -0,005809 | -0,014231 | 0,0038740 |  |
| 3 | 1 | -5,33333 | 3,214550 | -1,65912 | 0,0971 | -0,007364 | -0,015539 | 0,0014194 |  |
| 4&5 | 1 | -6,47500 | 3,500000 | -1,85000 | 0,0643 | -0,009463 | -0,020091 | 0,0010002 |  |

**Oneway Analysis of original-glrlm-RunVariance By GrG**

**Quantiles**

| **Level** | **Minimum** | **10%** | **25%** | **Median** | **75%** | **90%** | **Maximum** |
| --- | --- | --- | --- | --- | --- | --- | --- |
| 1 | 0,01402 | 0,017506 | 0,022736 | 0,027637 | 0,043963 | 0,047739 | 0,047847 |
| 2 | 0,01422 | 0,017376 | 0,026882 | 0,039822 | 0,049406 | 0,054168 | 0,262458 |
| 3 | 0,02322 | 0,026545 | 0,031673 | 0,043825 | 0,044465 | 0,047429 | 0,048917 |
| 4&5 | 0,025085 | 0,027231 | 0,028952 | 0,039398 | 0,049164 | 0,054853 | 0,058952 |

**Nonparametric Comparisons For Each Pair Using Wilcoxon Method**

| **q*** | **Alpha** |
| --- | --- |
| 1,95996 | 0,05 |

| **Level** | **- Level** | **Score Mean Difference** | **Std Err Dif** | **Z** | **p-Value** | **Hodges-Lehmann** | **Lower CL** | **Upper CL** | **Difference Plot** |
| --- | --- | --- | --- | --- | --- | --- | --- | --- | --- |
| 4&5 | 1 | 6,241667 | 3,500000 | 1,783333 | 0,0745 | 0,0071930 | -0,000526 | 0,0150291 |  |
| 3 | 1 | 5,200000 | 3,214550 | 1,617645 | 0,1057 | 0,0049177 | -0,001900 | 0,0169405 |  |
| 2 | 1 | 4,075362 | 3,688211 | 1,104970 | 0,2692 | 0,0042101 | -0,004255 | 0,0146682 |  |
| 4&5 | 2 | 1,822826 | 3,839063 | 0,474810 | 0,6349 | 0,0017776 | -0,006521 | 0,0097615 |  |
| 3 | 2 | 1,762319 | 3,688211 | 0,477825 | 0,6328 | 0,0014837 | -0,006923 | 0,0091338 |  |
| 4&5 | 3 | 0,058333 | 3,500000 | 0,016667 | 0,9867 | 0,0002924 | -0,006564 | 0,0078814 |  |

**Oneway Analysis of original-glrlm-GrayLevelNonUniformity By GrG**

**Quantiles**

| **Level** | **Minimum** | **10%** | **25%** | **Median** | **75%** | **90%** | **Maximum** |
| --- | --- | --- | --- | --- | --- | --- | --- |
| 1 | 1,852781 | 2,201518 | 3,312501 | 5,623959 | 10,85998 | 15,79331 | 16,83839 |
| 2 | 1,961111 | 2,506486 | 3,933629 | 5,700868 | 12,82094 | 55,83482 | 98,60145 |
| 3 | 3,059173 | 3,934645 | 6,511811 | 10,27394 | 14,60733 | 40,292 | 44,90477 |
| 4&5 | 2,390432 | 3,401257 | 5,117483 | 13,00041 | 61,06747 | 133,7884 | 405,8616 |

**Nonparametric Comparisons For Each Pair Using Wilcoxon Method**

| **q*** | **Alpha** |
| --- | --- |
| 1,95996 | 0,05 |

| **Level** | **- Level** | **Score Mean Difference** | **Std Err Dif** | **Z** | **p-Value** | **Hodges-Lehmann** | **Lower CL** | **Upper CL** | **Difference Plot** |
| --- | --- | --- | --- | --- | --- | --- | --- | --- | --- |
| 4&5 | 1 | 7,875000 | 3,500000 | 2,250000 | 0,0244* | 6,736884 | 0,53765 | 19,63339 |  |
| 4&5 | 2 | 6,777174 | 3,839063 | 1,765320 | 0,0775 | 4,989872 | -0,35425 | 15,96409 |  |
| 3 | 1 | 6,400000 | 3,214550 | 1,990947 | 0,0465* | 3,843053 | -0,10348 | 8,96767 |  |
| 3 | 2 | 5,286957 | 3,688211 | 1,433475 | 0,1517 | 2,557183 | -1,71661 | 7,58624 |  |
| 2 | 1 | 2,973913 | 3,688211 | 0,806329 | 0,4201 | 1,070735 | -1,79576 | 4,39775 |  |
| 4&5 | 3 | 2,625000 | 3,500000 | 0,750000 | 0,4533 | 2,440882 | -3,66488 | 17,10369 |  |

**Oneway Analysis of original-glrlm-LongRunEmphasis By GrG**

**Quantiles**

| **Level** | **Minimum** | **10%** | **25%** | **Median** | **75%** | **90%** | **Maximum** |
| --- | --- | --- | --- | --- | --- | --- | --- |
| 1 | 1,045659 | 1,052746 | 1,069878 | 1,086081 | 1,138576 | 1,143904 | 1,144727 |
| 2 | 1,044389 | 1,054782 | 1,074938 | 1,118126 | 1,143111 | 1,159115 | 1,936815 |
| 3 | 1,07357 | 1,082351 | 1,096335 | 1,127758 | 1,135258 | 1,141105 | 1,145198 |
| 4&5 | 1,073859 | 1,080853 | 1,090711 | 1,113149 | 1,143081 | 1,160162 | 1,170041 |

**Nonparametric Comparisons For Each Pair Using Wilcoxon Method**

| **q*** | **Alpha** |
| --- | --- |
| 1,95996 | 0,05 |

| **Level** | **- Level** | **Score Mean Difference** | **Std Err Dif** | **Z** | **p-Value** | **Hodges-Lehmann** | **Lower CL** | **Upper CL** | **Difference Plot** |
| --- | --- | --- | --- | --- | --- | --- | --- | --- | --- |
| 4&5 | 1 | 6,008333 | 3,500000 | 1,716667 | 0,0860 | 0,0189507 | -0,004946 | 0,0423203 |  |
| 3 | 1 | 4,400000 | 3,214550 | 1,368776 | 0,1711 | 0,0172659 | -0,007020 | 0,0520879 |  |
| 2 | 1 | 3,855072 | 3,688211 | 1,045242 | 0,2959 | 0,0117252 | -0,013338 | 0,0445976 |  |
| 3 | 2 | 1,872464 | 3,688211 | 0,507689 | 0,6117 | 0,0054887 | -0,018350 | 0,0289627 |  |
| 4&5 | 2 | 1,729348 | 3,839063 | 0,450461 | 0,6524 | 0,0057307 | -0,018810 | 0,0280592 |  |
| 4&5 | 3 | 0,058333 | 3,500000 | 0,016667 | 0,9867 | 0,0000994 | -0,021734 | 0,0188947 |  |

**Oneway Analysis of original-glrlm-ShortRunHighGrayLevelEmphasis By GrG**

**Quantiles**

| **Level** | **Minimum** | **10%** | **25%** | **Median** | **75%** | **90%** | **Maximum** |
| --- | --- | --- | --- | --- | --- | --- | --- |
| 1 | 127,8 | 157,2254 | 209,7197 | 292,955 | 579,728 | 923,9376 | 1098,802 |
| 2 | 3,600202 | 141,4281 | 298,5877 | 477,8597 | 855,721 | 966,8926 | 1018,738 |
| 3 | 141,9518 | 161,9342 | 242,9934 | 342,0569 | 514,7705 | 934,2901 | 1074,721 |
| 4&5 | 164,368 | 225,6907 | 303,3014 | 488,037 | 765,8145 | 1317,455 | 2018,638 |

**Nonparametric Comparisons For Each Pair Using Wilcoxon Method**

| **q*** | **Alpha** |
| --- | --- |
| 1,95996 | 0,05 |

| **Level** | **- Level** | **Score Mean Difference** | **Std Err Dif** | **Z** | **p-Value** | **Hodges-Lehmann** | **Lower CL** | **Upper CL** | **Difference Plot** |
| --- | --- | --- | --- | --- | --- | --- | --- | --- | --- |
| 4&5 | 1 | 6,82500 | 3,500000 | 1,95000 | 0,0512 | 156,266 | -1,096 | 416,6028 |  |
| 4&5 | 3 | 6,00833 | 3,500000 | 1,71667 | 0,0860 | 135,933 | -29,699 | 386,5131 |  |
| 2 | 1 | 5,50725 | 3,688211 | 1,49320 | 0,1354 | 134,027 | -57,257 | 334,6844 |  |
| 4&5 | 2 | 1,35543 | 3,839063 | 0,35306 | 0,7240 | 39,898 | -178,135 | 252,4930 |  |
| 3 | 1 | 0,80000 | 3,214550 | 0,24887 | 0,8035 | 24,232 | -136,027 | 158,4470 |  |
| 3 | 2 | -5,06667 | 3,688211 | -1,37375 | 0,1695 | -127,573 | -335,908 | 56,6647 |  |

**Oneway Analysis of original-glrlm-RunLengthNonUniformity By GrG**

**Quantiles**

| **Level** | **Minimum** | **10%** | **25%** | **Median** | **75%** | **90%** | **Maximum** |
| --- | --- | --- | --- | --- | --- | --- | --- |
| 1 | 25,46438 | 38,18842 | 48,73383 | 91,45249 | 179,0931 | 326,5458 | 370,1581 |
| 2 | 15,28333 | 28,01744 | 51,75183 | 113,07 | 281,2341 | 1267,698 | 2537,388 |
| 3 | 30,71371 | 62,82845 | 101,2292 | 233,187 | 345,1499 | 907,8399 | 1043,539 |
| 4&5 | 25,81212 | 53,12229 | 82,60127 | 318,2347 | 1564,302 | 3042,775 | 14563,78 |

**Nonparametric Comparisons For Each Pair Using Wilcoxon Method**

| **q*** | **Alpha** |
| --- | --- |
| 1,95996 | 0,05 |

| **Level** | **- Level** | **Score Mean Difference** | **Std Err Dif** | **Z** | **p-Value** | **Hodges-Lehmann** | **Lower CL** | **Upper CL** | **Difference Plot** |
| --- | --- | --- | --- | --- | --- | --- | --- | --- | --- |
| 4&5 | 1 | 9,275000 | 3,500000 | 2,650000 | 0,0080* | 212,5019 | 26,2404 | 366,4658 |  |
| 4&5 | 2 | 7,244565 | 3,839063 | 1,887066 | 0,0592 | 172,7383 | -1,5530 | 326,2248 |  |
| 3 | 1 | 6,933333 | 3,214550 | 2,156860 | 0,0310* | 91,0289 | 5,2493 | 207,7226 |  |
| 3 | 2 | 4,846377 | 3,688211 | 1,314018 | 0,1888 | 63,9158 | -37,5726 | 167,4301 |  |
| 4&5 | 3 | 3,675000 | 3,500000 | 1,050000 | 0,2937 | 93,5811 | -69,9619 | 329,2576 |  |
| 2 | 1 | 2,202899 | 3,688211 | 0,597281 | 0,5503 | 20,5300 | -39,7203 | 103,3648 |  |

**Oneway Analysis of original-glrlm-ShortRunEmphasis By GrG**

**Quantiles**

| **Level** | **Minimum** | **10%** | **25%** | **Median** | **75%** | **90%** | **Maximum** |
| --- | --- | --- | --- | --- | --- | --- | --- |
| 1 | 0,965583 | 0,965867 | 0,966614 | 0,97848 | 0,983935 | 0,987928 | 0,988585 |
| 2 | 0,827472 | 0,963314 | 0,967963 | 0,972233 | 0,981266 | 0,986305 | 0,988903 |
| 3 | 0,965921 | 0,96606 | 0,967981 | 0,970723 | 0,97673 | 0,979626 | 0,981607 |
| 4&5 | 0,961811 | 0,963387 | 0,967181 | 0,972815 | 0,979339 | 0,980856 | 0,982617 |

**Nonparametric Comparisons For Each Pair Using Wilcoxon Method**

| **q*** | **Alpha** |
| --- | --- |
| 1,95996 | 0,05 |

| **Level** | **- Level** | **Score Mean Difference** | **Std Err Dif** | **Z** | **p-Value** | **Hodges-Lehmann** | **Lower CL** | **Upper CL** | **Difference Plot** |
| --- | --- | --- | --- | --- | --- | --- | --- | --- | --- |
| 4&5 | 3 | 0,87500 | 3,500000 | 0,25000 | 0,8026 | 0,000499 | -0,003864 | 0,0052484 |  |
| 4&5 | 2 | -1,72935 | 3,839063 | -0,45046 | 0,6524 | -0,001236 | -0,006181 | 0,0040161 |  |
| 3 | 2 | -2,09275 | 3,688211 | -0,56742 | 0,5704 | -0,001613 | -0,006513 | 0,0034037 |  |
| 2 | 1 | -3,63478 | 3,688211 | -0,98551 | 0,3244 | -0,002440 | -0,009537 | 0,0036504 |  |
| 3 | 1 | -4,40000 | 3,214550 | -1,36878 | 0,1711 | -0,004525 | -0,011384 | 0,0019242 |  |
| 4&5 | 1 | -5,07500 | 3,500000 | -1,45000 | 0,1471 | -0,003794 | -0,010007 | 0,0017342 |  |

**Oneway Analysis of original-glrlm-LongRunHighGrayLevelEmphasis By GrG**

**Quantiles**

| **Level** | **Minimum** | **10%** | **25%** | **Median** | **75%** | **90%** | **Maximum** |
| --- | --- | --- | --- | --- | --- | --- | --- |
| 1 | 135,5997 | 167,2135 | 243,3557 | 313,307 | 655,6974 | 1007,492 | 1193,606 |
| 2 | 8,631241 | 159,4088 | 337,2338 | 537,2158 | 913,5698 | 1146,009 | 1189,177 |
| 3 | 164,6487 | 191,0836 | 272,8179 | 372,1654 | 577,785 | 1050,366 | 1251,719 |
| 4&5 | 187,5038 | 263,0949 | 334,5637 | 582,4032 | 891,0106 | 1447,573 | 2335,755 |

**Nonparametric Comparisons For Each Pair Using Wilcoxon Method**

| **q*** | **Alpha** |
| --- | --- |
| 1,95996 | 0,05 |

| **Level** | **- Level** | **Score Mean Difference** | **Std Err Dif** | **Z** | **p-Value** | **Hodges-Lehmann** | **Lower CL** | **Upper CL** | **Difference Plot** |
| --- | --- | --- | --- | --- | --- | --- | --- | --- | --- |
| 4&5 | 1 | 7,29167 | 3,500000 | 2,08333 | 0,0372* | 182,070 | 3,754 | 476,3922 |  |
| 4&5 | 3 | 6,12500 | 3,500000 | 1,75000 | 0,0801 | 153,301 | -23,322 | 446,1296 |  |
| 2 | 1 | 5,61739 | 3,688211 | 1,52307 | 0,1277 | 151,609 | -57,533 | 384,2668 |  |
| 4&5 | 2 | 1,91630 | 3,839063 | 0,49916 | 0,6177 | 49,473 | -179,895 | 285,8277 |  |
| 3 | 1 | 1,20000 | 3,214550 | 0,37330 | 0,7089 | 31,432 | -138,362 | 181,9987 |  |
| 3 | 2 | -4,84638 | 3,688211 | -1,31402 | 0,1888 | -130,769 | -362,334 | 63,7060 |  |

**Oneway Analysis of original-glrlm-RunPercentage By GrG**

**Quantiles**

| **Level** | **Minimum** | **10%** | **25%** | **Median** | **75%** | **90%** | **Maximum** |
| --- | --- | --- | --- | --- | --- | --- | --- |
| 1 | 0,955347 | 0,955765 | 0,957066 | 0,972424 | 0,978507 | 0,983987 | 0,985871 |
| 2 | 0,810147 | 0,951464 | 0,956769 | 0,963801 | 0,975962 | 0,982576 | 0,985755 |
| 3 | 0,955369 | 0,956119 | 0,95781 | 0,96085 | 0,969508 | 0,973777 | 0,97669 |
| 4&5 | 0,948928 | 0,951322 | 0,956287 | 0,964673 | 0,972184 | 0,974558 | 0,97683 |

**Nonparametric Comparisons For Each Pair Using Wilcoxon Method**

| **q*** | **Alpha** |
| --- | --- |
| 1,95996 | 0,05 |

| **Level** | **- Level** | **Score Mean Difference** | **Std Err Dif** | **Z** | **p-Value** | **Hodges-Lehmann** | **Lower CL** | **Upper CL** | **Difference Plot** |
| --- | --- | --- | --- | --- | --- | --- | --- | --- | --- |
| 4&5 | 3 | 0,29167 | 3,500000 | 0,08333 | 0,9336 | 0,000369 | -0,005570 | 0,0063896 |  |
| 4&5 | 2 | -1,86957 | 3,838918 | -0,48700 | 0,6263 | -0,001779 | -0,007933 | 0,0055099 |  |
| 3 | 2 | -2,03768 | 3,688009 | -0,55252 | 0,5806 | -0,001738 | -0,008426 | 0,0049023 |  |
| 2 | 1 | -3,85507 | 3,688211 | -1,04524 | 0,2959 | -0,003167 | -0,012968 | 0,0045976 |  |
| 3 | 1 | -4,40000 | 3,214550 | -1,36878 | 0,1711 | -0,005410 | -0,015291 | 0,0021789 |  |
| 4&5 | 1 | -5,54167 | 3,500000 | -1,58333 | 0,1133 | -0,005666 | -0,013103 | 0,0015008 |  |

**Oneway Analysis of original-glrlm-LongRunLowGrayLevelEmphasis By GrG**

**Quantiles**

| **Level** | **Minimum** | **10%** | **25%** | **Median** | **75%** | **90%** | **Maximum** |
| --- | --- | --- | --- | --- | --- | --- | --- |
| 1 | 0,005548 | 0,005969 | 0,011505 | 0,020639 | 0,045456 | 0,068249 | 0,09267 |
| 2 | 0,002248 | 0,002597 | 0,009141 | 0,015393 | 0,035204 | 0,138524 | 0,856265 |
| 3 | 0,002412 | 0,004979 | 0,010401 | 0,017117 | 0,030962 | 0,043676 | 0,052606 |
| 4&5 | 0,001105 | 0,00228 | 0,004309 | 0,008998 | 0,020677 | 0,052894 | 0,078846 |

**Nonparametric Comparisons For Each Pair Using Wilcoxon Method**

| **q*** | **Alpha** |
| --- | --- |
| 1,95996 | 0,05 |

| **Level** | **- Level** | **Score Mean Difference** | **Std Err Dif** | **Z** | **p-Value** | **Hodges-Lehmann** | **Lower CL** | **Upper CL** | **Difference Plot** |
| --- | --- | --- | --- | --- | --- | --- | --- | --- | --- |
| 3 | 2 | -0,11014 | 3,688211 | -0,02986 | 0,9762 | -0,000160 | -0,012246 | 0,009397 |  |
| 3 | 1 | -2,93333 | 3,214550 | -0,91252 | 0,3615 | -0,005710 | -0,022427 | 0,004900 |  |
| 2 | 1 | -3,74493 | 3,688211 | -1,01538 | 0,3099 | -0,005202 | -0,018391 | 0,007364 |  |
| 4&5 | 2 | -4,90761 | 3,839063 | -1,27833 | 0,2011 | -0,006684 | -0,015554 | 0,001951 |  |
| 4&5 | 3 | -5,77500 | 3,500000 | -1,65000 | 0,0989 | -0,006275 | -0,013864 | 0,002175 |  |
| 4&5 | 1 | -7,52500 | 3,500000 | -2,15000 | 0,0316* | -0,011240 | -0,029683 | -0,000920 |  |

**Oneway Analysis of original-glrlm-RunEntropy By GrG**

**Quantiles**

| **Level** | **Minimum** | **10%** | **25%** | **Median** | **75%** | **90%** | **Maximum** |
| --- | --- | --- | --- | --- | --- | --- | --- |
| 1 | 3,971147 | 4,021416 | 4,392545 | 4,553553 | 4,726679 | 5,060206 | 5,128723 |
| 2 | 2,390519 | 3,346317 | 4,372606 | 4,823381 | 5,083307 | 5,329032 | 5,570541 |
| 3 | 3,783441 | 4,021137 | 4,541921 | 4,804145 | 5,131884 | 5,200237 | 5,219779 |
| 4&5 | 3,770475 | 4,169338 | 4,565433 | 4,814961 | 5,418857 | 5,67542 | 5,843222 |

**Nonparametric Comparisons For Each Pair Using Wilcoxon Method**

| **q*** | **Alpha** |
| --- | --- |
| 1,95996 | 0,05 |

| **Level** | **- Level** | **Score Mean Difference** | **Std Err Dif** | **Z** | **p-Value** | **Hodges-Lehmann** | **Lower CL** | **Upper CL** | **Difference Plot** |
| --- | --- | --- | --- | --- | --- | --- | --- | --- | --- |
| 4&5 | 1 | 7,991667 | 3,500000 | 2,283333 | 0,0224* | 0,3499380 | 0,041010 | 0,7270143 |  |
| 3 | 1 | 6,133333 | 3,214550 | 1,907991 | 0,0564 | 0,2392317 | -0,013894 | 0,5250498 |  |
| 4&5 | 2 | 4,533696 | 3,839063 | 1,180938 | 0,2376 | 0,2136188 | -0,154775 | 0,6072096 |  |
| 2 | 1 | 4,185507 | 3,688211 | 1,134834 | 0,2564 | 0,2048226 | -0,146677 | 0,5183520 |  |
| 4&5 | 3 | 2,391667 | 3,500000 | 0,683333 | 0,4944 | 0,1615799 | -0,228229 | 0,5561500 |  |
| 3 | 2 | 1,211594 | 3,688211 | 0,328505 | 0,7425 | 0,0540455 | -0,263951 | 0,3963975 |  |

**Oneway Analysis of original-glrlm-HighGrayLevelRunEmphasis By GrG**

**Quantiles**

| **Level** | **Minimum** | **10%** | **25%** | **Median** | **75%** | **90%** | **Maximum** |
| --- | --- | --- | --- | --- | --- | --- | --- |
| 1 | 129,3599 | 159,223 | 216,1507 | 297,0254 | 594,2131 | 939,9789 | 1116,089 |
| 2 | 4,424969 | 144,7693 | 306,3169 | 488,9144 | 867,2908 | 1001,373 | 1049,44 |
| 3 | 146,3938 | 166,9541 | 248,8145 | 350,752 | 526,541 | 956,8557 | 1108,743 |
| 4&5 | 168,6595 | 232,7029 | 309,4441 | 505,5095 | 789,6467 | 1341,991 | 2077,596 |

**Nonparametric Comparisons For Each Pair Using Wilcoxon Method**

| **q*** | **Alpha** |
| --- | --- |
| 1,95996 | 0,05 |

| **Level** | **- Level** | **Score Mean Difference** | **Std Err Dif** | **Z** | **p-Value** | **Hodges-Lehmann** | **Lower CL** | **Upper CL** | **Difference Plot** |
| --- | --- | --- | --- | --- | --- | --- | --- | --- | --- |
| 4&5 | 1 | 6,94167 | 3,500000 | 1,98333 | 0,0473* | 160,533 | -0,207 | 423,2779 |  |
| 4&5 | 3 | 6,00833 | 3,500000 | 1,71667 | 0,0860 | 138,321 | -28,602 | 392,4759 |  |
| 2 | 1 | 5,61739 | 3,688211 | 1,52307 | 0,1277 | 134,070 | -59,538 | 345,0106 |  |
| 4&5 | 2 | 1,35543 | 3,839063 | 0,35306 | 0,7240 | 43,546 | -179,088 | 257,9889 |  |
| 3 | 1 | 1,06667 | 3,214550 | 0,33182 | 0,7400 | 25,373 | -136,646 | 163,1628 |  |
| 3 | 2 | -5,06667 | 3,688211 | -1,37375 | 0,1695 | -125,656 | -342,521 | 57,2988 |  |

**Oneway Analysis of original-glrlm-RunLengthNonUniformityNormalized By GrG**

**Quantiles**

| **Level** | **Minimum** | **10%** | **25%** | **Median** | **75%** | **90%** | **Maximum** |
| --- | --- | --- | --- | --- | --- | --- | --- |
| 1 | 0,913688 | 0,914878 | 0,915868 | 0,944951 | 0,960341 | 0,969853 | 0,971959 |
| 2 | 0,678678 | 0,907718 | 0,919202 | 0,932171 | 0,952006 | 0,965248 | 0,971559 |
| 3 | 0,914416 | 0,914641 | 0,919005 | 0,925704 | 0,940366 | 0,948001 | 0,953561 |
| 4&5 | 0,90445 | 0,908046 | 0,917055 | 0,930858 | 0,947595 | 0,950931 | 0,95545 |

**Nonparametric Comparisons For Each Pair Using Wilcoxon Method**

| **q*** | **Alpha** |
| --- | --- |
| 1,95996 | 0,05 |

| **Level** | **- Level** | **Score Mean Difference** | **Std Err Dif** | **Z** | **p-Value** | **Hodges-Lehmann** | **Lower CL** | **Upper CL** | **Difference Plot** |
| --- | --- | --- | --- | --- | --- | --- | --- | --- | --- |
| 4&5 | 3 | 0,87500 | 3,500000 | 0,25000 | 0,8026 | 0,001490 | -0,009410 | 0,0126981 |  |
| 4&5 | 2 | -1,54239 | 3,839063 | -0,40176 | 0,6879 | -0,003278 | -0,015367 | 0,0098106 |  |
| 3 | 2 | -2,53333 | 3,688211 | -0,68687 | 0,4922 | -0,004211 | -0,016795 | 0,0085670 |  |
| 2 | 1 | -3,52464 | 3,688211 | -0,95565 | 0,3392 | -0,006067 | -0,023550 | 0,0093291 |  |
| 3 | 1 | -4,40000 | 3,214550 | -1,36878 | 0,1711 | -0,010682 | -0,028083 | 0,0041343 |  |
| 4&5 | 1 | -4,84167 | 3,500000 | -1,38333 | 0,1666 | -0,009688 | -0,025440 | 0,0038382 |  |

**Oneway Analysis of original-glszm-GrayLevelVariance By GrG**

**Quantiles**

| **Level** | **Minimum** | **10%** | **25%** | **Median** | **75%** | **90%** | **Maximum** |
| --- | --- | --- | --- | --- | --- | --- | --- |
| 1 | 26,7275 | 29,71856 | 37,05202 | 49,9386 | 74,80966 | 105,9101 | 138,3861 |
| 2 | 1,25 | 25,14577 | 43,46314 | 62,74858 | 95,88572 | 126,2046 | 179,7537 |
| 3 | 28,6263 | 28,88726 | 32,02778 | 55,4316 | 70,39816 | 99,03577 | 106,7683 |
| 4&5 | 23,72222 | 36,94285 | 43,95263 | 64,75102 | 114,7624 | 184,7927 | 193,0242 |

**Nonparametric Comparisons For Each Pair Using Wilcoxon Method**

| **q*** | **Alpha** |
| --- | --- |
| 1,95996 | 0,05 |

| **Level** | **- Level** | **Score Mean Difference** | **Std Err Dif** | **Z** | **p-Value** | **Hodges-Lehmann** | **Lower CL** | **Upper CL** | **Difference Plot** |
| --- | --- | --- | --- | --- | --- | --- | --- | --- | --- |
| 4&5 | 1 | 4,95833 | 3,500000 | 1,41667 | 0,1566 | 14,0705 | -6,4514 | 44,28627 |  |
| 4&5 | 3 | 4,72500 | 3,500000 | 1,35000 | 0,1770 | 13,0657 | -8,3056 | 43,63370 |  |
| 2 | 1 | 4,51594 | 3,688211 | 1,22443 | 0,2208 | 13,0790 | -9,5513 | 32,91594 |  |
| 4&5 | 2 | 1,07500 | 3,839063 | 0,28002 | 0,7795 | 3,7669 | -18,7225 | 31,90557 |  |
| 3 | 1 | 0,26667 | 3,214550 | 0,08296 | 0,9339 | 0,3938 | -17,9108 | 20,09064 |  |
| 3 | 2 | -3,74493 | 3,688211 | -1,01538 | 0,3099 | -11,1092 | -32,6425 | 7,91697 |  |

**Oneway Analysis of original-glszm-ZoneVariance By GrG**

**Quantiles**

| **Level** | **Minimum** | **10%** | **25%** | **Median** | **75%** | **90%** | **Maximum** |
| --- | --- | --- | --- | --- | --- | --- | --- |
| 1 | 0,224375 | 0,28625 | 0,482299 | 1,398543 | 2,467631 | 2,659789 | 2,736455 |
| 2 | 0,177515 | 0,350921 | 0,662132 | 1,545533 | 3,083918 | 5,179978 | 48,6875 |
| 3 | 0,548701 | 0,865271 | 1,272638 | 1,526042 | 2,680367 | 3,037156 | 3,407456 |
| 4&5 | 0,569722 | 0,674 | 0,831947 | 1,683239 | 3,255344 | 6,512293 | 6,727899 |

**Nonparametric Comparisons For Each Pair Using Wilcoxon Method**

| **q*** | **Alpha** |
| --- | --- |
| 1,95996 | 0,05 |

| **Level** | **- Level** | **Score Mean Difference** | **Std Err Dif** | **Z** | **p-Value** | **Hodges-Lehmann** | **Lower CL** | **Upper CL** | **Difference Plot** |
| --- | --- | --- | --- | --- | --- | --- | --- | --- | --- |
| 4&5 | 1 | 6,24167 | 3,500000 | 1,78333 | 0,0745 | 0,552087 | -0,055217 | 1,715225 |  |
| 3 | 1 | 5,73333 | 3,214550 | 1,78356 | 0,0745 | 0,671000 | -0,122128 | 1,202727 |  |
| 2 | 1 | 3,41449 | 3,688211 | 0,92579 | 0,3546 | 0,286045 | -0,423548 | 1,086074 |  |
| 4&5 | 2 | 2,94457 | 3,839063 | 0,76700 | 0,4431 | 0,273384 | -0,437842 | 1,167482 |  |
| 3 | 2 | 2,42319 | 3,688211 | 0,65701 | 0,5112 | 0,307205 | -0,449231 | 1,016918 |  |
| 4&5 | 3 | -0,05833 | 3,500000 | -0,01667 | 0,9867 | -0,021742 | -0,694441 | 1,201759 |  |

**Oneway Analysis of original-glszm-GrayLevelNonUniformityNormalized By GrG**

**Quantiles**

| **Level** | **Minimum** | **10%** | **25%** | **Median** | **75%** | **90%** | **Maximum** |
| --- | --- | --- | --- | --- | --- | --- | --- |
| 1 | 0,036209 | 0,036673 | 0,045557 | 0,04873 | 0,055884 | 0,070255 | 0,070637 |
| 2 | 0,023951 | 0,031631 | 0,033002 | 0,042146 | 0,05272 | 0,099589 | 0,25 |
| 3 | 0,031823 | 0,031917 | 0,034707 | 0,041724 | 0,053243 | 0,068447 | 0,070295 |
| 4&5 | 0,022073 | 0,023114 | 0,028942 | 0,041624 | 0,050556 | 0,061719 | 0,08 |

**Nonparametric Comparisons For Each Pair Using Wilcoxon Method**

| **q*** | **Alpha** |
| --- | --- |
| 1,95996 | 0,05 |

| **Level** | **- Level** | **Score Mean Difference** | **Std Err Dif** | **Z** | **p-Value** | **Hodges-Lehmann** | **Lower CL** | **Upper CL** | **Difference Plot** |
| --- | --- | --- | --- | --- | --- | --- | --- | --- | --- |
| 3 | 2 | -0,22029 | 3,688211 | -0,05973 | 0,9524 | -0,000337 | -0,010166 | 0,008397 |  |
| 4&5 | 3 | -2,74167 | 3,499755 | -0,78339 | 0,4334 | -0,003636 | -0,013061 | 0,006292 |  |
| 4&5 | 2 | -4,15978 | 3,838918 | -1,08358 | 0,2786 | -0,004292 | -0,013827 | 0,004345 |  |
| 2 | 1 | -5,06667 | 3,688211 | -1,37375 | 0,1695 | -0,006646 | -0,014790 | 0,003670 |  |
| 3 | 1 | -6,13333 | 3,214550 | -1,90799 | 0,0564 | -0,006794 | -0,015084 | 0,001093 |  |
| 4&5 | 1 | -7,29167 | 3,499755 | -2,08348 | 0,0372* | -0,010346 | -0,019444 | -0,001197 |  |

**Oneway Analysis of original-glszm-SizeZoneNonUniformityNormalized By GrG**

**Quantiles**

| **Level** | **Minimum** | **10%** | **25%** | **Median** | **75%** | **90%** | **Maximum** |
| --- | --- | --- | --- | --- | --- | --- | --- |
| 1 | 0,408173 | 0,413286 | 0,468751 | 0,552632 | 0,647059 | 0,671685 | 0,672337 |
| 2 | 0,25 | 0,387402 | 0,439242 | 0,486917 | 0,63179 | 0,688539 | 0,693762 |
| 3 | 0,407407 | 0,408427 | 0,467538 | 0,49562 | 0,535108 | 0,57815 | 0,589295 |
| 4&5 | 0,440442 | 0,448127 | 0,47087 | 0,496359 | 0,561938 | 0,647111 | 0,69375 |

**Nonparametric Comparisons For Each Pair Using Wilcoxon Method**

| **q*** | **Alpha** |
| --- | --- |
| 1,95996 | 0,05 |

| **Level** | **- Level** | **Score Mean Difference** | **Std Err Dif** | **Z** | **p-Value** | **Hodges-Lehmann** | **Lower CL** | **Upper CL** | **Difference Plot** |
| --- | --- | --- | --- | --- | --- | --- | --- | --- | --- |
| 4&5 | 2 | 2,10326 | 3,839063 | 0,54786 | 0,5838 | 0,010456 | -0,042335 | 0,0569579 |  |
| 4&5 | 3 | 0,87500 | 3,500000 | 0,25000 | 0,8026 | 0,002573 | -0,036689 | 0,0577491 |  |
| 3 | 2 | 0,11014 | 3,688211 | 0,02986 | 0,9762 | 0,004229 | -0,069723 | 0,0545747 |  |
| 4&5 | 1 | -2,97500 | 3,500000 | -0,85000 | 0,3953 | -0,022719 | -0,098784 | 0,0321675 |  |
| 2 | 1 | -3,52464 | 3,688211 | -0,95565 | 0,3392 | -0,028219 | -0,115758 | 0,0310690 |  |
| 3 | 1 | -4,13333 | 3,214550 | -1,28582 | 0,1985 | -0,045633 | -0,122347 | 0,0240081 |  |

**Oneway Analysis of original-glszm-SizeZoneNonUniformity By GrG**

**Quantiles**

| **Level** | **Minimum** | **10%** | **25%** | **Median** | **75%** | **90%** | **Maximum** |
| --- | --- | --- | --- | --- | --- | --- | --- |
| 1 | 11,1 | 17,04 | 26,85 | 33 | 69,92308 | 106,1966 | 116,7189 |
| 2 | 1 | 7,74332 | 25,91525 | 42,14943 | 113,7222 | 327,1653 | 703,8592 |
| 3 | 11,19048 | 17,67619 | 40,91667 | 62,18421 | 105,4162 | 287,4516 | 335,5347 |
| 4&5 | 13,1 | 24,225 | 33,74323 | 116,175 | 447,7035 | 900,5825 | 4397,37 |

**Nonparametric Comparisons For Each Pair Using Wilcoxon Method**

| **q*** | **Alpha** |
| --- | --- |
| 1,95996 | 0,05 |

| **Level** | **- Level** | **Score Mean Difference** | **Std Err Dif** | **Z** | **p-Value** | **Hodges-Lehmann** | **Lower CL** | **Upper CL** | **Difference Plot** |
| --- | --- | --- | --- | --- | --- | --- | --- | --- | --- |
| 4&5 | 1 | 9,741667 | 3,500000 | 2,783333 | 0,0054* | 68,93634 | 7,5457 | 120,7513 |  |
| 4&5 | 2 | 7,711957 | 3,839063 | 2,008812 | 0,0446* | 64,02270 | 1,6574 | 110,8339 |  |
| 3 | 1 | 6,133333 | 3,214550 | 1,907991 | 0,0564 | 26,98582 | -4,3793 | 64,0071 |  |
| 3 | 2 | 4,846377 | 3,688211 | 1,314018 | 0,1888 | 18,39850 | -14,6410 | 53,7996 |  |
| 4&5 | 3 | 4,491667 | 3,500000 | 1,283333 | 0,1994 | 35,49173 | -21,6464 | 104,4034 |  |
| 2 | 1 | 1,101449 | 3,688211 | 0,298641 | 0,7652 | 3,51364 | -17,5385 | 28,6695 |  |

**Oneway Analysis of original-glszm-GrayLevelNonUniformity By GrG**

**Quantiles**

| **Level** | **Minimum** | **10%** | **25%** | **Median** | **75%** | **90%** | **Maximum** |
| --- | --- | --- | --- | --- | --- | --- | --- |
| 1 | 1,4 | 1,76 | 2,387755 | 3,151515 | 5,827586 | 9,017719 | 9,020202 |
| 2 | 1 | 1,487449 | 1,863636 | 3,462963 | 6,71123 | 26,69414 | 48,78424 |
| 3 | 1,47619 | 2,44118 | 3,77551 | 5,693182 | 9,242215 | 20,44314 | 23,49631 |
| 4&5 | 1,6 | 2,077203 | 3,15 | 7,146229 | 32,31787 | 60,23769 | 199,0071 |

**Nonparametric Comparisons For Each Pair Using Wilcoxon Method**

| **q*** | **Alpha** |
| --- | --- |
| 1,95996 | 0,05 |

| **Level** | **- Level** | **Score Mean Difference** | **Std Err Dif** | **Z** | **p-Value** | **Hodges-Lehmann** | **Lower CL** | **Upper CL** | **Difference Plot** |
| --- | --- | --- | --- | --- | --- | --- | --- | --- | --- |
| 4&5 | 1 | 8,108333 | 3,500000 | 2,316667 | 0,0205* | 3,476934 | 0,31579 | 9,331346 |  |
| 4&5 | 2 | 8,085870 | 3,839063 | 2,106209 | 0,0352* | 3,420713 | 0,18636 | 8,687355 |  |
| 3 | 1 | 6,666667 | 3,214550 | 2,073903 | 0,0381* | 2,028912 | 0,11677 | 4,342105 |  |
| 3 | 2 | 6,498551 | 3,688211 | 1,761979 | 0,0781 | 1,911874 | -0,30952 | 3,907468 |  |
| 4&5 | 3 | 2,741667 | 3,500000 | 0,783333 | 0,4334 | 1,627967 | -1,80508 | 8,089471 |  |
| 2 | 1 | 0,220290 | 3,688211 | 0,059728 | 0,9524 | 0,061538 | -1,45009 | 1,637681 |  |

**Oneway Analysis of original-glszm-LargeAreaEmphasis By GrG**

**Quantiles**

| **Level** | **Minimum** | **10%** | **25%** | **Median** | **75%** | **90%** | **Maximum** |
| --- | --- | --- | --- | --- | --- | --- | --- |
| 1 | 1,725 | 1,843846 | 2,408163 | 3,45098 | 5,616162 | 6,164242 | 6,287879 |
| 2 | 1,692308 | 1,950303 | 2,869565 | 4,421053 | 6,272566 | 9,047368 | 186,75 |
| 3 | 2,492958 | 2,892638 | 3,643599 | 4,611111 | 6,085714 | 6,509291 | 6,592308 |
| 4&5 | 2,483333 | 2,719831 | 2,945098 | 4,372342 | 6,460015 | 10,03037 | 10,8723 |

**Nonparametric Comparisons For Each Pair Using Wilcoxon Method**

| **q*** | **Alpha** |
| --- | --- |
| 1,95996 | 0,05 |

| **Level** | **- Level** | **Score Mean Difference** | **Std Err Dif** | **Z** | **p-Value** | **Hodges-Lehmann** | **Lower CL** | **Upper CL** | **Difference Plot** |
| --- | --- | --- | --- | --- | --- | --- | --- | --- | --- |
| 4&5 | 1 | 6,59167 | 3,500000 | 1,88333 | 0,0597 | 0,97511 | -0,13060 | 2,709189 |  |
| 3 | 1 | 5,86667 | 3,214550 | 1,82503 | 0,0680 | 1,12406 | -0,14593 | 2,202948 |  |
| 2 | 1 | 4,07536 | 3,688211 | 1,10497 | 0,2692 | 0,59322 | -0,65822 | 2,039635 |  |
| 3 | 2 | 2,53333 | 3,688211 | 0,68687 | 0,4922 | 0,32807 | -0,81877 | 1,615155 |  |
| 4&5 | 2 | 2,47717 | 3,839063 | 0,64525 | 0,5188 | 0,39804 | -0,92319 | 1,773446 |  |
| 4&5 | 3 | -0,75833 | 3,500000 | -0,21667 | 0,8285 | -0,10552 | -1,16027 | 1,575284 |  |

**Oneway Analysis of original-glszm-SmallAreaHighGrayLevelEmphasis By GrG**

**Quantiles**

| **Level** | **Minimum** | **10%** | **25%** | **Median** | **75%** | **90%** | **Maximum** |
| --- | --- | --- | --- | --- | --- | --- | --- |
| 1 | 120,3321 | 139,752 | 167,2847 | 234,0313 | 427,852 | 766,4076 | 934,0037 |
| 2 | 4,022206 | 86,63895 | 222,9662 | 393,3949 | 639,9086 | 739,9083 | 804,1994 |
| 3 | 87,79946 | 109,5437 | 182,4144 | 249,1482 | 403,1853 | 775,9389 | 825,7027 |
| 4&5 | 111,0974 | 186,8561 | 235,997 | 375,6102 | 584,1715 | 1140,007 | 1766,947 |

**Nonparametric Comparisons For Each Pair Using Wilcoxon Method**

| **q*** | **Alpha** |
| --- | --- |
| 1,95996 | 0,05 |

| **Level** | **- Level** | **Score Mean Difference** | **Std Err Dif** | **Z** | **p-Value** | **Hodges-Lehmann** | **Lower CL** | **Upper CL** | **Difference Plot** |
| --- | --- | --- | --- | --- | --- | --- | --- | --- | --- |
| 4&5 | 1 | 6,94167 | 3,500000 | 1,98333 | 0,0473* | 118,759 | -1,772 | 337,5263 |  |
| 4&5 | 3 | 5,89167 | 3,500000 | 1,68333 | 0,0923 | 118,305 | -26,354 | 322,8473 |  |
| 2 | 1 | 5,50725 | 3,688211 | 1,49320 | 0,1354 | 113,906 | -50,718 | 288,5680 |  |
| 4&5 | 2 | 1,16848 | 3,839063 | 0,30437 | 0,7608 | 24,541 | -126,902 | 213,9011 |  |
| 3 | 1 | 0,80000 | 3,214550 | 0,24887 | 0,8035 | 14,223 | -108,301 | 123,6966 |  |
| 3 | 2 | -4,29565 | 3,688211 | -1,16470 | 0,2441 | -91,204 | -289,413 | 60,9236 |  |

**Oneway Analysis of original-glszm-ZonePercentage By GrG**

**Quantiles**

| **Level** | **Minimum** | **10%** | **25%** | **Median** | **75%** | **90%** | **Maximum** |
| --- | --- | --- | --- | --- | --- | --- | --- |
| 1 | 0,511628 | 0,526602 | 0,571429 | 0,708333 | 0,740741 | 0,804082 | 0,816327 |
| 2 | 0,085106 | 0,504033 | 0,54902 | 0,6 | 0,708333 | 0,791297 | 0,814815 |
| 3 | 0,518771 | 0,528937 | 0,560345 | 0,581395 | 0,649438 | 0,702617 | 0,717172 |
| 4&5 | 0,484375 | 0,496043 | 0,548123 | 0,615659 | 0,686259 | 0,714286 | 0,722892 |

**Nonparametric Comparisons For Each Pair Using Wilcoxon Method**

| **q*** | **Alpha** |
| --- | --- |
| 1,95996 | 0,05 |

| **Level** | **- Level** | **Score Mean Difference** | **Std Err Dif** | **Z** | **p-Value** | **Hodges-Lehmann** | **Lower CL** | **Upper CL** | **Difference Plot** |
| --- | --- | --- | --- | --- | --- | --- | --- | --- | --- |
| 4&5 | 3 | 1,57500 | 3,499755 | 0,45003 | 0,6527 | 0,010986 | -0,037159 | 0,0648475 |  |
| 4&5 | 2 | -0,88804 | 3,838918 | -0,23133 | 0,8171 | -0,004456 | -0,067526 | 0,0547483 |  |
| 3 | 2 | -1,87246 | 3,688211 | -0,50769 | 0,6117 | -0,012471 | -0,074013 | 0,0447368 |  |
| 2 | 1 | -4,02029 | 3,688009 | -1,09010 | 0,2757 | -0,039162 | -0,128816 | 0,0285714 |  |
| 3 | 1 | -5,46667 | 3,214550 | -1,70060 | 0,0890 | -0,060314 | -0,147931 | 0,0099668 |  |
| 4&5 | 1 | -5,77500 | 3,499755 | -1,65012 | 0,0989 | -0,046895 | -0,127250 | 0,0079861 |  |

**Oneway Analysis of original-glszm-LargeAreaLowGrayLevelEmphasis By GrG**

**Quantiles**

| **Level** | **Minimum** | **10%** | **25%** | **Median** | **75%** | **90%** | **Maximum** |
| --- | --- | --- | --- | --- | --- | --- | --- |
| 1 | 0,01644 | 0,016481 | 0,018632 | 0,050637 | 0,112168 | 0,235851 | 0,294624 |
| 2 | 0,01035 | 0,011177 | 0,019871 | 0,031383 | 0,085716 | 1,28755 | 84,62674 |
| 3 | 0,009047 | 0,014239 | 0,0281 | 0,04589 | 0,083273 | 0,112098 | 0,128508 |
| 4&5 | 0,00856 | 0,008965 | 0,012288 | 0,028194 | 0,046954 | 0,109141 | 0,189797 |

**Nonparametric Comparisons For Each Pair Using Wilcoxon Method**

| **q*** | **Alpha** |
| --- | --- |
| 1,95996 | 0,05 |

| **Level** | **- Level** | **Score Mean Difference** | **Std Err Dif** | **Z** | **p-Value** | **Hodges-Lehmann** | **Lower CL** | **Upper CL** | **Difference Plot** |
| --- | --- | --- | --- | --- | --- | --- | --- | --- | --- |
| 3 | 2 | 2,64348 | 3,688211 | 0,71674 | 0,4735 | 0,008229 | -0,014268 | 0,0314652 |  |
| 3 | 1 | -1,06667 | 3,214550 | -0,33182 | 0,7400 | -0,002142 | -0,044930 | 0,0228657 |  |
| 2 | 1 | -2,86377 | 3,688211 | -0,77647 | 0,4375 | -0,008158 | -0,044985 | 0,0130393 |  |
| 4&5 | 2 | -3,59891 | 3,839063 | -0,93745 | 0,3485 | -0,005505 | -0,023100 | 0,0093929 |  |
| 4&5 | 3 | -5,30833 | 3,500000 | -1,51667 | 0,1294 | -0,015168 | -0,039930 | 0,0051143 |  |
| 4&5 | 1 | -6,24167 | 3,500000 | -1,78333 | 0,0745 | -0,019247 | -0,055920 | 0,0043659 |  |

**Oneway Analysis of original-glszm-LargeAreaHighGrayLevelEmphasis By GrG**

**Quantiles**

| **Level** | **Minimum** | **10%** | **25%** | **Median** | **75%** | **90%** | **Maximum** |
| --- | --- | --- | --- | --- | --- | --- | --- |
| 1 | 252,8421 | 387,0368 | 563,75 | 1152,355 | 2246,292 | 2940,065 | 3278,803 |
| 2 | 253,2308 | 727,6571 | 1074,826 | 1379,183 | 3250,506 | 6725,823 | 6802,799 |
| 3 | 677,7963 | 764,2042 | 883,4592 | 1558,138 | 2253,309 | 4708,437 | 6948,769 |
| 4&5 | 530 | 705,2991 | 1264,579 | 2324,806 | 4001,657 | 6930,869 | 15257,9 |

**Nonparametric Comparisons For Each Pair Using Wilcoxon Method**

| **q*** | **Alpha** |
| --- | --- |
| 1,95996 | 0,05 |

| **Level** | **- Level** | **Score Mean Difference** | **Std Err Dif** | **Z** | **p-Value** | **Hodges-Lehmann** | **Lower CL** | **Upper CL** | **Difference Plot** |
| --- | --- | --- | --- | --- | --- | --- | --- | --- | --- |
| 4&5 | 1 | 9,27500 | 3,500000 | 2,65000 | 0,0080* | 1018,76 | 162,750 | 2315,753 |  |
| 4&5 | 3 | 5,30833 | 3,500000 | 1,51667 | 0,1294 | 656,66 | -225,654 | 2094,876 |  |
| 2 | 1 | 5,17681 | 3,688211 | 1,40361 | 0,1604 | 515,95 | -230,029 | 1479,859 |  |
| 4&5 | 2 | 4,62717 | 3,839063 | 1,20529 | 0,2281 | 422,65 | -547,827 | 1654,104 |  |
| 3 | 1 | 3,20000 | 3,214550 | 0,99547 | 0,3195 | 345,31 | -416,231 | 1025,199 |  |
| 3 | 2 | -1,43188 | 3,688211 | -0,38823 | 0,6978 | -139,18 | -997,197 | 478,442 |  |

**Oneway Analysis of original-glszm-HighGrayLevelZoneEmphasis By GrG**

**Quantiles**

| **Level** | **Minimum** | **10%** | **25%** | **Median** | **75%** | **90%** | **Maximum** |
| --- | --- | --- | --- | --- | --- | --- | --- |
| 1 | 144,1842 | 176,8149 | 226,9182 | 291,0455 | 600,2209 | 925,8218 | 1111,346 |
| 2 | 7,5 | 132,3555 | 277,4348 | 528,5591 | 874,5977 | 1013,721 | 1093,52 |
| 3 | 142,2778 | 156,6825 | 272,581 | 351,96 | 520,2215 | 993,275 | 1129,31 |
| 4&5 | 159,8333 | 259,0631 | 299,6408 | 522,2714 | 805,0316 | 1400,416 | 2311,652 |

**Nonparametric Comparisons For Each Pair Using Wilcoxon Method**

| **q*** | **Alpha** |
| --- | --- |
| 1,95996 | 0,05 |

| **Level** | **- Level** | **Score Mean Difference** | **Std Err Dif** | **Z** | **p-Value** | **Hodges-Lehmann** | **Lower CL** | **Upper CL** | **Difference Plot** |
| --- | --- | --- | --- | --- | --- | --- | --- | --- | --- |
| 4&5 | 1 | 7,29167 | 3,500000 | 2,08333 | 0,0372* | 158,693 | 2,976 | 446,2755 |  |
| 2 | 1 | 5,72754 | 3,688211 | 1,55293 | 0,1204 | 146,727 | -55,915 | 375,2402 |  |
| 4&5 | 3 | 5,42500 | 3,500000 | 1,55000 | 0,1211 | 150,979 | -36,638 | 405,4941 |  |
| 4&5 | 2 | 1,44891 | 3,839063 | 0,37741 | 0,7059 | 34,039 | -172,948 | 280,2978 |  |
| 3 | 1 | 1,20000 | 3,214550 | 0,37330 | 0,7089 | 30,565 | -126,122 | 154,5695 |  |
| 3 | 2 | -4,73623 | 3,688211 | -1,28415 | 0,1991 | -126,945 | -369,060 | 74,5252 |  |

**Oneway Analysis of original-glszm-SmallAreaEmphasis By GrG**

**Quantiles**

| **Level** | **Minimum** | **10%** | **25%** | **Median** | **75%** | **90%** | **Maximum** |
| --- | --- | --- | --- | --- | --- | --- | --- |
| 1 | 0,660138 | 0,664254 | 0,710202 | 0,768056 | 0,830031 | 0,845323 | 0,846528 |
| 2 | 0,253802 | 0,627374 | 0,687943 | 0,725079 | 0,823014 | 0,849016 | 0,857323 |
| 3 | 0,652384 | 0,658639 | 0,710742 | 0,731957 | 0,75873 | 0,788883 | 0,795858 |
| 4&5 | 0,688683 | 0,695832 | 0,713195 | 0,73133 | 0,777976 | 0,822787 | 0,849121 |

**Nonparametric Comparisons For Each Pair Using Wilcoxon Method**

| **q*** | **Alpha** |
| --- | --- |
| 1,95996 | 0,05 |

| **Level** | **- Level** | **Score Mean Difference** | **Std Err Dif** | **Z** | **p-Value** | **Hodges-Lehmann** | **Lower CL** | **Upper CL** | **Difference Plot** |
| --- | --- | --- | --- | --- | --- | --- | --- | --- | --- |
| 4&5 | 2 | 1,91630 | 3,839063 | 0,49916 | 0,6177 | 0,008715 | -0,030587 | 0,0436161 |  |
| 4&5 | 3 | 0,75833 | 3,500000 | 0,21667 | 0,8285 | 0,002129 | -0,024703 | 0,0425029 |  |
| 3 | 2 | 0,33043 | 3,688211 | 0,08959 | 0,9286 | 0,002616 | -0,047509 | 0,0429771 |  |
| 4&5 | 1 | -2,50833 | 3,500000 | -0,71667 | 0,4736 | -0,016715 | -0,063912 | 0,0228507 |  |
| 2 | 1 | -3,74493 | 3,688211 | -1,01538 | 0,3099 | -0,019997 | -0,081179 | 0,0209440 |  |
| 3 | 1 | -3,86667 | 3,214550 | -1,20286 | 0,2290 | -0,033424 | -0,084921 | 0,0189357 |  |

**Oneway Analysis of original-glszm-LowGrayLevelZoneEmphasis By GrG**

**Quantiles**

| **Level** | **Minimum** | **10%** | **25%** | **Median** | **75%** | **90%** | **Maximum** |
| --- | --- | --- | --- | --- | --- | --- | --- |
| 1 | 0,007373 | 0,007383 | 0,014345 | 0,025605 | 0,04282 | 0,053846 | 0,055997 |
| 2 | 0,002538 | 0,00307 | 0,013112 | 0,022124 | 0,042736 | 0,092695 | 0,355903 |
| 3 | 0,003132 | 0,006598 | 0,013572 | 0,017757 | 0,033441 | 0,05316 | 0,072188 |
| 4&5 | 0,001198 | 0,002115 | 0,005 | 0,011928 | 0,02674 | 0,066616 | 0,072304 |

**Nonparametric Comparisons For Each Pair Using Wilcoxon Method**

| **q*** | **Alpha** |
| --- | --- |
| 1,95996 | 0,05 |

| **Level** | **- Level** | **Score Mean Difference** | **Std Err Dif** | **Z** | **p-Value** | **Hodges-Lehmann** | **Lower CL** | **Upper CL** | **Difference Plot** |
| --- | --- | --- | --- | --- | --- | --- | --- | --- | --- |
| 3 | 2 | -0,55072 | 3,688211 | -0,14932 | 0,8813 | -0,000525 | -0,013622 | 0,011317 |  |
| 2 | 1 | -2,09275 | 3,688211 | -0,56742 | 0,5704 | -0,004020 | -0,017478 | 0,010062 |  |
| 3 | 1 | -2,53333 | 3,214550 | -0,78808 | 0,4306 | -0,005436 | -0,017987 | 0,007119 |  |
| 4&5 | 2 | -5,56196 | 3,839063 | -1,44878 | 0,1474 | -0,008579 | -0,018773 | 0,002230 |  |
| 4&5 | 3 | -5,77500 | 3,500000 | -1,65000 | 0,0989 | -0,007829 | -0,015567 | 0,001636 |  |
| 4&5 | 1 | -7,29167 | 3,500000 | -2,08333 | 0,0372* | -0,011221 | -0,023668 | -0,000400 |  |

**Oneway Analysis of original-glszm-ZoneEntropy By GrG**

**Quantiles**

| **Level** | **Minimum** | **10%** | **25%** | **Median** | **75%** | **90%** | **Maximum** |
| --- | --- | --- | --- | --- | --- | --- | --- |
| 1 | 3,984184 | 4,279252 | 4,596439 | 5,214288 | 5,674554 | 5,888476 | 6,062191 |
| 2 | 2 | 3,742917 | 4,896821 | 5,535029 | 6,077099 | 6,624341 | 6,78314 |
| 3 | 4,201841 | 4,559816 | 5,235749 | 5,738001 | 6,082177 | 6,344323 | 6,379709 |
| 4&5 | 3,921928 | 4,701304 | 5,114276 | 6,0487 | 6,554981 | 6,875855 | 7,305317 |

**Nonparametric Comparisons For Each Pair Using Wilcoxon Method**

| **q*** | **Alpha** |
| --- | --- |
| 1,95996 | 0,05 |

| **Level** | **- Level** | **Score Mean Difference** | **Std Err Dif** | **Z** | **p-Value** | **Hodges-Lehmann** | **Lower CL** | **Upper CL** | **Difference Plot** |
| --- | --- | --- | --- | --- | --- | --- | --- | --- | --- |
| 4&5 | 1 | 8,925000 | 3,500000 | 2,550000 | 0,0108* | 0,7158930 | 0,197796 | 1,277145 |  |
| 3 | 1 | 6,933333 | 3,214550 | 2,156860 | 0,0310* | 0,4731874 | 0,019986 | 0,979367 |  |
| 4&5 | 2 | 6,403261 | 3,839063 | 1,667923 | 0,0953 | 0,5172517 | -0,115473 | 1,085830 |  |
| 4&5 | 3 | 4,141667 | 3,500000 | 1,183333 | 0,2367 | 0,2864095 | -0,279913 | 0,801683 |  |
| 2 | 1 | 3,414493 | 3,688211 | 0,925786 | 0,3546 | 0,2960129 | -0,328488 | 0,866116 |  |
| 3 | 2 | 2,753623 | 3,688211 | 0,746601 | 0,4553 | 0,2078583 | -0,359037 | 0,761065 |  |

**Oneway Analysis of original-glszm-SmallAreaLowGrayLevelEmphasis By GrG**

**Quantiles**

| **Level** | **Minimum** | **10%** | **25%** | **Median** | **75%** | **90%** | **Maximum** |
| --- | --- | --- | --- | --- | --- | --- | --- |
| 1 | 0,006442 | 0,006696 | 0,011173 | 0,019354 | 0,036188 | 0,049088 | 0,053987 |
| 2 | 0,002079 | 0,002554 | 0,012688 | 0,018636 | 0,028429 | 0,059138 | 0,108879 |
| 3 | 0,002845 | 0,006062 | 0,00971 | 0,016752 | 0,031641 | 0,048068 | 0,066936 |
| 4&5 | 0,001018 | 0,001356 | 0,004064 | 0,010378 | 0,022379 | 0,046263 | 0,071915 |

**Nonparametric Comparisons For Each Pair Using Wilcoxon Method**

| **q*** | **Alpha** |
| --- | --- |
| 1,95996 | 0,05 |

| **Level** | **- Level** | **Score Mean Difference** | **Std Err Dif** | **Z** | **p-Value** | **Hodges-Lehmann** | **Lower CL** | **Upper CL** | **Difference Plot** |
| --- | --- | --- | --- | --- | --- | --- | --- | --- | --- |
| 3 | 2 | -0,66087 | 3,688211 | -0,17918 | 0,8578 | -0,000681 | -0,010131 | 0,0095072 |  |
| 2 | 1 | -0,99130 | 3,688211 | -0,26878 | 0,7881 | -0,001420 | -0,011288 | 0,0080416 |  |
| 3 | 1 | -2,00000 | 3,214550 | -0,62217 | 0,5338 | -0,002601 | -0,011263 | 0,0088650 |  |
| 4&5 | 3 | -5,54167 | 3,500000 | -1,58333 | 0,1133 | -0,006269 | -0,014239 | 0,0020954 |  |
| 4&5 | 2 | -5,84239 | 3,839063 | -1,52183 | 0,1281 | -0,007264 | -0,014833 | 0,0010974 |  |
| 4&5 | 1 | -6,70833 | 3,500000 | -1,91667 | 0,0553 | -0,007680 | -0,016366 | 0,0008218 |  |

**Oneway Analysis of original-ngtdm-Coarseness By GrG**

**Quantiles**

| **Level** | **Minimum** | **10%** | **25%** | **Median** | **75%** | **90%** | **Maximum** |
| --- | --- | --- | --- | --- | --- | --- | --- |
| 1 | 0,016537 | 0,01692 | 0,028659 | 0,050707 | 0,064517 | 0,097117 | 0,117766 |
| 2 | 0,002607 | 0,005544 | 0,022386 | 0,038063 | 0,056627 | 0,107871 | 0,14397 |
| 3 | 0,005745 | 0,006719 | 0,016186 | 0,023072 | 0,047058 | 0,079095 | 0,118322 |
| 4&5 | 0,00052 | 0,002236 | 0,005331 | 0,018483 | 0,044497 | 0,066664 | 0,109965 |

**Nonparametric Comparisons For Each Pair Using Wilcoxon Method**

| **q*** | **Alpha** |
| --- | --- |
| 1,95996 | 0,05 |

| **Level** | **- Level** | **Score Mean Difference** | **Std Err Dif** | **Z** | **p-Value** | **Hodges-Lehmann** | **Lower CL** | **Upper CL** | **Difference Plot** |
| --- | --- | --- | --- | --- | --- | --- | --- | --- | --- |
| 2 | 1 | -2,09275 | 3,688211 | -0,56742 | 0,5704 | -0,007496 | -0,025634 | 0,013229 |  |
| 4&5 | 3 | -3,55833 | 3,500000 | -1,01667 | 0,3093 | -0,005889 | -0,020322 | 0,009090 |  |
| 3 | 2 | -4,73623 | 3,688211 | -1,28415 | 0,1991 | -0,009569 | -0,027767 | 0,006597 |  |
| 3 | 1 | -6,13333 | 3,214550 | -1,90799 | 0,0564 | -0,015546 | -0,036293 | 0,001443 |  |
| 4&5 | 2 | -7,43152 | 3,839063 | -1,93576 | 0,0529 | -0,016012 | -0,033470 | 0,000559 |  |
| 4&5 | 1 | -8,92500 | 3,500000 | -2,55000 | 0,0108* | -0,023226 | -0,041227 | -0,005273 |  |

**Oneway Analysis of original-ngtdm-Complexity By GrG**

**Quantiles**

| **Level** | **Minimum** | **10%** | **25%** | **Median** | **75%** | **90%** | **Maximum** |
| --- | --- | --- | --- | --- | --- | --- | --- |
| 1 | 606,834 | 623,6909 | 1022,625 | 1262,63 | 2253,003 | 3817,247 | 5510,771 |
| 2 | 3,066231 | 316,3025 | 1053,749 | 2147,659 | 3451,204 | 5704,362 | 7839,335 |
| 3 | 378,8591 | 502,5873 | 897,6132 | 1723,112 | 3985,783 | 4652,343 | 5329,073 |
| 4&5 | 535,5644 | 638,3771 | 1205,049 | 2672,229 | 5815,856 | 11326,57 | 21094,04 |

**Nonparametric Comparisons For Each Pair Using Wilcoxon Method**

| **q*** | **Alpha** |
| --- | --- |
| 1,95996 | 0,05 |

| **Level** | **- Level** | **Score Mean Difference** | **Std Err Dif** | **Z** | **p-Value** | **Hodges-Lehmann** | **Lower CL** | **Upper CL** | **Difference Plot** |
| --- | --- | --- | --- | --- | --- | --- | --- | --- | --- |
| 4&5 | 1 | 6,35833 | 3,500000 | 1,81667 | 0,0693 | 935,376 | -74,59 | 3191,137 |  |
| 4&5 | 2 | 4,06630 | 3,839063 | 1,05919 | 0,2895 | 594,933 | -490,27 | 2481,205 |  |
| 2 | 1 | 3,85507 | 3,688211 | 1,04524 | 0,2959 | 446,542 | -430,43 | 1855,452 |  |
| 4&5 | 3 | 3,79167 | 3,500000 | 1,08333 | 0,2787 | 710,839 | -462,42 | 2831,953 |  |
| 3 | 1 | 1,86667 | 3,214550 | 0,58069 | 0,5614 | 340,945 | -513,40 | 1512,959 |  |
| 3 | 2 | -0,66087 | 3,688211 | -0,17918 | 0,8578 | -117,355 | -1347,23 | 854,760 |  |

**Oneway Analysis of original-ngtdm-Strength By GrG**

**Quantiles**

| **Level** | **Minimum** | **10%** | **25%** | **Median** | **75%** | **90%** | **Maximum** |
| --- | --- | --- | --- | --- | --- | --- | --- |
| 1 | 5,576144 | 6,914202 | 9,105158 | 14,13712 | 24,71015 | 28,97449 | 29,14612 |
| 2 | 0,789187 | 3,880086 | 7,709753 | 14,58148 | 22,46106 | 34,50761 | 38,38237 |
| 3 | 3,267361 | 3,656958 | 6,760134 | 10,78929 | 14,41082 | 18,55781 | 18,66805 |
| 4&5 | 1,958236 | 2,067434 | 4,149771 | 8,33967 | 20,56637 | 25,79032 | 31,46339 |

**Nonparametric Comparisons For Each Pair Using Wilcoxon Method**

| **q*** | **Alpha** |
| --- | --- |
| 1,95996 | 0,05 |

| **Level** | **- Level** | **Score Mean Difference** | **Std Err Dif** | **Z** | **p-Value** | **Hodges-Lehmann** | **Lower CL** | **Upper CL** | **Difference Plot** |
| --- | --- | --- | --- | --- | --- | --- | --- | --- | --- |
| 4&5 | 3 | 0,00000 | 3,500000 | 0,00000 | 1,0000 | -0,18280 | -4,8525 | 7,008399 |  |
| 2 | 1 | -0,88116 | 3,688211 | -0,23891 | 0,8112 | -0,72721 | -7,0680 | 5,879325 |  |
| 4&5 | 2 | -5,28152 | 3,839063 | -1,37573 | 0,1689 | -4,04634 | -9,9375 | 2,635044 |  |
| 3 | 1 | -5,73333 | 3,214550 | -1,78356 | 0,0745 | -4,59633 | -12,0002 | 0,432621 |  |
| 4&5 | 1 | -5,77500 | 3,500000 | -1,65000 | 0,0989 | -4,22122 | -9,8175 | 1,263966 |  |
| 3 | 2 | -6,05797 | 3,688211 | -1,64252 | 0,1005 | -4,21060 | -10,2919 | 1,474027 |  |

**Oneway Analysis of original-ngtdm-Contrast By GrG**

**Quantiles**

| **Level** | **Minimum** | **10%** | **25%** | **Median** | **75%** | **90%** | **Maximum** |
| --- | --- | --- | --- | --- | --- | --- | --- |
| 1 | 0,270921 | 0,27753 | 0,350578 | 0,72397 | 0,996461 | 1,449447 | 1,902302 |
| 2 | 0,049897 | 0,173014 | 0,260094 | 0,520331 | 1,000909 | 2,270286 | 2,530431 |
| 3 | 0,218599 | 0,241971 | 0,272655 | 0,42029 | 0,625861 | 0,678572 | 0,678915 |
| 4&5 | 0,150017 | 0,18304 | 0,230044 | 0,3839 | 0,568869 | 1,742607 | 2,086431 |

**Nonparametric Comparisons For Each Pair Using Wilcoxon Method**

| **q*** | **Alpha** |
| --- | --- |
| 1,95996 | 0,05 |

| **Level** | **- Level** | **Score Mean Difference** | **Std Err Dif** | **Z** | **p-Value** | **Hodges-Lehmann** | **Lower CL** | **Upper CL** | **Difference Plot** |
| --- | --- | --- | --- | --- | --- | --- | --- | --- | --- |
| 2 | 1 | -1,98261 | 3,688211 | -0,53755 | 0,5909 | -0,067657 | -0,361562 | 0,284321 |  |
| 4&5 | 3 | -2,15833 | 3,500000 | -0,61667 | 0,5375 | -0,042843 | -0,172342 | 0,136242 |  |
| 3 | 2 | -4,07536 | 3,688211 | -1,10497 | 0,2692 | -0,123919 | -0,407781 | 0,079750 |  |
| 4&5 | 2 | -5,00109 | 3,839063 | -1,30268 | 0,1927 | -0,149980 | -0,414183 | 0,054764 |  |
| 3 | 1 | -6,66667 | 3,214550 | -2,07390 | 0,0381* | -0,209064 | -0,505370 | -0,007373 |  |
| 4&5 | 1 | -7,29167 | 3,500000 | -2,08333 | 0,0372* | -0,198720 | -0,517372 | -0,009875 |  |

**Oneway Analysis of original-ngtdm-Busyness By GrG**

**Quantiles**

| **Level** | **Minimum** | **10%** | **25%** | **Median** | **75%** | **90%** | **Maximum** |
| --- | --- | --- | --- | --- | --- | --- | --- |
| 1 | 0,023412 | 0,024812 | 0,038078 | 0,067907 | 0,093671 | 0,107748 | 0,108661 |
| 2 | 0,024852 | 0,032656 | 0,040403 | 0,055647 | 0,094038 | 0,169509 | 1,295467 |
| 3 | 0,039493 | 0,041587 | 0,062269 | 0,082431 | 0,104663 | 0,164187 | 0,181286 |
| 4&5 | 0,033325 | 0,036401 | 0,039684 | 0,080029 | 0,191622 | 0,384327 | 0,421662 |

**Nonparametric Comparisons For Each Pair Using Wilcoxon Method**

| **q*** | **Alpha** |
| --- | --- |
| 1,95996 | 0,05 |

| **Level** | **- Level** | **Score Mean Difference** | **Std Err Dif** | **Z** | **p-Value** | **Hodges-Lehmann** | **Lower CL** | **Upper CL** | **Difference Plot** |
| --- | --- | --- | --- | --- | --- | --- | --- | --- | --- |
| 3 | 2 | 5,50725 | 3,688211 | 1,49320 | 0,1354 | 0,016588 | -0,008025 | 0,0416674 |  |
| 4&5 | 2 | 4,25326 | 3,839063 | 1,10789 | 0,2679 | 0,012894 | -0,011895 | 0,0615444 |  |
| 4&5 | 1 | 4,14167 | 3,500000 | 1,18333 | 0,2367 | 0,016886 | -0,015238 | 0,0782489 |  |
| 3 | 1 | 4,00000 | 3,214550 | 1,24434 | 0,2134 | 0,015296 | -0,009910 | 0,0456832 |  |
| 4&5 | 3 | 0,00000 | 3,500000 | 0,00000 | 1,0000 | -0,000567 | -0,029738 | 0,0644021 |  |
| 2 | 1 | -0,33043 | 3,688211 | -0,08959 | 0,9286 | -0,001800 | -0,026061 | 0,0261315 |  |
