# Supplementary material for: Comparison of machine learning algorithms to predict clinically significant prostate cancer of the peripheral zone with multiparametric MRI using clinical assessment categories and radiomic features
Source: Eur Radiol. 2020 Jul 16;30(12):6757–69. doi: 10.1007/s00330-020-07064-5 (PMC7599168; doi:10.1007/s00330-020-07064-5)
Supplement: Supplementary file 4 — (DOC 4497 kb) [file 330_2020_7064_MOESM4_ESM.doc]

**Fit Group**

**Oneway Analysis of original-shape-Maximum3DDiameter By GrG <=2 vs >=3**

**Quantiles**

| **Level** | **Minimum** | **10%** | **25%** | **Median** | **75%** | **90%** | **Maximum** |
| --- | --- | --- | --- | --- | --- | --- | --- |
| 1/2 | 9,641576 | 9,92285 | 15,29905 | 18,68871 | 21,21879 | 39,39422 | 57,28075 |
| 3/4&5 | 11,33647 | 13,00336 | 17,74587 | 24,67963 | 30,57959 | 51,55602 | 89,77997 |

**Oneway Anova**

**Summary of Fit**

| Rsquare | 0,06443 |
| --- | --- |
| Adj Rsquare | 0,04958 |
| Root Mean Square Error | 13,67491 |
| Mean of Response | 24,59501 |
| Observations (or Sum Wgts) | 65 |

**Pooled t Test**

3/4&5-1/2

Assuming equal variances

| Difference | 7,0870 | t Ratio | 2,082946 |
| --- | --- | --- | --- |
| Std Err Dif | 3,4024 | DF | 63 |
| Upper CL Dif | 13,8862 | Prob > |t| | 0,0413* |
| Lower CL Dif | 0,2879 | Prob > t | 0,0207* |
| Confidence | 0,95 | Prob < t | 0,9793 |

**Analysis of Variance**

| **Source** | **DF** | **Sum of Squares** | **Mean Square** | **F Ratio** | **Prob > F** |
| --- | --- | --- | --- | --- | --- |
| GrG <=2 vs >=3 | 1 | 811,343 | 811,343 | 4,3387 | 0,0413* |
| Error | 63 | 11781,195 | 187,003 |  |  |
| C. Total | 64 | 12592,538 |  |  |  |

**Means for Oneway Anova**

| **Level** | **Number** | **Mean** | **Std Error** | **Lower 95%** | **Upper 95%** |
| --- | --- | --- | --- | --- | --- |
| 1/2 | 30 | 20,7789 | 2,4967 | 15,790 | 25,768 |
| 3/4&5 | 35 | 27,8659 | 2,3115 | 23,247 | 32,485 |

Std Error uses a pooled estimate of error variance

**Nonparametric Comparisons For Each Pair Using Wilcoxon Method**

| **q*** | **Alpha** |
| --- | --- |
| 1,95996 | 0,05 |

| **Level** | **- Level** | **Score Mean Difference** | **Std Err Dif** | **Z** | **p-Value** | **Hodges-Lehmann** | **Lower CL** | **Upper CL** | **Difference Plot** |
| --- | --- | --- | --- | --- | --- | --- | --- | --- | --- |
| 3/4&5 | 1/2 | 11,48333 | 4,704355 | 2,441001 | 0,0146* | 5,642008 | 1,413198 | 9,397937 |  |

**Oneway Analysis of original-shape-Maximum2DDiameterSlice By GrG <=2 vs >=3**

**Quantiles**

| **Level** | **Minimum** | **10%** | **25%** | **Median** | **75%** | **90%** | **Maximum** |
| --- | --- | --- | --- | --- | --- | --- | --- |
| 1/2 | 7,18022 | 8,23844 | 11,69301 | 16,46366 | 20,13232 | 34,49965 | 56,01587 |
| 3/4&5 | 7,333333 | 11,56331 | 12,44508 | 21,08185 | 28,50341 | 43,56286 | 72,60242 |

**Oneway Anova**

**Summary of Fit**

| Rsquare | 0,055061 |
| --- | --- |
| Adj Rsquare | 0,040061 |
| Root Mean Square Error | 12,39645 |
| Mean of Response | 21,10011 |
| Observations (or Sum Wgts) | 65 |

**Pooled t Test**

3/4&5-1/2

Assuming equal variances

| Difference | 5,909 | t Ratio | 1,915968 |
| --- | --- | --- | --- |
| Std Err Dif | 3,084 | DF | 63 |
| Upper CL Dif | 12,073 | Prob > |t| | 0,0599 |
| Lower CL Dif | -0,254 | Prob > t | 0,0300* |
| Confidence | 0,95 | Prob < t | 0,9700 |

**Analysis of Variance**

| **Source** | **DF** | **Sum of Squares** | **Mean Square** | **F Ratio** | **Prob > F** |
| --- | --- | --- | --- | --- | --- |
| GrG <=2 vs >=3 | 1 | 564,120 | 564,120 | 3,6709 | 0,0599 |
| Error | 63 | 9681,336 | 153,672 |  |  |
| C. Total | 64 | 10245,456 |  |  |  |

**Means for Oneway Anova**

| **Level** | **Number** | **Mean** | **Std Error** | **Lower 95%** | **Upper 95%** |
| --- | --- | --- | --- | --- | --- |
| 1/2 | 30 | 17,9181 | 2,2633 | 13,395 | 22,441 |
| 3/4&5 | 35 | 23,8276 | 2,0954 | 19,640 | 28,015 |

Std Error uses a pooled estimate of error variance

**Nonparametric Comparisons For Each Pair Using Wilcoxon Method**

| **q*** | **Alpha** |
| --- | --- |
| 1,95996 | 0,05 |

| **Level** | **- Level** | **Score Mean Difference** | **Std Err Dif** | **Z** | **p-Value** | **Hodges-Lehmann** | **Lower CL** | **Upper CL** | **Difference Plot** |
| --- | --- | --- | --- | --- | --- | --- | --- | --- | --- |
| 3/4&5 | 1/2 | 10,61667 | 4,703789 | 2,257046 | 0,0240* | 4,359278 | 0,6857730 | 8,956381 |  |

**Oneway Analysis of original-shape-Sphericity By GrG <=2 vs >=3**

**Quantiles**

| **Level** | **Minimum** | **10%** | **25%** | **Median** | **75%** | **90%** | **Maximum** |
| --- | --- | --- | --- | --- | --- | --- | --- |
| 1/2 | 0,444155 | 0,462688 | 0,50836 | 0,589535 | 0,675414 | 0,824563 | 0,890704 |
| 3/4&5 | 0,367849 | 0,429981 | 0,501388 | 0,600324 | 0,676724 | 0,734874 | 0,753439 |

**Oneway Anova**

**Summary of Fit**

| Rsquare | 0,009875 |
| --- | --- |
| Adj Rsquare | -0,00584 |
| Root Mean Square Error | 0,114831 |
| Mean of Response | 0,598113 |
| Observations (or Sum Wgts) | 65 |

**Pooled t Test**

3/4&5-1/2

Assuming equal variances

| Difference | -0,02265 | t Ratio | -0,79266 |
| --- | --- | --- | --- |
| Std Err Dif | 0,02857 | DF | 63 |
| Upper CL Dif | 0,03445 | Prob > |t| | 0,4310 |
| Lower CL Dif | -0,07974 | Prob > t | 0,7845 |
| Confidence | 0,95 | Prob < t | 0,2155 |

**Analysis of Variance**

| **Source** | **DF** | **Sum of Squares** | **Mean Square** | **F Ratio** | **Prob > F** |
| --- | --- | --- | --- | --- | --- |
| GrG <=2 vs >=3 | 1 | 0,00828502 | 0,008285 | 0,6283 | 0,4310 |
| Error | 63 | 0,83073411 | 0,013186 |  |  |
| C. Total | 64 | 0,83901913 |  |  |  |

**Means for Oneway Anova**

| **Level** | **Number** | **Mean** | **Std Error** | **Lower 95%** | **Upper 95%** |
| --- | --- | --- | --- | --- | --- |
| 1/2 | 30 | 0,610308 | 0,02097 | 0,56841 | 0,65220 |
| 3/4&5 | 35 | 0,587661 | 0,01941 | 0,54887 | 0,62645 |

Std Error uses a pooled estimate of error variance

**Nonparametric Comparisons For Each Pair Using Wilcoxon Method**

| **q*** | **Alpha** |
| --- | --- |
| 1,95996 | 0,05 |

| **Level** | **- Level** | **Score Mean Difference** | **Std Err Dif** | **Z** | **p-Value** | **Hodges-Lehmann** | **Lower CL** | **Upper CL** | **Difference Plot** |
| --- | --- | --- | --- | --- | --- | --- | --- | --- | --- |
| 3/4&5 | 1/2 | -1,76429 | 4,704355 | -0,375032 | 0,7076 | -0,010040 | -0,073848 | 0,0507388 |  |

**Oneway Analysis of original-shape-MinorAxis By GrG <=2 vs >=3**

**Quantiles**

| **Level** | **Minimum** | **10%** | **25%** | **Median** | **75%** | **90%** | **Maximum** |
| --- | --- | --- | --- | --- | --- | --- | --- |
| 1/2 | 6,658432 | 7,410975 | 9,391673 | 12,08262 | 14,70817 | 27,62001 | 33,44799 |
| 3/4&5 | 6,463433 | 9,009079 | 10,67523 | 14,99432 | 16,94276 | 35,09984 | 60,20007 |

**Oneway Anova**

**Summary of Fit**

| Rsquare | 0,045403 |
| --- | --- |
| Adj Rsquare | 0,03025 |
| Root Mean Square Error | 9,339237 |
| Mean of Response | 15,92382 |
| Observations (or Sum Wgts) | 65 |

**Pooled t Test**

3/4&5-1/2

Assuming equal variances

| Difference | 4,0223 | t Ratio | 1,731015 |
| --- | --- | --- | --- |
| Std Err Dif | 2,3237 | DF | 63 |
| Upper CL Dif | 8,6658 | Prob > |t| | 0,0883 |
| Lower CL Dif | -0,6212 | Prob > t | 0,0442* |
| Confidence | 0,95 | Prob < t | 0,9558 |

**Analysis of Variance**

| **Source** | **DF** | **Sum of Squares** | **Mean Square** | **F Ratio** | **Prob > F** |
| --- | --- | --- | --- | --- | --- |
| GrG <=2 vs >=3 | 1 | 261,3513 | 261,351 | 2,9964 | 0,0883 |
| Error | 63 | 5494,9446 | 87,221 |  |  |
| C. Total | 64 | 5756,2959 |  |  |  |

**Means for Oneway Anova**

| **Level** | **Number** | **Mean** | **Std Error** | **Lower 95%** | **Upper 95%** |
| --- | --- | --- | --- | --- | --- |
| 1/2 | 30 | 13,7580 | 1,7051 | 10,351 | 17,165 |
| 3/4&5 | 35 | 17,7803 | 1,5786 | 14,626 | 20,935 |

Std Error uses a pooled estimate of error variance

**Nonparametric Comparisons For Each Pair Using Wilcoxon Method**

| **q*** | **Alpha** |
| --- | --- |
| 1,95996 | 0,05 |

| **Level** | **- Level** | **Score Mean Difference** | **Std Err Dif** | **Z** | **p-Value** | **Hodges-Lehmann** | **Lower CL** | **Upper CL** | **Difference Plot** |
| --- | --- | --- | --- | --- | --- | --- | --- | --- | --- |
| 3/4&5 | 1/2 | 9,007143 | 4,704355 | 1,914639 | 0,0555 | 2,676297 | -0,070783 | 4,961815 |  |

**Oneway Analysis of original-shape-Elongation By GrG <=2 vs >=3**

**Quantiles**

| **Level** | **Minimum** | **10%** | **25%** | **Median** | **75%** | **90%** | **Maximum** |
| --- | --- | --- | --- | --- | --- | --- | --- |
| 1/2 | 0,466848 | 0,524837 | 0,636473 | 0,713239 | 0,836079 | 0,878747 | 0,901339 |
| 3/4&5 | 0,384147 | 0,544512 | 0,639581 | 0,723974 | 0,826245 | 0,894992 | 0,908154 |

**Oneway Anova**

**Summary of Fit**

| Rsquare | 8,521e-5 |
| --- | --- |
| Adj Rsquare | -0,01579 |
| Root Mean Square Error | 0,128648 |
| Mean of Response | 0,721483 |
| Observations (or Sum Wgts) | 65 |

**Pooled t Test**

3/4&5-1/2

Assuming equal variances

| Difference | 0,00235 | t Ratio | 0,07327 |
| --- | --- | --- | --- |
| Std Err Dif | 0,03201 | DF | 63 |
| Upper CL Dif | 0,06631 | Prob > |t| | 0,9418 |
| Lower CL Dif | -0,06162 | Prob > t | 0,4709 |
| Confidence | 0,95 | Prob < t | 0,5291 |

**Analysis of Variance**

| **Source** | **DF** | **Sum of Squares** | **Mean Square** | **F Ratio** | **Prob > F** |
| --- | --- | --- | --- | --- | --- |
| GrG <=2 vs >=3 | 1 | 0,0000889 | 0,000089 | 0,0054 | 0,9418 |
| Error | 63 | 1,0426691 | 0,016550 |  |  |
| C. Total | 64 | 1,0427579 |  |  |  |

**Means for Oneway Anova**

| **Level** | **Number** | **Mean** | **Std Error** | **Lower 95%** | **Upper 95%** |
| --- | --- | --- | --- | --- | --- |
| 1/2 | 30 | 0,720221 | 0,02349 | 0,67328 | 0,76716 |
| 3/4&5 | 35 | 0,722566 | 0,02175 | 0,67911 | 0,76602 |

Std Error uses a pooled estimate of error variance

**Nonparametric Comparisons For Each Pair Using Wilcoxon Method**

| **q*** | **Alpha** |
| --- | --- |
| 1,95996 | 0,05 |

| **Level** | **- Level** | **Score Mean Difference** | **Std Err Dif** | **Z** | **p-Value** | **Hodges-Lehmann** | **Lower CL** | **Upper CL** | **Difference Plot** |
| --- | --- | --- | --- | --- | --- | --- | --- | --- | --- |
| 3/4&5 | 1/2 | 1,330952 | 4,704355 | 0,2829192 | 0,7772 | 0,0072238 | -0,060414 | 0,0679889 |  |

**Oneway Analysis of original-shape-SurfaceVolumeRatio By GrG <=2 vs >=3**

**Quantiles**

| **Level** | **Minimum** | **10%** | **25%** | **Median** | **75%** | **90%** | **Maximum** |
| --- | --- | --- | --- | --- | --- | --- | --- |
| 1/2 | 0,410717 | 0,521179 | 0,688822 | 0,826565 | 0,959185 | 1,056588 | 1,167969 |
| 3/4&5 | 0,236315 | 0,392017 | 0,564509 | 0,638368 | 0,847445 | 0,94709 | 1,192864 |

**Oneway Anova**

**Summary of Fit**

| Rsquare | 0,105991 |
| --- | --- |
| Adj Rsquare | 0,091801 |
| Root Mean Square Error | 0,197712 |
| Mean of Response | 0,74414 |
| Observations (or Sum Wgts) | 65 |

**Pooled t Test**

3/4&5-1/2

Assuming equal variances

| Difference | -0,13444 | t Ratio | -2,73297 |
| --- | --- | --- | --- |
| Std Err Dif | 0,04919 | DF | 63 |
| Upper CL Dif | -0,03614 | Prob > |t| | 0,0081* |
| Lower CL Dif | -0,23274 | Prob > t | 0,9959 |
| Confidence | 0,95 | Prob < t | 0,0041* |

**Analysis of Variance**

| **Source** | **DF** | **Sum of Squares** | **Mean Square** | **F Ratio** | **Prob > F** |
| --- | --- | --- | --- | --- | --- |
| GrG <=2 vs >=3 | 1 | 0,2919697 | 0,291970 | 7,4691 | 0,0081* |
| Error | 63 | 2,4626843 | 0,039090 |  |  |
| C. Total | 64 | 2,7546540 |  |  |  |

**Means for Oneway Anova**

| **Level** | **Number** | **Mean** | **Std Error** | **Lower 95%** | **Upper 95%** |
| --- | --- | --- | --- | --- | --- |
| 1/2 | 30 | 0,816531 | 0,03610 | 0,74440 | 0,88867 |
| 3/4&5 | 35 | 0,682090 | 0,03342 | 0,61531 | 0,74887 |

Std Error uses a pooled estimate of error variance

**Nonparametric Comparisons For Each Pair Using Wilcoxon Method**

| **q*** | **Alpha** |
| --- | --- |
| 1,95996 | 0,05 |

| **Level** | **- Level** | **Score Mean Difference** | **Std Err Dif** | **Z** | **p-Value** | **Hodges-Lehmann** | **Lower CL** | **Upper CL** | **Difference Plot** |
| --- | --- | --- | --- | --- | --- | --- | --- | --- | --- |
| 3/4&5 | 1/2 | -12,3500 | 4,704355 | -2,62523 | 0,0087* | -0,140094 | -0,240153 | -0,040856 |  |

**Oneway Analysis of original-shape-Volume By GrG <=2 vs >=3**

**Quantiles**

| **Level** | **Minimum** | **10%** | **25%** | **Median** | **75%** | **90%** | **Maximum** |
| --- | --- | --- | --- | --- | --- | --- | --- |
| 1/2 | 204,8004 | 302,8492 | 533,3762 | 844,2877 | 1729,6 | 8905,6 | 18630,39 |
| 3/4&5 | 179,1999 | 462,08 | 735,6805 | 1919,999 | 2982,399 | 19595,51 | 142600,5 |

**Oneway Anova**

**Summary of Fit**

| Rsquare | 0,026345 |
| --- | --- |
| Adj Rsquare | 0,01089 |
| Root Mean Square Error | 18002,93 |
| Mean of Response | 5393,539 |
| Observations (or Sum Wgts) | 65 |

**Pooled t Test**

3/4&5-1/2

Assuming equal variances

| Difference | 5848 | t Ratio | 1,305624 |
| --- | --- | --- | --- |
| Std Err Dif | 4479 | DF | 63 |
| Upper CL Dif | 14799 | Prob > |t| | 0,1964 |
| Lower CL Dif | -3103 | Prob > t | 0,0982 |
| Confidence | 0,95 | Prob < t | 0,9018 |

**Analysis of Variance**

| **Source** | **DF** | **Sum of Squares** | **Mean Square** | **F Ratio** | **Prob > F** |
| --- | --- | --- | --- | --- | --- |
| GrG <=2 vs >=3 | 1 | 552487676 | 552487676 | 1,7047 | 0,1964 |
| Error | 63 | 2,0419e+10 | 324105601 |  |  |
| C. Total | 64 | 2,0971e+10 |  |  |  |

**Means for Oneway Anova**

| **Level** | **Number** | **Mean** | **Std Error** | **Lower 95%** | **Upper 95%** |
| --- | --- | --- | --- | --- | --- |
| 1/2 | 30 | 2244,50 | 3286,9 | -4324 | 8813 |
| 3/4&5 | 35 | 8092,71 | 3043,1 | 2012 | 14174 |

Std Error uses a pooled estimate of error variance

**Nonparametric Comparisons For Each Pair Using Wilcoxon Method**

| **q*** | **Alpha** |
| --- | --- |
| 1,95996 | 0,05 |

| **Level** | **- Level** | **Score Mean Difference** | **Std Err Dif** | **Z** | **p-Value** | **Hodges-Lehmann** | **Lower CL** | **Upper CL** | **Difference Plot** |
| --- | --- | --- | --- | --- | --- | --- | --- | --- | --- |
| 3/4&5 | 1/2 | 11,73095 | 4,704303 | 2,493664 | 0,0126* | 821,8453 | 111,9577 | 1555,201 |  |

**Oneway Analysis of original-shape-MajorAxis By GrG <=2 vs >=3**

**Quantiles**

| **Level** | **Minimum** | **10%** | **25%** | **Median** | **75%** | **90%** | **Maximum** |
| --- | --- | --- | --- | --- | --- | --- | --- |
| 1/2 | 9,100898 | 10,70117 | 15,09509 | 16,98917 | 19,86969 | 33,65837 | 53,20742 |
| 3/4&5 | 11,25113 | 13,15334 | 17,64516 | 20,47631 | 25,18065 | 43,8147 | 70,62249 |

**Oneway Anova**

**Summary of Fit**

| Rsquare | 0,047827 |
| --- | --- |
| Adj Rsquare | 0,032713 |
| Root Mean Square Error | 11,14882 |
| Mean of Response | 21,85179 |
| Observations (or Sum Wgts) | 65 |

**Pooled t Test**

3/4&5-1/2

Assuming equal variances

| Difference | 4,934 | t Ratio | 1,778896 |
| --- | --- | --- | --- |
| Std Err Dif | 2,774 | DF | 63 |
| Upper CL Dif | 10,478 | Prob > |t| | 0,0801 |
| Lower CL Dif | -0,609 | Prob > t | 0,0400* |
| Confidence | 0,95 | Prob < t | 0,9600 |

**Analysis of Variance**

| **Source** | **DF** | **Sum of Squares** | **Mean Square** | **F Ratio** | **Prob > F** |
| --- | --- | --- | --- | --- | --- |
| GrG <=2 vs >=3 | 1 | 393,3313 | 393,331 | 3,1645 | 0,0801 |
| Error | 63 | 7830,6553 | 124,296 |  |  |
| C. Total | 64 | 8223,9865 |  |  |  |

**Means for Oneway Anova**

| **Level** | **Number** | **Mean** | **Std Error** | **Lower 95%** | **Upper 95%** |
| --- | --- | --- | --- | --- | --- |
| 1/2 | 30 | 19,1948 | 2,0355 | 15,127 | 23,262 |
| 3/4&5 | 35 | 24,1292 | 1,8845 | 20,363 | 27,895 |

Std Error uses a pooled estimate of error variance

**Nonparametric Comparisons For Each Pair Using Wilcoxon Method**

| **q*** | **Alpha** |
| --- | --- |
| 1,95996 | 0,05 |

| **Level** | **- Level** | **Score Mean Difference** | **Std Err Dif** | **Z** | **p-Value** | **Hodges-Lehmann** | **Lower CL** | **Upper CL** | **Difference Plot** |
| --- | --- | --- | --- | --- | --- | --- | --- | --- | --- |
| 3/4&5 | 1/2 | 10,30714 | 4,704355 | 2,190979 | 0,0285* | 3,485792 | 0,6279036 | 6,592825 |  |

**Oneway Analysis of original-shape-SurfaceArea By GrG <=2 vs >=3**

**Quantiles**

| **Level** | **Minimum** | **10%** | **25%** | **Median** | **75%** | **90%** | **Maximum** |
| --- | --- | --- | --- | --- | --- | --- | --- |
| 1/2 | 210,0281 | 246,7 | 460,4152 | 782,2897 | 1267,331 | 4449,758 | 7651,818 |
| 3/4&5 | 213,7611 | 391,8274 | 649,3602 | 1357,833 | 1907,757 | 7639,359 | 33698,62 |

**Oneway Anova**

**Summary of Fit**

| Rsquare | 0,033141 |
| --- | --- |
| Adj Rsquare | 0,017794 |
| Root Mean Square Error | 4428,171 |
| Mean of Response | 2196,287 |
| Observations (or Sum Wgts) | 65 |

**Pooled t Test**

3/4&5-1/2

Assuming equal variances

| Difference | 1619,0 | t Ratio | 1,469513 |
| --- | --- | --- | --- |
| Std Err Dif | 1101,8 | DF | 63 |
| Upper CL Dif | 3820,7 | Prob > |t| | 0,1467 |
| Lower CL Dif | -582,6 | Prob > t | 0,0733 |
| Confidence | 0,95 | Prob < t | 0,9267 |

**Analysis of Variance**

| **Source** | **DF** | **Sum of Squares** | **Mean Square** | **F Ratio** | **Prob > F** |
| --- | --- | --- | --- | --- | --- |
| GrG <=2 vs >=3 | 1 | 42344367,6 | 42344368 | 2,1595 | 0,1467 |
| Error | 63 | 1235348110 | 19608700 |  |  |
| C. Total | 64 | 1277692477 |  |  |  |

**Means for Oneway Anova**

| **Level** | **Number** | **Mean** | **Std Error** | **Lower 95%** | **Upper 95%** |
| --- | --- | --- | --- | --- | --- |
| 1/2 | 30 | 1324,49 | 808,47 | -291 | 2940,1 |
| 3/4&5 | 35 | 2943,54 | 748,50 | 1448 | 4439,3 |

Std Error uses a pooled estimate of error variance

**Nonparametric Comparisons For Each Pair Using Wilcoxon Method**

| **q*** | **Alpha** |
| --- | --- |
| 1,95996 | 0,05 |

| **Level** | **- Level** | **Score Mean Difference** | **Std Err Dif** | **Z** | **p-Value** | **Hodges-Lehmann** | **Lower CL** | **Upper CL** | **Difference Plot** |
| --- | --- | --- | --- | --- | --- | --- | --- | --- | --- |
| 3/4&5 | 1/2 | 9,935714 | 4,704355 | 2,112025 | 0,0347* | 430,6889 | 22,12939 | 874,0939 |  |

**Oneway Analysis of original-shape-Flatness By GrG <=2 vs >=3**

**Quantiles**

| **Level** | **Minimum** | **10%** | **25%** | **Median** | **75%** | **90%** | **Maximum** |
| --- | --- | --- | --- | --- | --- | --- | --- |
| 1/2 | 0,306083 | 0,357687 | 0,392484 | 0,498422 | 0,634139 | 0,723536 | 0,770513 |
| 3/4&5 | 0,306632 | 0,360767 | 0,469866 | 0,525712 | 0,614884 | 0,716169 | 0,78413 |

**Oneway Anova**

**Summary of Fit**

| Rsquare | 0,011677 |
| --- | --- |
| Adj Rsquare | -0,00401 |
| Root Mean Square Error | 0,127114 |
| Mean of Response | 0,529672 |
| Observations (or Sum Wgts) | 65 |

**Pooled t Test**

3/4&5-1/2

Assuming equal variances

| Difference | 0,02729 | t Ratio | 0,86276 |
| --- | --- | --- | --- |
| Std Err Dif | 0,03163 | DF | 63 |
| Upper CL Dif | 0,09049 | Prob > |t| | 0,3915 |
| Lower CL Dif | -0,03591 | Prob > t | 0,1958 |
| Confidence | 0,95 | Prob < t | 0,8042 |

**Analysis of Variance**

| **Source** | **DF** | **Sum of Squares** | **Mean Square** | **F Ratio** | **Prob > F** |
| --- | --- | --- | --- | --- | --- |
| GrG <=2 vs >=3 | 1 | 0,0120272 | 0,012027 | 0,7444 | 0,3915 |
| Error | 63 | 1,0179487 | 0,016158 |  |  |
| C. Total | 64 | 1,0299759 |  |  |  |

**Means for Oneway Anova**

| **Level** | **Number** | **Mean** | **Std Error** | **Lower 95%** | **Upper 95%** |
| --- | --- | --- | --- | --- | --- |
| 1/2 | 30 | 0,514979 | 0,02321 | 0,46860 | 0,56136 |
| 3/4&5 | 35 | 0,542266 | 0,02149 | 0,49933 | 0,58520 |

Std Error uses a pooled estimate of error variance

**Nonparametric Comparisons For Each Pair Using Wilcoxon Method**

| **q*** | **Alpha** |
| --- | --- |
| 1,95996 | 0,05 |

| **Level** | **- Level** | **Score Mean Difference** | **Std Err Dif** | **Z** | **p-Value** | **Hodges-Lehmann** | **Lower CL** | **Upper CL** | **Difference Plot** |
| --- | --- | --- | --- | --- | --- | --- | --- | --- | --- |
| 3/4&5 | 1/2 | 4,178571 | 4,704355 | 0,8882348 | 0,3744 | 0,0342948 | -0,037251 | 0,1033792 |  |

**Oneway Analysis of original-shape-LeastAxis By GrG <=2 vs >=3**

**Quantiles**

| **Level** | **Minimum** | **10%** | **25%** | **Median** | **75%** | **90%** | **Maximum** |
| --- | --- | --- | --- | --- | --- | --- | --- |
| 1/2 | 4,164745 | 4,862808 | 6,999869 | 8,535103 | 10,49471 | 15,8391 | 17,74243 |
| 3/4&5 | 3,889939 | 6,935077 | 8,014138 | 11,30823 | 13,50098 | 27,30469 | 53,30986 |

**Oneway Anova**

**Summary of Fit**

| Rsquare | 0,074821 |
| --- | --- |
| Adj Rsquare | 0,060135 |
| Root Mean Square Error | 7,272148 |
| Mean of Response | 11,59251 |
| Observations (or Sum Wgts) | 65 |

**Pooled t Test**

3/4&5-1/2

Assuming equal variances

| Difference | 4,08407 | t Ratio | 2,257192 |
| --- | --- | --- | --- |
| Std Err Dif | 1,80936 | DF | 63 |
| Upper CL Dif | 7,69979 | Prob > |t| | 0,0275* |
| Lower CL Dif | 0,46836 | Prob > t | 0,0137* |
| Confidence | 0,95 | Prob < t | 0,9863 |

**Analysis of Variance**

| **Source** | **DF** | **Sum of Squares** | **Mean Square** | **F Ratio** | **Prob > F** |
| --- | --- | --- | --- | --- | --- |
| GrG <=2 vs >=3 | 1 | 269,4402 | 269,440 | 5,0949 | 0,0275* |
| Error | 63 | 3331,7002 | 52,884 |  |  |
| C. Total | 64 | 3601,1404 |  |  |  |

**Means for Oneway Anova**

| **Level** | **Number** | **Mean** | **Std Error** | **Lower 95%** | **Upper 95%** |
| --- | --- | --- | --- | --- | --- |
| 1/2 | 30 | 9,3934 | 1,3277 | 6,740 | 12,047 |
| 3/4&5 | 35 | 13,4775 | 1,2292 | 11,021 | 15,934 |

Std Error uses a pooled estimate of error variance

**Nonparametric Comparisons For Each Pair Using Wilcoxon Method**

| **q*** | **Alpha** |
| --- | --- |
| 1,95996 | 0,05 |

| **Level** | **- Level** | **Score Mean Difference** | **Std Err Dif** | **Z** | **p-Value** | **Hodges-Lehmann** | **Lower CL** | **Upper CL** | **Difference Plot** |
| --- | --- | --- | --- | --- | --- | --- | --- | --- | --- |
| 3/4&5 | 1/2 | 10,18333 | 4,704355 | 2,164661 | 0,0304* | 2,171108 | 0,2420413 | 3,940577 |  |

**Oneway Analysis of original-shape-Maximum2DDiameterColumn By GrG <=2 vs >=3**

**Quantiles**

| **Level** | **Minimum** | **10%** | **25%** | **Median** | **75%** | **90%** | **Maximum** |
| --- | --- | --- | --- | --- | --- | --- | --- |
| 1/2 | 6,434628 | 7,642567 | 13,62781 | 16,12317 | 19,79849 | 38,92102 | 56,46096 |
| 3/4&5 | 8,772684 | 11,25779 | 15,05723 | 20,32142 | 29,55342 | 44,21548 | 83,19999 |

**Oneway Anova**

**Summary of Fit**

| Rsquare | 0,036163 |
| --- | --- |
| Adj Rsquare | 0,020864 |
| Root Mean Square Error | 13,20827 |
| Mean of Response | 21,65605 |
| Observations (or Sum Wgts) | 65 |

**Pooled t Test**

3/4&5-1/2

Assuming equal variances

| Difference | 5,053 | t Ratio | 1,537442 |
| --- | --- | --- | --- |
| Std Err Dif | 3,286 | DF | 63 |
| Upper CL Dif | 11,620 | Prob > |t| | 0,1292 |
| Lower CL Dif | -1,515 | Prob > t | 0,0646 |
| Confidence | 0,95 | Prob < t | 0,9354 |

**Analysis of Variance**

| **Source** | **DF** | **Sum of Squares** | **Mean Square** | **F Ratio** | **Prob > F** |
| --- | --- | --- | --- | --- | --- |
| GrG <=2 vs >=3 | 1 | 412,372 | 412,372 | 2,3637 | 0,1292 |
| Error | 63 | 10990,873 | 174,458 |  |  |
| C. Total | 64 | 11403,244 |  |  |  |

**Means for Oneway Anova**

| **Level** | **Number** | **Mean** | **Std Error** | **Lower 95%** | **Upper 95%** |
| --- | --- | --- | --- | --- | --- |
| 1/2 | 30 | 18,9355 | 2,4115 | 14,116 | 23,754 |
| 3/4&5 | 35 | 23,9880 | 2,2326 | 19,526 | 28,449 |

Std Error uses a pooled estimate of error variance

**Nonparametric Comparisons For Each Pair Using Wilcoxon Method**

| **q*** | **Alpha** |
| --- | --- |
| 1,95996 | 0,05 |

| **Level** | **- Level** | **Score Mean Difference** | **Std Err Dif** | **Z** | **p-Value** | **Hodges-Lehmann** | **Lower CL** | **Upper CL** | **Difference Plot** |
| --- | --- | --- | --- | --- | --- | --- | --- | --- | --- |
| 3/4&5 | 1/2 | 8,326190 | 4,704200 | 1,769948 | 0,0767 | 3,659389 | -0,391133 | 7,661634 |  |

**neway Analysis of original-shape-Maximum2DDiameterRow By GrG <=2 vs >=3**

**Quantiles**

| **Level** | **Minimum** | **10%** | **25%** | **Median** | **75%** | **90%** | **Maximum** |
| --- | --- | --- | --- | --- | --- | --- | --- |
| 1/2 | 8 | 8,960157 | 10,7629 | 15,00122 | 19,1189 | 35,4655 | 37 |
| 3/4&5 | 4,480078 | 10,40142 | 14,03183 | 17,15862 | 24,20918 | 49,14974 | 81,27035 |

**Oneway Anova**

**Summary of Fit**

| Rsquare | 0,052846 |
| --- | --- |
| Adj Rsquare | 0,037811 |
| Root Mean Square Error | 12,75046 |
| Mean of Response | 19,80809 |
| Observations (or Sum Wgts) | 65 |

**Pooled t Test**

3/4&5-1/2

Assuming equal variances

| Difference | 5,948 | t Ratio | 1,874841 |
| --- | --- | --- | --- |
| Std Err Dif | 3,172 | DF | 63 |
| Upper CL Dif | 12,287 | Prob > |t| | 0,0655 |
| Lower CL Dif | -0,392 | Prob > t | 0,0327* |
| Confidence | 0,95 | Prob < t | 0,9673 |

**Analysis of Variance**

| **Source** | **DF** | **Sum of Squares** | **Mean Square** | **F Ratio** | **Prob > F** |
| --- | --- | --- | --- | --- | --- |
| GrG <=2 vs >=3 | 1 | 571,453 | 571,453 | 3,5150 | 0,0655 |
| Error | 63 | 10242,184 | 162,574 |  |  |
| C. Total | 64 | 10813,638 |  |  |  |

**Means for Oneway Anova**

| **Level** | **Number** | **Mean** | **Std Error** | **Lower 95%** | **Upper 95%** |
| --- | --- | --- | --- | --- | --- |
| 1/2 | 30 | 16,6055 | 2,3279 | 11,954 | 21,257 |
| 3/4&5 | 35 | 22,5532 | 2,1552 | 18,246 | 26,860 |

Std Error uses a pooled estimate of error variance

**Nonparametric Comparisons For Each Pair Using Wilcoxon Method**

| **q*** | **Alpha** |
| --- | --- |
| 1,95996 | 0,05 |

| **Level** | **- Level** | **Score Mean Difference** | **Std Err Dif** | **Z** | **p-Value** | **Hodges-Lehmann** | **Lower CL** | **Upper CL** | **Difference Plot** |
| --- | --- | --- | --- | --- | --- | --- | --- | --- | --- |
| 3/4&5 | 1/2 | 8,945238 | 4,703995 | 1,901626 | 0,0572 | 3,231390 | -0,077398 | 6,666667 |  |

**Oneway Analysis of original-gldm-GrayLevelVariance By GrG <=2 vs >=3**

**Quantiles**

| **Level** | **Minimum** | **10%** | **25%** | **Median** | **75%** | **90%** | **Maximum** |
| --- | --- | --- | --- | --- | --- | --- | --- |
| 1/2 | 0,636487 | 28,91289 | 37,59633 | 52,04618 | 72,18462 | 119,9811 | 168,7429 |
| 3/4&5 | 23,35417 | 24,97875 | 37,14081 | 51,02718 | 80,98606 | 124,7732 | 187,0951 |

**Oneway Anova**

**Summary of Fit**

| Rsquare | 0,00182 |
| --- | --- |
| Adj Rsquare | -0,01402 |
| Root Mean Square Error | 38,09468 |
| Mean of Response | 61,8405 |
| Observations (or Sum Wgts) | 65 |

**Pooled t Test**

3/4&5-1/2

Assuming equal variances

| Difference | 3,212 | t Ratio | 0,338934 |
| --- | --- | --- | --- |
| Std Err Dif | 9,478 | DF | 63 |
| Upper CL Dif | 22,153 | Prob > |t| | 0,7358 |
| Lower CL Dif | -15,728 | Prob > t | 0,3679 |
| Confidence | 0,95 | Prob < t | 0,6321 |

**Analysis of Variance**

| **Source** | **DF** | **Sum of Squares** | **Mean Square** | **F Ratio** | **Prob > F** |
| --- | --- | --- | --- | --- | --- |
| GrG <=2 vs >=3 | 1 | 166,709 | 166,71 | 0,1149 | 0,7358 |
| Error | 63 | 91425,873 | 1451,20 |  |  |
| C. Total | 64 | 91592,582 |  |  |  |

**Means for Oneway Anova**

| **Level** | **Number** | **Mean** | **Std Error** | **Lower 95%** | **Upper 95%** |
| --- | --- | --- | --- | --- | --- |
| 1/2 | 30 | 60,1107 | 6,9551 | 46,212 | 74,009 |
| 3/4&5 | 35 | 63,3232 | 6,4392 | 50,456 | 76,191 |

Std Error uses a pooled estimate of error variance

**Nonparametric Comparisons For Each Pair Using Wilcoxon Method**

| **q*** | **Alpha** |
| --- | --- |
| 1,95996 | 0,05 |

| **Level** | **- Level** | **Score Mean Difference** | **Std Err Dif** | **Z** | **p-Value** | **Hodges-Lehmann** | **Lower CL** | **Upper CL** | **Difference Plot** |
| --- | --- | --- | --- | --- | --- | --- | --- | --- | --- |
| 3/4&5 | 1/2 | 0,2166667 | 4,704355 | 0,0460566 | 0,9633 | 0,1955243 | -13,5047 | 14,68427 |  |

**Oneway Analysis of original-gldm-HighGrayLevelEmphasis By GrG <=2 vs >=3**

**Quantiles**

| **Level** | **Minimum** | **10%** | **25%** | **Median** | **75%** | **90%** | **Maximum** |
| --- | --- | --- | --- | --- | --- | --- | --- |
| 1/2 | 4,468085 | 165,5339 | 228,0258 | 450,5519 | 759,9355 | 1025,716 | 1115,486 |
| 3/4&5 | 146,2632 | 203,8684 | 269,1352 | 391,3647 | 701,5 | 1168,688 | 2059,788 |

**Oneway Anova**

**Summary of Fit**

| Rsquare | 0,004339 |
| --- | --- |
| Adj Rsquare | -0,01146 |
| Root Mean Square Error | 364,552 |
| Mean of Response | 526,8917 |
| Observations (or Sum Wgts) | 65 |

**Pooled t Test**

3/4&5-1/2

Assuming equal variances

| Difference | 47,53 | t Ratio | 0,524001 |
| --- | --- | --- | --- |
| Std Err Dif | 90,70 | DF | 63 |
| Upper CL Dif | 228,78 | Prob > |t| | 0,6021 |
| Lower CL Dif | -133,73 | Prob > t | 0,3011 |
| Confidence | 0,95 | Prob < t | 0,6989 |

**Analysis of Variance**

| **Source** | **DF** | **Sum of Squares** | **Mean Square** | **F Ratio** | **Prob > F** |
| --- | --- | --- | --- | --- | --- |
| GrG <=2 vs >=3 | 1 | 36490,7 | 36491 | 0,2746 | 0,6021 |
| Error | 63 | 8372582,6 | 132898 |  |  |
| C. Total | 64 | 8409073,4 |  |  |  |

**Means for Oneway Anova**

| **Level** | **Number** | **Mean** | **Std Error** | **Lower 95%** | **Upper 95%** |
| --- | --- | --- | --- | --- | --- |
| 1/2 | 30 | 501,299 | 66,558 | 368,29 | 634,30 |
| 3/4&5 | 35 | 548,828 | 61,621 | 425,69 | 671,97 |

Std Error uses a pooled estimate of error variance

**Nonparametric Comparisons For Each Pair Using Wilcoxon Method**

| **q*** | **Alpha** |
| --- | --- |
| 1,95996 | 0,05 |

| **Level** | **- Level** | **Score Mean Difference** | **Std Err Dif** | **Z** | **p-Value** | **Hodges-Lehmann** | **Lower CL** | **Upper CL** | **Difference Plot** |
| --- | --- | --- | --- | --- | --- | --- | --- | --- | --- |
| 3/4&5 | 1/2 | 0,8357143 | 4,704355 | 0,1776470 | 0,8590 | 21,40745 | -136,581 | 154,7275 |  |

**Oneway Analysis of original-gldm-DependenceEntropy By GrG <=2 vs >=3**

**Quantiles**

| **Level** | **Minimum** | **10%** | **25%** | **Median** | **75%** | **90%** | **Maximum** |
| --- | --- | --- | --- | --- | --- | --- | --- |
| 1/2 | 4,06526 | 4,432529 | 5,185696 | 5,765954 | 6,089135 | 6,820077 | 7,016533 |
| 3/4&5 | 4,164498 | 4,98239 | 5,385632 | 6,104047 | 6,494931 | 6,972174 | 7,573548 |

**Oneway Anova**

**Summary of Fit**

| Rsquare | 0,055971 |
| --- | --- |
| Adj Rsquare | 0,040986 |
| Root Mean Square Error | 0,771562 |
| Mean of Response | 5,830784 |
| Observations (or Sum Wgts) | 65 |

**Pooled t Test**

3/4&5-1/2

Assuming equal variances

| Difference | 0,37101 | t Ratio | 1,932671 |
| --- | --- | --- | --- |
| Std Err Dif | 0,19197 | DF | 63 |
| Upper CL Dif | 0,75464 | Prob > |t| | 0,0578 |
| Lower CL Dif | -0,01261 | Prob > t | 0,0289* |
| Confidence | 0,95 | Prob < t | 0,9711 |

**Analysis of Variance**

| **Source** | **DF** | **Sum of Squares** | **Mean Square** | **F Ratio** | **Prob > F** |
| --- | --- | --- | --- | --- | --- |
| GrG <=2 vs >=3 | 1 | 2,223604 | 2,22360 | 3,7352 | 0,0578 |
| Error | 63 | 37,504391 | 0,59531 |  |  |
| C. Total | 64 | 39,727995 |  |  |  |

**Means for Oneway Anova**

| **Level** | **Number** | **Mean** | **Std Error** | **Lower 95%** | **Upper 95%** |
| --- | --- | --- | --- | --- | --- |
| 1/2 | 30 | 5,63101 | 0,14087 | 5,3495 | 5,9125 |
| 3/4&5 | 35 | 6,00202 | 0,13042 | 5,7414 | 6,2626 |

Std Error uses a pooled estimate of error variance

**Nonparametric Comparisons For Each Pair Using Wilcoxon Method**

| **q*** | **Alpha** |
| --- | --- |
| 1,95996 | 0,05 |

| **Level** | **- Level** | **Score Mean Difference** | **Std Err Dif** | **Z** | **p-Value** | **Hodges-Lehmann** | **Lower CL** | **Upper CL** | **Difference Plot** |
| --- | --- | --- | --- | --- | --- | --- | --- | --- | --- |
| 3/4&5 | 1/2 | 9,564286 | 4,704355 | 2,033071 | 0,0420* | 0,3924687 | 0,0205741 | 0,7691855 |  |

**Oneway Analysis of original-gldm-DependenceNonUniformity By GrG <=2 vs >=3**

**Quantiles**

| **Level** | **Minimum** | **10%** | **25%** | **Median** | **75%** | **90%** | **Maximum** |
| --- | --- | --- | --- | --- | --- | --- | --- |
| 1/2 | 6,914894 | 14,30967 | 30,63235 | 45,15952 | 96,92934 | 369,4523 | 788,9354 |
| 3/4&5 | 11 | 24,85476 | 39,09009 | 102,8333 | 163,1087 | 833,1968 | 4518,518 |

**Oneway Anova**

**Summary of Fit**

| Rsquare | 0,029435 |
| --- | --- |
| Adj Rsquare | 0,014029 |
| Root Mean Square Error | 578,8821 |
| Mean of Response | 211,6651 |
| Observations (or Sum Wgts) | 65 |

**Pooled t Test**

3/4&5-1/2

Assuming equal variances

| Difference | 199,09 | t Ratio | 1,382254 |
| --- | --- | --- | --- |
| Std Err Dif | 144,03 | DF | 63 |
| Upper CL Dif | 486,91 | Prob > |t| | 0,1718 |
| Lower CL Dif | -88,73 | Prob > t | 0,0859 |
| Confidence | 0,95 | Prob < t | 0,9141 |

**Analysis of Variance**

| **Source** | **DF** | **Sum of Squares** | **Mean Square** | **F Ratio** | **Prob > F** |
| --- | --- | --- | --- | --- | --- |
| GrG <=2 vs >=3 | 1 | 640259 | 640259 | 1,9106 | 0,1718 |
| Error | 63 | 21111586 | 335105 |  |  |
| C. Total | 64 | 21751845 |  |  |  |

**Means for Oneway Anova**

| **Level** | **Number** | **Mean** | **Std Error** | **Lower 95%** | **Upper 95%** |
| --- | --- | --- | --- | --- | --- |
| 1/2 | 30 | 104,465 | 105,69 | -106,7 | 315,67 |
| 3/4&5 | 35 | 303,551 | 97,85 | 108,0 | 499,09 |

Std Error uses a pooled estimate of error variance

**Nonparametric Comparisons For Each Pair Using Wilcoxon Method**

| **q*** | **Alpha** |
| --- | --- |
| 1,95996 | 0,05 |

| **Level** | **- Level** | **Score Mean Difference** | **Std Err Dif** | **Z** | **p-Value** | **Hodges-Lehmann** | **Lower CL** | **Upper CL** | **Difference Plot** |
| --- | --- | --- | --- | --- | --- | --- | --- | --- | --- |
| 3/4&5 | 1/2 | 10,36905 | 4,704355 | 2,204138 | 0,0275* | 33,00868 | 1,896491 | 75,68600 |  |

**Oneway Analysis of original-gldm-GrayLevelNonUniformity By GrG <=2 vs >=3**

**Quantiles**

| **Level** | **Minimum** | **10%** | **25%** | **Median** | **75%** | **90%** | **Maximum** |
| --- | --- | --- | --- | --- | --- | --- | --- |
| 1/2 | 2,836735 | 3,414706 | 4,757228 | 5,936602 | 15,31806 | 48,39409 | 104,2085 |
| 3/4&5 | 2,5 | 3,647647 | 6,837838 | 11,06667 | 22,78049 | 105,874 | 430,8082 |

**Oneway Anova**

**Summary of Fit**

| Rsquare | 0,032179 |
| --- | --- |
| Adj Rsquare | 0,016817 |
| Root Mean Square Error | 58,11881 |
| Mean of Response | 26,44147 |
| Observations (or Sum Wgts) | 65 |

**Pooled t Test**

3/4&5-1/2

Assuming equal variances

| Difference | 20,929 | t Ratio | 1,447303 |
| --- | --- | --- | --- |
| Std Err Dif | 14,460 | DF | 63 |
| Upper CL Dif | 49,825 | Prob > |t| | 0,1528 |
| Lower CL Dif | -7,968 | Prob > t | 0,0764 |
| Confidence | 0,95 | Prob < t | 0,9236 |

**Analysis of Variance**

| **Source** | **DF** | **Sum of Squares** | **Mean Square** | **F Ratio** | **Prob > F** |
| --- | --- | --- | --- | --- | --- |
| GrG <=2 vs >=3 | 1 | 7075,42 | 7075,42 | 2,0947 | 0,1528 |
| Error | 63 | 212801,16 | 3377,80 |  |  |
| C. Total | 64 | 219876,58 |  |  |  |

**Means for Oneway Anova**

| **Level** | **Number** | **Mean** | **Std Error** | **Lower 95%** | **Upper 95%** |
| --- | --- | --- | --- | --- | --- |
| 1/2 | 30 | 15,1723 | 10,611 | -6,03 | 36,377 |
| 3/4&5 | 35 | 36,1008 | 9,824 | 16,47 | 55,732 |

Std Error uses a pooled estimate of error variance

**Nonparametric Comparisons For Each Pair Using Wilcoxon Method**

| **q*** | **Alpha** |
| --- | --- |
| 1,95996 | 0,05 |

| **Level** | **- Level** | **Score Mean Difference** | **Std Err Dif** | **Z** | **p-Value** | **Hodges-Lehmann** | **Lower CL** | **Upper CL** | **Difference Plot** |
| --- | --- | --- | --- | --- | --- | --- | --- | --- | --- |
| 3/4&5 | 1/2 | 8,604762 | 4,704303 | 1,829126 | 0,0674 | 3,390089 | -0,277778 | 8,474341 |  |

**Oneway Analysis of original-gldm-SmallDependenceEmphasis By GrG <=2 vs >=3**

**Quantiles**

| **Level** | **Minimum** | **10%** | **25%** | **Median** | **75%** | **90%** | **Maximum** |
| --- | --- | --- | --- | --- | --- | --- | --- |
| 1/2 | 0,063013 | 0,438837 | 0,460413 | 0,512591 | 0,627451 | 0,67764 | 0,714994 |
| 3/4&5 | 0,423587 | 0,4446 | 0,470807 | 0,525828 | 0,578706 | 0,628189 | 0,663294 |

**Oneway Anova**

**Summary of Fit**

| Rsquare | 0,000202 |
| --- | --- |
| Adj Rsquare | -0,01567 |
| Root Mean Square Error | 0,097442 |
| Mean of Response | 0,528173 |
| Observations (or Sum Wgts) | 65 |

**Pooled t Test**

3/4&5-1/2

Assuming equal variances

| Difference | -0,00274 | t Ratio | -0,11282 |
| --- | --- | --- | --- |
| Std Err Dif | 0,02424 | DF | 63 |
| Upper CL Dif | 0,04571 | Prob > |t| | 0,9105 |
| Lower CL Dif | -0,05118 | Prob > t | 0,5447 |
| Confidence | 0,95 | Prob < t | 0,4553 |

**Analysis of Variance**

| **Source** | **DF** | **Sum of Squares** | **Mean Square** | **F Ratio** | **Prob > F** |
| --- | --- | --- | --- | --- | --- |
| GrG <=2 vs >=3 | 1 | 0,00012085 | 0,000121 | 0,0127 | 0,9105 |
| Error | 63 | 0,59817727 | 0,009495 |  |  |
| C. Total | 64 | 0,59829812 |  |  |  |

**Means for Oneway Anova**

| **Level** | **Number** | **Mean** | **Std Error** | **Lower 95%** | **Upper 95%** |
| --- | --- | --- | --- | --- | --- |
| 1/2 | 30 | 0,529645 | 0,01779 | 0,49409 | 0,56520 |
| 3/4&5 | 35 | 0,526910 | 0,01647 | 0,49400 | 0,55982 |

Std Error uses a pooled estimate of error variance

**Nonparametric Comparisons For Each Pair Using Wilcoxon Method**

| **q*** | **Alpha** |
| --- | --- |
| 1,95996 | 0,05 |

| **Level** | **- Level** | **Score Mean Difference** | **Std Err Dif** | **Z** | **p-Value** | **Hodges-Lehmann** | **Lower CL** | **Upper CL** | **Difference Plot** |
| --- | --- | --- | --- | --- | --- | --- | --- | --- | --- |
| 3/4&5 | 1/2 | -2,50714 | 4,704355 | -0,532941 | 0,5941 | -0,008898 | -0,051456 | 0,0317347 |  |

**Oneway Analysis of original-gldm-SmallDependenceHighGrayLevelEmphasis By GrG <=2 vs >=3**

**Quantiles**

| **Level** | **Minimum** | **10%** | **25%** | **Median** | **75%** | **90%** | **Maximum** |
| --- | --- | --- | --- | --- | --- | --- | --- |
| 1/2 | 0,4792 | 92,95831 | 127,8209 | 242,519 | 442,8038 | 531,7102 | 762,8201 |
| 3/4&5 | 63,74049 | 101,0191 | 150,3631 | 223,5505 | 367,718 | 609,4012 | 1097,426 |

**Oneway Anova**

**Summary of Fit**

| Rsquare | 0,002226 |
| --- | --- |
| Adj Rsquare | -0,01361 |
| Root Mean Square Error | 209,6375 |
| Mean of Response | 292,7228 |
| Observations (or Sum Wgts) | 65 |

**Pooled t Test**

3/4&5-1/2

Assuming equal variances

| Difference | 19,55 | t Ratio | 0,374883 |
| --- | --- | --- | --- |
| Std Err Dif | 52,16 | DF | 63 |
| Upper CL Dif | 123,79 | Prob > |t| | 0,7090 |
| Lower CL Dif | -84,68 | Prob > t | 0,3545 |
| Confidence | 0,95 | Prob < t | 0,6455 |

**Analysis of Variance**

| **Source** | **DF** | **Sum of Squares** | **Mean Square** | **F Ratio** | **Prob > F** |
| --- | --- | --- | --- | --- | --- |
| GrG <=2 vs >=3 | 1 | 6176,3 | 6176,3 | 0,1405 | 0,7090 |
| Error | 63 | 2768716,0 | 43947,9 |  |  |
| C. Total | 64 | 2774892,3 |  |  |  |

**Means for Oneway Anova**

| **Level** | **Number** | **Mean** | **Std Error** | **Lower 95%** | **Upper 95%** |
| --- | --- | --- | --- | --- | --- |
| 1/2 | 30 | 282,194 | 38,274 | 205,71 | 358,68 |
| 3/4&5 | 35 | 301,748 | 35,435 | 230,94 | 372,56 |

Std Error uses a pooled estimate of error variance

**Nonparametric Comparisons For Each Pair Using Wilcoxon Method**

| **q*** | **Alpha** |
| --- | --- |
| 1,95996 | 0,05 |

| **Level** | **- Level** | **Score Mean Difference** | **Std Err Dif** | **Z** | **p-Value** | **Hodges-Lehmann** | **Lower CL** | **Upper CL** | **Difference Plot** |
| --- | --- | --- | --- | --- | --- | --- | --- | --- | --- |
| 3/4&5 | 1/2 | 0,5261905 | 4,704355 | 0,1118518 | 0,9109 | 3,648778 | -81,4452 | 79,88290 |  |

**Oneway Analysis of original-gldm-DependenceNonUniformityNormalized By GrG <=2 vs >=3**

**Quantiles**

| **Level** | **Minimum** | **10%** | **25%** | **Median** | **75%** | **90%** | **Maximum** |
| --- | --- | --- | --- | --- | --- | --- | --- |
| 1/2 | 0,147125 | 0,265464 | 0,287589 | 0,33242 | 0,410265 | 0,448624 | 0,496043 |
| 3/4&5 | 0,250873 | 0,272289 | 0,287474 | 0,323529 | 0,362838 | 0,403734 | 0,422832 |

**Oneway Anova**

**Summary of Fit**

| Rsquare | 0,016677 |
| --- | --- |
| Adj Rsquare | 0,001068 |
| Root Mean Square Error | 0,062703 |
| Mean of Response | 0,33587 |
| Observations (or Sum Wgts) | 65 |

**Pooled t Test**

3/4&5-1/2

Assuming equal variances

| Difference | -0,01613 | t Ratio | -1,03366 |
| --- | --- | --- | --- |
| Std Err Dif | 0,01560 | DF | 63 |
| Upper CL Dif | 0,01505 | Prob > |t| | 0,3052 |
| Lower CL Dif | -0,04730 | Prob > t | 0,8474 |
| Confidence | 0,95 | Prob < t | 0,1526 |

**Analysis of Variance**

| **Source** | **DF** | **Sum of Squares** | **Mean Square** | **F Ratio** | **Prob > F** |
| --- | --- | --- | --- | --- | --- |
| GrG <=2 vs >=3 | 1 | 0,00420074 | 0,004201 | 1,0684 | 0,3052 |
| Error | 63 | 0,24769278 | 0,003932 |  |  |
| C. Total | 64 | 0,25189352 |  |  |  |

**Means for Oneway Anova**

| **Level** | **Number** | **Mean** | **Std Error** | **Lower 95%** | **Upper 95%** |
| --- | --- | --- | --- | --- | --- |
| 1/2 | 30 | 0,344553 | 0,01145 | 0,32168 | 0,36743 |
| 3/4&5 | 35 | 0,328427 | 0,01060 | 0,30725 | 0,34961 |

Std Error uses a pooled estimate of error variance

**Nonparametric Comparisons For Each Pair Using Wilcoxon Method**

| **q*** | **Alpha** |
| --- | --- |
| 1,95996 | 0,05 |

| **Level** | **- Level** | **Score Mean Difference** | **Std Err Dif** | **Z** | **p-Value** | **Hodges-Lehmann** | **Lower CL** | **Upper CL** | **Difference Plot** |
| --- | --- | --- | --- | --- | --- | --- | --- | --- | --- |
| 3/4&5 | 1/2 | -4,92143 | 4,704355 | -1,04614 | 0,2955 | -0,014120 | -0,045654 | 0,0145991 |  |

**Oneway Analysis of original-gldm-LargeDependenceEmphasis By GrG <=2 vs >=3**

**Quantiles**

| **Level** | **Minimum** | **10%** | **25%** | **Median** | **75%** | **90%** | **Maximum** |
| --- | --- | --- | --- | --- | --- | --- | --- |
| 1/2 | 2,55102 | 2,859314 | 3,552083 | 4,540834 | 5,849987 | 6,294292 | 39,46809 |
| 3/4&5 | 3,072289 | 3,410975 | 3,857143 | 4,961905 | 5,696913 | 6,435063 | 7,104848 |

**Oneway Anova**

**Summary of Fit**

| Rsquare | 0,009142 |
| --- | --- |
| Adj Rsquare | -0,00659 |
| Root Mean Square Error | 4,471686 |
| Mean of Response | 5,269953 |
| Observations (or Sum Wgts) | 65 |

**Pooled t Test**

3/4&5-1/2

Assuming equal variances

| Difference | -0,8482 | t Ratio | -0,7624 |
| --- | --- | --- | --- |
| Std Err Dif | 1,1126 | DF | 63 |
| Upper CL Dif | 1,3751 | Prob > |t| | 0,4487 |
| Lower CL Dif | -3,0716 | Prob > t | 0,7757 |
| Confidence | 0,95 | Prob < t | 0,2243 |

**Analysis of Variance**

| **Source** | **DF** | **Sum of Squares** | **Mean Square** | **F Ratio** | **Prob > F** |
| --- | --- | --- | --- | --- | --- |
| GrG <=2 vs >=3 | 1 | 11,6228 | 11,6228 | 0,5813 | 0,4487 |
| Error | 63 | 1259,7465 | 19,9960 |  |  |
| C. Total | 64 | 1271,3693 |  |  |  |

**Means for Oneway Anova**

| **Level** | **Number** | **Mean** | **Std Error** | **Lower 95%** | **Upper 95%** |
| --- | --- | --- | --- | --- | --- |
| 1/2 | 30 | 5,72670 | 0,81641 | 4,0952 | 7,3582 |
| 3/4&5 | 35 | 4,87846 | 0,75585 | 3,3680 | 6,3889 |

Std Error uses a pooled estimate of error variance

**Nonparametric Comparisons For Each Pair Using Wilcoxon Method**

| **q*** | **Alpha** |
| --- | --- |
| 1,95996 | 0,05 |

| **Level** | **- Level** | **Score Mean Difference** | **Std Err Dif** | **Z** | **p-Value** | **Hodges-Lehmann** | **Lower CL** | **Upper CL** | **Difference Plot** |
| --- | --- | --- | --- | --- | --- | --- | --- | --- | --- |
| 3/4&5 | 1/2 | 3,930952 | 4,704355 | 0,8355987 | 0,4034 | 0,2455109 | -0,438722 | 0,8986440 |  |

**Oneway Analysis of original-gldm-LargeDependenceLowGrayLevelEmphasis By GrG <=2 vs >=3**

**Quantiles**

| **Level** | **Minimum** | **10%** | **25%** | **Median** | **75%** | **90%** | **Maximum** |
| --- | --- | --- | --- | --- | --- | --- | --- |
| 1/2 | 0,008983 | 0,010571 | 0,014947 | 0,028759 | 0,073403 | 0,560722 | 14,01079 |
| 3/4&5 | 0,005259 | 0,008738 | 0,016732 | 0,03327 | 0,068029 | 0,111842 | 0,243848 |

**Oneway Anova**

**Summary of Fit**

| Rsquare | 0,023811 |
| --- | --- |
| Adj Rsquare | 0,008316 |
| Root Mean Square Error | 1,73444 |
| Mean of Response | 0,295155 |
| Observations (or Sum Wgts) | 65 |

**Pooled t Test**

3/4&5-1/2

Assuming equal variances

| Difference | -0,5350 | t Ratio | -1,23963 |
| --- | --- | --- | --- |
| Std Err Dif | 0,4315 | DF | 63 |
| Upper CL Dif | 0,3274 | Prob > |t| | 0,2197 |
| Lower CL Dif | -1,3973 | Prob > t | 0,8901 |
| Confidence | 0,95 | Prob < t | 0,1099 |

**Analysis of Variance**

| **Source** | **DF** | **Sum of Squares** | **Mean Square** | **F Ratio** | **Prob > F** |
| --- | --- | --- | --- | --- | --- |
| GrG <=2 vs >=3 | 1 | 4,62280 | 4,62280 | 1,5367 | 0,2197 |
| Error | 63 | 189,52169 | 3,00828 |  |  |
| C. Total | 64 | 194,14450 |  |  |  |

**Means for Oneway Anova**

| **Level** | **Number** | **Mean** | **Std Error** | **Lower 95%** | **Upper 95%** |
| --- | --- | --- | --- | --- | --- |
| 1/2 | 30 | 0,583206 | 0,31666 | -0,0496 | 1,2160 |
| 3/4&5 | 35 | 0,048255 | 0,29317 | -0,5376 | 0,6341 |

Std Error uses a pooled estimate of error variance

**Nonparametric Comparisons For Each Pair Using Wilcoxon Method**

| **q*** | **Alpha** |
| --- | --- |
| 1,95996 | 0,05 |

| **Level** | **- Level** | **Score Mean Difference** | **Std Err Dif** | **Z** | **p-Value** | **Hodges-Lehmann** | **Lower CL** | **Upper CL** | **Difference Plot** |
| --- | --- | --- | --- | --- | --- | --- | --- | --- | --- |
| 3/4&5 | 1/2 | -1,76429 | 4,704355 | -0,375032 | 0,7076 | -0,003197 | -0,017578 | 0,0113410 |  |

**Oneway Analysis of original-gldm-DependenceVariance By GrG <=2 vs >=3**

**Quantiles**

| **Level** | **Minimum** | **10%** | **25%** | **Median** | **75%** | **90%** | **Maximum** |
| --- | --- | --- | --- | --- | --- | --- | --- |
| 1/2 | 0,409722 | 0,457437 | 0,724537 | 0,893983 | 1,29972 | 1,550546 | 4,229968 |
| 3/4&5 | 0,504573 | 0,596448 | 0,830939 | 1,046308 | 1,291879 | 1,547725 | 1,685829 |

**Oneway Anova**

**Summary of Fit**

| Rsquare | 0,000144 |
| --- | --- |
| Adj Rsquare | -0,01573 |
| Root Mean Square Error | 0,529236 |
| Mean of Response | 1,071583 |
| Observations (or Sum Wgts) | 65 |

**Pooled t Test**

3/4&5-1/2

Assuming equal variances

| Difference | -0,01252 | t Ratio | -0,09511 |
| --- | --- | --- | --- |
| Std Err Dif | 0,13168 | DF | 63 |
| Upper CL Dif | 0,25061 | Prob > |t| | 0,9245 |
| Lower CL Dif | -0,27566 | Prob > t | 0,5377 |
| Confidence | 0,95 | Prob < t | 0,4623 |

**Analysis of Variance**

| **Source** | **DF** | **Sum of Squares** | **Mean Square** | **F Ratio** | **Prob > F** |
| --- | --- | --- | --- | --- | --- |
| GrG <=2 vs >=3 | 1 | 0,002534 | 0,002534 | 0,0090 | 0,9245 |
| Error | 63 | 17,645723 | 0,280091 |  |  |
| C. Total | 64 | 17,648257 |  |  |  |

**Means for Oneway Anova**

| **Level** | **Number** | **Mean** | **Std Error** | **Lower 95%** | **Upper 95%** |
| --- | --- | --- | --- | --- | --- |
| 1/2 | 30 | 1,07833 | 0,09662 | 0,88524 | 1,2714 |
| 3/4&5 | 35 | 1,06580 | 0,08946 | 0,88704 | 1,2446 |

Std Error uses a pooled estimate of error variance

**Nonparametric Comparisons For Each Pair Using Wilcoxon Method**

| **q*** | **Alpha** |
| --- | --- |
| 1,95996 | 0,05 |

| **Level** | **- Level** | **Score Mean Difference** | **Std Err Dif** | **Z** | **p-Value** | **Hodges-Lehmann** | **Lower CL** | **Upper CL** | **Difference Plot** |
| --- | --- | --- | --- | --- | --- | --- | --- | --- | --- |
| 3/4&5 | 1/2 | 3,869048 | 4,704355 | 0,8224396 | 0,4108 | 0,0901267 | -0,126412 | 0,2534032 |  |

**Oneway Analysis of original-gldm-LargeDependenceHighGrayLevelEmphasis By GrG <=2 vs >=3**

**Quantiles**

| **Level** | **Minimum** | **10%** | **25%** | **Median** | **75%** | **90%** | **Maximum** |
| --- | --- | --- | --- | --- | --- | --- | --- |
| 1/2 | 197,234 | 481,5816 | 1063,161 | 1579,065 | 2857,565 | 5488,163 | 6274,986 |
| 3/4&5 | 717,0278 | 899,1498 | 1146,638 | 1930,821 | 3401,117 | 4975,836 | 10906,75 |

**Oneway Anova**

**Summary of Fit**

| Rsquare | 0,009435 |
| --- | --- |
| Adj Rsquare | -0,00629 |
| Root Mean Square Error | 1869,225 |
| Mean of Response | 2372,473 |
| Observations (or Sum Wgts) | 65 |

**Pooled t Test**

3/4&5-1/2

Assuming equal variances

| Difference | 360,3 | t Ratio | 0,774656 |
| --- | --- | --- | --- |
| Std Err Dif | 465,1 | DF | 63 |
| Upper CL Dif | 1289,7 | Prob > |t| | 0,4414 |
| Lower CL Dif | -569,1 | Prob > t | 0,2207 |
| Confidence | 0,95 | Prob < t | 0,7793 |

**Analysis of Variance**

| **Source** | **DF** | **Sum of Squares** | **Mean Square** | **F Ratio** | **Prob > F** |
| --- | --- | --- | --- | --- | --- |
| GrG <=2 vs >=3 | 1 | 2096724 | 2096724 | 0,6001 | 0,4414 |
| Error | 63 | 220122048 | 3494001 |  |  |
| C. Total | 64 | 222218772 |  |  |  |

**Means for Oneway Anova**

| **Level** | **Number** | **Mean** | **Std Error** | **Lower 95%** | **Upper 95%** |
| --- | --- | --- | --- | --- | --- |
| 1/2 | 30 | 2178,48 | 341,27 | 1496,5 | 2860,5 |
| 3/4&5 | 35 | 2538,75 | 315,96 | 1907,4 | 3170,1 |

Std Error uses a pooled estimate of error variance

**Nonparametric Comparisons For Each Pair Using Wilcoxon Method**

| **q*** | **Alpha** |
| --- | --- |
| 1,95996 | 0,05 |

| **Level** | **- Level** | **Score Mean Difference** | **Std Err Dif** | **Z** | **p-Value** | **Hodges-Lehmann** | **Lower CL** | **Upper CL** | **Difference Plot** |
| --- | --- | --- | --- | --- | --- | --- | --- | --- | --- |
| 3/4&5 | 1/2 | 3,002381 | 4,704355 | 0,6382131 | 0,5233 | 218,6933 | -359,751 | 859,0469 |  |

**Oneway Analysis of original-gldm-SmallDependenceLowGrayLevelEmphasis By GrG <=2 vs >=3**

**Quantiles**

| **Level** | **Minimum** | **10%** | **25%** | **Median** | **75%** | **90%** | **Maximum** |
| --- | --- | --- | --- | --- | --- | --- | --- |
| 1/2 | 0,001223 | 0,001555 | 0,00763 | 0,012313 | 0,020027 | 0,033234 | 0,038529 |
| 3/4&5 | 0,000602 | 0,00178 | 0,004939 | 0,009121 | 0,016832 | 0,029541 | 0,051497 |

**Oneway Anova**

**Summary of Fit**

| Rsquare | 0,010073 |
| --- | --- |
| Adj Rsquare | -0,00564 |
| Root Mean Square Error | 0,011194 |
| Mean of Response | 0,013431 |
| Observations (or Sum Wgts) | 65 |

**Pooled t Test**

3/4&5-1/2

Assuming equal variances

| Difference | -0,00223 | t Ratio | -0,80067 |
| --- | --- | --- | --- |
| Std Err Dif | 0,00279 | DF | 63 |
| Upper CL Dif | 0,00334 | Prob > |t| | 0,4263 |
| Lower CL Dif | -0,00780 | Prob > t | 0,7868 |
| Confidence | 0,95 | Prob < t | 0,2132 |

**Analysis of Variance**

| **Source** | **DF** | **Sum of Squares** | **Mean Square** | **F Ratio** | **Prob > F** |
| --- | --- | --- | --- | --- | --- |
| GrG <=2 vs >=3 | 1 | 0,00008032 | 0,000080 | 0,6411 | 0,4263 |
| Error | 63 | 0,00789368 | 0,000125 |  |  |
| C. Total | 64 | 0,00797400 |  |  |  |

**Means for Oneway Anova**

| **Level** | **Number** | **Mean** | **Std Error** | **Lower 95%** | **Upper 95%** |
| --- | --- | --- | --- | --- | --- |
| 1/2 | 30 | 0,014632 | 0,00204 | 0,01055 | 0,01872 |
| 3/4&5 | 35 | 0,012402 | 0,00189 | 0,00862 | 0,01618 |

Std Error uses a pooled estimate of error variance

**Nonparametric Comparisons For Each Pair Using Wilcoxon Method**

| **q*** | **Alpha** |
| --- | --- |
| 1,95996 | 0,05 |

| **Level** | **- Level** | **Score Mean Difference** | **Std Err Dif** | **Z** | **p-Value** | **Hodges-Lehmann** | **Lower CL** | **Upper CL** | **Difference Plot** |
| --- | --- | --- | --- | --- | --- | --- | --- | --- | --- |
| 3/4&5 | 1/2 | -5,97381 | 4,704355 | -1,26985 | 0,2041 | -0,003023 | -0,007434 | 0,0015923 |  |

**Oneway Analysis of original-gldm-LowGrayLevelEmphasis By GrG <=2 vs >=3**

**Quantiles**

| **Level** | **Minimum** | **10%** | **25%** | **Median** | **75%** | **90%** | **Maximum** |
| --- | --- | --- | --- | --- | --- | --- | --- |
| 1/2 | 0,002012 | 0,002376 | 0,008666 | 0,015223 | 0,03438 | 0,076319 | 0,452866 |
| 3/4&5 | 0,000978 | 0,002763 | 0,005704 | 0,012894 | 0,019908 | 0,043662 | 0,072861 |

**Oneway Anova**

**Summary of Fit**

| Rsquare | 0,032341 |
| --- | --- |
| Adj Rsquare | 0,016981 |
| Root Mean Square Error | 0,057499 |
| Mean of Response | 0,026703 |
| Observations (or Sum Wgts) | 65 |

**Pooled t Test**

3/4&5-1/2

Assuming equal variances

| Difference | -0,02076 | t Ratio | -1,45105 |
| --- | --- | --- | --- |
| Std Err Dif | 0,01431 | DF | 63 |
| Upper CL Dif | 0,00783 | Prob > |t| | 0,1517 |
| Lower CL Dif | -0,04935 | Prob > t | 0,9241 |
| Confidence | 0,95 | Prob < t | 0,0759 |

**Analysis of Variance**

| **Source** | **DF** | **Sum of Squares** | **Mean Square** | **F Ratio** | **Prob > F** |
| --- | --- | --- | --- | --- | --- |
| GrG <=2 vs >=3 | 1 | 0,00696116 | 0,006961 | 2,1056 | 0,1517 |
| Error | 63 | 0,20828385 | 0,003306 |  |  |
| C. Total | 64 | 0,21524500 |  |  |  |

**Means for Oneway Anova**

| **Level** | **Number** | **Mean** | **Std Error** | **Lower 95%** | **Upper 95%** |
| --- | --- | --- | --- | --- | --- |
| 1/2 | 30 | 0,037881 | 0,01050 | 0,0169 | 0,05886 |
| 3/4&5 | 35 | 0,017122 | 0,00972 | -0,0023 | 0,03654 |

Std Error uses a pooled estimate of error variance

**Nonparametric Comparisons For Each Pair Using Wilcoxon Method**

| **q*** | **Alpha** |
| --- | --- |
| 1,95996 | 0,05 |

| **Level** | **- Level** | **Score Mean Difference** | **Std Err Dif** | **Z** | **p-Value** | **Hodges-Lehmann** | **Lower CL** | **Upper CL** | **Difference Plot** |
| --- | --- | --- | --- | --- | --- | --- | --- | --- | --- |
| 3/4&5 | 1/2 | -5,04524 | 4,704355 | -1,07246 | 0,2835 | -0,003398 | -0,010080 | 0,0022583 |  |

**Oneway Analysis of original-glcm-JointAverage By GrG <=2 vs >=3**

**Quantiles**

| **Level** | **Minimum** | **10%** | **25%** | **Median** | **75%** | **90%** | **Maximum** |
| --- | --- | --- | --- | --- | --- | --- | --- |
| 1/2 | 2,025363 | 11,78842 | 13,39698 | 18,91 | 24,29126 | 29,95168 | 30,2646 |
| 3/4&5 | 10,8119 | 12,964 | 14,64875 | 17,23898 | 23,99238 | 31,83839 | 42,7696 |

**Oneway Anova**

**Summary of Fit**

| Rsquare | 0,003523 |
| --- | --- |
| Adj Rsquare | -0,01229 |
| Root Mean Square Error | 7,198512 |
| Mean of Response | 19,55982 |
| Observations (or Sum Wgts) | 65 |

**Pooled t Test**

3/4&5-1/2

Assuming equal variances

| Difference | 0,8453 | t Ratio | 0,471969 |
| --- | --- | --- | --- |
| Std Err Dif | 1,7910 | DF | 63 |
| Upper CL Dif | 4,4244 | Prob > |t| | 0,6386 |
| Lower CL Dif | -2,7338 | Prob > t | 0,3193 |
| Confidence | 0,95 | Prob < t | 0,6807 |

**Analysis of Variance**

| **Source** | **DF** | **Sum of Squares** | **Mean Square** | **F Ratio** | **Prob > F** |
| --- | --- | --- | --- | --- | --- |
| GrG <=2 vs >=3 | 1 | 11,5428 | 11,5428 | 0,2228 | 0,6386 |
| Error | 63 | 3264,5702 | 51,8186 |  |  |
| C. Total | 64 | 3276,1131 |  |  |  |

**Means for Oneway Anova**

| **Level** | **Number** | **Mean** | **Std Error** | **Lower 95%** | **Upper 95%** |
| --- | --- | --- | --- | --- | --- |
| 1/2 | 30 | 19,1047 | 1,3143 | 16,478 | 21,731 |
| 3/4&5 | 35 | 19,9500 | 1,2168 | 17,518 | 22,381 |

Std Error uses a pooled estimate of error variance

**Nonparametric Comparisons For Each Pair Using Wilcoxon Method**

| **q*** | **Alpha** |
| --- | --- |
| 1,95996 | 0,05 |

| **Level** | **- Level** | **Score Mean Difference** | **Std Err Dif** | **Z** | **p-Value** | **Hodges-Lehmann** | **Lower CL** | **Upper CL** | **Difference Plot** |
| --- | --- | --- | --- | --- | --- | --- | --- | --- | --- |
| 3/4&5 | 1/2 | 1,207143 | 4,704355 | 0,2566012 | 0,7975 | 0,5344692 | -3,43467 | 3,400820 |  |

**Oneway Analysis of original-glcm-SumAverage By GrG <=2 vs >=3**

**Quantiles**

| **Level** | **Minimum** | **10%** | **25%** | **Median** | **75%** | **90%** | **Maximum** |
| --- | --- | --- | --- | --- | --- | --- | --- |
| 1/2 | 4,050725 | 23,57683 | 26,79396 | 37,82001 | 48,58252 | 59,90336 | 60,52921 |
| 3/4&5 | 21,62381 | 25,92801 | 29,29749 | 34,47795 | 47,98476 | 63,67679 | 85,53921 |

**Oneway Anova**

**Summary of Fit**

| Rsquare | 0,003523 |
| --- | --- |
| Adj Rsquare | -0,01229 |
| Root Mean Square Error | 14,39702 |
| Mean of Response | 39,11965 |
| Observations (or Sum Wgts) | 65 |

**Pooled t Test**

3/4&5-1/2

Assuming equal variances

| Difference | 1,6906 | t Ratio | 0,471969 |
| --- | --- | --- | --- |
| Std Err Dif | 3,5821 | DF | 63 |
| Upper CL Dif | 8,8488 | Prob > |t| | 0,6386 |
| Lower CL Dif | -5,4676 | Prob > t | 0,3193 |
| Confidence | 0,95 | Prob < t | 0,6807 |

**Analysis of Variance**

| **Source** | **DF** | **Sum of Squares** | **Mean Square** | **F Ratio** | **Prob > F** |
| --- | --- | --- | --- | --- | --- |
| GrG <=2 vs >=3 | 1 | 46,171 | 46,171 | 0,2228 | 0,6386 |
| Error | 63 | 13058,281 | 207,274 |  |  |
| C. Total | 64 | 13104,452 |  |  |  |

**Means for Oneway Anova**

| **Level** | **Number** | **Mean** | **Std Error** | **Lower 95%** | **Upper 95%** |
| --- | --- | --- | --- | --- | --- |
| 1/2 | 30 | 38,2093 | 2,6285 | 32,957 | 43,462 |
| 3/4&5 | 35 | 39,8999 | 2,4335 | 35,037 | 44,763 |

Std Error uses a pooled estimate of error variance

**Nonparametric Comparisons For Each Pair Using Wilcoxon Method**

| **q*** | **Alpha** |
| --- | --- |
| 1,95996 | 0,05 |

| **Level** | **- Level** | **Score Mean Difference** | **Std Err Dif** | **Z** | **p-Value** | **Hodges-Lehmann** | **Lower CL** | **Upper CL** | **Difference Plot** |
| --- | --- | --- | --- | --- | --- | --- | --- | --- | --- |
| 3/4&5 | 1/2 | 1,207143 | 4,704355 | 0,2566012 | 0,7975 | 1,068938 | -6,86935 | 6,801641 |  |

**Oneway Analysis of original-glcm-JointEntropy By GrG <=2 vs >=3**

**Quantiles**

| **Level** | **Minimum** | **10%** | **25%** | **Median** | **75%** | **90%** | **Maximum** |
| --- | --- | --- | --- | --- | --- | --- | --- |
| 1/2 | 2,886963 | 4,240379 | 5,729618 | 6,635548 | 7,747325 | 8,940191 | 9,545729 |
| 3/4&5 | 3,783291 | 5,58707 | 6,317658 | 7,788475 | 8,602772 | 9,468878 | 10,45828 |

**Oneway Anova**

**Summary of Fit**

| Rsquare | 0,076476 |
| --- | --- |
| Adj Rsquare | 0,061816 |
| Root Mean Square Error | 1,530238 |
| Mean of Response | 7,134934 |
| Observations (or Sum Wgts) | 65 |

**Pooled t Test**

3/4&5-1/2

Assuming equal variances

| Difference | 0,86962 | t Ratio | 2,28406 |
| --- | --- | --- | --- |
| Std Err Dif | 0,38073 | DF | 63 |
| Upper CL Dif | 1,63045 | Prob > |t| | 0,0257* |
| Lower CL Dif | 0,10878 | Prob > t | 0,0129* |
| Confidence | 0,95 | Prob < t | 0,9871 |

**Analysis of Variance**

| **Source** | **DF** | **Sum of Squares** | **Mean Square** | **F Ratio** | **Prob > F** |
| --- | --- | --- | --- | --- | --- |
| GrG <=2 vs >=3 | 1 | 12,21612 | 12,2161 | 5,2169 | 0,0257* |
| Error | 63 | 147,52264 | 2,3416 |  |  |
| C. Total | 64 | 159,73876 |  |  |  |

**Means for Oneway Anova**

| **Level** | **Number** | **Mean** | **Std Error** | **Lower 95%** | **Upper 95%** |
| --- | --- | --- | --- | --- | --- |
| 1/2 | 30 | 6,66668 | 0,27938 | 6,1084 | 7,2250 |
| 3/4&5 | 35 | 7,53630 | 0,25866 | 7,0194 | 8,0532 |

Std Error uses a pooled estimate of error variance

**Nonparametric Comparisons For Each Pair Using Wilcoxon Method**

| **q*** | **Alpha** |
| --- | --- |
| 1,95996 | 0,05 |

| **Level** | **- Level** | **Score Mean Difference** | **Std Err Dif** | **Z** | **p-Value** | **Hodges-Lehmann** | **Lower CL** | **Upper CL** | **Difference Plot** |
| --- | --- | --- | --- | --- | --- | --- | --- | --- | --- |
| 3/4&5 | 1/2 | 10,67857 | 4,704355 | 2,269933 | 0,0232* | 0,9004705 | 0,0903975 | 1,642937 |  |

**Oneway Analysis of original-glcm-ClusterShade By GrG <=2 vs >=3**

**Quantiles**

| **Level** | **Minimum** | **10%** | **25%** | **Median** | **75%** | **90%** | **Maximum** |
| --- | --- | --- | --- | --- | --- | --- | --- |
| 1/2 | -2455,52 | -80,9138 | -8,21737 | 271,444 | 695,5622 | 1765,949 | 4973,719 |
| 3/4&5 | -488,485 | -292,776 | 7,822719 | 317,6254 | 1462,832 | 4722,829 | 8848,825 |

**Oneway Anova**

**Summary of Fit**

| Rsquare | 0,030975 |
| --- | --- |
| Adj Rsquare | 0,015594 |
| Root Mean Square Error | 1803,185 |
| Mean of Response | 876,0161 |
| Observations (or Sum Wgts) | 65 |

**Pooled t Test**

3/4&5-1/2

Assuming equal variances

| Difference | 636,7 | t Ratio | 1,41909 |
| --- | --- | --- | --- |
| Std Err Dif | 448,6 | DF | 63 |
| Upper CL Dif | 1533,2 | Prob > |t| | 0,1608 |
| Lower CL Dif | -259,9 | Prob > t | 0,0804 |
| Confidence | 0,95 | Prob < t | 0,9196 |

**Analysis of Variance**

| **Source** | **DF** | **Sum of Squares** | **Mean Square** | **F Ratio** | **Prob > F** |
| --- | --- | --- | --- | --- | --- |
| GrG <=2 vs >=3 | 1 | 6547875 | 6547875 | 2,0138 | 0,1608 |
| Error | 63 | 204842977 | 3251476 |  |  |
| C. Total | 64 | 211390853 |  |  |  |

**Means for Oneway Anova**

| **Level** | **Number** | **Mean** | **Std Error** | **Lower 95%** | **Upper 95%** |
| --- | --- | --- | --- | --- | --- |
| 1/2 | 30 | 533,20 | 329,22 | -124,7 | 1191,1 |
| 3/4&5 | 35 | 1169,86 | 304,79 | 560,8 | 1778,9 |

Std Error uses a pooled estimate of error variance

**Nonparametric Comparisons For Each Pair Using Wilcoxon Method**

| **q*** | **Alpha** |
| --- | --- |
| 1,95996 | 0,05 |

| **Level** | **- Level** | **Score Mean Difference** | **Std Err Dif** | **Z** | **p-Value** | **Hodges-Lehmann** | **Lower CL** | **Upper CL** | **Difference Plot** |
| --- | --- | --- | --- | --- | --- | --- | --- | --- | --- |
| 3/4&5 | 1/2 | 2,383333 | 4,704355 | 0,5066228 | 0,6124 | 75,64642 | -215,213 | 404,1328 |  |

**Oneway Analysis of original-glcm-MaximumProbability By GrG <=2 vs >=3**

**Quantiles**

| **Level** | **Minimum** | **10%** | **25%** | **Median** | **75%** | **90%** | **Maximum** |
| --- | --- | --- | --- | --- | --- | --- | --- |
| 1/2 | 0,005748 | 0,009535 | 0,015519 | 0,025295 | 0,036955 | 0,094238 | 0,20984 |
| 3/4&5 | 0,003615 | 0,006187 | 0,010117 | 0,013621 | 0,029026 | 0,04313 | 0,101805 |

**Oneway Anova**

**Summary of Fit**

| Rsquare | 0,053246 |
| --- | --- |
| Adj Rsquare | 0,038219 |
| Root Mean Square Error | 0,032952 |
| Mean of Response | 0,029009 |
| Observations (or Sum Wgts) | 65 |

**Pooled t Test**

3/4&5-1/2

Assuming equal variances

| Difference | -0,01543 | t Ratio | -1,88234 |
| --- | --- | --- | --- |
| Std Err Dif | 0,00820 | DF | 63 |
| Upper CL Dif | 0,00095 | Prob > |t| | 0,0644 |
| Lower CL Dif | -0,03182 | Prob > t | 0,9678 |
| Confidence | 0,95 | Prob < t | 0,0322* |

**Analysis of Variance**

| **Source** | **DF** | **Sum of Squares** | **Mean Square** | **F Ratio** | **Prob > F** |
| --- | --- | --- | --- | --- | --- |
| GrG <=2 vs >=3 | 1 | 0,00384740 | 0,003847 | 3,5432 | 0,0644 |
| Error | 63 | 0,06840908 | 0,001086 |  |  |
| C. Total | 64 | 0,07225648 |  |  |  |

**Means for Oneway Anova**

| **Level** | **Number** | **Mean** | **Std Error** | **Lower 95%** | **Upper 95%** |
| --- | --- | --- | --- | --- | --- |
| 1/2 | 30 | 0,037319 | 0,00602 | 0,02530 | 0,04934 |
| 3/4&5 | 35 | 0,021887 | 0,00557 | 0,01076 | 0,03302 |

Std Error uses a pooled estimate of error variance

**Nonparametric Comparisons For Each Pair Using Wilcoxon Method**

| **q*** | **Alpha** |
| --- | --- |
| 1,95996 | 0,05 |

| **Level** | **- Level** | **Score Mean Difference** | **Std Err Dif** | **Z** | **p-Value** | **Hodges-Lehmann** | **Lower CL** | **Upper CL** | **Difference Plot** |
| --- | --- | --- | --- | --- | --- | --- | --- | --- | --- |
| 3/4&5 | 1/2 | -10,5548 | 4,704355 | -2,24362 | 0,0249* | -0,007734 | -0,014810 | -0,000718 |  |

**Oneway Analysis of original-glcm-Idmn By GrG <=2 vs >=3**

**Quantiles**

| **Level** | **Minimum** | **10%** | **25%** | **Median** | **75%** | **90%** | **Maximum** |
| --- | --- | --- | --- | --- | --- | --- | --- |
| 1/2 | 0,908704 | 0,915513 | 0,932685 | 0,95207 | 0,968232 | 0,982078 | 0,984015 |
| 3/4&5 | 0,901195 | 0,919087 | 0,94025 | 0,960209 | 0,969041 | 0,982344 | 0,988362 |

**Oneway Anova**

**Summary of Fit**

| Rsquare | 0,008138 |
| --- | --- |
| Adj Rsquare | -0,00761 |
| Root Mean Square Error | 0,022363 |
| Mean of Response | 0,95383 |
| Observations (or Sum Wgts) | 65 |

**Pooled t Test**

3/4&5-1/2

Assuming equal variances

| Difference | 0,00400 | t Ratio | 0,718966 |
| --- | --- | --- | --- |
| Std Err Dif | 0,00556 | DF | 63 |
| Upper CL Dif | 0,01512 | Prob > |t| | 0,4748 |
| Lower CL Dif | -0,00712 | Prob > t | 0,2374 |
| Confidence | 0,95 | Prob < t | 0,7626 |

**Analysis of Variance**

| **Source** | **DF** | **Sum of Squares** | **Mean Square** | **F Ratio** | **Prob > F** |
| --- | --- | --- | --- | --- | --- |
| GrG <=2 vs >=3 | 1 | 0,00025850 | 0,000259 | 0,5169 | 0,4748 |
| Error | 63 | 0,03150539 | 0,000500 |  |  |
| C. Total | 64 | 0,03176389 |  |  |  |

**Means for Oneway Anova**

| **Level** | **Number** | **Mean** | **Std Error** | **Lower 95%** | **Upper 95%** |
| --- | --- | --- | --- | --- | --- |
| 1/2 | 30 | 0,951676 | 0,00408 | 0,94352 | 0,95984 |
| 3/4&5 | 35 | 0,955676 | 0,00378 | 0,94812 | 0,96323 |

Std Error uses a pooled estimate of error variance

**Nonparametric Comparisons For Each Pair Using Wilcoxon Method**

| **q*** | **Alpha** |
| --- | --- |
| 1,95996 | 0,05 |

| **Level** | **- Level** | **Score Mean Difference** | **Std Err Dif** | **Z** | **p-Value** | **Hodges-Lehmann** | **Lower CL** | **Upper CL** | **Difference Plot** |
| --- | --- | --- | --- | --- | --- | --- | --- | --- | --- |
| 3/4&5 | 1/2 | 3,373810 | 4,704355 | 0,7171673 | 0,4733 | 0,0045827 | -0,007067 | 0,0158454 |  |

**Oneway Analysis of original-glcm-JointEnergy By GrG <=2 vs >=3**

**Quantiles**

| **Level** | **Minimum** | **10%** | **25%** | **Median** | **75%** | **90%** | **Maximum** |
| --- | --- | --- | --- | --- | --- | --- | --- |
| 1/2 | 0,001829 | 0,002665 | 0,005351 | 0,01089 | 0,020034 | 0,062064 | 0,150516 |
| 3/4&5 | 0,001129 | 0,001876 | 0,002961 | 0,005482 | 0,013236 | 0,023042 | 0,07976 |

**Oneway Anova**

**Summary of Fit**

| Rsquare | 0,046417 |
| --- | --- |
| Adj Rsquare | 0,031281 |
| Root Mean Square Error | 0,025296 |
| Mean of Response | 0,015918 |
| Observations (or Sum Wgts) | 65 |

**Pooled t Test**

3/4&5-1/2

Assuming equal variances

| Difference | -0,01102 | t Ratio | -1,75118 |
| --- | --- | --- | --- |
| Std Err Dif | 0,00629 | DF | 63 |
| Upper CL Dif | 0,00156 | Prob > |t| | 0,0848 |
| Lower CL Dif | -0,02360 | Prob > t | 0,9576 |
| Confidence | 0,95 | Prob < t | 0,0424* |

**Analysis of Variance**

| **Source** | **DF** | **Sum of Squares** | **Mean Square** | **F Ratio** | **Prob > F** |
| --- | --- | --- | --- | --- | --- |
| GrG <=2 vs >=3 | 1 | 0,00196236 | 0,001962 | 3,0666 | 0,0848 |
| Error | 63 | 0,04031421 | 0,000640 |  |  |
| C. Total | 64 | 0,04227657 |  |  |  |

**Means for Oneway Anova**

| **Level** | **Number** | **Mean** | **Std Error** | **Lower 95%** | **Upper 95%** |
| --- | --- | --- | --- | --- | --- |
| 1/2 | 30 | 0,021853 | 0,00462 | 0,01262 | 0,03108 |
| 3/4&5 | 35 | 0,010831 | 0,00428 | 0,00229 | 0,01938 |

Std Error uses a pooled estimate of error variance

**Nonparametric Comparisons For Each Pair Using Wilcoxon Method**

| **q*** | **Alpha** |
| --- | --- |
| 1,95996 | 0,05 |

| **Level** | **- Level** | **Score Mean Difference** | **Std Err Dif** | **Z** | **p-Value** | **Hodges-Lehmann** | **Lower CL** | **Upper CL** | **Difference Plot** |
| --- | --- | --- | --- | --- | --- | --- | --- | --- | --- |
| 3/4&5 | 1/2 | -10,7405 | 4,704355 | -2,28309 | 0,0224* | -0,003892 | -0,008245 | -0,000498 |  |

**Oneway Analysis of original-glcm-Contrast By GrG <=2 vs >=3**

**Quantiles**

| **Level** | **Minimum** | **10%** | **25%** | **Median** | **75%** | **90%** | **Maximum** |
| --- | --- | --- | --- | --- | --- | --- | --- |
| 1/2 | 0,871287 | 41,34459 | 48,88575 | 69,38204 | 105,1371 | 142,1112 | 207,3697 |
| 3/4&5 | 37,57956 | 41,72039 | 52,04558 | 65,78068 | 98,48386 | 143,0014 | 232,2285 |

**Oneway Anova**

**Summary of Fit**

| Rsquare | 0,000319 |
| --- | --- |
| Adj Rsquare | -0,01555 |
| Root Mean Square Error | 42,5637 |
| Mean of Response | 80,70491 |
| Observations (or Sum Wgts) | 65 |

**Pooled t Test**

3/4&5-1/2

Assuming equal variances

| Difference | 1,502 | t Ratio | 0,14184 |
| --- | --- | --- | --- |
| Std Err Dif | 10,590 | DF | 63 |
| Upper CL Dif | 22,665 | Prob > |t| | 0,8877 |
| Lower CL Dif | -19,661 | Prob > t | 0,4438 |
| Confidence | 0,95 | Prob < t | 0,5562 |

**Analysis of Variance**

| **Source** | **DF** | **Sum of Squares** | **Mean Square** | **F Ratio** | **Prob > F** |
| --- | --- | --- | --- | --- | --- |
| GrG <=2 vs >=3 | 1 | 36,45 | 36,45 | 0,0201 | 0,8877 |
| Error | 63 | 114135,13 | 1811,67 |  |  |
| C. Total | 64 | 114171,58 |  |  |  |

**Means for Oneway Anova**

| **Level** | **Number** | **Mean** | **Std Error** | **Lower 95%** | **Upper 95%** |
| --- | --- | --- | --- | --- | --- |
| 1/2 | 30 | 79,8961 | 7,7710 | 64,367 | 95,425 |
| 3/4&5 | 35 | 81,3982 | 7,1946 | 67,021 | 95,775 |

Std Error uses a pooled estimate of error variance

**Nonparametric Comparisons For Each Pair Using Wilcoxon Method**

| **q*** | **Alpha** |
| --- | --- |
| 1,95996 | 0,05 |

| **Level** | **- Level** | **Score Mean Difference** | **Std Err Dif** | **Z** | **p-Value** | **Hodges-Lehmann** | **Lower CL** | **Upper CL** | **Difference Plot** |
| --- | --- | --- | --- | --- | --- | --- | --- | --- | --- |
| 3/4&5 | 1/2 | 0,3404762 | 4,704355 | 0,0723747 | 0,9423 | 0,5424153 | -15,5865 | 17,43754 |  |

**Oneway Analysis of original-glcm-DifferenceEntropy By GrG <=2 vs >=3**

**Quantiles**

| **Level** | **Minimum** | **10%** | **25%** | **Median** | **75%** | **90%** | **Maximum** |
| --- | --- | --- | --- | --- | --- | --- | --- |
| 1/2 | 1,179581 | 2,960222 | 3,593003 | 3,766282 | 4,007777 | 4,191338 | 4,314705 |
| 3/4&5 | 2,501643 | 3,457232 | 3,654926 | 3,923036 | 4,21046 | 4,451305 | 4,719208 |

**Oneway Anova**

**Summary of Fit**

| Rsquare | 0,046261 |
| --- | --- |
| Adj Rsquare | 0,031122 |
| Root Mean Square Error | 0,550252 |
| Mean of Response | 3,791333 |
| Observations (or Sum Wgts) | 65 |

**Pooled t Test**

3/4&5-1/2

Assuming equal variances

| Difference | 0,23932 | t Ratio | 1,748087 |
| --- | --- | --- | --- |
| Std Err Dif | 0,13691 | DF | 63 |
| Upper CL Dif | 0,51291 | Prob > |t| | 0,0853 |
| Lower CL Dif | -0,03426 | Prob > t | 0,0427* |
| Confidence | 0,95 | Prob < t | 0,9573 |

**Analysis of Variance**

| **Source** | **DF** | **Sum of Squares** | **Mean Square** | **F Ratio** | **Prob > F** |
| --- | --- | --- | --- | --- | --- |
| GrG <=2 vs >=3 | 1 | 0,925230 | 0,925230 | 3,0558 | 0,0853 |
| Error | 63 | 19,074976 | 0,302777 |  |  |
| C. Total | 64 | 20,000206 |  |  |  |

**Means for Oneway Anova**

| **Level** | **Number** | **Mean** | **Std Error** | **Lower 95%** | **Upper 95%** |
| --- | --- | --- | --- | --- | --- |
| 1/2 | 30 | 3,66247 | 0,10046 | 3,4617 | 3,8632 |
| 3/4&5 | 35 | 3,90179 | 0,09301 | 3,7159 | 4,0877 |

Std Error uses a pooled estimate of error variance

**Nonparametric Comparisons For Each Pair Using Wilcoxon Method**

| **q*** | **Alpha** |
| --- | --- |
| 1,95996 | 0,05 |

| **Level** | **- Level** | **Score Mean Difference** | **Std Err Dif** | **Z** | **p-Value** | **Hodges-Lehmann** | **Lower CL** | **Upper CL** | **Difference Plot** |
| --- | --- | --- | --- | --- | --- | --- | --- | --- | --- |
| 3/4&5 | 1/2 | 7,273810 | 4,704355 | 1,546186 | 0,1221 | 0,1532835 | -0,048133 | 0,3462271 |  |

**Oneway Analysis of original-glcm-InverseVariance By GrG <=2 vs >=3**

**Quantiles**

| **Level** | **Minimum** | **10%** | **25%** | **Median** | **75%** | **90%** | **Maximum** |
| --- | --- | --- | --- | --- | --- | --- | --- |
| 1/2 | 0,101151 | 0,118023 | 0,13285 | 0,157577 | 0,186806 | 0,204459 | 0,528035 |
| 3/4&5 | 0,118181 | 0,125044 | 0,137466 | 0,157157 | 0,178137 | 0,19132 | 0,195496 |

**Oneway Anova**

**Summary of Fit**

| Rsquare | 0,017739 |
| --- | --- |
| Adj Rsquare | 0,002148 |
| Root Mean Square Error | 0,053548 |
| Mean of Response | 0,164754 |
| Observations (or Sum Wgts) | 65 |

**Pooled t Test**

3/4&5-1/2

Assuming equal variances

| Difference | -0,01421 | t Ratio | -1,06666 |
| --- | --- | --- | --- |
| Std Err Dif | 0,01332 | DF | 63 |
| Upper CL Dif | 0,01241 | Prob > |t| | 0,2902 |
| Lower CL Dif | -0,04084 | Prob > t | 0,8549 |
| Confidence | 0,95 | Prob < t | 0,1451 |

**Analysis of Variance**

| **Source** | **DF** | **Sum of Squares** | **Mean Square** | **F Ratio** | **Prob > F** |
| --- | --- | --- | --- | --- | --- |
| GrG <=2 vs >=3 | 1 | 0,00326243 | 0,003262 | 1,1378 | 0,2902 |
| Error | 63 | 0,18064760 | 0,002867 |  |  |
| C. Total | 64 | 0,18391003 |  |  |  |

**Means for Oneway Anova**

| **Level** | **Number** | **Mean** | **Std Error** | **Lower 95%** | **Upper 95%** |
| --- | --- | --- | --- | --- | --- |
| 1/2 | 30 | 0,172406 | 0,00978 | 0,15287 | 0,19194 |
| 3/4&5 | 35 | 0,158195 | 0,00905 | 0,14011 | 0,17628 |

Std Error uses a pooled estimate of error variance

**Nonparametric Comparisons For Each Pair Using Wilcoxon Method**

| **q*** | **Alpha** |
| --- | --- |
| 1,95996 | 0,05 |

| **Level** | **- Level** | **Score Mean Difference** | **Std Err Dif** | **Z** | **p-Value** | **Hodges-Lehmann** | **Lower CL** | **Upper CL** | **Difference Plot** |
| --- | --- | --- | --- | --- | --- | --- | --- | --- | --- |
| 3/4&5 | 1/2 | -1,45476 | 4,704355 | -0,309237 | 0,7571 | -0,001961 | -0,016769 | 0,0127548 |  |

**Oneway Analysis of original-glcm-DifferenceVariance By GrG <=2 vs >=3**

**Quantiles**

| **Level** | **Minimum** | **10%** | **25%** | **Median** | **75%** | **90%** | **Maximum** |
| --- | --- | --- | --- | --- | --- | --- | --- |
| 1/2 | 0,335983 | 14,75096 | 19,14186 | 26,76359 | 38,98303 | 52,45765 | 90,11708 |
| 3/4&5 | 13,88514 | 15,43463 | 19,01097 | 26,6353 | 39,73583 | 58,07574 | 104,0875 |

**Oneway Anova**

**Summary of Fit**

| Rsquare | 0,003081 |
| --- | --- |
| Adj Rsquare | -0,01274 |
| Root Mean Square Error | 17,88134 |
| Mean of Response | 31,37578 |
| Observations (or Sum Wgts) | 65 |

**Pooled t Test**

3/4&5-1/2

Assuming equal variances

| Difference | 1,963 | t Ratio | 0,441236 |
| --- | --- | --- | --- |
| Std Err Dif | 4,449 | DF | 63 |
| Upper CL Dif | 10,854 | Prob > |t| | 0,6606 |
| Lower CL Dif | -6,928 | Prob > t | 0,3303 |
| Confidence | 0,95 | Prob < t | 0,6697 |

**Analysis of Variance**

| **Source** | **DF** | **Sum of Squares** | **Mean Square** | **F Ratio** | **Prob > F** |
| --- | --- | --- | --- | --- | --- |
| GrG <=2 vs >=3 | 1 | 62,250 | 62,250 | 0,1947 | 0,6606 |
| Error | 63 | 20143,772 | 319,742 |  |  |
| C. Total | 64 | 20206,023 |  |  |  |

**Means for Oneway Anova**

| **Level** | **Number** | **Mean** | **Std Error** | **Lower 95%** | **Upper 95%** |
| --- | --- | --- | --- | --- | --- |
| 1/2 | 30 | 30,3188 | 3,2647 | 23,795 | 36,843 |
| 3/4&5 | 35 | 32,2818 | 3,0225 | 26,242 | 38,322 |

Std Error uses a pooled estimate of error variance

**Nonparametric Comparisons For Each Pair Using Wilcoxon Method**

| **q*** | **Alpha** |
| --- | --- |
| 1,95996 | 0,05 |

| **Level** | **- Level** | **Score Mean Difference** | **Std Err Dif** | **Z** | **p-Value** | **Hodges-Lehmann** | **Lower CL** | **Upper CL** | **Difference Plot** |
| --- | --- | --- | --- | --- | --- | --- | --- | --- | --- |
| 3/4&5 | 1/2 | 1,454762 | 4,704355 | 0,3092373 | 0,7571 | 0,9338612 | -5,42264 | 7,605239 |  |

**Oneway Analysis of original-glcm-Idn By GrG <=2 vs >=3**

**Quantiles**

| **Level** | **Minimum** | **10%** | **25%** | **Median** | **75%** | **90%** | **Maximum** |
| --- | --- | --- | --- | --- | --- | --- | --- |
| 1/2 | 0,810268 | 0,815353 | 0,836565 | 0,861932 | 0,88297 | 0,910434 | 0,916302 |
| 3/4&5 | 0,791767 | 0,821634 | 0,845923 | 0,868621 | 0,888124 | 0,91024 | 0,930662 |

**Oneway Anova**

**Summary of Fit**

| Rsquare | 0,006803 |
| --- | --- |
| Adj Rsquare | -0,00896 |
| Root Mean Square Error | 0,032216 |
| Mean of Response | 0,864535 |
| Observations (or Sum Wgts) | 65 |

**Pooled t Test**

3/4&5-1/2

Assuming equal variances

| Difference | 0,00527 | t Ratio | 0,656918 |
| --- | --- | --- | --- |
| Std Err Dif | 0,00802 | DF | 63 |
| Upper CL Dif | 0,02128 | Prob > |t| | 0,5136 |
| Lower CL Dif | -0,01075 | Prob > t | 0,2568 |
| Confidence | 0,95 | Prob < t | 0,7432 |

**Analysis of Variance**

| **Source** | **DF** | **Sum of Squares** | **Mean Square** | **F Ratio** | **Prob > F** |
| --- | --- | --- | --- | --- | --- |
| GrG <=2 vs >=3 | 1 | 0,00044789 | 0,000448 | 0,4315 | 0,5136 |
| Error | 63 | 0,06538647 | 0,001038 |  |  |
| C. Total | 64 | 0,06583436 |  |  |  |

**Means for Oneway Anova**

| **Level** | **Number** | **Mean** | **Std Error** | **Lower 95%** | **Upper 95%** |
| --- | --- | --- | --- | --- | --- |
| 1/2 | 30 | 0,861699 | 0,00588 | 0,84995 | 0,87345 |
| 3/4&5 | 35 | 0,866965 | 0,00545 | 0,85608 | 0,87785 |

Std Error uses a pooled estimate of error variance

**Nonparametric Comparisons For Each Pair Using Wilcoxon Method**

| **q*** | **Alpha** |
| --- | --- |
| 1,95996 | 0,05 |

| **Level** | **- Level** | **Score Mean Difference** | **Std Err Dif** | **Z** | **p-Value** | **Hodges-Lehmann** | **Lower CL** | **Upper CL** | **Difference Plot** |
| --- | --- | --- | --- | --- | --- | --- | --- | --- | --- |
| 3/4&5 | 1/2 | 3,435714 | 4,704355 | 0,7303264 | 0,4652 | 0,0062100 | -0,010367 | 0,0240158 |  |

**Oneway Analysis of original-glcm-Idm By GrG <=2 vs >=3**

**Quantiles**

| **Level** | **Minimum** | **10%** | **25%** | **Median** | **75%** | **90%** | **Maximum** |
| --- | --- | --- | --- | --- | --- | --- | --- |
| 1/2 | 0,111005 | 0,122722 | 0,145314 | 0,161493 | 0,186679 | 0,20023 | 0,674197 |
| 3/4&5 | 0,114081 | 0,125862 | 0,145225 | 0,156047 | 0,177357 | 0,189915 | 0,214419 |

**Oneway Anova**

**Summary of Fit**

| Rsquare | 0,022525 |
| --- | --- |
| Adj Rsquare | 0,007009 |
| Root Mean Square Error | 0,068837 |
| Mean of Response | 0,16843 |
| Observations (or Sum Wgts) | 65 |

**Pooled t Test**

3/4&5-1/2

Assuming equal variances

| Difference | -0,02064 | t Ratio | -1,20489 |
| --- | --- | --- | --- |
| Std Err Dif | 0,01713 | DF | 63 |
| Upper CL Dif | 0,01359 | Prob > |t| | 0,2328 |
| Lower CL Dif | -0,05486 | Prob > t | 0,8836 |
| Confidence | 0,95 | Prob < t | 0,1164 |

**Analysis of Variance**

| **Source** | **DF** | **Sum of Squares** | **Mean Square** | **F Ratio** | **Prob > F** |
| --- | --- | --- | --- | --- | --- |
| GrG <=2 vs >=3 | 1 | 0,00687932 | 0,006879 | 1,4518 | 0,2328 |
| Error | 63 | 0,29853149 | 0,004739 |  |  |
| C. Total | 64 | 0,30541081 |  |  |  |

**Means for Oneway Anova**

| **Level** | **Number** | **Mean** | **Std Error** | **Lower 95%** | **Upper 95%** |
| --- | --- | --- | --- | --- | --- |
| 1/2 | 30 | 0,179542 | 0,01257 | 0,15443 | 0,20466 |
| 3/4&5 | 35 | 0,158905 | 0,01164 | 0,13565 | 0,18216 |

Std Error uses a pooled estimate of error variance

**Nonparametric Comparisons For Each Pair Using Wilcoxon Method**

| **q*** | **Alpha** |
| --- | --- |
| 1,95996 | 0,05 |

| **Level** | **- Level** | **Score Mean Difference** | **Std Err Dif** | **Z** | **p-Value** | **Hodges-Lehmann** | **Lower CL** | **Upper CL** | **Difference Plot** |
| --- | --- | --- | --- | --- | --- | --- | --- | --- | --- |
| 3/4&5 | 1/2 | -3,43571 | 4,704355 | -0,730326 | 0,4652 | -0,004697 | -0,018302 | 0,0096132 |  |

**Oneway Analysis of original-glcm-Correlation By GrG <=2 vs >=3**

**Quantiles**

| **Level** | **Minimum** | **10%** | **25%** | **Median** | **75%** | **90%** | **Maximum** |
| --- | --- | --- | --- | --- | --- | --- | --- |
| 1/2 | -0,16306 | -0,01087 | 0,132935 | 0,219202 | 0,41344 | 0,498907 | 0,565814 |
| 3/4&5 | -0,07444 | 0,03706 | 0,16558 | 0,28357 | 0,362208 | 0,494239 | 0,578163 |

**Oneway Anova**

**Summary of Fit**

| Rsquare | 0,002802 |
| --- | --- |
| Adj Rsquare | -0,01303 |
| Root Mean Square Error | 0,168163 |
| Mean of Response | 0,259202 |
| Observations (or Sum Wgts) | 65 |

**Pooled t Test**

3/4&5-1/2

Assuming equal variances

| Difference | 0,01760 | t Ratio | 0,420766 |
| --- | --- | --- | --- |
| Std Err Dif | 0,04184 | DF | 63 |
| Upper CL Dif | 0,10122 | Prob > |t| | 0,6754 |
| Lower CL Dif | -0,06601 | Prob > t | 0,3377 |
| Confidence | 0,95 | Prob < t | 0,6623 |

**Analysis of Variance**

| **Source** | **DF** | **Sum of Squares** | **Mean Square** | **F Ratio** | **Prob > F** |
| --- | --- | --- | --- | --- | --- |
| GrG <=2 vs >=3 | 1 | 0,0050066 | 0,005007 | 0,1770 | 0,6754 |
| Error | 63 | 1,7815740 | 0,028279 |  |  |
| C. Total | 64 | 1,7865806 |  |  |  |

**Means for Oneway Anova**

| **Level** | **Number** | **Mean** | **Std Error** | **Lower 95%** | **Upper 95%** |
| --- | --- | --- | --- | --- | --- |
| 1/2 | 30 | 0,249722 | 0,03070 | 0,18837 | 0,31108 |
| 3/4&5 | 35 | 0,267327 | 0,02842 | 0,21052 | 0,32413 |

Std Error uses a pooled estimate of error variance

**Nonparametric Comparisons For Each Pair Using Wilcoxon Method**

| **q*** | **Alpha** |
| --- | --- |
| 1,95996 | 0,05 |

| **Level** | **- Level** | **Score Mean Difference** | **Std Err Dif** | **Z** | **p-Value** | **Hodges-Lehmann** | **Lower CL** | **Upper CL** | **Difference Plot** |
| --- | --- | --- | --- | --- | --- | --- | --- | --- | --- |
| 3/4&5 | 1/2 | 2,197619 | 4,704355 | 0,4671457 | 0,6404 | 0,0248648 | -0,074399 | 0,1114945 |  |

**Oneway Analysis of original-glcm-Autocorrelation By GrG <=2 vs >=3**

**Quantiles**

| **Level** | **Minimum** | **10%** | **25%** | **Median** | **75%** | **90%** | **Maximum** |
| --- | --- | --- | --- | --- | --- | --- | --- |
| 1/2 | 4,269287 | 143,5188 | 194,483 | 368,5184 | 653,1398 | 927,903 | 951,8811 |
| 3/4&5 | 121,1122 | 174,5514 | 217,3891 | 311,9801 | 581,0507 | 1048,681 | 1915,536 |

**Oneway Anova**

**Summary of Fit**

| Rsquare | 0,003437 |
| --- | --- |
| Adj Rsquare | -0,01238 |
| Root Mean Square Error | 334,9159 |
| Mean of Response | 451,3517 |
| Observations (or Sum Wgts) | 65 |

**Pooled t Test**

3/4&5-1/2

Assuming equal variances

| Difference | 38,84 | t Ratio | 0,466123 |
| --- | --- | --- | --- |
| Std Err Dif | 83,33 | DF | 63 |
| Upper CL Dif | 205,36 | Prob > |t| | 0,6427 |
| Lower CL Dif | -127,68 | Prob > t | 0,3214 |
| Confidence | 0,95 | Prob < t | 0,6786 |

**Analysis of Variance**

| **Source** | **DF** | **Sum of Squares** | **Mean Square** | **F Ratio** | **Prob > F** |
| --- | --- | --- | --- | --- | --- |
| GrG <=2 vs >=3 | 1 | 24370,9 | 24371 | 0,2173 | 0,6427 |
| Error | 63 | 7066624,6 | 112169 |  |  |
| C. Total | 64 | 7090995,5 |  |  |  |

**Means for Oneway Anova**

| **Level** | **Number** | **Mean** | **Std Error** | **Lower 95%** | **Upper 95%** |
| --- | --- | --- | --- | --- | --- |
| 1/2 | 30 | 430,437 | 61,147 | 308,24 | 552,63 |
| 3/4&5 | 35 | 469,279 | 56,611 | 356,15 | 582,41 |

Std Error uses a pooled estimate of error variance

**Nonparametric Comparisons For Each Pair Using Wilcoxon Method**

| **q*** | **Alpha** |
| --- | --- |
| 1,95996 | 0,05 |

| **Level** | **- Level** | **Score Mean Difference** | **Std Err Dif** | **Z** | **p-Value** | **Hodges-Lehmann** | **Lower CL** | **Upper CL** | **Difference Plot** |
| --- | --- | --- | --- | --- | --- | --- | --- | --- | --- |
| 3/4&5 | 1/2 | 1,021429 | 4,704355 | 0,2171241 | 0,8281 | 14,16472 | -125,200 | 119,8331 |  |

**Oneway Analysis of original-glcm-SumEntropy By GrG <=2 vs >=3**

**Quantiles**

| **Level** | **Minimum** | **10%** | **25%** | **Median** | **75%** | **90%** | **Maximum** |
| --- | --- | --- | --- | --- | --- | --- | --- |
| 1/2 | 2,119551 | 3,218483 | 4,294999 | 4,763883 | 5,262218 | 5,751575 | 5,816552 |
| 3/4&5 | 2,71124 | 4,052045 | 4,54069 | 5,092043 | 5,52639 | 5,869448 | 6,321469 |

**Oneway Anova**

**Summary of Fit**

| Rsquare | 0,052444 |
| --- | --- |
| Adj Rsquare | 0,037403 |
| Root Mean Square Error | 0,834295 |
| Mean of Response | 4,84316 |
| Observations (or Sum Wgts) | 65 |

**Pooled t Test**

3/4&5-1/2

Assuming equal variances

| Difference | 0,38761 | t Ratio | 1,867305 |
| --- | --- | --- | --- |
| Std Err Dif | 0,20758 | DF | 63 |
| Upper CL Dif | 0,80242 | Prob > |t| | 0,0665 |
| Lower CL Dif | -0,02720 | Prob > t | 0,0333* |
| Confidence | 0,95 | Prob < t | 0,9667 |

**Analysis of Variance**

| **Source** | **DF** | **Sum of Squares** | **Mean Square** | **F Ratio** | **Prob > F** |
| --- | --- | --- | --- | --- | --- |
| GrG <=2 vs >=3 | 1 | 2,426997 | 2,42700 | 3,4868 | 0,0665 |
| Error | 63 | 43,850997 | 0,69605 |  |  |
| C. Total | 64 | 46,277994 |  |  |  |

**Means for Oneway Anova**

| **Level** | **Number** | **Mean** | **Std Error** | **Lower 95%** | **Upper 95%** |
| --- | --- | --- | --- | --- | --- |
| 1/2 | 30 | 4,63445 | 0,15232 | 4,3301 | 4,9388 |
| 3/4&5 | 35 | 5,02206 | 0,14102 | 4,7402 | 5,3039 |

Std Error uses a pooled estimate of error variance

**Nonparametric Comparisons For Each Pair Using Wilcoxon Method**

| **q*** | **Alpha** |
| --- | --- |
| 1,95996 | 0,05 |

| **Level** | **- Level** | **Score Mean Difference** | **Std Err Dif** | **Z** | **p-Value** | **Hodges-Lehmann** | **Lower CL** | **Upper CL** | **Difference Plot** |
| --- | --- | --- | --- | --- | --- | --- | --- | --- | --- |
| 3/4&5 | 1/2 | 8,821429 | 4,704355 | 1,875162 | 0,0608 | 0,3520823 | -0,015093 | 0,7242516 |  |

**Oneway Analysis of original-glcm-SumSquares By GrG <=2 vs >=3**

**Quantiles**

| **Level** | **Minimum** | **10%** | **25%** | **Median** | **75%** | **90%** | **Maximum** |
| --- | --- | --- | --- | --- | --- | --- | --- |
| 1/2 | 0,594845 | 26,96278 | 34,81579 | 50,71497 | 70,56793 | 121,8786 | 141,4891 |
| 3/4&5 | 20,76083 | 25,97019 | 32,67509 | 49,30146 | 74,07974 | 113,0512 | 179,9478 |

**Oneway Anova**

**Summary of Fit**

| Rsquare | 0,003088 |
| --- | --- |
| Adj Rsquare | -0,01274 |
| Root Mean Square Error | 35,23537 |
| Mean of Response | 58,27866 |
| Observations (or Sum Wgts) | 65 |

**Pooled t Test**

3/4&5-1/2

Assuming equal variances

| Difference | 3,872 | t Ratio | 0,441723 |
| --- | --- | --- | --- |
| Std Err Dif | 8,767 | DF | 63 |
| Upper CL Dif | 21,392 | Prob > |t| | 0,6602 |
| Lower CL Dif | -13,647 | Prob > t | 0,3301 |
| Confidence | 0,95 | Prob < t | 0,6699 |

**Analysis of Variance**

| **Source** | **DF** | **Sum of Squares** | **Mean Square** | **F Ratio** | **Prob > F** |
| --- | --- | --- | --- | --- | --- |
| GrG <=2 vs >=3 | 1 | 242,247 | 242,25 | 0,1951 | 0,6602 |
| Error | 63 | 78216,477 | 1241,53 |  |  |
| C. Total | 64 | 78458,723 |  |  |  |

**Means for Oneway Anova**

| **Level** | **Number** | **Mean** | **Std Error** | **Lower 95%** | **Upper 95%** |
| --- | --- | --- | --- | --- | --- |
| 1/2 | 30 | 56,1935 | 6,4331 | 43,338 | 69,049 |
| 3/4&5 | 35 | 60,0660 | 5,9559 | 48,164 | 71,968 |

Std Error uses a pooled estimate of error variance

**Nonparametric Comparisons For Each Pair Using Wilcoxon Method**

| **q*** | **Alpha** |
| --- | --- |
| 1,95996 | 0,05 |

| **Level** | **- Level** | **Score Mean Difference** | **Std Err Dif** | **Z** | **p-Value** | **Hodges-Lehmann** | **Lower CL** | **Upper CL** | **Difference Plot** |
| --- | --- | --- | --- | --- | --- | --- | --- | --- | --- |
| 3/4&5 | 1/2 | 0,4642857 | 4,704355 | 0,0986928 | 0,9214 | 0,7056281 | -12,6165 | 15,40431 |  |

**Oneway Analysis of original-glcm-ClusterProminence By GrG <=2 vs >=3**

**Quantiles**

| **Level** | **Minimum** | **10%** | **25%** | **Median** | **75%** | **90%** | **Maximum** |
| --- | --- | --- | --- | --- | --- | --- | --- |
| 1/2 | 5,141301 | 12988,72 | 18141,12 | 41388,17 | 108175,8 | 241658,6 | 551451,9 |
| 3/4&5 | 6306,547 | 10649,11 | 22100,47 | 43914,06 | 120907,4 | 380588,3 | 864685,9 |

**Oneway Anova**

**Summary of Fit**

| Rsquare | 0,013504 |
| --- | --- |
| Adj Rsquare | -0,00215 |
| Root Mean Square Error | 166414,9 |
| Mean of Response | 108634,7 |
| Observations (or Sum Wgts) | 65 |

**Pooled t Test**

3/4&5-1/2

Assuming equal variances

| Difference | 38451 | t Ratio | 0,928657 |
| --- | --- | --- | --- |
| Std Err Dif | 41405 | DF | 63 |
| Upper CL Dif | 121193 | Prob > |t| | 0,3566 |
| Lower CL Dif | -44290 | Prob > t | 0,1783 |
| Confidence | 0,95 | Prob < t | 0,8217 |

**Analysis of Variance**

| **Source** | **DF** | **Sum of Squares** | **Mean Square** | **F Ratio** | **Prob > F** |
| --- | --- | --- | --- | --- | --- |
| GrG <=2 vs >=3 | 1 | 2,3883e+10 | 2,388e+10 | 0,8624 | 0,3566 |
| Error | 63 | 1,7447e+12 | 2,769e+10 |  |  |
| C. Total | 64 | 1,7686e+12 |  |  |  |

**Means for Oneway Anova**

| **Level** | **Number** | **Mean** | **Std Error** | **Lower 95%** | **Upper 95%** |
| --- | --- | --- | --- | --- | --- |
| 1/2 | 30 | 87930 | 30383 | 27215 | 148646 |
| 3/4&5 | 35 | 126381 | 28129 | 70170 | 182593 |

Std Error uses a pooled estimate of error variance

**Nonparametric Comparisons For Each Pair Using Wilcoxon Method**

| **q*** | **Alpha** |
| --- | --- |
| 1,95996 | 0,05 |

| **Level** | **- Level** | **Score Mean Difference** | **Std Err Dif** | **Z** | **p-Value** | **Hodges-Lehmann** | **Lower CL** | **Upper CL** | **Difference Plot** |
| --- | --- | --- | --- | --- | --- | --- | --- | --- | --- |
| 3/4&5 | 1/2 | 2,197619 | 4,704355 | 0,4671457 | 0,6404 | 5443,668 | -17641,9 | 29772,39 |  |

**Oneway Analysis of original-glcm-Imc2 By GrG <=2 vs >=3**

**Quantiles**

| **Level** | **Minimum** | **10%** | **25%** | **Median** | **75%** | **90%** | **Maximum** |
| --- | --- | --- | --- | --- | --- | --- | --- |
| 1/2 | 0,675327 | 0,825033 | 0,967833 | 0,992589 | 0,99745 | 0,998612 | 0,998706 |
| 3/4&5 | 0,695685 | 0,822489 | 0,898391 | 0,982343 | 0,994386 | 0,99755 | 0,998683 |

**Oneway Anova**

**Summary of Fit**

| Rsquare | 0,014475 |
| --- | --- |
| Adj Rsquare | -0,00117 |
| Root Mean Square Error | 0,075539 |
| Mean of Response | 0,950353 |
| Observations (or Sum Wgts) | 65 |

**Pooled t Test**

3/4&5-1/2

Assuming equal variances

| Difference | -0,01808 | t Ratio | -0,96193 |
| --- | --- | --- | --- |
| Std Err Dif | 0,01879 | DF | 63 |
| Upper CL Dif | 0,01948 | Prob > |t| | 0,3398 |
| Lower CL Dif | -0,05564 | Prob > t | 0,8301 |
| Confidence | 0,95 | Prob < t | 0,1699 |

**Analysis of Variance**

| **Source** | **DF** | **Sum of Squares** | **Mean Square** | **F Ratio** | **Prob > F** |
| --- | --- | --- | --- | --- | --- |
| GrG <=2 vs >=3 | 1 | 0,00527992 | 0,005280 | 0,9253 | 0,3398 |
| Error | 63 | 0,35948366 | 0,005706 |  |  |
| C. Total | 64 | 0,36476358 |  |  |  |

**Means for Oneway Anova**

| **Level** | **Number** | **Mean** | **Std Error** | **Lower 95%** | **Upper 95%** |
| --- | --- | --- | --- | --- | --- |
| 1/2 | 30 | 0,960088 | 0,01379 | 0,93253 | 0,98765 |
| 3/4&5 | 35 | 0,942009 | 0,01277 | 0,91649 | 0,96752 |

Std Error uses a pooled estimate of error variance

**Nonparametric Comparisons For Each Pair Using Wilcoxon Method**

| **q*** | **Alpha** |
| --- | --- |
| 1,95996 | 0,05 |

| **Level** | **- Level** | **Score Mean Difference** | **Std Err Dif** | **Z** | **p-Value** | **Hodges-Lehmann** | **Lower CL** | **Upper CL** | **Difference Plot** |
| --- | --- | --- | --- | --- | --- | --- | --- | --- | --- |
| 3/4&5 | 1/2 | -7,08810 | 4,704355 | -1,50671 | 0,1319 | -0,004606 | -0,018596 | 0,0013336 |  |

**Oneway Analysis of original-glcm-Imc1 By GrG <=2 vs >=3**

**Quantiles**

| **Level** | **Minimum** | **10%** | **25%** | **Median** | **75%** | **90%** | **Maximum** |
| --- | --- | --- | --- | --- | --- | --- | --- |
| 1/2 | -0,80779 | -0,69095 | -0,61671 | -0,50574 | -0,29779 | -0,15857 | -0,10946 |
| 3/4&5 | -0,85465 | -0,65793 | -0,48336 | -0,38276 | -0,19097 | -0,11616 | -0,07315 |

**Oneway Anova**

**Summary of Fit**

| Rsquare | 0,051171 |
| --- | --- |
| Adj Rsquare | 0,03611 |
| Root Mean Square Error | 0,198535 |
| Mean of Response | -0,41273 |
| Observations (or Sum Wgts) | 65 |

**Pooled t Test**

3/4&5-1/2

Assuming equal variances

| Difference | 0,09105 | t Ratio | 1,843271 |
| --- | --- | --- | --- |
| Std Err Dif | 0,04940 | DF | 63 |
| Upper CL Dif | 0,18976 | Prob > |t| | 0,0700 |
| Lower CL Dif | -0,00766 | Prob > t | 0,0350* |
| Confidence | 0,95 | Prob < t | 0,9650 |

**Analysis of Variance**

| **Source** | **DF** | **Sum of Squares** | **Mean Square** | **F Ratio** | **Prob > F** |
| --- | --- | --- | --- | --- | --- |
| GrG <=2 vs >=3 | 1 | 0,1339222 | 0,133922 | 3,3976 | 0,0700 |
| Error | 63 | 2,4832192 | 0,039416 |  |  |
| C. Total | 64 | 2,6171415 |  |  |  |

**Means for Oneway Anova**

| **Level** | **Number** | **Mean** | **Std Error** | **Lower 95%** | **Upper 95%** |
| --- | --- | --- | --- | --- | --- |
| 1/2 | 30 | -0,46176 | 0,03625 | -0,5342 | -0,3893 |
| 3/4&5 | 35 | -0,37070 | 0,03356 | -0,4378 | -0,3036 |

Std Error uses a pooled estimate of error variance

**Nonparametric Comparisons For Each Pair Using Wilcoxon Method**

| **q*** | **Alpha** |
| --- | --- |
| 1,95996 | 0,05 |

| **Level** | **- Level** | **Score Mean Difference** | **Std Err Dif** | **Z** | **p-Value** | **Hodges-Lehmann** | **Lower CL** | **Upper CL** | **Difference Plot** |
| --- | --- | --- | --- | --- | --- | --- | --- | --- | --- |
| 3/4&5 | 1/2 | 9,069048 | 4,704355 | 1,927798 | 0,0539 | 0,1074535 | -0,002313 | 0,2062861 |  |

**Oneway Analysis of original-glcm-DifferenceAverage By GrG <=2 vs >=3**

**Quantiles**

| **Level** | **Minimum** | **10%** | **25%** | **Median** | **75%** | **90%** | **Maximum** |
| --- | --- | --- | --- | --- | --- | --- | --- |
| 1/2 | 0,688219 | 5,065467 | 5,303898 | 6,425913 | 7,906604 | 9,265981 | 10,59021 |
| 3/4&5 | 4,836442 | 5,009507 | 5,558504 | 6,299376 | 7,567884 | 9,176033 | 11,00236 |

**Oneway Anova**

**Summary of Fit**

| Rsquare | 0,0014 |
| --- | --- |
| Adj Rsquare | -0,01445 |
| Root Mean Square Error | 1,736242 |
| Mean of Response | 6,678715 |
| Observations (or Sum Wgts) | 65 |

**Pooled t Test**

3/4&5-1/2

Assuming equal variances

| Difference | 0,12838 | t Ratio | 0,297179 |
| --- | --- | --- | --- |
| Std Err Dif | 0,43199 | DF | 63 |
| Upper CL Dif | 0,99164 | Prob > |t| | 0,7673 |
| Lower CL Dif | -0,73488 | Prob > t | 0,3837 |
| Confidence | 0,95 | Prob < t | 0,6163 |

**Analysis of Variance**

| **Source** | **DF** | **Sum of Squares** | **Mean Square** | **F Ratio** | **Prob > F** |
| --- | --- | --- | --- | --- | --- |
| GrG <=2 vs >=3 | 1 | 0,26623 | 0,26623 | 0,0883 | 0,7673 |
| Error | 63 | 189,91583 | 3,01454 |  |  |
| C. Total | 64 | 190,18206 |  |  |  |

**Means for Oneway Anova**

| **Level** | **Number** | **Mean** | **Std Error** | **Lower 95%** | **Upper 95%** |
| --- | --- | --- | --- | --- | --- |
| 1/2 | 30 | 6,60959 | 0,31699 | 5,9761 | 7,2430 |
| 3/4&5 | 35 | 6,73797 | 0,29348 | 6,1515 | 7,3244 |

Std Error uses a pooled estimate of error variance

**Nonparametric Comparisons For Each Pair Using Wilcoxon Method**

| **q*** | **Alpha** |
| --- | --- |
| 1,95996 | 0,05 |

| **Level** | **- Level** | **Score Mean Difference** | **Std Err Dif** | **Z** | **p-Value** | **Hodges-Lehmann** | **Lower CL** | **Upper CL** | **Difference Plot** |
| --- | --- | --- | --- | --- | --- | --- | --- | --- | --- |
| 3/4&5 | 1/2 | 0,2166667 | 4,704355 | 0,0460566 | 0,9633 | 0,0192041 | -0,775248 | 0,7340513 |  |

**Oneway Analysis of original-glcm-Id By GrG <=2 vs >=3**

**Quantiles**

| **Level** | **Minimum** | **10%** | **25%** | **Median** | **75%** | **90%** | **Maximum** |
| --- | --- | --- | --- | --- | --- | --- | --- |
| 1/2 | 0,194069 | 0,203236 | 0,227215 | 0,247619 | 0,276539 | 0,289009 | 0,686402 |
| 3/4&5 | 0,190995 | 0,206207 | 0,23209 | 0,245628 | 0,266763 | 0,281975 | 0,3039 |

**Oneway Anova**

**Summary of Fit**

| Rsquare | 0,018616 |
| --- | --- |
| Adj Rsquare | 0,003038 |
| Root Mean Square Error | 0,061849 |
| Mean of Response | 0,254189 |
| Observations (or Sum Wgts) | 65 |

**Pooled t Test**

3/4&5-1/2

Assuming equal variances

| Difference | -0,01682 | t Ratio | -1,09318 |
| --- | --- | --- | --- |
| Std Err Dif | 0,01539 | DF | 63 |
| Upper CL Dif | 0,01393 | Prob > |t| | 0,2785 |
| Lower CL Dif | -0,04757 | Prob > t | 0,8608 |
| Confidence | 0,95 | Prob < t | 0,1392 |

**Analysis of Variance**

| **Source** | **DF** | **Sum of Squares** | **Mean Square** | **F Ratio** | **Prob > F** |
| --- | --- | --- | --- | --- | --- |
| GrG <=2 vs >=3 | 1 | 0,00457136 | 0,004571 | 1,1950 | 0,2785 |
| Error | 63 | 0,24099326 | 0,003825 |  |  |
| C. Total | 64 | 0,24556462 |  |  |  |

**Means for Oneway Anova**

| **Level** | **Number** | **Mean** | **Std Error** | **Lower 95%** | **Upper 95%** |
| --- | --- | --- | --- | --- | --- |
| 1/2 | 30 | 0,263247 | 0,01129 | 0,24068 | 0,28581 |
| 3/4&5 | 35 | 0,246425 | 0,01045 | 0,22553 | 0,26732 |

Std Error uses a pooled estimate of error variance

**Nonparametric Comparisons For Each Pair Using Wilcoxon Method**

| **q*** | **Alpha** |
| --- | --- |
| 1,95996 | 0,05 |

| **Level** | **- Level** | **Score Mean Difference** | **Std Err Dif** | **Z** | **p-Value** | **Hodges-Lehmann** | **Lower CL** | **Upper CL** | **Difference Plot** |
| --- | --- | --- | --- | --- | --- | --- | --- | --- | --- |
| 3/4&5 | 1/2 | -1,64048 | 4,704355 | -0,348714 | 0,7273 | -0,002266 | -0,019242 | 0,0127378 |  |

**Oneway Analysis of original-glcm-ClusterTendency By GrG <=2 vs >=3**

**Quantiles**

| **Level** | **Minimum** | **10%** | **25%** | **Median** | **75%** | **90%** | **Maximum** |
| --- | --- | --- | --- | --- | --- | --- | --- |
| 1/2 | 1,508091 | 65,32315 | 81,39555 | 117,6041 | 191,6357 | 316,84 | 441,1564 |
| 3/4&5 | 42,48593 | 55,26187 | 81,97091 | 121,8216 | 207,0911 | 320,0184 | 487,5625 |

**Oneway Anova**

**Summary of Fit**

| Rsquare | 0,004502 |
| --- | --- |
| Adj Rsquare | -0,0113 |
| Root Mean Square Error | 105,3303 |
| Mean of Response | 152,4097 |
| Observations (or Sum Wgts) | 65 |

**Pooled t Test**

3/4&5-1/2

Assuming equal variances

| Difference | 13,988 | t Ratio | 0,533748 |
| --- | --- | --- | --- |
| Std Err Dif | 26,207 | DF | 63 |
| Upper CL Dif | 66,358 | Prob > |t| | 0,5954 |
| Lower CL Dif | -38,382 | Prob > t | 0,2977 |
| Confidence | 0,95 | Prob < t | 0,7023 |

**Analysis of Variance**

| **Source** | **DF** | **Sum of Squares** | **Mean Square** | **F Ratio** | **Prob > F** |
| --- | --- | --- | --- | --- | --- |
| GrG <=2 vs >=3 | 1 | 3160,67 | 3160,7 | 0,2849 | 0,5954 |
| Error | 63 | 698952,04 | 11094,5 |  |  |
| C. Total | 64 | 702112,72 |  |  |  |

**Means for Oneway Anova**

| **Level** | **Number** | **Mean** | **Std Error** | **Lower 95%** | **Upper 95%** |
| --- | --- | --- | --- | --- | --- |
| 1/2 | 30 | 144,878 | 19,231 | 106,45 | 183,31 |
| 3/4&5 | 35 | 158,866 | 17,804 | 123,29 | 194,44 |

Std Error uses a pooled estimate of error variance

**Nonparametric Comparisons For Each Pair Using Wilcoxon Method**

| **q*** | **Alpha** |
| --- | --- |
| 1,95996 | 0,05 |

| **Level** | **- Level** | **Score Mean Difference** | **Std Err Dif** | **Z** | **p-Value** | **Hodges-Lehmann** | **Lower CL** | **Upper CL** | **Difference Plot** |
| --- | --- | --- | --- | --- | --- | --- | --- | --- | --- |
| 3/4&5 | 1/2 | 1,578571 | 4,704355 | 0,3355554 | 0,7372 | 6,115411 | -30,1416 | 42,68415 |  |

**Oneway Analysis of original-firstorder-InterquartileRange By GrG <=2 vs >=3**

**Quantiles**

| **Level** | **Minimum** | **10%** | **25%** | **Median** | **75%** | **90%** | **Maximum** |
| --- | --- | --- | --- | --- | --- | --- | --- |
| 1/2 | 29,5 | 171,125 | 196,375 | 246,625 | 293,1875 | 386,675 | 508 |
| 3/4&5 | 155 | 170,55 | 188 | 229 | 302,75 | 405,05 | 502 |

**Oneway Anova**

**Summary of Fit**

| Rsquare | 0,000342 |
| --- | --- |
| Adj Rsquare | -0,01553 |
| Root Mean Square Error | 88,49263 |
| Mean of Response | 257,0577 |
| Observations (or Sum Wgts) | 65 |

**Pooled t Test**

3/4&5-1/2

Assuming equal variances

| Difference | 3,233 | t Ratio | 0,146852 |
| --- | --- | --- | --- |
| Std Err Dif | 22,018 | DF | 63 |
| Upper CL Dif | 47,232 | Prob > |t| | 0,8837 |
| Lower CL Dif | -40,765 | Prob > t | 0,4419 |
| Confidence | 0,95 | Prob < t | 0,5581 |

**Analysis of Variance**

| **Source** | **DF** | **Sum of Squares** | **Mean Square** | **F Ratio** | **Prob > F** |
| --- | --- | --- | --- | --- | --- |
| GrG <=2 vs >=3 | 1 | 168,88 | 168,88 | 0,0216 | 0,8837 |
| Error | 63 | 493349,59 | 7830,95 |  |  |
| C. Total | 64 | 493518,47 |  |  |  |

**Means for Oneway Anova**

| **Level** | **Number** | **Mean** | **Std Error** | **Lower 95%** | **Upper 95%** |
| --- | --- | --- | --- | --- | --- |
| 1/2 | 30 | 255,317 | 16,156 | 223,03 | 287,60 |
| 3/4&5 | 35 | 258,550 | 14,958 | 228,66 | 288,44 |

Std Error uses a pooled estimate of error variance

**Nonparametric Comparisons For Each Pair Using Wilcoxon Method**

| **q*** | **Alpha** |
| --- | --- |
| 1,95996 | 0,05 |

| **Level** | **- Level** | **Score Mean Difference** | **Std Err Dif** | **Z** | **p-Value** | **Hodges-Lehmann** | **Lower CL** | **Upper CL** | **Difference Plot** |
| --- | --- | --- | --- | --- | --- | --- | --- | --- | --- |
| 3/4&5 | 1/2 | -1,20714 | 4,704200 | -0,256610 | 0,7975 | -6,25000 | -42,0000 | 33,00000 |  |

**Oneway Analysis of original-firstorder-Skewness By GrG <=2 vs >=3**

**Quantiles**

| **Level** | **Minimum** | **10%** | **25%** | **Median** | **75%** | **90%** | **Maximum** |
| --- | --- | --- | --- | --- | --- | --- | --- |
| 1/2 | -0,84298 | -0,5038 | -0,07657 | 0,164594 | 0,336941 | 0,62175 | 0,973451 |
| 3/4&5 | -0,86303 | -0,76285 | -0,15947 | 0,174569 | 0,463517 | 0,725149 | 1,321986 |

**Oneway Anova**

**Summary of Fit**

| Rsquare | 0,001083 |
| --- | --- |
| Adj Rsquare | -0,01477 |
| Root Mean Square Error | 0,445272 |
| Mean of Response | 0,142791 |
| Observations (or Sum Wgts) | 65 |

**Pooled t Test**

3/4&5-1/2

Assuming equal variances

| Difference | 0,02895 | t Ratio | 0,261297 |
| --- | --- | --- | --- |
| Std Err Dif | 0,11079 | DF | 63 |
| Upper CL Dif | 0,25034 | Prob > |t| | 0,7947 |
| Lower CL Dif | -0,19244 | Prob > t | 0,3974 |
| Confidence | 0,95 | Prob < t | 0,6026 |

**Analysis of Variance**

| **Source** | **DF** | **Sum of Squares** | **Mean Square** | **F Ratio** | **Prob > F** |
| --- | --- | --- | --- | --- | --- |
| GrG <=2 vs >=3 | 1 | 0,013537 | 0,013537 | 0,0683 | 0,7947 |
| Error | 63 | 12,490835 | 0,198267 |  |  |
| C. Total | 64 | 12,504372 |  |  |  |

**Means for Oneway Anova**

| **Level** | **Number** | **Mean** | **Std Error** | **Lower 95%** | **Upper 95%** |
| --- | --- | --- | --- | --- | --- |
| 1/2 | 30 | 0,127204 | 0,08130 | -0,0353 | 0,28966 |
| 3/4&5 | 35 | 0,156152 | 0,07526 | 0,0057 | 0,30656 |

Std Error uses a pooled estimate of error variance

**Nonparametric Comparisons For Each Pair Using Wilcoxon Method**

| **q*** | **Alpha** |
| --- | --- |
| 1,95996 | 0,05 |

| **Level** | **- Level** | **Score Mean Difference** | **Std Err Dif** | **Z** | **p-Value** | **Hodges-Lehmann** | **Lower CL** | **Upper CL** | **Difference Plot** |
| --- | --- | --- | --- | --- | --- | --- | --- | --- | --- |
| 3/4&5 | 1/2 | 1,640476 | 4,704355 | 0,3487144 | 0,7273 | 0,0336456 | -0,163967 | 0,2394405 |  |

**Oneway Analysis of original-firstorder-Uniformity By GrG <=2 vs >=3**

**Quantiles**

| **Level** | **Minimum** | **10%** | **25%** | **Median** | **75%** | **90%** | **Maximum** |
| --- | --- | --- | --- | --- | --- | --- | --- |
| 1/2 | 0,024622 | 0,035849 | 0,040273 | 0,051868 | 0,057385 | 0,095189 | 0,338162 |
| 3/4&5 | 0,023847 | 0,029815 | 0,035588 | 0,045903 | 0,056044 | 0,069032 | 0,095156 |

**Oneway Anova**

**Summary of Fit**

| Rsquare | 0,030137 |
| --- | --- |
| Adj Rsquare | 0,014742 |
| Root Mean Square Error | 0,039203 |
| Mean of Response | 0,05473 |
| Observations (or Sum Wgts) | 65 |

**Pooled t Test**

3/4&5-1/2

Assuming equal variances

| Difference | -0,01365 | t Ratio | -1,39915 |
| --- | --- | --- | --- |
| Std Err Dif | 0,00975 | DF | 63 |
| Upper CL Dif | 0,00584 | Prob > |t| | 0,1667 |
| Lower CL Dif | -0,03314 | Prob > t | 0,9167 |
| Confidence | 0,95 | Prob < t | 0,0833 |

**Analysis of Variance**

| **Source** | **DF** | **Sum of Squares** | **Mean Square** | **F Ratio** | **Prob > F** |
| --- | --- | --- | --- | --- | --- |
| GrG <=2 vs >=3 | 1 | 0,00300863 | 0,003009 | 1,9576 | 0,1667 |
| Error | 63 | 0,09682382 | 0,001537 |  |  |
| C. Total | 64 | 0,09983245 |  |  |  |

**Means for Oneway Anova**

| **Level** | **Number** | **Mean** | **Std Error** | **Lower 95%** | **Upper 95%** |
| --- | --- | --- | --- | --- | --- |
| 1/2 | 30 | 0,062079 | 0,00716 | 0,04778 | 0,07638 |
| 3/4&5 | 35 | 0,048431 | 0,00663 | 0,03519 | 0,06167 |

Std Error uses a pooled estimate of error variance

**Nonparametric Comparisons For Each Pair Using Wilcoxon Method**

| **q*** | **Alpha** |
| --- | --- |
| 1,95996 | 0,05 |

| **Level** | **- Level** | **Score Mean Difference** | **Std Err Dif** | **Z** | **p-Value** | **Hodges-Lehmann** | **Lower CL** | **Upper CL** | **Difference Plot** |
| --- | --- | --- | --- | --- | --- | --- | --- | --- | --- |
| 3/4&5 | 1/2 | -6,80952 | 4,704303 | -1,44751 | 0,1478 | -0,004839 | -0,012137 | 0,0025753 |  |

**Oneway Analysis of original-firstorder-Median By GrG <=2 vs >=3**

**Quantiles**

| **Level** | **Minimum** | **10%** | **25%** | **Median** | **75%** | **90%** | **Maximum** |
| --- | --- | --- | --- | --- | --- | --- | --- |
| 1/2 | 158 | 593,95 | 789,5 | 975 | 1084 | 1189,95 | 1334 |
| 3/4&5 | 538 | 627,7 | 776 | 859 | 983 | 1029,6 | 1092 |

**Oneway Anova**

**Summary of Fit**

| Rsquare | 0,034701 |
| --- | --- |
| Adj Rsquare | 0,019378 |
| Root Mean Square Error | 198,3017 |
| Mean of Response | 884,3692 |
| Observations (or Sum Wgts) | 65 |

**Pooled t Test**

3/4&5-1/2

Assuming equal variances

| Difference | -74,25 | t Ratio | -1,5049 |
| --- | --- | --- | --- |
| Std Err Dif | 49,34 | DF | 63 |
| Upper CL Dif | 24,35 | Prob > |t| | 0,1373 |
| Lower CL Dif | -172,85 | Prob > t | 0,9313 |
| Confidence | 0,95 | Prob < t | 0,0687 |

**Analysis of Variance**

| **Source** | **DF** | **Sum of Squares** | **Mean Square** | **F Ratio** | **Prob > F** |
| --- | --- | --- | --- | --- | --- |
| GrG <=2 vs >=3 | 1 | 89057,2 | 89057,2 | 2,2647 | 0,1373 |
| Error | 63 | 2477385,5 | 39323,6 |  |  |
| C. Total | 64 | 2566442,6 |  |  |  |

**Means for Oneway Anova**

| **Level** | **Number** | **Mean** | **Std Error** | **Lower 95%** | **Upper 95%** |
| --- | --- | --- | --- | --- | --- |
| 1/2 | 30 | 924,350 | 36,205 | 852,00 | 996,70 |
| 3/4&5 | 35 | 850,100 | 33,519 | 783,12 | 917,08 |

Std Error uses a pooled estimate of error variance

**Nonparametric Comparisons For Each Pair Using Wilcoxon Method**

| **q*** | **Alpha** |
| --- | --- |
| 1,95996 | 0,05 |

| **Level** | **- Level** | **Score Mean Difference** | **Std Err Dif** | **Z** | **p-Value** | **Hodges-Lehmann** | **Lower CL** | **Upper CL** | **Difference Plot** |
| --- | --- | --- | --- | --- | --- | --- | --- | --- | --- |
| 3/4&5 | 1/2 | -8,88333 | 4,704303 | -1,88834 | 0,0590 | -97,5000 | -186,000 | 7,000000 |  |

**Oneway Analysis of original-firstorder-Energy By GrG <=2 vs >=3**

**Quantiles**

| **Level** | **Minimum** | **10%** | **25%** | **Median** | **75%** | **90%** | **Maximum** |
| --- | --- | --- | --- | --- | --- | --- | --- |
| 1/2 | 1236184 | 36717249 | 73385290 | 1,184e+8 | 3,522e+8 | 9,051e+8 | 3,998e+9 |
| 3/4&5 | 25692711 | 49093311 | 99317634 | 1,782e+8 | 4,175e+8 | 2,509e+9 | 2,1e+10 |

**Oneway Anova**

**Summary of Fit**

| Rsquare | 0,018791 |
| --- | --- |
| Adj Rsquare | 0,003216 |
| Root Mean Square Error | 2,683e+9 |
| Mean of Response | 7,645e+8 |
| Observations (or Sum Wgts) | 65 |

**Pooled t Test**

3/4&5-1/2

Assuming equal variances

| Difference | 733375481 | t Ratio | 1,098414 |
| --- | --- | --- | --- |
| Std Err Dif | 667667409 | DF | 63 |
| Upper CL Dif | 2,0676e+9 | Prob > |t| | 0,2762 |
| Lower CL Dif | -6,009e+8 | Prob > t | 0,1381 |
| Confidence | 0,95 | Prob < t | 0,8619 |

**Analysis of Variance**

| **Source** | **DF** | **Sum of Squares** | **Mean Square** | **F Ratio** | **Prob > F** |
| --- | --- | --- | --- | --- | --- |
| GrG <=2 vs >=3 | 1 | 8,6882e+18 | 8,688e+18 | 1,2065 | 0,2762 |
| Error | 63 | 4,5367e+20 | 7,201e+18 |  |  |
| C. Total | 64 | 4,6235e+20 |  |  |  |

**Means for Oneway Anova**

| **Level** | **Number** | **Mean** | **Std Error** | **Lower 95%** | **Upper 95%** |
| --- | --- | --- | --- | --- | --- |
| 1/2 | 30 | 369606006 | 489933935 | -6,094e+8 | 1,3487e+9 |
| 3/4&5 | 35 | 1,103e+9 | 453590684 | 196552833 | 2,0094e+9 |

Std Error uses a pooled estimate of error variance

**Nonparametric Comparisons For Each Pair Using Wilcoxon Method**

| **q*** | **Alpha** |
| --- | --- |
| 1,95996 | 0,05 |

| **Level** | **- Level** | **Score Mean Difference** | **Std Err Dif** | **Z** | **p-Value** | **Hodges-Lehmann** | **Lower CL** | **Upper CL** | **Difference Plot** |
| --- | --- | --- | --- | --- | --- | --- | --- | --- | --- |
| 3/4&5 | 1/2 | 6,716667 | 4,704355 | 1,427755 | 0,1534 | 46741023 | -20380549 | 139794567 |  |

**Oneway Analysis of original-firstorder-RobustMeanAbsoluteDeviation By GrG <=2 vs >=3**

**Quantiles**

| **Level** | **Minimum** | **10%** | **25%** | **Median** | **75%** | **90%** | **Maximum** |
| --- | --- | --- | --- | --- | --- | --- | --- |
| 1/2 | 13,57618 | 72,39618 | 87,65439 | 101,7667 | 122,7706 | 168,5905 | 201,0735 |
| 3/4&5 | 63,00053 | 73,6279 | 78,9651 | 97,46814 | 126,4278 | 170,1944 | 200,1718 |

**Oneway Anova**

**Summary of Fit**

| Rsquare | 0,000145 |
| --- | --- |
| Adj Rsquare | -0,01573 |
| Root Mean Square Error | 36,11905 |
| Mean of Response | 107,7035 |
| Observations (or Sum Wgts) | 65 |

**Pooled t Test**

3/4&5-1/2

Assuming equal variances

| Difference | 0,858 | t Ratio | 0,095439 |
| --- | --- | --- | --- |
| Std Err Dif | 8,987 | DF | 63 |
| Upper CL Dif | 18,816 | Prob > |t| | 0,9243 |
| Lower CL Dif | -17,101 | Prob > t | 0,4621 |
| Confidence | 0,95 | Prob < t | 0,5379 |

**Analysis of Variance**

| **Source** | **DF** | **Sum of Squares** | **Mean Square** | **F Ratio** | **Prob > F** |
| --- | --- | --- | --- | --- | --- |
| GrG <=2 vs >=3 | 1 | 11,883 | 11,88 | 0,0091 | 0,9243 |
| Error | 63 | 82188,906 | 1304,59 |  |  |
| C. Total | 64 | 82200,789 |  |  |  |

**Means for Oneway Anova**

| **Level** | **Number** | **Mean** | **Std Error** | **Lower 95%** | **Upper 95%** |
| --- | --- | --- | --- | --- | --- |
| 1/2 | 30 | 107,242 | 6,5944 | 94,064 | 120,42 |
| 3/4&5 | 35 | 108,099 | 6,1052 | 95,899 | 120,30 |

Std Error uses a pooled estimate of error variance

**Nonparametric Comparisons For Each Pair Using Wilcoxon Method**

| **q*** | **Alpha** |
| --- | --- |
| 1,95996 | 0,05 |

| **Level** | **- Level** | **Score Mean Difference** | **Std Err Dif** | **Z** | **p-Value** | **Hodges-Lehmann** | **Lower CL** | **Upper CL** | **Difference Plot** |
| --- | --- | --- | --- | --- | --- | --- | --- | --- | --- |
| 3/4&5 | 1/2 | -1,39286 | 4,704355 | -0,296078 | 0,7672 | -1,98628 | -15,8970 | 14,15761 |  |

**Oneway Analysis of original-firstorder-MeanAbsoluteDeviation By GrG <=2 vs >=3**

**Quantiles**

| **Level** | **Minimum** | **10%** | **25%** | **Median** | **75%** | **90%** | **Maximum** |
| --- | --- | --- | --- | --- | --- | --- | --- |
| 1/2 | 17,37347 | 106,6118 | 121,5769 | 146,3807 | 172,1218 | 228,6278 | 269,5574 |
| 3/4&5 | 94,40554 | 101,8013 | 119,1727 | 145,1578 | 177,6733 | 234,2368 | 277,1082 |

**Oneway Anova**

**Summary of Fit**

| Rsquare | 0,001347 |
| --- | --- |
| Adj Rsquare | -0,0145 |
| Root Mean Square Error | 47,48978 |
| Mean of Response | 151,8059 |
| Observations (or Sum Wgts) | 65 |

**Pooled t Test**

3/4&5-1/2

Assuming equal variances

| Difference | 3,444 | t Ratio | 0,29151 |
| --- | --- | --- | --- |
| Std Err Dif | 11,816 | DF | 63 |
| Upper CL Dif | 27,056 | Prob > |t| | 0,7716 |
| Lower CL Dif | -20,168 | Prob > t | 0,3858 |
| Confidence | 0,95 | Prob < t | 0,6142 |

**Analysis of Variance**

| **Source** | **DF** | **Sum of Squares** | **Mean Square** | **F Ratio** | **Prob > F** |
| --- | --- | --- | --- | --- | --- |
| GrG <=2 vs >=3 | 1 | 191,65 | 191,65 | 0,0850 | 0,7716 |
| Error | 63 | 142082,61 | 2255,28 |  |  |
| C. Total | 64 | 142274,26 |  |  |  |

**Means for Oneway Anova**

| **Level** | **Number** | **Mean** | **Std Error** | **Lower 95%** | **Upper 95%** |
| --- | --- | --- | --- | --- | --- |
| 1/2 | 30 | 149,951 | 8,6704 | 132,62 | 167,28 |
| 3/4&5 | 35 | 153,396 | 8,0272 | 137,35 | 169,44 |

Std Error uses a pooled estimate of error variance

**Nonparametric Comparisons For Each Pair Using Wilcoxon Method**

| **q*** | **Alpha** |
| --- | --- |
| 1,95996 | 0,05 |

| **Level** | **- Level** | **Score Mean Difference** | **Std Err Dif** | **Z** | **p-Value** | **Hodges-Lehmann** | **Lower CL** | **Upper CL** | **Difference Plot** |
| --- | --- | --- | --- | --- | --- | --- | --- | --- | --- |
| 3/4&5 | 1/2 | -0,464286 | 4,704355 | -0,098693 | 0,9214 | -0,944395 | -20,2705 | 19,31780 |  |

**Oneway Analysis of original-firstorder-TotalEnergy By GrG <=2 vs >=3**

**Quantiles**

| **Level** | **Minimum** | **10%** | **25%** | **Median** | **75%** | **90%** | **Maximum** |
| --- | --- | --- | --- | --- | --- | --- | --- |
| 1/2 | 7934058 | 2,35e+8 | 4,759e+8 | 7,938e+8 | 2,065e+9 | 5,646e+9 | 2,56e+10 |
| 3/4&5 | 1,644e+8 | 3,142e+8 | 6,888e+8 | 1,045e+9 | 2,672e+9 | 1,61e+10 | 1,8e+11 |

**Oneway Anova**

**Summary of Fit**

| Rsquare | 0,01904 |
| --- | --- |
| Adj Rsquare | 0,003469 |
| Root Mean Square Error | 2,26e+10 |
| Mean of Response | 5,688e+9 |
| Observations (or Sum Wgts) | 65 |

**Pooled t Test**

3/4&5-1/2

Assuming equal variances

| Difference | 6,2193e+9 | t Ratio | 1,105796 |
| --- | --- | --- | --- |
| Std Err Dif | 5,6243e+9 | DF | 63 |
| Upper CL Dif | 1,746e+10 | Prob > |t| | 0,2730 |
| Lower CL Dif | -5,02e+9 | Prob > t | 0,1365 |
| Confidence | 0,95 | Prob < t | 0,8635 |

**Analysis of Variance**

| **Source** | **DF** | **Sum of Squares** | **Mean Square** | **F Ratio** | **Prob > F** |
| --- | --- | --- | --- | --- | --- |
| GrG <=2 vs >=3 | 1 | 6,2483e+20 | 6,248e+20 | 1,2228 | 0,2730 |
| Error | 63 | 3,2192e+22 | 5,11e+20 |  |  |
| C. Total | 64 | 3,2817e+22 |  |  |  |

**Means for Oneway Anova**

| **Level** | **Number** | **Mean** | **Std Error** | **Lower 95%** | **Upper 95%** |
| --- | --- | --- | --- | --- | --- |
| 1/2 | 30 | 2,3394e+9 | 4,1271e+9 | -5,908e+9 | 1,059e+10 |
| 3/4&5 | 35 | 8,5587e+9 | 3,8209e+9 | 923187592 | 1,619e+10 |

Std Error uses a pooled estimate of error variance

**Nonparametric Comparisons For Each Pair Using Wilcoxon Method**

| **q*** | **Alpha** |
| --- | --- |
| 1,95996 | 0,05 |

| **Level** | **- Level** | **Score Mean Difference** | **Std Err Dif** | **Z** | **p-Value** | **Hodges-Lehmann** | **Lower CL** | **Upper CL** | **Difference Plot** |
| --- | --- | --- | --- | --- | --- | --- | --- | --- | --- |
| 3/4&5 | 1/2 | 7,707143 | 4,704355 | 1,638300 | 0,1014 | 328845567 | -72603633 | 891054630 |  |

**Oneway Analysis of original-firstorder-Maximum By GrG <=2 vs >=3**

**Quantiles**

| **Level** | **Minimum** | **10%** | **25%** | **Median** | **75%** | **90%** | **Maximum** |
| --- | --- | --- | --- | --- | --- | --- | --- |
| 1/2 | 206 | 1137,2 | 1254,5 | 1408 | 1570,5 | 1963,2 | 2143 |
| 3/4&5 | 1032 | 1063,2 | 1151 | 1372 | 1619 | 2015,8 | 2492 |

**Oneway Anova**

**Summary of Fit**

| Rsquare | 0,000388 |
| --- | --- |
| Adj Rsquare | -0,01548 |
| Root Mean Square Error | 358,9274 |
| Mean of Response | 1422,354 |
| Observations (or Sum Wgts) | 65 |

**Pooled t Test**

3/4&5-1/2

Assuming equal variances

| Difference | 13,97 | t Ratio | 0,156395 |
| --- | --- | --- | --- |
| Std Err Dif | 89,30 | DF | 63 |
| Upper CL Dif | 192,43 | Prob > |t| | 0,8762 |
| Lower CL Dif | -164,49 | Prob > t | 0,4381 |
| Confidence | 0,95 | Prob < t | 0,5619 |

**Analysis of Variance**

| **Source** | **DF** | **Sum of Squares** | **Mean Square** | **F Ratio** | **Prob > F** |
| --- | --- | --- | --- | --- | --- |
| GrG <=2 vs >=3 | 1 | 3151,1 | 3151 | 0,0245 | 0,8762 |
| Error | 63 | 8116217,8 | 128829 |  |  |
| C. Total | 64 | 8119368,9 |  |  |  |

**Means for Oneway Anova**

| **Level** | **Number** | **Mean** | **Std Error** | **Lower 95%** | **Upper 95%** |
| --- | --- | --- | --- | --- | --- |
| 1/2 | 30 | 1414,83 | 65,531 | 1283,9 | 1545,8 |
| 3/4&5 | 35 | 1428,80 | 60,670 | 1307,6 | 1550,0 |

Std Error uses a pooled estimate of error variance

**Nonparametric Comparisons For Each Pair Using Wilcoxon Method**

| **q*** | **Alpha** |
| --- | --- |
| 1,95996 | 0,05 |

| **Level** | **- Level** | **Score Mean Difference** | **Std Err Dif** | **Z** | **p-Value** | **Hodges-Lehmann** | **Lower CL** | **Upper CL** | **Difference Plot** |
| --- | --- | --- | --- | --- | --- | --- | --- | --- | --- |
| 3/4&5 | 1/2 | -2,72381 | 4,704303 | -0,579004 | 0,5626 | -40,5000 | -190,000 | 131,0000 |  |

**Oneway Analysis of original-firstorder-RootMeanSquared By GrG <=2 vs >=3**

**Quantiles**

| **Level** | **Minimum** | **10%** | **25%** | **Median** | **75%** | **90%** | **Maximum** |
| --- | --- | --- | --- | --- | --- | --- | --- |
| 1/2 | 162,1783 | 615,2937 | 830,9906 | 989,4723 | 1105,162 | 1220,57 | 1362,221 |
| 3/4&5 | 561,8323 | 670,0548 | 788,8704 | 881,8707 | 1020,287 | 1093,135 | 1122,147 |

**Oneway Anova**

**Summary of Fit**

| Rsquare | 0,0274 |
| --- | --- |
| Adj Rsquare | 0,011962 |
| Root Mean Square Error | 199,9555 |
| Mean of Response | 913,5233 |
| Observations (or Sum Wgts) | 65 |

**Pooled t Test**

3/4&5-1/2

Assuming equal variances

| Difference | -66,28 | t Ratio | -1,33223 |
| --- | --- | --- | --- |
| Std Err Dif | 49,75 | DF | 63 |
| Upper CL Dif | 33,14 | Prob > |t| | 0,1876 |
| Lower CL Dif | -165,70 | Prob > t | 0,9062 |
| Confidence | 0,95 | Prob < t | 0,0938 |

**Analysis of Variance**

| **Source** | **DF** | **Sum of Squares** | **Mean Square** | **F Ratio** | **Prob > F** |
| --- | --- | --- | --- | --- | --- |
| GrG <=2 vs >=3 | 1 | 70961,8 | 70961,8 | 1,7748 | 0,1876 |
| Error | 63 | 2518879,3 | 39982,2 |  |  |
| C. Total | 64 | 2589841,1 |  |  |  |

**Means for Oneway Anova**

| **Level** | **Number** | **Mean** | **Std Error** | **Lower 95%** | **Upper 95%** |
| --- | --- | --- | --- | --- | --- |
| 1/2 | 30 | 949,212 | 36,507 | 876,26 | 1022,2 |
| 3/4&5 | 35 | 882,933 | 33,799 | 815,39 | 950,5 |

Std Error uses a pooled estimate of error variance

**Nonparametric Comparisons For Each Pair Using Wilcoxon Method**

| **q*** | **Alpha** |
| --- | --- |
| 1,95996 | 0,05 |

| **Level** | **- Level** | **Score Mean Difference** | **Std Err Dif** | **Z** | **p-Value** | **Hodges-Lehmann** | **Lower CL** | **Upper CL** | **Difference Plot** |
| --- | --- | --- | --- | --- | --- | --- | --- | --- | --- |
| 3/4&5 | 1/2 | -8,26429 | 4,704355 | -1,75673 | 0,0790 | -88,8351 | -178,005 | 13,18938 |  |

**Oneway Analysis of original-firstorder-90Percentile By GrG <=2 vs >=3**

**Quantiles**

| **Level** | **Minimum** | **10%** | **25%** | **Median** | **75%** | **90%** | **Maximum** |
| --- | --- | --- | --- | --- | --- | --- | --- |
| 1/2 | 193 | 830,22 | 1002,35 | 1212,4 | 1337,275 | 1463,4 | 1699,2 |
| 3/4&5 | 793,8 | 858,1 | 976,1 | 1082,5 | 1263,9 | 1416,5 | 1537 |

**Oneway Anova**

**Summary of Fit**

| Rsquare | 0,014238 |
| --- | --- |
| Adj Rsquare | -0,00141 |
| Root Mean Square Error | 244,1366 |
| Mean of Response | 1136,469 |
| Observations (or Sum Wgts) | 65 |

**Pooled t Test**

3/4&5-1/2

Assuming equal variances

| Difference | -57,94 | t Ratio | -0,95393 |
| --- | --- | --- | --- |
| Std Err Dif | 60,74 | DF | 63 |
| Upper CL Dif | 63,44 | Prob > |t| | 0,3438 |
| Lower CL Dif | -179,33 | Prob > t | 0,8281 |
| Confidence | 0,95 | Prob < t | 0,1719 |

**Analysis of Variance**

| **Source** | **DF** | **Sum of Squares** | **Mean Square** | **F Ratio** | **Prob > F** |
| --- | --- | --- | --- | --- | --- |
| GrG <=2 vs >=3 | 1 | 54237,2 | 54237,2 | 0,9100 | 0,3438 |
| Error | 63 | 3754968,9 | 59602,7 |  |  |
| C. Total | 64 | 3809206,1 |  |  |  |

**Means for Oneway Anova**

| **Level** | **Number** | **Mean** | **Std Error** | **Lower 95%** | **Upper 95%** |
| --- | --- | --- | --- | --- | --- |
| 1/2 | 30 | 1167,67 | 44,573 | 1078,6 | 1256,7 |
| 3/4&5 | 35 | 1109,73 | 41,267 | 1027,3 | 1192,2 |

Std Error uses a pooled estimate of error variance

**Nonparametric Comparisons For Each Pair Using Wilcoxon Method**

| **q*** | **Alpha** |
| --- | --- |
| 1,95996 | 0,05 |

| **Level** | **- Level** | **Score Mean Difference** | **Std Err Dif** | **Z** | **p-Value** | **Hodges-Lehmann** | **Lower CL** | **Upper CL** | **Difference Plot** |
| --- | --- | --- | --- | --- | --- | --- | --- | --- | --- |
| 3/4&5 | 1/2 | -6,34524 | 4,704355 | -1,34880 | 0,1774 | -85,2000 | -204,600 | 35,20000 |  |

**Oneway Analysis of original-firstorder-Minimum By GrG <=2 vs >=3**

**Quantiles**

| **Level** | **Minimum** | **10%** | **25%** | **Median** | **75%** | **90%** | **Maximum** |
| --- | --- | --- | --- | --- | --- | --- | --- |
| 1/2 | 0 | 110,2 | 326,75 | 471,5 | 615 | 757,4 | 867 |
| 3/4&5 | 0 | 0 | 237 | 387 | 511 | 662 | 752 |

**Oneway Anova**

**Summary of Fit**

| Rsquare | 0,046036 |
| --- | --- |
| Adj Rsquare | 0,030893 |
| Root Mean Square Error | 221,5955 |
| Mean of Response | 406,5692 |
| Observations (or Sum Wgts) | 65 |

**Pooled t Test**

3/4&5-1/2

Assuming equal variances

| Difference | -96,13 | t Ratio | -1,74362 |
| --- | --- | --- | --- |
| Std Err Dif | 55,13 | DF | 63 |
| Upper CL Dif | 14,04 | Prob > |t| | 0,0861 |
| Lower CL Dif | -206,31 | Prob > t | 0,9569 |
| Confidence | 0,95 | Prob < t | 0,0431* |

**Analysis of Variance**

| **Source** | **DF** | **Sum of Squares** | **Mean Square** | **F Ratio** | **Prob > F** |
| --- | --- | --- | --- | --- | --- |
| GrG <=2 vs >=3 | 1 | 149287,7 | 149288 | 3,0402 | 0,0861 |
| Error | 63 | 3093586,3 | 49105 |  |  |
| C. Total | 64 | 3242873,9 |  |  |  |

**Means for Oneway Anova**

| **Level** | **Number** | **Mean** | **Std Error** | **Lower 95%** | **Upper 95%** |
| --- | --- | --- | --- | --- | --- |
| 1/2 | 30 | 458,333 | 40,458 | 377,49 | 539,18 |
| 3/4&5 | 35 | 362,200 | 37,456 | 287,35 | 437,05 |

Std Error uses a pooled estimate of error variance

**Nonparametric Comparisons For Each Pair Using Wilcoxon Method**

| **q*** | **Alpha** |
| --- | --- |
| 1,95996 | 0,05 |

| **Level** | **- Level** | **Score Mean Difference** | **Std Err Dif** | **Z** | **p-Value** | **Hodges-Lehmann** | **Lower CL** | **Upper CL** | **Difference Plot** |
| --- | --- | --- | --- | --- | --- | --- | --- | --- | --- |
| 3/4&5 | 1/2 | -8,38810 | 4,702349 | -1,78381 | 0,0745 | -97,5000 | -214,000 | 13,00000 |  |

**Oneway Analysis of original-firstorder-Entropy By GrG <=2 vs >=3**

**Quantiles**

| **Level** | **Minimum** | **10%** | **25%** | **Median** | **75%** | **90%** | **Maximum** |
| --- | --- | --- | --- | --- | --- | --- | --- |
| 1/2 | 1,658936 | 3,76114 | 4,345791 | 4,483584 | 4,876727 | 5,067668 | 5,497533 |
| 3/4&5 | 3,679395 | 4,044636 | 4,374921 | 4,633003 | 5,047389 | 5,284099 | 5,585106 |

**Oneway Anova**

**Summary of Fit**

| Rsquare | 0,035845 |
| --- | --- |
| Adj Rsquare | 0,020541 |
| Root Mean Square Error | 0,571559 |
| Mean of Response | 4,57559 |
| Observations (or Sum Wgts) | 65 |

**Pooled t Test**

3/4&5-1/2

Assuming equal variances

| Difference | 0,21764 | t Ratio | 1,530431 |
| --- | --- | --- | --- |
| Std Err Dif | 0,14221 | DF | 63 |
| Upper CL Dif | 0,50182 | Prob > |t| | 0,1309 |
| Lower CL Dif | -0,06654 | Prob > t | 0,0655 |
| Confidence | 0,95 | Prob < t | 0,9345 |

**Analysis of Variance**

| **Source** | **DF** | **Sum of Squares** | **Mean Square** | **F Ratio** | **Prob > F** |
| --- | --- | --- | --- | --- | --- |
| GrG <=2 vs >=3 | 1 | 0,765156 | 0,765156 | 2,3422 | 0,1309 |
| Error | 63 | 20,580835 | 0,326680 |  |  |
| C. Total | 64 | 21,345991 |  |  |  |

**Means for Oneway Anova**

| **Level** | **Number** | **Mean** | **Std Error** | **Lower 95%** | **Upper 95%** |
| --- | --- | --- | --- | --- | --- |
| 1/2 | 30 | 4,45840 | 0,10435 | 4,2499 | 4,6669 |
| 3/4&5 | 35 | 4,67604 | 0,09661 | 4,4830 | 4,8691 |

Std Error uses a pooled estimate of error variance

**Nonparametric Comparisons For Each Pair Using Wilcoxon Method**

| **q*** | **Alpha** |
| --- | --- |
| 1,95996 | 0,05 |

| **Level** | **- Level** | **Score Mean Difference** | **Std Err Dif** | **Z** | **p-Value** | **Hodges-Lehmann** | **Lower CL** | **Upper CL** | **Difference Plot** |
| --- | --- | --- | --- | --- | --- | --- | --- | --- | --- |
| 3/4&5 | 1/2 | 6,345238 | 4,704355 | 1,348801 | 0,1774 | 0,1490726 | -0,089478 | 0,3744823 |  |

**Oneway Analysis of original-firstorder-Range By GrG <=2 vs >=3**

**Quantiles**

| **Level** | **Minimum** | **10%** | **25%** | **Median** | **75%** | **90%** | **Maximum** |
| --- | --- | --- | --- | --- | --- | --- | --- |
| 1/2 | 76 | 541,8 | 719,75 | 920,5 | 1218,75 | 1445,9 | 1702 |
| 3/4&5 | 487 | 618,8 | 747 | 889 | 1321 | 1762 | 2492 |

**Oneway Anova**

**Summary of Fit**

| Rsquare | 0,018152 |
| --- | --- |
| Adj Rsquare | 0,002567 |
| Root Mean Square Error | 410,0269 |
| Mean of Response | 1015,785 |
| Observations (or Sum Wgts) | 65 |

**Pooled t Test**

3/4&5-1/2

Assuming equal variances

| Difference | 110,10 | t Ratio | 1,079227 |
| --- | --- | --- | --- |
| Std Err Dif | 102,02 | DF | 63 |
| Upper CL Dif | 313,97 | Prob > |t| | 0,2846 |
| Lower CL Dif | -93,77 | Prob > t | 0,1423 |
| Confidence | 0,95 | Prob < t | 0,8577 |

**Analysis of Variance**

| **Source** | **DF** | **Sum of Squares** | **Mean Square** | **F Ratio** | **Prob > F** |
| --- | --- | --- | --- | --- | --- |
| GrG <=2 vs >=3 | 1 | 195817 | 195817 | 1,1647 | 0,2846 |
| Error | 63 | 10591692 | 168122 |  |  |
| C. Total | 64 | 10787509 |  |  |  |

**Means for Oneway Anova**

| **Level** | **Number** | **Mean** | **Std Error** | **Lower 95%** | **Upper 95%** |
| --- | --- | --- | --- | --- | --- |
| 1/2 | 30 | 956,50 | 74,860 | 806,90 | 1106,1 |
| 3/4&5 | 35 | 1066,60 | 69,307 | 928,10 | 1205,1 |

Std Error uses a pooled estimate of error variance

**Nonparametric Comparisons For Each Pair Using Wilcoxon Method**

| **q*** | **Alpha** |
| --- | --- |
| 1,95996 | 0,05 |

| **Level** | **- Level** | **Score Mean Difference** | **Std Err Dif** | **Z** | **p-Value** | **Hodges-Lehmann** | **Lower CL** | **Upper CL** | **Difference Plot** |
| --- | --- | --- | --- | --- | --- | --- | --- | --- | --- |
| 3/4&5 | 1/2 | 2,692857 | 4,704303 | 0,5724242 | 0,5670 | 39,50000 | -133,000 | 245,0000 |  |

**Oneway Analysis of original-firstorder-Variance By GrG <=2 vs >=3**

**Quantiles**

| **Level** | **Minimum** | **10%** | **25%** | **Median** | **75%** | **90%** | **Maximum** |
| --- | --- | --- | --- | --- | --- | --- | --- |
| 1/2 | 428,7225 | 17836,72 | 23538,42 | 32309,48 | 45147,98 | 75391,18 | 105212,5 |
| 3/4&5 | 14562,47 | 15639,04 | 22970,9 | 32151,37 | 50536,41 | 78455,38 | 116634,2 |

**Oneway Anova**

**Summary of Fit**

| Rsquare | 0,001814 |
| --- | --- |
| Adj Rsquare | -0,01403 |
| Root Mean Square Error | 23880,36 |
| Mean of Response | 38654,71 |
| Observations (or Sum Wgts) | 65 |

**Pooled t Test**

3/4&5-1/2

Assuming equal variances

| Difference | 2011 | t Ratio | 0,338378 |
| --- | --- | --- | --- |
| Std Err Dif | 5942 | DF | 63 |
| Upper CL Dif | 13884 | Prob > |t| | 0,7362 |
| Lower CL Dif | -9863 | Prob > t | 0,3681 |
| Confidence | 0,95 | Prob < t | 0,6319 |

**Analysis of Variance**

| **Source** | **DF** | **Sum of Squares** | **Mean Square** | **F Ratio** | **Prob > F** |
| --- | --- | --- | --- | --- | --- |
| GrG <=2 vs >=3 | 1 | 65295975,8 | 65295976 | 0,1145 | 0,7362 |
| Error | 63 | 3,5927e+10 | 570271472 |  |  |
| C. Total | 64 | 3,5992e+10 |  |  |  |

**Means for Oneway Anova**

| **Level** | **Number** | **Mean** | **Std Error** | **Lower 95%** | **Upper 95%** |
| --- | --- | --- | --- | --- | --- |
| 1/2 | 30 | 37572,1 | 4359,9 | 28859 | 46285 |
| 3/4&5 | 35 | 39582,6 | 4036,5 | 31516 | 47649 |

Std Error uses a pooled estimate of error variance

**Nonparametric Comparisons For Each Pair Using Wilcoxon Method**

| **q*** | **Alpha** |
| --- | --- |
| 1,95996 | 0,05 |

| **Level** | **- Level** | **Score Mean Difference** | **Std Err Dif** | **Z** | **p-Value** | **Hodges-Lehmann** | **Lower CL** | **Upper CL** | **Difference Plot** |
| --- | --- | --- | --- | --- | --- | --- | --- | --- | --- |
| 3/4&5 | 1/2 | 0,4023810 | 4,704355 | 0,0855337 | 0,9318 | 231,2663 | -8540,64 | 9174,664 |  |

**Oneway Analysis of original-firstorder-10Percentile By GrG <=2 vs >=3**

**Quantiles**

| **Level** | **Minimum** | **10%** | **25%** | **Median** | **75%** | **90%** | **Maximum** |
| --- | --- | --- | --- | --- | --- | --- | --- |
| 1/2 | 1,8 | 405,25 | 616,525 | 743,3 | 821,125 | 934,28 | 1069,6 |
| 3/4&5 | 211,8 | 454,98 | 552,9 | 612 | 702,8 | 837,76 | 880,4 |

**Oneway Anova**

**Summary of Fit**

| Rsquare | 0,031292 |
| --- | --- |
| Adj Rsquare | 0,015916 |
| Root Mean Square Error | 188,6389 |
| Mean of Response | 655,8738 |
| Observations (or Sum Wgts) | 65 |

**Pooled t Test**

3/4&5-1/2

Assuming equal variances

| Difference | -66,96 | t Ratio | -1,42656 |
| --- | --- | --- | --- |
| Std Err Dif | 46,93 | DF | 63 |
| Upper CL Dif | 26,84 | Prob > |t| | 0,1586 |
| Lower CL Dif | -160,75 | Prob > t | 0,9207 |
| Confidence | 0,95 | Prob < t | 0,0793 |

**Analysis of Variance**

| **Source** | **DF** | **Sum of Squares** | **Mean Square** | **F Ratio** | **Prob > F** |
| --- | --- | --- | --- | --- | --- |
| GrG <=2 vs >=3 | 1 | 72417,8 | 72417,8 | 2,0351 | 0,1586 |
| Error | 63 | 2241833,1 | 35584,7 |  |  |
| C. Total | 64 | 2314250,9 |  |  |  |

**Means for Oneway Anova**

| **Level** | **Number** | **Mean** | **Std Error** | **Lower 95%** | **Upper 95%** |
| --- | --- | --- | --- | --- | --- |
| 1/2 | 30 | 691,927 | 34,441 | 623,10 | 760,75 |
| 3/4&5 | 35 | 624,971 | 31,886 | 561,25 | 688,69 |

Std Error uses a pooled estimate of error variance

**Nonparametric Comparisons For Each Pair Using Wilcoxon Method**

| **q*** | **Alpha** |
| --- | --- |
| 1,95996 | 0,05 |

| **Level** | **- Level** | **Score Mean Difference** | **Std Err Dif** | **Z** | **p-Value** | **Hodges-Lehmann** | **Lower CL** | **Upper CL** | **Difference Plot** |
| --- | --- | --- | --- | --- | --- | --- | --- | --- | --- |
| 3/4&5 | 1/2 | -9,81190 | 4,704355 | -2,08571 | 0,0370* | -89,5500 | -172,300 | -7,70000 |  |

**Oneway Analysis of original-firstorder-Kurtosis By GrG <=2 vs >=3**

**Quantiles**

| **Level** | **Minimum** | **10%** | **25%** | **Median** | **75%** | **90%** | **Maximum** |
| --- | --- | --- | --- | --- | --- | --- | --- |
| 1/2 | 1,46581 | 2,151525 | 2,335418 | 2,703902 | 3,568395 | 3,878268 | 4,832345 |
| 3/4&5 | 1,859402 | 2,29162 | 2,596577 | 2,904763 | 3,647208 | 4,153173 | 5,654035 |

**Oneway Anova**

**Summary of Fit**

| Rsquare | 0,016917 |
| --- | --- |
| Adj Rsquare | 0,001312 |
| Root Mean Square Error | 0,777748 |
| Mean of Response | 3,021767 |
| Observations (or Sum Wgts) | 65 |

**Pooled t Test**

3/4&5-1/2

Assuming equal variances

| Difference | 0,20148 | t Ratio | 1,041205 |
| --- | --- | --- | --- |
| Std Err Dif | 0,19351 | DF | 63 |
| Upper CL Dif | 0,58818 | Prob > |t| | 0,3018 |
| Lower CL Dif | -0,18521 | Prob > t | 0,1509 |
| Confidence | 0,95 | Prob < t | 0,8491 |

**Analysis of Variance**

| **Source** | **DF** | **Sum of Squares** | **Mean Square** | **F Ratio** | **Prob > F** |
| --- | --- | --- | --- | --- | --- |
| GrG <=2 vs >=3 | 1 | 0,655768 | 0,655768 | 1,0841 | 0,3018 |
| Error | 63 | 38,108202 | 0,604892 |  |  |
| C. Total | 64 | 38,763970 |  |  |  |

**Means for Oneway Anova**

| **Level** | **Number** | **Mean** | **Std Error** | **Lower 95%** | **Upper 95%** |
| --- | --- | --- | --- | --- | --- |
| 1/2 | 30 | 2,91328 | 0,14200 | 2,6295 | 3,1970 |
| 3/4&5 | 35 | 3,11476 | 0,13146 | 2,8520 | 3,3775 |

Std Error uses a pooled estimate of error variance

**Nonparametric Comparisons For Each Pair Using Wilcoxon Method**

| **q*** | **Alpha** |
| --- | --- |
| 1,95996 | 0,05 |

| **Level** | **- Level** | **Score Mean Difference** | **Std Err Dif** | **Z** | **p-Value** | **Hodges-Lehmann** | **Lower CL** | **Upper CL** | **Difference Plot** |
| --- | --- | --- | --- | --- | --- | --- | --- | --- | --- |
| 3/4&5 | 1/2 | 5,045238 | 4,704355 | 1,072461 | 0,2835 | 0,1988317 | -0,135430 | 0,5134747 |  |

**Oneway Analysis of original-firstorder-Mean By GrG <=2 vs >=3**

**Quantiles**

| **Level** | **Minimum** | **10%** | **25%** | **Median** | **75%** | **90%** | **Maximum** |
| --- | --- | --- | --- | --- | --- | --- | --- |
| 1/2 | 160,8511 | 595,2349 | 817,5238 | 972,872 | 1082,251 | 1211,656 | 1345,697 |
| 3/4&5 | 513,191 | 655,0052 | 763,2892 | 864,9769 | 1006,721 | 1059,029 | 1100,615 |

**Oneway Anova**

**Summary of Fit**

| Rsquare | 0,029368 |
| --- | --- |
| Adj Rsquare | 0,013962 |
| Root Mean Square Error | 200,3208 |
| Mean of Response | 891,9884 |
| Observations (or Sum Wgts) | 65 |

**Pooled t Test**

3/4&5-1/2

Assuming equal variances

| Difference | -68,81 | t Ratio | -1,38065 |
| --- | --- | --- | --- |
| Std Err Dif | 49,84 | DF | 63 |
| Upper CL Dif | 30,79 | Prob > |t| | 0,1723 |
| Lower CL Dif | -168,41 | Prob > t | 0,9139 |
| Confidence | 0,95 | Prob < t | 0,0861 |

**Analysis of Variance**

| **Source** | **DF** | **Sum of Squares** | **Mean Square** | **F Ratio** | **Prob > F** |
| --- | --- | --- | --- | --- | --- |
| GrG <=2 vs >=3 | 1 | 76492,5 | 76492,5 | 1,9062 | 0,1723 |
| Error | 63 | 2528091,0 | 40128,4 |  |  |
| C. Total | 64 | 2604583,5 |  |  |  |

**Means for Oneway Anova**

| **Level** | **Number** | **Mean** | **Std Error** | **Lower 95%** | **Upper 95%** |
| --- | --- | --- | --- | --- | --- |
| 1/2 | 30 | 929,042 | 36,573 | 855,96 | 1002,1 |
| 3/4&5 | 35 | 860,228 | 33,860 | 792,56 | 927,9 |

Std Error uses a pooled estimate of error variance

**Nonparametric Comparisons For Each Pair Using Wilcoxon Method**

| **q*** | **Alpha** |
| --- | --- |
| 1,95996 | 0,05 |

| **Level** | **- Level** | **Score Mean Difference** | **Std Err Dif** | **Z** | **p-Value** | **Hodges-Lehmann** | **Lower CL** | **Upper CL** | **Difference Plot** |
| --- | --- | --- | --- | --- | --- | --- | --- | --- | --- |
| 3/4&5 | 1/2 | -8,51190 | 4,704355 | -1,80937 | 0,0704 | -89,6163 | -180,523 | 8,681588 |  |

**Oneway Analysis of original-glrlm-ShortRunLowGrayLevelEmphasis By GrG <=2 vs >=3**

**Quantiles**

| **Level** | **Minimum** | **10%** | **25%** | **Median** | **75%** | **90%** | **Maximum** |
| --- | --- | --- | --- | --- | --- | --- | --- |
| 1/2 | 0,001993 | 0,00236 | 0,008848 | 0,015421 | 0,034337 | 0,07001 | 0,399814 |
| 3/4&5 | 0,000964 | 0,002742 | 0,005788 | 0,012997 | 0,020437 | 0,043457 | 0,071207 |

**Oneway Anova**

**Summary of Fit**

| Rsquare | 0,031979 |
| --- | --- |
| Adj Rsquare | 0,016614 |
| Root Mean Square Error | 0,050887 |
| Mean of Response | 0,025569 |
| Observations (or Sum Wgts) | 65 |

**Pooled t Test**

3/4&5-1/2

Assuming equal variances

| Difference | -0,01827 | t Ratio | -1,44265 |
| --- | --- | --- | --- |
| Std Err Dif | 0,01266 | DF | 63 |
| Upper CL Dif | 0,00704 | Prob > |t| | 0,1541 |
| Lower CL Dif | -0,04357 | Prob > t | 0,9230 |
| Confidence | 0,95 | Prob < t | 0,0770 |

**Analysis of Variance**

| **Source** | **DF** | **Sum of Squares** | **Mean Square** | **F Ratio** | **Prob > F** |
| --- | --- | --- | --- | --- | --- |
| GrG <=2 vs >=3 | 1 | 0,00538924 | 0,005389 | 2,0812 | 0,1541 |
| Error | 63 | 0,16313492 | 0,002589 |  |  |
| C. Total | 64 | 0,16852416 |  |  |  |

**Means for Oneway Anova**

| **Level** | **Number** | **Mean** | **Std Error** | **Lower 95%** | **Upper 95%** |
| --- | --- | --- | --- | --- | --- |
| 1/2 | 30 | 0,035405 | 0,00929 | 0,0168 | 0,05397 |
| 3/4&5 | 35 | 0,017139 | 0,00860 | -4,9e-5 | 0,03433 |

Std Error uses a pooled estimate of error variance

**Nonparametric Comparisons For Each Pair Using Wilcoxon Method**

| **q*** | **Alpha** |
| --- | --- |
| 1,95996 | 0,05 |

| **Level** | **- Level** | **Score Mean Difference** | **Std Err Dif** | **Z** | **p-Value** | **Hodges-Lehmann** | **Lower CL** | **Upper CL** | **Difference Plot** |
| --- | --- | --- | --- | --- | --- | --- | --- | --- | --- |
| 3/4&5 | 1/2 | -5,10714 | 4,704355 | -1,08562 | 0,2776 | -0,003526 | -0,010291 | 0,0021683 |  |

**Oneway Analysis of original-glrlm-GrayLevelVariance By GrG <=2 vs >=3**

**Quantiles**

| **Level** | **Minimum** | **10%** | **25%** | **Median** | **75%** | **90%** | **Maximum** |
| --- | --- | --- | --- | --- | --- | --- | --- |
| 1/2 | 0,661157 | 29,52889 | 37,79968 | 52,27359 | 72,77887 | 120,9439 | 169,6237 |
| 3/4&5 | 23,34965 | 25,24014 | 37,62642 | 51,4546 | 82,00434 | 124,1908 | 187,5598 |

**Oneway Anova**

**Summary of Fit**

| Rsquare | 0,001986 |
| --- | --- |
| Adj Rsquare | -0,01386 |
| Root Mean Square Error | 38,12806 |
| Mean of Response | 62,29591 |
| Observations (or Sum Wgts) | 65 |

**Pooled t Test**

3/4&5-1/2

Assuming equal variances

| Difference | 3,359 | t Ratio | 0,354068 |
| --- | --- | --- | --- |
| Std Err Dif | 9,487 | DF | 63 |
| Upper CL Dif | 22,316 | Prob > |t| | 0,7245 |
| Lower CL Dif | -15,598 | Prob > t | 0,3622 |
| Confidence | 0,95 | Prob < t | 0,6378 |

**Analysis of Variance**

| **Source** | **DF** | **Sum of Squares** | **Mean Square** | **F Ratio** | **Prob > F** |
| --- | --- | --- | --- | --- | --- |
| GrG <=2 vs >=3 | 1 | 182,248 | 182,25 | 0,1254 | 0,7245 |
| Error | 63 | 91586,182 | 1453,75 |  |  |
| C. Total | 64 | 91768,430 |  |  |  |

**Means for Oneway Anova**

| **Level** | **Number** | **Mean** | **Std Error** | **Lower 95%** | **Upper 95%** |
| --- | --- | --- | --- | --- | --- |
| 1/2 | 30 | 60,4873 | 6,9612 | 46,576 | 74,398 |
| 3/4&5 | 35 | 63,8462 | 6,4448 | 50,967 | 76,725 |

Std Error uses a pooled estimate of error variance

**Nonparametric Comparisons For Each Pair Using Wilcoxon Method**

| **q*** | **Alpha** |
| --- | --- |
| 1,95996 | 0,05 |

| **Level** | **- Level** | **Score Mean Difference** | **Std Err Dif** | **Z** | **p-Value** | **Hodges-Lehmann** | **Lower CL** | **Upper CL** | **Difference Plot** |
| --- | --- | --- | --- | --- | --- | --- | --- | --- | --- |
| 3/4&5 | 1/2 | 0,2785714 | 4,704355 | 0,0592157 | 0,9528 | 0,3904106 | -13,3857 | 15,00639 |  |

**Oneway Analysis of original-glrlm-LowGrayLevelRunEmphasis By GrG <=2 vs >=3**

**Quantiles**

| **Level** | **Minimum** | **10%** | **25%** | **Median** | **75%** | **90%** | **Maximum** |
| --- | --- | --- | --- | --- | --- | --- | --- |
| 1/2 | 0,002042 | 0,002414 | 0,008904 | 0,015594 | 0,034825 | 0,073649 | 0,46662 |
| 3/4&5 | 0,00099 | 0,002802 | 0,005819 | 0,013205 | 0,020467 | 0,044195 | 0,072734 |

**Oneway Anova**

**Summary of Fit**

| Rsquare | 0,031169 |
| --- | --- |
| Adj Rsquare | 0,015791 |
| Root Mean Square Error | 0,058829 |
| Mean of Response | 0,027003 |
| Observations (or Sum Wgts) | 65 |

**Pooled t Test**

3/4&5-1/2

Assuming equal variances

| Difference | -0,02084 | t Ratio | -1,42367 |
| --- | --- | --- | --- |
| Std Err Dif | 0,01464 | DF | 63 |
| Upper CL Dif | 0,00841 | Prob > |t| | 0,1595 |
| Lower CL Dif | -0,05009 | Prob > t | 0,9203 |
| Confidence | 0,95 | Prob < t | 0,0797 |

**Analysis of Variance**

| **Source** | **DF** | **Sum of Squares** | **Mean Square** | **F Ratio** | **Prob > F** |
| --- | --- | --- | --- | --- | --- |
| GrG <=2 vs >=3 | 1 | 0,00701444 | 0,007014 | 2,0268 | 0,1595 |
| Error | 63 | 0,21803023 | 0,003461 |  |  |
| C. Total | 64 | 0,22504467 |  |  |  |

**Means for Oneway Anova**

| **Level** | **Number** | **Mean** | **Std Error** | **Lower 95%** | **Upper 95%** |
| --- | --- | --- | --- | --- | --- |
| 1/2 | 30 | 0,038223 | 0,01074 | 0,0168 | 0,05969 |
| 3/4&5 | 35 | 0,017385 | 0,00994 | -0,0025 | 0,03726 |

Std Error uses a pooled estimate of error variance

**Nonparametric Comparisons For Each Pair Using Wilcoxon Method**

| **q*** | **Alpha** |
| --- | --- |
| 1,95996 | 0,05 |

| **Level** | **- Level** | **Score Mean Difference** | **Std Err Dif** | **Z** | **p-Value** | **Hodges-Lehmann** | **Lower CL** | **Upper CL** | **Difference Plot** |
| --- | --- | --- | --- | --- | --- | --- | --- | --- | --- |
| 3/4&5 | 1/2 | -5,04524 | 4,704355 | -1,07246 | 0,2835 | -0,003559 | -0,010352 | 0,0022761 |  |

**Oneway Analysis of original-glrlm-GrayLevelNonUniformityNormalized By GrG <=2 vs >=3**

**Quantiles**

| **Level** | **Minimum** | **10%** | **25%** | **Median** | **75%** | **90%** | **Maximum** |
| --- | --- | --- | --- | --- | --- | --- | --- |
| 1/2 | 0,024548 | 0,035524 | 0,039989 | 0,051368 | 0,056959 | 0,092941 | 0,336305 |
| 3/4&5 | 0,023779 | 0,029603 | 0,035316 | 0,045178 | 0,055853 | 0,068447 | 0,093269 |

**Oneway Anova**

**Summary of Fit**

| Rsquare | 0,030266 |
| --- | --- |
| Adj Rsquare | 0,014874 |
| Root Mean Square Error | 0,038892 |
| Mean of Response | 0,05416 |
| Observations (or Sum Wgts) | 65 |

**Pooled t Test**

3/4&5-1/2

Assuming equal variances

| Difference | -0,01357 | t Ratio | -1,40224 |
| --- | --- | --- | --- |
| Std Err Dif | 0,00968 | DF | 63 |
| Upper CL Dif | 0,00577 | Prob > |t| | 0,1658 |
| Lower CL Dif | -0,03291 | Prob > t | 0,9171 |
| Confidence | 0,95 | Prob < t | 0,0829 |

**Analysis of Variance**

| **Source** | **DF** | **Sum of Squares** | **Mean Square** | **F Ratio** | **Prob > F** |
| --- | --- | --- | --- | --- | --- |
| GrG <=2 vs >=3 | 1 | 0,00297422 | 0,002974 | 1,9663 | 0,1658 |
| Error | 63 | 0,09529464 | 0,001513 |  |  |
| C. Total | 64 | 0,09826887 |  |  |  |

**Means for Oneway Anova**

| **Level** | **Number** | **Mean** | **Std Error** | **Lower 95%** | **Upper 95%** |
| --- | --- | --- | --- | --- | --- |
| 1/2 | 30 | 0,061467 | 0,00710 | 0,04728 | 0,07566 |
| 3/4&5 | 35 | 0,047898 | 0,00657 | 0,03476 | 0,06103 |

Std Error uses a pooled estimate of error variance

**Nonparametric Comparisons For Each Pair Using Wilcoxon Method**

| **q*** | **Alpha** |
| --- | --- |
| 1,95996 | 0,05 |

| **Level** | **- Level** | **Score Mean Difference** | **Std Err Dif** | **Z** | **p-Value** | **Hodges-Lehmann** | **Lower CL** | **Upper CL** | **Difference Plot** |
| --- | --- | --- | --- | --- | --- | --- | --- | --- | --- |
| 3/4&5 | 1/2 | -6,53095 | 4,704355 | -1,38828 | 0,1651 | -0,004669 | -0,011846 | 0,0024793 |  |

**Oneway Analysis of original-glrlm-RunVariance By GrG <=2 vs >=3**

**Quantiles**

| **Level** | **Minimum** | **10%** | **25%** | **Median** | **75%** | **90%** | **Maximum** |
| --- | --- | --- | --- | --- | --- | --- | --- |
| 1/2 | 0,020107 | 0,022847 | 0,027364 | 0,039671 | 0,046446 | 0,050982 | 0,262458 |
| 3/4&5 | 0,02322 | 0,027257 | 0,031327 | 0,041306 | 0,045802 | 0,051875 | 0,058952 |

**Oneway Anova**

**Summary of Fit**

| Rsquare | 0,007603 |
| --- | --- |
| Adj Rsquare | -0,00815 |
| Root Mean Square Error | 0,029578 |
| Mean of Response | 0,041728 |
| Observations (or Sum Wgts) | 65 |

**Pooled t Test**

3/4&5-1/2

Assuming equal variances

| Difference | -0,00511 | t Ratio | -0,69475 |
| --- | --- | --- | --- |
| Std Err Dif | 0,00736 | DF | 63 |
| Upper CL Dif | 0,00959 | Prob > |t| | 0,4898 |
| Lower CL Dif | -0,01982 | Prob > t | 0,7551 |
| Confidence | 0,95 | Prob < t | 0,2449 |

**Analysis of Variance**

| **Source** | **DF** | **Sum of Squares** | **Mean Square** | **F Ratio** | **Prob > F** |
| --- | --- | --- | --- | --- | --- |
| GrG <=2 vs >=3 | 1 | 0,00042227 | 0,000422 | 0,4827 | 0,4898 |
| Error | 63 | 0,05511610 | 0,000875 |  |  |
| C. Total | 64 | 0,05553838 |  |  |  |

**Means for Oneway Anova**

| **Level** | **Number** | **Mean** | **Std Error** | **Lower 95%** | **Upper 95%** |
| --- | --- | --- | --- | --- | --- |
| 1/2 | 30 | 0,044481 | 0,00540 | 0,03369 | 0,05527 |
| 3/4&5 | 35 | 0,039368 | 0,00500 | 0,02938 | 0,04936 |

Std Error uses a pooled estimate of error variance

**Nonparametric Comparisons For Each Pair Using Wilcoxon Method**

| **q*** | **Alpha** |
| --- | --- |
| 1,95996 | 0,05 |

| **Level** | **- Level** | **Score Mean Difference** | **Std Err Dif** | **Z** | **p-Value** | **Hodges-Lehmann** | **Lower CL** | **Upper CL** | **Difference Plot** |
| --- | --- | --- | --- | --- | --- | --- | --- | --- | --- |
| 3/4&5 | 1/2 | 3,559524 | 4,704355 | 0,7566444 | 0,4493 | 0,0018127 | -0,003764 | 0,0069151 |  |

**Oneway Analysis of original-glrlm-GrayLevelNonUniformity By GrG <=2 vs >=3**

**Quantiles**

| **Level** | **Minimum** | **10%** | **25%** | **Median** | **75%** | **90%** | **Maximum** |
| --- | --- | --- | --- | --- | --- | --- | --- |
| 1/2 | 2,781333 | 3,332635 | 4,583501 | 5,680547 | 13,25408 | 45,99752 | 98,60145 |
| 3/4&5 | 2,390432 | 3,463968 | 6,511811 | 10,55953 | 21,62198 | 100,3209 | 405,8616 |

**Oneway Anova**

**Summary of Fit**

| Rsquare | 0,032573 |
| --- | --- |
| Adj Rsquare | 0,017217 |
| Root Mean Square Error | 54,75528 |
| Mean of Response | 25,01228 |
| Observations (or Sum Wgts) | 65 |

**Pooled t Test**

3/4&5-1/2

Assuming equal variances

| Difference | 19,842 | t Ratio | 1,456436 |
| --- | --- | --- | --- |
| Std Err Dif | 13,623 | DF | 63 |
| Upper CL Dif | 47,066 | Prob > |t| | 0,1502 |
| Lower CL Dif | -7,383 | Prob > t | 0,0751 |
| Confidence | 0,95 | Prob < t | 0,9249 |

**Analysis of Variance**

| **Source** | **DF** | **Sum of Squares** | **Mean Square** | **F Ratio** | **Prob > F** |
| --- | --- | --- | --- | --- | --- |
| GrG <=2 vs >=3 | 1 | 6359,67 | 6359,67 | 2,1212 | 0,1502 |
| Error | 63 | 188882,84 | 2998,14 |  |  |
| C. Total | 64 | 195242,51 |  |  |  |

**Means for Oneway Anova**

| **Level** | **Number** | **Mean** | **Std Error** | **Lower 95%** | **Upper 95%** |
| --- | --- | --- | --- | --- | --- |
| 1/2 | 30 | 14,3283 | 9,9969 | -5,65 | 34,305 |
| 3/4&5 | 35 | 34,1700 | 9,2553 | 15,67 | 52,665 |

Std Error uses a pooled estimate of error variance

**Nonparametric Comparisons For Each Pair Using Wilcoxon Method**

| **q*** | **Alpha** |
| --- | --- |
| 1,95996 | 0,05 |

| **Level** | **- Level** | **Score Mean Difference** | **Std Err Dif** | **Z** | **p-Value** | **Hodges-Lehmann** | **Lower CL** | **Upper CL** | **Difference Plot** |
| --- | --- | --- | --- | --- | --- | --- | --- | --- | --- |
| 3/4&5 | 1/2 | 9,069048 | 4,704355 | 1,927798 | 0,0539 | 3,145874 | -0,125125 | 8,206315 |  |

**Oneway Analysis of original-glrlm-LongRunEmphasis By GrG <=2 vs >=3**

**Quantiles**

| **Level** | **Minimum** | **10%** | **25%** | **Median** | **75%** | **90%** | **Maximum** |
| --- | --- | --- | --- | --- | --- | --- | --- |
| 1/2 | 1,05747 | 1,06994 | 1,078293 | 1,117875 | 1,140645 | 1,15174 | 1,936815 |
| 3/4&5 | 1,07357 | 1,082425 | 1,096335 | 1,126986 | 1,136335 | 1,151407 | 1,170041 |

**Oneway Anova**

**Summary of Fit**

| Rsquare | 0,009025 |
| --- | --- |
| Adj Rsquare | -0,00671 |
| Root Mean Square Error | 0,106233 |
| Mean of Response | 1,126528 |
| Observations (or Sum Wgts) | 65 |

**Pooled t Test**

3/4&5-1/2

Assuming equal variances

| Difference | -0,02002 | t Ratio | -0,75745 |
| --- | --- | --- | --- |
| Std Err Dif | 0,02643 | DF | 63 |
| Upper CL Dif | 0,03280 | Prob > |t| | 0,4516 |
| Lower CL Dif | -0,07284 | Prob > t | 0,7742 |
| Confidence | 0,95 | Prob < t | 0,2258 |

**Analysis of Variance**

| **Source** | **DF** | **Sum of Squares** | **Mean Square** | **F Ratio** | **Prob > F** |
| --- | --- | --- | --- | --- | --- |
| GrG <=2 vs >=3 | 1 | 0,00647479 | 0,006475 | 0,5737 | 0,4516 |
| Error | 63 | 0,71098876 | 0,011286 |  |  |
| C. Total | 64 | 0,71746355 |  |  |  |

**Means for Oneway Anova**

| **Level** | **Number** | **Mean** | **Std Error** | **Lower 95%** | **Upper 95%** |
| --- | --- | --- | --- | --- | --- |
| 1/2 | 30 | 1,13731 | 0,01940 | 1,0985 | 1,1761 |
| 3/4&5 | 35 | 1,11729 | 0,01796 | 1,0814 | 1,1532 |

Std Error uses a pooled estimate of error variance

**Nonparametric Comparisons For Each Pair Using Wilcoxon Method**

| **q*** | **Alpha** |
| --- | --- |
| 1,95996 | 0,05 |

| **Level** | **- Level** | **Score Mean Difference** | **Std Err Dif** | **Z** | **p-Value** | **Hodges-Lehmann** | **Lower CL** | **Upper CL** | **Difference Plot** |
| --- | --- | --- | --- | --- | --- | --- | --- | --- | --- |
| 3/4&5 | 1/2 | 3,559524 | 4,704355 | 0,7566444 | 0,4493 | 0,0059832 | -0,011291 | 0,0204999 |  |

**Oneway Analysis of original-glrlm-ShortRunHighGrayLevelEmphasis By GrG <=2 vs >=3**

**Quantiles**

| **Level** | **Minimum** | **10%** | **25%** | **Median** | **75%** | **90%** | **Maximum** |
| --- | --- | --- | --- | --- | --- | --- | --- |
| 1/2 | 3,600202 | 160,8091 | 222,7725 | 439,2094 | 738,3797 | 990,9489 | 1098,802 |
| 3/4&5 | 141,9518 | 196,6619 | 260,9281 | 382,1881 | 683,8233 | 1136,629 | 2018,638 |

**Oneway Anova**

**Summary of Fit**

| Rsquare | 0,004249 |
| --- | --- |
| Adj Rsquare | -0,01156 |
| Root Mean Square Error | 356,8545 |
| Mean of Response | 514,931 |
| Observations (or Sum Wgts) | 65 |

**Pooled t Test**

3/4&5-1/2

Assuming equal variances

| Difference | 46,04 | t Ratio | 0,518485 |
| --- | --- | --- | --- |
| Std Err Dif | 88,79 | DF | 63 |
| Upper CL Dif | 223,46 | Prob > |t| | 0,6059 |
| Lower CL Dif | -131,39 | Prob > t | 0,3030 |
| Confidence | 0,95 | Prob < t | 0,6970 |

**Analysis of Variance**

| **Source** | **DF** | **Sum of Squares** | **Mean Square** | **F Ratio** | **Prob > F** |
| --- | --- | --- | --- | --- | --- |
| GrG <=2 vs >=3 | 1 | 34233,7 | 34234 | 0,2688 | 0,6059 |
| Error | 63 | 8022741,9 | 127345 |  |  |
| C. Total | 64 | 8056975,6 |  |  |  |

**Means for Oneway Anova**

| **Level** | **Number** | **Mean** | **Std Error** | **Lower 95%** | **Upper 95%** |
| --- | --- | --- | --- | --- | --- |
| 1/2 | 30 | 490,143 | 65,152 | 359,95 | 620,34 |
| 3/4&5 | 35 | 536,178 | 60,319 | 415,64 | 656,72 |

Std Error uses a pooled estimate of error variance

**Nonparametric Comparisons For Each Pair Using Wilcoxon Method**

| **q*** | **Alpha** |
| --- | --- |
| 1,95996 | 0,05 |

| **Level** | **- Level** | **Score Mean Difference** | **Std Err Dif** | **Z** | **p-Value** | **Hodges-Lehmann** | **Lower CL** | **Upper CL** | **Difference Plot** |
| --- | --- | --- | --- | --- | --- | --- | --- | --- | --- |
| 3/4&5 | 1/2 | 0,8357143 | 4,704355 | 0,1776470 | 0,8590 | 19,51436 | -135,999 | 150,9821 |  |

**Oneway Analysis of original-glrlm-RunLengthNonUniformity By GrG <=2 vs >=3**

**Quantiles**

| **Level** | **Minimum** | **10%** | **25%** | **Median** | **75%** | **90%** | **Maximum** |
| --- | --- | --- | --- | --- | --- | --- | --- |
| 1/2 | 26,86072 | 32,23425 | 67,31522 | 119,6673 | 261,6525 | 1224,214 | 2537,388 |
| 3/4&5 | 25,81212 | 60,20982 | 93,95591 | 271,589 | 413,4958 | 2667,763 | 14563,78 |

**Oneway Anova**

**Summary of Fit**

| Rsquare | 0,027982 |
| --- | --- |
| Adj Rsquare | 0,012553 |
| Root Mean Square Error | 1870,956 |
| Mean of Response | 650,1946 |
| Observations (or Sum Wgts) | 65 |

**Pooled t Test**

3/4&5-1/2

Assuming equal variances

| Difference | 626,9 | t Ratio | 1,346702 |
| --- | --- | --- | --- |
| Std Err Dif | 465,5 | DF | 63 |
| Upper CL Dif | 1557,1 | Prob > |t| | 0,1829 |
| Lower CL Dif | -303,3 | Prob > t | 0,0915 |
| Confidence | 0,95 | Prob < t | 0,9085 |

**Analysis of Variance**

| **Source** | **DF** | **Sum of Squares** | **Mean Square** | **F Ratio** | **Prob > F** |
| --- | --- | --- | --- | --- | --- |
| GrG <=2 vs >=3 | 1 | 6348489 | 6348489 | 1,8136 | 0,1829 |
| Error | 63 | 220529962 | 3500476 |  |  |
| C. Total | 64 | 226878451 |  |  |  |

**Means for Oneway Anova**

| **Level** | **Number** | **Mean** | **Std Error** | **Lower 95%** | **Upper 95%** |
| --- | --- | --- | --- | --- | --- |
| 1/2 | 30 | 312,634 | 341,59 | -370,0 | 995,2 |
| 3/4&5 | 35 | 939,532 | 316,25 | 307,6 | 1571,5 |

Std Error uses a pooled estimate of error variance

**Nonparametric Comparisons For Each Pair Using Wilcoxon Method**

| **q*** | **Alpha** |
| --- | --- |
| 1,95996 | 0,05 |

| **Level** | **- Level** | **Score Mean Difference** | **Std Err Dif** | **Z** | **p-Value** | **Hodges-Lehmann** | **Lower CL** | **Upper CL** | **Difference Plot** |
| --- | --- | --- | --- | --- | --- | --- | --- | --- | --- |
| 3/4&5 | 1/2 | 10,55476 | 4,704355 | 2,243615 | 0,0249* | 101,4276 | 9,235119 | 213,1138 |  |

**Oneway Analysis of original-glrlm-ShortRunEmphasis By GrG <=2 vs >=3**

**Quantiles**

| **Level** | **Minimum** | **10%** | **25%** | **Median** | **75%** | **90%** | **Maximum** |
| --- | --- | --- | --- | --- | --- | --- | --- |
| 1/2 | 0,827472 | 0,964473 | 0,967626 | 0,972615 | 0,980427 | 0,984447 | 0,987491 |
| 3/4&5 | 0,961811 | 0,965452 | 0,967981 | 0,971703 | 0,977744 | 0,980465 | 0,982617 |

**Oneway Anova**

**Summary of Fit**

| Rsquare | 0,006304 |
| --- | --- |
| Adj Rsquare | -0,00947 |
| Root Mean Square Error | 0,019284 |
| Mean of Response | 0,971018 |
| Observations (or Sum Wgts) | 65 |

**Pooled t Test**

3/4&5-1/2

Assuming equal variances

| Difference | 0,00303 | t Ratio | 0,632172 |
| --- | --- | --- | --- |
| Std Err Dif | 0,00480 | DF | 63 |
| Upper CL Dif | 0,01262 | Prob > |t| | 0,5296 |
| Lower CL Dif | -0,00655 | Prob > t | 0,2648 |
| Confidence | 0,95 | Prob < t | 0,7352 |

**Analysis of Variance**

| **Source** | **DF** | **Sum of Squares** | **Mean Square** | **F Ratio** | **Prob > F** |
| --- | --- | --- | --- | --- | --- |
| GrG <=2 vs >=3 | 1 | 0,00014861 | 0,000149 | 0,3996 | 0,5296 |
| Error | 63 | 0,02342766 | 0,000372 |  |  |
| C. Total | 64 | 0,02357628 |  |  |  |

**Means for Oneway Anova**

| **Level** | **Number** | **Mean** | **Std Error** | **Lower 95%** | **Upper 95%** |
| --- | --- | --- | --- | --- | --- |
| 1/2 | 30 | 0,969385 | 0,00352 | 0,96235 | 0,97642 |
| 3/4&5 | 35 | 0,972418 | 0,00326 | 0,96590 | 0,97893 |

Std Error uses a pooled estimate of error variance

**Nonparametric Comparisons For Each Pair Using Wilcoxon Method**

| **q*** | **Alpha** |
| --- | --- |
| 1,95996 | 0,05 |

| **Level** | **- Level** | **Score Mean Difference** | **Std Err Dif** | **Z** | **p-Value** | **Hodges-Lehmann** | **Lower CL** | **Upper CL** | **Difference Plot** |
| --- | --- | --- | --- | --- | --- | --- | --- | --- | --- |
| 3/4&5 | 1/2 | -3,49762 | 4,704355 | -0,743485 | 0,4572 | -0,001309 | -0,004757 | 0,0024394 |  |

**Oneway Analysis of original-glrlm-LongRunHighGrayLevelEmphasis By GrG <=2 vs >=3**

**Quantiles**

| **Level** | **Minimum** | **10%** | **25%** | **Median** | **75%** | **90%** | **Maximum** |
| --- | --- | --- | --- | --- | --- | --- | --- |
| 1/2 | 8,631241 | 186,0696 | 256,0944 | 496,0213 | 861,1906 | 1174,753 | 1193,606 |
| 3/4&5 | 164,6487 | 229,022 | 304,9891 | 427,0072 | 781,0171 | 1319,531 | 2335,755 |

**Oneway Anova**

**Summary of Fit**

| Rsquare | 0,004905 |
| --- | --- |
| Adj Rsquare | -0,01089 |
| Root Mean Square Error | 407,4914 |
| Mean of Response | 585,0367 |
| Observations (or Sum Wgts) | 65 |

**Pooled t Test**

3/4&5-1/2

Assuming equal variances

| Difference | 56,50 | t Ratio | 0,557276 |
| --- | --- | --- | --- |
| Std Err Dif | 101,39 | DF | 63 |
| Upper CL Dif | 259,11 | Prob > |t| | 0,5793 |
| Lower CL Dif | -146,10 | Prob > t | 0,2897 |
| Confidence | 0,95 | Prob < t | 0,7103 |

**Analysis of Variance**

| **Source** | **DF** | **Sum of Squares** | **Mean Square** | **F Ratio** | **Prob > F** |
| --- | --- | --- | --- | --- | --- |
| GrG <=2 vs >=3 | 1 | 51568 | 51568 | 0,3106 | 0,5793 |
| Error | 63 | 10461104 | 166049 |  |  |
| C. Total | 64 | 10512672 |  |  |  |

**Means for Oneway Anova**

| **Level** | **Number** | **Mean** | **Std Error** | **Lower 95%** | **Upper 95%** |
| --- | --- | --- | --- | --- | --- |
| 1/2 | 30 | 554,613 | 74,397 | 405,94 | 703,28 |
| 3/4&5 | 35 | 611,114 | 68,879 | 473,47 | 748,76 |

Std Error uses a pooled estimate of error variance

**Nonparametric Comparisons For Each Pair Using Wilcoxon Method**

| **q*** | **Alpha** |
| --- | --- |
| 1,95996 | 0,05 |

| **Level** | **- Level** | **Score Mean Difference** | **Std Err Dif** | **Z** | **p-Value** | **Hodges-Lehmann** | **Lower CL** | **Upper CL** | **Difference Plot** |
| --- | --- | --- | --- | --- | --- | --- | --- | --- | --- |
| 3/4&5 | 1/2 | 1,269048 | 4,704355 | 0,2697602 | 0,7873 | 25,75355 | -143,689 | 169,3893 |  |

**Oneway Analysis of original-glrlm-RunPercentage By GrG <=2 vs >=3**

**Quantiles**

| **Level** | **Minimum** | **10%** | **25%** | **Median** | **75%** | **90%** | **Maximum** |
| --- | --- | --- | --- | --- | --- | --- | --- |
| 1/2 | 0,810147 | 0,95329 | 0,956739 | 0,963977 | 0,97476 | 0,978482 | 0,982732 |
| 3/4&5 | 0,948928 | 0,954029 | 0,957666 | 0,96337 | 0,970441 | 0,974043 | 0,97683 |

**Oneway Anova**

**Summary of Fit**

| Rsquare | 0,004598 |
| --- | --- |
| Adj Rsquare | -0,0112 |
| Root Mean Square Error | 0,021064 |
| Mean of Response | 0,962445 |
| Observations (or Sum Wgts) | 65 |

**Pooled t Test**

3/4&5-1/2

Assuming equal variances

| Difference | 0,00283 | t Ratio | 0,539467 |
| --- | --- | --- | --- |
| Std Err Dif | 0,00524 | DF | 63 |
| Upper CL Dif | 0,01330 | Prob > |t| | 0,5915 |
| Lower CL Dif | -0,00765 | Prob > t | 0,2957 |
| Confidence | 0,95 | Prob < t | 0,7043 |

**Analysis of Variance**

| **Source** | **DF** | **Sum of Squares** | **Mean Square** | **F Ratio** | **Prob > F** |
| --- | --- | --- | --- | --- | --- |
| GrG <=2 vs >=3 | 1 | 0,00012913 | 0,000129 | 0,2910 | 0,5915 |
| Error | 63 | 0,02795346 | 0,000444 |  |  |
| C. Total | 64 | 0,02808259 |  |  |  |

**Means for Oneway Anova**

| **Level** | **Number** | **Mean** | **Std Error** | **Lower 95%** | **Upper 95%** |
| --- | --- | --- | --- | --- | --- |
| 1/2 | 30 | 0,960922 | 0,00385 | 0,95324 | 0,96861 |
| 3/4&5 | 35 | 0,963750 | 0,00356 | 0,95663 | 0,97086 |

Std Error uses a pooled estimate of error variance

**Nonparametric Comparisons For Each Pair Using Wilcoxon Method**

| **q*** | **Alpha** |
| --- | --- |
| 1,95996 | 0,05 |

| **Level** | **- Level** | **Score Mean Difference** | **Std Err Dif** | **Z** | **p-Value** | **Hodges-Lehmann** | **Lower CL** | **Upper CL** | **Difference Plot** |
| --- | --- | --- | --- | --- | --- | --- | --- | --- | --- |
| 3/4&5 | 1/2 | -3,55952 | 4,704252 | -0,756661 | 0,4493 | -0,001823 | -0,006326 | 0,0031460 |  |

**Oneway Analysis of original-glrlm-LongRunLowGrayLevelEmphasis By GrG <=2 vs >=3**

**Quantiles**

| **Level** | **Minimum** | **10%** | **25%** | **Median** | **75%** | **90%** | **Maximum** |
| --- | --- | --- | --- | --- | --- | --- | --- |
| 1/2 | 0,002248 | 0,002659 | 0,009147 | 0,01631 | 0,036423 | 0,088201 | 0,856265 |
| 3/4&5 | 0,001105 | 0,003059 | 0,005949 | 0,014049 | 0,020764 | 0,04719 | 0,078846 |

**Oneway Anova**

**Summary of Fit**

| Rsquare | 0,02795 |
| --- | --- |
| Adj Rsquare | 0,012521 |
| Root Mean Square Error | 0,105969 |
| Mean of Response | 0,034769 |
| Observations (or Sum Wgts) | 65 |

**Pooled t Test**

3/4&5-1/2

Assuming equal variances

| Difference | -0,03549 | t Ratio | -1,34592 |
| --- | --- | --- | --- |
| Std Err Dif | 0,02637 | DF | 63 |
| Upper CL Dif | 0,01720 | Prob > |t| | 0,1832 |
| Lower CL Dif | -0,08817 | Prob > t | 0,9084 |
| Confidence | 0,95 | Prob < t | 0,0916 |

**Analysis of Variance**

| **Source** | **DF** | **Sum of Squares** | **Mean Square** | **F Ratio** | **Prob > F** |
| --- | --- | --- | --- | --- | --- |
| GrG <=2 vs >=3 | 1 | 0,02034241 | 0,020342 | 1,8115 | 0,1832 |
| Error | 63 | 0,70745950 | 0,011230 |  |  |
| C. Total | 64 | 0,72780191 |  |  |  |

**Means for Oneway Anova**

| **Level** | **Number** | **Mean** | **Std Error** | **Lower 95%** | **Upper 95%** |
| --- | --- | --- | --- | --- | --- |
| 1/2 | 30 | 0,053877 | 0,01935 | 0,0152 | 0,09254 |
| 3/4&5 | 35 | 0,018391 | 0,01791 | -0,0174 | 0,05419 |

Std Error uses a pooled estimate of error variance

**Nonparametric Comparisons For Each Pair Using Wilcoxon Method**

| **q*** | **Alpha** |
| --- | --- |
| 1,95996 | 0,05 |

| **Level** | **- Level** | **Score Mean Difference** | **Std Err Dif** | **Z** | **p-Value** | **Hodges-Lehmann** | **Lower CL** | **Upper CL** | **Difference Plot** |
| --- | --- | --- | --- | --- | --- | --- | --- | --- | --- |
| 3/4&5 | 1/2 | -4,61190 | 4,704355 | -0,980348 | 0,3269 | -0,003494 | -0,010806 | 0,0024107 |  |

**Oneway Analysis of original-glrlm-RunEntropy By GrG <=2 vs >=3**

**Quantiles**

| **Level** | **Minimum** | **10%** | **25%** | **Median** | **75%** | **90%** | **Maximum** |
| --- | --- | --- | --- | --- | --- | --- | --- |
| 1/2 | 2,390519 | 3,852115 | 4,442574 | 4,642763 | 5,019057 | 5,30925 | 5,570541 |
| 3/4&5 | 3,770475 | 4,167929 | 4,555723 | 4,804145 | 5,18721 | 5,442697 | 5,843222 |

**Oneway Anova**

**Summary of Fit**

| Rsquare | 0,040684 |
| --- | --- |
| Adj Rsquare | 0,025457 |
| Root Mean Square Error | 0,550237 |
| Mean of Response | 4,744569 |
| Observations (or Sum Wgts) | 65 |

**Pooled t Test**

3/4&5-1/2

Assuming equal variances

| Difference | 0,22378 | t Ratio | 1,63456 |
| --- | --- | --- | --- |
| Std Err Dif | 0,13690 | DF | 63 |
| Upper CL Dif | 0,49735 | Prob > |t| | 0,1071 |
| Lower CL Dif | -0,04980 | Prob > t | 0,0536 |
| Confidence | 0,95 | Prob < t | 0,9464 |

**Analysis of Variance**

| **Source** | **DF** | **Sum of Squares** | **Mean Square** | **F Ratio** | **Prob > F** |
| --- | --- | --- | --- | --- | --- |
| GrG <=2 vs >=3 | 1 | 0,808912 | 0,808912 | 2,6718 | 0,1071 |
| Error | 63 | 19,073923 | 0,302761 |  |  |
| C. Total | 64 | 19,882835 |  |  |  |

**Means for Oneway Anova**

| **Level** | **Number** | **Mean** | **Std Error** | **Lower 95%** | **Upper 95%** |
| --- | --- | --- | --- | --- | --- |
| 1/2 | 30 | 4,62407 | 0,10046 | 4,4233 | 4,8248 |
| 3/4&5 | 35 | 4,84785 | 0,09301 | 4,6620 | 5,0337 |

Std Error uses a pooled estimate of error variance

**Nonparametric Comparisons For Each Pair Using Wilcoxon Method**

| **q*** | **Alpha** |
| --- | --- |
| 1,95996 | 0,05 |

| **Level** | **- Level** | **Score Mean Difference** | **Std Err Dif** | **Z** | **p-Value** | **Hodges-Lehmann** | **Lower CL** | **Upper CL** | **Difference Plot** |
| --- | --- | --- | --- | --- | --- | --- | --- | --- | --- |
| 3/4&5 | 1/2 | 7,088095 | 4,704355 | 1,506709 | 0,1319 | 0,1689472 | -0,077288 | 0,4093370 |  |

**Oneway Analysis of original-glrlm-HighGrayLevelRunEmphasis By GrG <=2 vs >=3**

**Quantiles**

| **Level** | **Minimum** | **10%** | **25%** | **Median** | **75%** | **90%** | **Maximum** |
| --- | --- | --- | --- | --- | --- | --- | --- |
| 1/2 | 4,424969 | 165,2875 | 229,0211 | 449,9455 | 761,6256 | 1026,721 | 1116,089 |
| 3/4&5 | 146,3938 | 202,4934 | 269,4176 | 390,1049 | 699,2614 | 1171,682 | 2077,596 |

**Oneway Anova**

**Summary of Fit**

| Rsquare | 0,004376 |
| --- | --- |
| Adj Rsquare | -0,01143 |
| Root Mean Square Error | 366,273 |
| Mean of Response | 528,2183 |
| Observations (or Sum Wgts) | 65 |

**Pooled t Test**

3/4&5-1/2

Assuming equal variances

| Difference | 47,96 | t Ratio | 0,526232 |
| --- | --- | --- | --- |
| Std Err Dif | 91,13 | DF | 63 |
| Upper CL Dif | 230,07 | Prob > |t| | 0,6006 |
| Lower CL Dif | -134,16 | Prob > t | 0,3003 |
| Confidence | 0,95 | Prob < t | 0,6997 |

**Analysis of Variance**

| **Source** | **DF** | **Sum of Squares** | **Mean Square** | **F Ratio** | **Prob > F** |
| --- | --- | --- | --- | --- | --- |
| GrG <=2 vs >=3 | 1 | 37150,4 | 37150 | 0,2769 | 0,6006 |
| Error | 63 | 8451824,5 | 134156 |  |  |
| C. Total | 64 | 8488975,0 |  |  |  |

**Means for Oneway Anova**

| **Level** | **Number** | **Mean** | **Std Error** | **Lower 95%** | **Upper 95%** |
| --- | --- | --- | --- | --- | --- |
| 1/2 | 30 | 502,396 | 66,872 | 368,76 | 636,03 |
| 3/4&5 | 35 | 550,352 | 61,911 | 426,63 | 674,07 |

Std Error uses a pooled estimate of error variance

**Nonparametric Comparisons For Each Pair Using Wilcoxon Method**

| **q*** | **Alpha** |
| --- | --- |
| 1,95996 | 0,05 |

| **Level** | **- Level** | **Score Mean Difference** | **Std Err Dif** | **Z** | **p-Value** | **Hodges-Lehmann** | **Lower CL** | **Upper CL** | **Difference Plot** |
| --- | --- | --- | --- | --- | --- | --- | --- | --- | --- |
| 3/4&5 | 1/2 | 0,8976190 | 4,704355 | 0,1908060 | 0,8487 | 19,85664 | -137,527 | 154,8980 |  |

**Oneway Analysis of original-glrlm-RunLengthNonUniformityNormalized By GrG <=2 vs >=3**

**Quantiles**

| **Level** | **Minimum** | **10%** | **25%** | **Median** | **75%** | **90%** | **Maximum** |
| --- | --- | --- | --- | --- | --- | --- | --- |
| 1/2 | 0,678678 | 0,91044 | 0,918369 | 0,931851 | 0,949584 | 0,959729 | 0,968448 |
| 3/4&5 | 0,90445 | 0,913214 | 0,919005 | 0,92824 | 0,942887 | 0,950438 | 0,95545 |

**Oneway Anova**

**Summary of Fit**

| Rsquare | 0,002944 |
| --- | --- |
| Adj Rsquare | -0,01288 |
| Root Mean Square Error | 0,035511 |
| Mean of Response | 0,928469 |
| Observations (or Sum Wgts) | 65 |

**Pooled t Test**

3/4&5-1/2

Assuming equal variances

| Difference | 0,00381 | t Ratio | 0,431285 |
| --- | --- | --- | --- |
| Std Err Dif | 0,00884 | DF | 63 |
| Upper CL Dif | 0,02147 | Prob > |t| | 0,6677 |
| Lower CL Dif | -0,01385 | Prob > t | 0,3339 |
| Confidence | 0,95 | Prob < t | 0,6661 |

**Analysis of Variance**

| **Source** | **DF** | **Sum of Squares** | **Mean Square** | **F Ratio** | **Prob > F** |
| --- | --- | --- | --- | --- | --- |
| GrG <=2 vs >=3 | 1 | 0,00023456 | 0,000235 | 0,1860 | 0,6677 |
| Error | 63 | 0,07944480 | 0,001261 |  |  |
| C. Total | 64 | 0,07967936 |  |  |  |

**Means for Oneway Anova**

| **Level** | **Number** | **Mean** | **Std Error** | **Lower 95%** | **Upper 95%** |
| --- | --- | --- | --- | --- | --- |
| 1/2 | 30 | 0,926417 | 0,00648 | 0,91346 | 0,93937 |
| 3/4&5 | 35 | 0,930228 | 0,00600 | 0,91823 | 0,94222 |

Std Error uses a pooled estimate of error variance

**Nonparametric Comparisons For Each Pair Using Wilcoxon Method**

| **q*** | **Alpha** |
| --- | --- |
| 1,95996 | 0,05 |

| **Level** | **- Level** | **Score Mean Difference** | **Std Err Dif** | **Z** | **p-Value** | **Hodges-Lehmann** | **Lower CL** | **Upper CL** | **Difference Plot** |
| --- | --- | --- | --- | --- | --- | --- | --- | --- | --- |
| 3/4&5 | 1/2 | -3,43571 | 4,704355 | -0,730326 | 0,4652 | -0,003179 | -0,012039 | 0,0058152 |  |

**Oneway Analysis of original-glszm-GrayLevelVariance By GrG <=2 vs >=3**

**Quantiles**

| **Level** | **Minimum** | **10%** | **25%** | **Median** | **75%** | **90%** | **Maximum** |
| --- | --- | --- | --- | --- | --- | --- | --- |
| 1/2 | 1,25 | 32,16629 | 41,39192 | 60,19984 | 83,96772 | 127,1653 | 179,7537 |
| 3/4&5 | 23,72222 | 29,90453 | 43,48722 | 56,74345 | 93,88075 | 127,8624 | 193,0242 |

**Oneway Anova**

**Summary of Fit**

| Rsquare | 0,00425 |
| --- | --- |
| Adj Rsquare | -0,01156 |
| Root Mean Square Error | 39,39538 |
| Mean of Response | 69,27837 |
| Observations (or Sum Wgts) | 65 |

**Pooled t Test**

3/4&5-1/2

Assuming equal variances

| Difference | 5,083 | t Ratio | 0,518574 |
| --- | --- | --- | --- |
| Std Err Dif | 9,802 | DF | 63 |
| Upper CL Dif | 24,670 | Prob > |t| | 0,6059 |
| Lower CL Dif | -14,504 | Prob > t | 0,3029 |
| Confidence | 0,95 | Prob < t | 0,6971 |

**Analysis of Variance**

| **Source** | **DF** | **Sum of Squares** | **Mean Square** | **F Ratio** | **Prob > F** |
| --- | --- | --- | --- | --- | --- |
| GrG <=2 vs >=3 | 1 | 417,361 | 417,36 | 0,2689 | 0,6059 |
| Error | 63 | 97775,766 | 1552,00 |  |  |
| C. Total | 64 | 98193,127 |  |  |  |

**Means for Oneway Anova**

| **Level** | **Number** | **Mean** | **Std Error** | **Lower 95%** | **Upper 95%** |
| --- | --- | --- | --- | --- | --- |
| 1/2 | 30 | 66,5414 | 7,1926 | 52,168 | 80,915 |
| 3/4&5 | 35 | 71,6244 | 6,6590 | 58,317 | 84,931 |

Std Error uses a pooled estimate of error variance

**Nonparametric Comparisons For Each Pair Using Wilcoxon Method**

| **q*** | **Alpha** |
| --- | --- |
| 1,95996 | 0,05 |

| **Level** | **- Level** | **Score Mean Difference** | **Std Err Dif** | **Z** | **p-Value** | **Hodges-Lehmann** | **Lower CL** | **Upper CL** | **Difference Plot** |
| --- | --- | --- | --- | --- | --- | --- | --- | --- | --- |
| 3/4&5 | 1/2 | 1,578571 | 4,704355 | 0,3355554 | 0,7372 | 2,584934 | -12,4588 | 18,37958 |  |

**Oneway Analysis of original-glszm-ZoneVariance By GrG <=2 vs >=3**

**Quantiles**

| **Level** | **Minimum** | **10%** | **25%** | **Median** | **75%** | **90%** | **Maximum** |
| --- | --- | --- | --- | --- | --- | --- | --- |
| 1/2 | 0,34451 | 0,482375 | 0,760095 | 1,476535 | 2,523826 | 3,555214 | 48,6875 |
| 3/4&5 | 0,548701 | 0,710667 | 1,165764 | 1,526042 | 2,738184 | 4,36852 | 6,727899 |

**Oneway Anova**

**Summary of Fit**

| Rsquare | 0,00825 |
| --- | --- |
| Adj Rsquare | -0,00749 |
| Root Mean Square Error | 5,989351 |
| Mean of Response | 2,674269 |
| Observations (or Sum Wgts) | 65 |

**Pooled t Test**

3/4&5-1/2

Assuming equal variances

| Difference | -1,0788 | t Ratio | -0,72394 |
| --- | --- | --- | --- |
| Std Err Dif | 1,4902 | DF | 63 |
| Upper CL Dif | 1,8991 | Prob > |t| | 0,4718 |
| Lower CL Dif | -4,0567 | Prob > t | 0,7641 |
| Confidence | 0,95 | Prob < t | 0,2359 |

**Analysis of Variance**

| **Source** | **DF** | **Sum of Squares** | **Mean Square** | **F Ratio** | **Prob > F** |
| --- | --- | --- | --- | --- | --- |
| GrG <=2 vs >=3 | 1 | 18,8001 | 18,8001 | 0,5241 | 0,4718 |
| Error | 63 | 2259,9562 | 35,8723 |  |  |
| C. Total | 64 | 2278,7563 |  |  |  |

**Means for Oneway Anova**

| **Level** | **Number** | **Mean** | **Std Error** | **Lower 95%** | **Upper 95%** |
| --- | --- | --- | --- | --- | --- |
| 1/2 | 30 | 3,25516 | 1,0935 | 1,0700 | 5,4403 |
| 3/4&5 | 35 | 2,17636 | 1,0124 | 0,1533 | 4,1994 |

Std Error uses a pooled estimate of error variance

**Nonparametric Comparisons For Each Pair Using Wilcoxon Method**

| **q*** | **Alpha** |
| --- | --- |
| 1,95996 | 0,05 |

| **Level** | **- Level** | **Score Mean Difference** | **Std Err Dif** | **Z** | **p-Value** | **Hodges-Lehmann** | **Lower CL** | **Upper CL** | **Difference Plot** |
| --- | --- | --- | --- | --- | --- | --- | --- | --- | --- |
| 3/4&5 | 1/2 | 5,045238 | 4,704355 | 1,072461 | 0,2835 | 0,2682880 | -0,232779 | 0,8444588 |  |

**Oneway Analysis of original-glszm-GrayLevelNonUniformityNormalized By GrG <=2 vs >=3**

**Quantiles**

| **Level** | **Minimum** | **10%** | **25%** | **Median** | **75%** | **90%** | **Maximum** |
| --- | --- | --- | --- | --- | --- | --- | --- |
| 1/2 | 0,023951 | 0,031831 | 0,036129 | 0,047487 | 0,052684 | 0,070568 | 0,25 |
| 3/4&5 | 0,022073 | 0,02735 | 0,032023 | 0,041724 | 0,050556 | 0,064386 | 0,08 |

**Oneway Anova**

**Summary of Fit**

| Rsquare | 0,031114 |
| --- | --- |
| Adj Rsquare | 0,015735 |
| Root Mean Square Error | 0,028537 |
| Mean of Response | 0,047589 |
| Observations (or Sum Wgts) | 65 |

**Pooled t Test**

3/4&5-1/2

Assuming equal variances

| Difference | -0,01010 | t Ratio | -1,42236 |
| --- | --- | --- | --- |
| Std Err Dif | 0,00710 | DF | 63 |
| Upper CL Dif | 0,00409 | Prob > |t| | 0,1599 |
| Lower CL Dif | -0,02429 | Prob > t | 0,9201 |
| Confidence | 0,95 | Prob < t | 0,0799 |

**Analysis of Variance**

| **Source** | **DF** | **Sum of Squares** | **Mean Square** | **F Ratio** | **Prob > F** |
| --- | --- | --- | --- | --- | --- |
| GrG <=2 vs >=3 | 1 | 0,00164759 | 0,001648 | 2,0231 | 0,1599 |
| Error | 63 | 0,05130587 | 0,000814 |  |  |
| C. Total | 64 | 0,05295346 |  |  |  |

**Means for Oneway Anova**

| **Level** | **Number** | **Mean** | **Std Error** | **Lower 95%** | **Upper 95%** |
| --- | --- | --- | --- | --- | --- |
| 1/2 | 30 | 0,053027 | 0,00521 | 0,04261 | 0,06344 |
| 3/4&5 | 35 | 0,042928 | 0,00482 | 0,03329 | 0,05257 |

Std Error uses a pooled estimate of error variance

**Nonparametric Comparisons For Each Pair Using Wilcoxon Method**

| **q*** | **Alpha** |
| --- | --- |
| 1,95996 | 0,05 |

| **Level** | **- Level** | **Score Mean Difference** | **Std Err Dif** | **Z** | **p-Value** | **Hodges-Lehmann** | **Lower CL** | **Upper CL** | **Difference Plot** |
| --- | --- | --- | --- | --- | --- | --- | --- | --- | --- |
| 3/4&5 | 1/2 | -6,40714 | 4,704303 | -1,36197 | 0,1732 | -0,004066 | -0,010482 | 0,0022126 |  |

**Oneway Analysis of original-glszm-SizeZoneNonUniformityNormalized By GrG <=2 vs >=3**

**Quantiles**

| **Level** | **Minimum** | **10%** | **25%** | **Median** | **75%** | **90%** | **Maximum** |
| --- | --- | --- | --- | --- | --- | --- | --- |
| 1/2 | 0,25 | 0,393519 | 0,443914 | 0,493918 | 0,58933 | 0,662931 | 0,693762 |
| 3/4&5 | 0,407407 | 0,437326 | 0,46875 | 0,496191 | 0,552549 | 0,581385 | 0,69375 |

**Oneway Anova**

**Summary of Fit**

| Rsquare | 0,000116 |
| --- | --- |
| Adj Rsquare | -0,01576 |
| Root Mean Square Error | 0,082831 |
| Mean of Response | 0,511462 |
| Observations (or Sum Wgts) | 65 |

**Pooled t Test**

3/4&5-1/2

Assuming equal variances

| Difference | -0,00176 | t Ratio | -0,08532 |
| --- | --- | --- | --- |
| Std Err Dif | 0,02061 | DF | 63 |
| Upper CL Dif | 0,03943 | Prob > |t| | 0,9323 |
| Lower CL Dif | -0,04294 | Prob > t | 0,5339 |
| Confidence | 0,95 | Prob < t | 0,4661 |

**Analysis of Variance**

| **Source** | **DF** | **Sum of Squares** | **Mean Square** | **F Ratio** | **Prob > F** |
| --- | --- | --- | --- | --- | --- |
| GrG <=2 vs >=3 | 1 | 0,00004995 | 0,000050 | 0,0073 | 0,9323 |
| Error | 63 | 0,43223777 | 0,006861 |  |  |
| C. Total | 64 | 0,43228771 |  |  |  |

**Means for Oneway Anova**

| **Level** | **Number** | **Mean** | **Std Error** | **Lower 95%** | **Upper 95%** |
| --- | --- | --- | --- | --- | --- |
| 1/2 | 30 | 0,512408 | 0,01512 | 0,48219 | 0,54263 |
| 3/4&5 | 35 | 0,510650 | 0,01400 | 0,48267 | 0,53863 |

Std Error uses a pooled estimate of error variance

**Nonparametric Comparisons For Each Pair Using Wilcoxon Method**

| **q*** | **Alpha** |
| --- | --- |
| 1,95996 | 0,05 |

| **Level** | **- Level** | **Score Mean Difference** | **Std Err Dif** | **Z** | **p-Value** | **Hodges-Lehmann** | **Lower CL** | **Upper CL** | **Difference Plot** |
| --- | --- | --- | --- | --- | --- | --- | --- | --- | --- |
| 3/4&5 | 1/2 | 0,9595238 | 4,704355 | 0,2039650 | 0,8384 | 0,0060958 | -0,036966 | 0,0402708 |  |

**Oneway Analysis of original-glszm-SizeZoneNonUniformity By GrG <=2 vs >=3**

**Quantiles**

| **Level** | **Minimum** | **10%** | **25%** | **Median** | **75%** | **90%** | **Maximum** |
| --- | --- | --- | --- | --- | --- | --- | --- |
| 1/2 | 1 | 16,46087 | 28,06423 | 44,75905 | 93,08556 | 320,1199 | 703,8592 |
| 3/4&5 | 11,19048 | 23,1 | 40,52113 | 94,29897 | 150,1906 | 747,7366 | 4397,37 |

**Oneway Anova**

**Summary of Fit**

| Rsquare | 0,029098 |
| --- | --- |
| Adj Rsquare | 0,013687 |
| Root Mean Square Error | 559,3636 |
| Mean of Response | 198,3159 |
| Observations (or Sum Wgts) | 65 |

**Pooled t Test**

3/4&5-1/2

Assuming equal variances

| Difference | 191,24 | t Ratio | 1,374094 |
| --- | --- | --- | --- |
| Std Err Dif | 139,17 | DF | 63 |
| Upper CL Dif | 469,35 | Prob > |t| | 0,1743 |
| Lower CL Dif | -86,88 | Prob > t | 0,0871 |
| Confidence | 0,95 | Prob < t | 0,9129 |

**Analysis of Variance**

| **Source** | **DF** | **Sum of Squares** | **Mean Square** | **F Ratio** | **Prob > F** |
| --- | --- | --- | --- | --- | --- |
| GrG <=2 vs >=3 | 1 | 590774 | 590774 | 1,8881 | 0,1743 |
| Error | 63 | 19711921 | 312888 |  |  |
| C. Total | 64 | 20302695 |  |  |  |

**Means for Oneway Anova**

| **Level** | **Number** | **Mean** | **Std Error** | **Lower 95%** | **Upper 95%** |
| --- | --- | --- | --- | --- | --- |
| 1/2 | 30 | 95,342 | 102,13 | -108,7 | 299,42 |
| 3/4&5 | 35 | 286,579 | 94,55 | 97,6 | 475,52 |

Std Error uses a pooled estimate of error variance

**Nonparametric Comparisons For Each Pair Using Wilcoxon Method**

| **q*** | **Alpha** |
| --- | --- |
| 1,95996 | 0,05 |

| **Level** | **- Level** | **Score Mean Difference** | **Std Err Dif** | **Z** | **p-Value** | **Hodges-Lehmann** | **Lower CL** | **Upper CL** | **Difference Plot** |
| --- | --- | --- | --- | --- | --- | --- | --- | --- | --- |
| 3/4&5 | 1/2 | 10,55476 | 4,704355 | 2,243615 | 0,0249* | 29,90181 | 3,577078 | 71,90946 |  |

**Oneway Analysis of original-glszm-GrayLevelNonUniformity By GrG <=2 vs >=3**

**Quantiles**

| **Level** | **Minimum** | **10%** | **25%** | **Median** | **75%** | **90%** | **Maximum** |
| --- | --- | --- | --- | --- | --- | --- | --- |
| 1/2 | 1 | 1,665775 | 2,685759 | 3,550322 | 7,01799 | 24,19195 | 48,78424 |
| 3/4&5 | 1,47619 | 2,21322 | 3,62963 | 6,269036 | 11,7191 | 50,99838 | 199,0071 |

**Oneway Anova**

**Summary of Fit**

| Rsquare | 0,034997 |
| --- | --- |
| Adj Rsquare | 0,019679 |
| Root Mean Square Error | 26,64821 |
| Mean of Response | 12,81191 |
| Observations (or Sum Wgts) | 65 |

**Pooled t Test**

3/4&5-1/2

Assuming equal variances

| Difference | 10,022 | t Ratio | 1,511536 |
| --- | --- | --- | --- |
| Std Err Dif | 6,630 | DF | 63 |
| Upper CL Dif | 23,271 | Prob > |t| | 0,1357 |
| Lower CL Dif | -3,228 | Prob > t | 0,0678 |
| Confidence | 0,95 | Prob < t | 0,9322 |

**Analysis of Variance**

| **Source** | **DF** | **Sum of Squares** | **Mean Square** | **F Ratio** | **Prob > F** |
| --- | --- | --- | --- | --- | --- |
| GrG <=2 vs >=3 | 1 | 1622,456 | 1622,46 | 2,2847 | 0,1357 |
| Error | 63 | 44738,010 | 710,13 |  |  |
| C. Total | 64 | 46360,466 |  |  |  |

**Means for Oneway Anova**

| **Level** | **Number** | **Mean** | **Std Error** | **Lower 95%** | **Upper 95%** |
| --- | --- | --- | --- | --- | --- |
| 1/2 | 30 | 7,4155 | 4,8653 | -2,307 | 17,138 |
| 3/4&5 | 35 | 17,4374 | 4,5044 | 8,436 | 26,439 |

Std Error uses a pooled estimate of error variance

**Nonparametric Comparisons For Each Pair Using Wilcoxon Method**

| **q*** | **Alpha** |
| --- | --- |
| 1,95996 | 0,05 |

| **Level** | **- Level** | **Score Mean Difference** | **Std Err Dif** | **Z** | **p-Value** | **Hodges-Lehmann** | **Lower CL** | **Upper CL** | **Difference Plot** |
| --- | --- | --- | --- | --- | --- | --- | --- | --- | --- |
| 3/4&5 | 1/2 | 10,86429 | 4,704355 | 2,309410 | 0,0209* | 2,233699 | 0,3125472 | 4,457971 |  |

**Oneway Analysis of original-glszm-LargeAreaEmphasis By GrG <=2 vs >=3**

**Quantiles**

| **Level** | **Minimum** | **10%** | **25%** | **Median** | **75%** | **90%** | **Maximum** |
| --- | --- | --- | --- | --- | --- | --- | --- |
| 1/2 | 1,923077 | 2,411578 | 2,811896 | 4,250269 | 5,642677 | 7,104606 | 186,75 |
| 3/4&5 | 2,483333 | 2,818983 | 3,159091 | 4,611111 | 6,20339 | 8,096623 | 10,8723 |

**Oneway Anova**

**Summary of Fit**

| Rsquare | 0,014516 |
| --- | --- |
| Adj Rsquare | -0,00113 |
| Root Mean Square Error | 22,68417 |
| Mean of Response | 7,474941 |
| Observations (or Sum Wgts) | 65 |

**Pooled t Test**

3/4&5-1/2

Assuming equal variances

| Difference | -5,437 | t Ratio | -0,9633 |
| --- | --- | --- | --- |
| Std Err Dif | 5,644 | DF | 63 |
| Upper CL Dif | 5,842 | Prob > |t| | 0,3391 |
| Lower CL Dif | -16,715 | Prob > t | 0,8305 |
| Confidence | 0,95 | Prob < t | 0,1695 |

**Analysis of Variance**

| **Source** | **DF** | **Sum of Squares** | **Mean Square** | **F Ratio** | **Prob > F** |
| --- | --- | --- | --- | --- | --- |
| GrG <=2 vs >=3 | 1 | 477,499 | 477,499 | 0,9280 | 0,3391 |
| Error | 63 | 32417,996 | 514,571 |  |  |
| C. Total | 64 | 32895,495 |  |  |  |

**Means for Oneway Anova**

| **Level** | **Number** | **Mean** | **Std Error** | **Lower 95%** | **Upper 95%** |
| --- | --- | --- | --- | --- | --- |
| 1/2 | 30 | 10,4025 | 4,1415 | 2,126 | 18,679 |
| 3/4&5 | 35 | 4,9656 | 3,8343 | -2,697 | 12,628 |

Std Error uses a pooled estimate of error variance

**Nonparametric Comparisons For Each Pair Using Wilcoxon Method**

| **q*** | **Alpha** |
| --- | --- |
| 1,95996 | 0,05 |

| **Level** | **- Level** | **Score Mean Difference** | **Std Err Dif** | **Z** | **p-Value** | **Hodges-Lehmann** | **Lower CL** | **Upper CL** | **Difference Plot** |
| --- | --- | --- | --- | --- | --- | --- | --- | --- | --- |
| 3/4&5 | 1/2 | 5,230952 | 4,704355 | 1,111938 | 0,2662 | 0,4443355 | -0,516291 | 1,357999 |  |

**Oneway Analysis of original-glszm-SmallAreaHighGrayLevelEmphasis By GrG <=2 vs >=3**

**Quantiles**

| **Level** | **Minimum** | **10%** | **25%** | **Median** | **75%** | **90%** | **Maximum** |
| --- | --- | --- | --- | --- | --- | --- | --- |
| 1/2 | 4,022206 | 123,5687 | 177,4246 | 307,0168 | 625,923 | 754,3457 | 934,0037 |
| 3/4&5 | 87,79946 | 134,6812 | 228,107 | 315,9198 | 574,1325 | 892,3029 | 1766,947 |

**Oneway Anova**

**Summary of Fit**

| Rsquare | 0,005233 |
| --- | --- |
| Adj Rsquare | -0,01056 |
| Root Mean Square Error | 302,2225 |
| Mean of Response | 412,3295 |
| Observations (or Sum Wgts) | 65 |

**Pooled t Test**

3/4&5-1/2

Assuming equal variances

| Difference | 43,29 | t Ratio | 0,575677 |
| --- | --- | --- | --- |
| Std Err Dif | 75,19 | DF | 63 |
| Upper CL Dif | 193,55 | Prob > |t| | 0,5669 |
| Lower CL Dif | -106,98 | Prob > t | 0,2834 |
| Confidence | 0,95 | Prob < t | 0,7166 |

**Analysis of Variance**

| **Source** | **DF** | **Sum of Squares** | **Mean Square** | **F Ratio** | **Prob > F** |
| --- | --- | --- | --- | --- | --- |
| GrG <=2 vs >=3 | 1 | 30269,9 | 30269,9 | 0,3314 | 0,5669 |
| Error | 63 | 5754321,8 | 91338,4 |  |  |
| C. Total | 64 | 5784591,7 |  |  |  |

**Means for Oneway Anova**

| **Level** | **Number** | **Mean** | **Std Error** | **Lower 95%** | **Upper 95%** |
| --- | --- | --- | --- | --- | --- |
| 1/2 | 30 | 389,021 | 55,178 | 278,76 | 499,29 |
| 3/4&5 | 35 | 432,309 | 51,085 | 330,22 | 534,39 |

Std Error uses a pooled estimate of error variance

**Nonparametric Comparisons For Each Pair Using Wilcoxon Method**

| **q*** | **Alpha** |
| --- | --- |
| 1,95996 | 0,05 |

| **Level** | **- Level** | **Score Mean Difference** | **Std Err Dif** | **Z** | **p-Value** | **Hodges-Lehmann** | **Lower CL** | **Upper CL** | **Difference Plot** |
| --- | --- | --- | --- | --- | --- | --- | --- | --- | --- |
| 3/4&5 | 1/2 | 1,269048 | 4,704355 | 0,2697602 | 0,7873 | 12,49822 | -101,078 | 114,6692 |  |

**Oneway Analysis of original-glszm-ZonePercentage By GrG <=2 vs >=3**

**Quantiles**

| **Level** | **Minimum** | **10%** | **25%** | **Median** | **75%** | **90%** | **Maximum** |
| --- | --- | --- | --- | --- | --- | --- | --- |
| 1/2 | 0,085106 | 0,519569 | 0,558293 | 0,612184 | 0,710495 | 0,75173 | 0,795918 |
| 3/4&5 | 0,484375 | 0,528937 | 0,556127 | 0,609524 | 0,668639 | 0,714286 | 0,722892 |

**Oneway Anova**

**Summary of Fit**

| Rsquare | 0,000275 |
| --- | --- |
| Adj Rsquare | -0,01559 |
| Root Mean Square Error | 0,10127 |
| Mean of Response | 0,611712 |
| Observations (or Sum Wgts) | 65 |

**Pooled t Test**

3/4&5-1/2

Assuming equal variances

| Difference | -0,00332 | t Ratio | -0,13157 |
| --- | --- | --- | --- |
| Std Err Dif | 0,02520 | DF | 63 |
| Upper CL Dif | 0,04704 | Prob > |t| | 0,8957 |
| Lower CL Dif | -0,05367 | Prob > t | 0,5521 |
| Confidence | 0,95 | Prob < t | 0,4479 |

**Analysis of Variance**

| **Source** | **DF** | **Sum of Squares** | **Mean Square** | **F Ratio** | **Prob > F** |
| --- | --- | --- | --- | --- | --- |
| GrG <=2 vs >=3 | 1 | 0,00017752 | 0,000178 | 0,0173 | 0,8957 |
| Error | 63 | 0,64610414 | 0,010256 |  |  |
| C. Total | 64 | 0,64628167 |  |  |  |

**Means for Oneway Anova**

| **Level** | **Number** | **Mean** | **Std Error** | **Lower 95%** | **Upper 95%** |
| --- | --- | --- | --- | --- | --- |
| 1/2 | 30 | 0,613497 | 0,01849 | 0,57655 | 0,65044 |
| 3/4&5 | 35 | 0,610182 | 0,01712 | 0,57597 | 0,64439 |

Std Error uses a pooled estimate of error variance

**Nonparametric Comparisons For Each Pair Using Wilcoxon Method**

| **q*** | **Alpha** |
| --- | --- |
| 1,95996 | 0,05 |

| **Level** | **- Level** | **Score Mean Difference** | **Std Err Dif** | **Z** | **p-Value** | **Hodges-Lehmann** | **Lower CL** | **Upper CL** | **Difference Plot** |
| --- | --- | --- | --- | --- | --- | --- | --- | --- | --- |
| 3/4&5 | 1/2 | -3,55952 | 4,704252 | -0,756661 | 0,4493 | -0,014216 | -0,053486 | 0,0249653 |  |

**Oneway Analysis of original-glszm-LargeAreaLowGrayLevelEmphasis By GrG <=2 vs >=3**

**Quantiles**

| **Level** | **Minimum** | **10%** | **25%** | **Median** | **75%** | **90%** | **Maximum** |
| --- | --- | --- | --- | --- | --- | --- | --- |
| 1/2 | 0,01035 | 0,012361 | 0,01848 | 0,030812 | 0,087497 | 0,278222 | 84,62674 |
| 3/4&5 | 0,00856 | 0,009502 | 0,017701 | 0,035849 | 0,072183 | 0,105264 | 0,189797 |

**Oneway Anova**

**Summary of Fit**

| Rsquare | 0,01918 |
| --- | --- |
| Adj Rsquare | 0,003611 |
| Root Mean Square Error | 10,47061 |
| Mean of Response | 1,382615 |
| Observations (or Sum Wgts) | 65 |

**Pooled t Test**

3/4&5-1/2

Assuming equal variances

| Difference | -2,8916 | t Ratio | -1,10994 |
| --- | --- | --- | --- |
| Std Err Dif | 2,6052 | DF | 63 |
| Upper CL Dif | 2,3144 | Prob > |t| | 0,2712 |
| Lower CL Dif | -8,0976 | Prob > t | 0,8644 |
| Confidence | 0,95 | Prob < t | 0,1356 |

**Analysis of Variance**

| **Source** | **DF** | **Sum of Squares** | **Mean Square** | **F Ratio** | **Prob > F** |
| --- | --- | --- | --- | --- | --- |
| GrG <=2 vs >=3 | 1 | 135,0643 | 135,064 | 1,2320 | 0,2712 |
| Error | 63 | 6906,9209 | 109,634 |  |  |
| C. Total | 64 | 7041,9852 |  |  |  |

**Means for Oneway Anova**

| **Level** | **Number** | **Mean** | **Std Error** | **Lower 95%** | **Upper 95%** |
| --- | --- | --- | --- | --- | --- |
| 1/2 | 30 | 2,93961 | 1,9117 | -0,881 | 6,7598 |
| 3/4&5 | 35 | 0,04805 | 1,7699 | -3,489 | 3,5848 |

Std Error uses a pooled estimate of error variance

**Nonparametric Comparisons For Each Pair Using Wilcoxon Method**

| **q*** | **Alpha** |
| --- | --- |
| 1,95996 | 0,05 |

| **Level** | **- Level** | **Score Mean Difference** | **Std Err Dif** | **Z** | **p-Value** | **Hodges-Lehmann** | **Lower CL** | **Upper CL** | **Difference Plot** |
| --- | --- | --- | --- | --- | --- | --- | --- | --- | --- |
| 3/4&5 | 1/2 | -2,38333 | 4,704355 | -0,506623 | 0,6124 | -0,002391 | -0,019007 | 0,0115912 |  |

**Oneway Analysis of original-glszm-LargeAreaHighGrayLevelEmphasis By GrG <=2 vs >=3**

**Quantiles**

| **Level** | **Minimum** | **10%** | **25%** | **Median** | **75%** | **90%** | **Maximum** |
| --- | --- | --- | --- | --- | --- | --- | --- |
| 1/2 | 252,8421 | 608,2279 | 1016,518 | 1314,188 | 2450,355 | 6572,673 | 6802,799 |
| 3/4&5 | 530 | 764,2042 | 1155,141 | 1643,15 | 3386,467 | 5540,449 | 15257,9 |

**Oneway Anova**

**Summary of Fit**

| Rsquare | 0,010486 |
| --- | --- |
| Adj Rsquare | -0,00522 |
| Root Mean Square Error | 2387,135 |
| Mean of Response | 2428,761 |
| Observations (or Sum Wgts) | 65 |

**Pooled t Test**

3/4&5-1/2

Assuming equal variances

| Difference | 485,3 | t Ratio | 0,817076 |
| --- | --- | --- | --- |
| Std Err Dif | 593,9 | DF | 63 |
| Upper CL Dif | 1672,2 | Prob > |t| | 0,4170 |
| Lower CL Dif | -701,6 | Prob > t | 0,2085 |
| Confidence | 0,95 | Prob < t | 0,7915 |

**Analysis of Variance**

| **Source** | **DF** | **Sum of Squares** | **Mean Square** | **F Ratio** | **Prob > F** |
| --- | --- | --- | --- | --- | --- |
| GrG <=2 vs >=3 | 1 | 3804338 | 3804338 | 0,6676 | 0,4170 |
| Error | 63 | 359000108 | 5698414 |  |  |
| C. Total | 64 | 362804446 |  |  |  |

**Means for Oneway Anova**

| **Level** | **Number** | **Mean** | **Std Error** | **Lower 95%** | **Upper 95%** |
| --- | --- | --- | --- | --- | --- |
| 1/2 | 30 | 2167,45 | 435,83 | 1296,5 | 3038,4 |
| 3/4&5 | 35 | 2652,74 | 403,50 | 1846,4 | 3459,1 |

Std Error uses a pooled estimate of error variance

**Nonparametric Comparisons For Each Pair Using Wilcoxon Method**

| **q*** | **Alpha** |
| --- | --- |
| 1,95996 | 0,05 |

| **Level** | **- Level** | **Score Mean Difference** | **Std Err Dif** | **Z** | **p-Value** | **Hodges-Lehmann** | **Lower CL** | **Upper CL** | **Difference Plot** |
| --- | --- | --- | --- | --- | --- | --- | --- | --- | --- |
| 3/4&5 | 1/2 | 5,292857 | 4,704355 | 1,125097 | 0,2605 | 276,3294 | -303,326 | 867,8338 |  |

**Oneway Analysis of original-glszm-HighGrayLevelZoneEmphasis By GrG <=2 vs >=3**

**Quantiles**

| **Level** | **Minimum** | **10%** | **25%** | **Median** | **75%** | **90%** | **Maximum** |
| --- | --- | --- | --- | --- | --- | --- | --- |
| 1/2 | 7,5 | 156,799 | 237,8782 | 439,5983 | 780,2919 | 1036,968 | 1111,346 |
| 3/4&5 | 142,2778 | 184,5476 | 292,4671 | 407,1272 | 730,0505 | 1213,247 | 2311,652 |

**Oneway Anova**

**Summary of Fit**

| Rsquare | 0,005555 |
| --- | --- |
| Adj Rsquare | -0,01023 |
| Root Mean Square Error | 389,5892 |
| Mean of Response | 544,5016 |
| Observations (or Sum Wgts) | 65 |

**Pooled t Test**

3/4&5-1/2

Assuming equal variances

| Difference | 57,50 | t Ratio | 0,593223 |
| --- | --- | --- | --- |
| Std Err Dif | 96,93 | DF | 63 |
| Upper CL Dif | 251,21 | Prob > |t| | 0,5552 |
| Lower CL Dif | -136,20 | Prob > t | 0,2776 |
| Confidence | 0,95 | Prob < t | 0,7224 |

**Analysis of Variance**

| **Source** | **DF** | **Sum of Squares** | **Mean Square** | **F Ratio** | **Prob > F** |
| --- | --- | --- | --- | --- | --- |
| GrG <=2 vs >=3 | 1 | 53413,4 | 53413 | 0,3519 | 0,5552 |
| Error | 63 | 9562126,4 | 151780 |  |  |
| C. Total | 64 | 9615539,8 |  |  |  |

**Means for Oneway Anova**

| **Level** | **Number** | **Mean** | **Std Error** | **Lower 95%** | **Upper 95%** |
| --- | --- | --- | --- | --- | --- |
| 1/2 | 30 | 513,539 | 71,129 | 371,40 | 655,68 |
| 3/4&5 | 35 | 571,041 | 65,853 | 439,45 | 702,64 |

Std Error uses a pooled estimate of error variance

**Nonparametric Comparisons For Each Pair Using Wilcoxon Method**

| **q*** | **Alpha** |
| --- | --- |
| 1,95996 | 0,05 |

| **Level** | **- Level** | **Score Mean Difference** | **Std Err Dif** | **Z** | **p-Value** | **Hodges-Lehmann** | **Lower CL** | **Upper CL** | **Difference Plot** |
| --- | --- | --- | --- | --- | --- | --- | --- | --- | --- |
| 3/4&5 | 1/2 | 1,145238 | 4,704355 | 0,2434421 | 0,8077 | 21,35490 | -132,328 | 152,3333 |  |

**Oneway Analysis of original-glszm-SmallAreaEmphasis By GrG <=2 vs >=3**

**Quantiles**

| **Level** | **Minimum** | **10%** | **25%** | **Median** | **75%** | **90%** | **Maximum** |
| --- | --- | --- | --- | --- | --- | --- | --- |
| 1/2 | 0,253802 | 0,646009 | 0,691805 | 0,729851 | 0,793764 | 0,840215 | 0,848357 |
| 3/4&5 | 0,652384 | 0,686464 | 0,711528 | 0,731957 | 0,771155 | 0,790688 | 0,849121 |

**Oneway Anova**

**Summary of Fit**

| Rsquare | 0,005523 |
| --- | --- |
| Adj Rsquare | -0,01026 |
| Root Mean Square Error | 0,080501 |
| Mean of Response | 0,733748 |
| Observations (or Sum Wgts) | 65 |

**Pooled t Test**

3/4&5-1/2

Assuming equal variances

| Difference | 0,01185 | t Ratio | 0,591509 |
| --- | --- | --- | --- |
| Std Err Dif | 0,02003 | DF | 63 |
| Upper CL Dif | 0,05187 | Prob > |t| | 0,5563 |
| Lower CL Dif | -0,02818 | Prob > t | 0,2781 |
| Confidence | 0,95 | Prob < t | 0,7219 |

**Analysis of Variance**

| **Source** | **DF** | **Sum of Squares** | **Mean Square** | **F Ratio** | **Prob > F** |
| --- | --- | --- | --- | --- | --- |
| GrG <=2 vs >=3 | 1 | 0,00226741 | 0,002267 | 0,3499 | 0,5563 |
| Error | 63 | 0,40826995 | 0,006480 |  |  |
| C. Total | 64 | 0,41053736 |  |  |  |

**Means for Oneway Anova**

| **Level** | **Number** | **Mean** | **Std Error** | **Lower 95%** | **Upper 95%** |
| --- | --- | --- | --- | --- | --- |
| 1/2 | 30 | 0,727369 | 0,01470 | 0,69800 | 0,75674 |
| 3/4&5 | 35 | 0,739217 | 0,01361 | 0,71202 | 0,76641 |

Std Error uses a pooled estimate of error variance

**Nonparametric Comparisons For Each Pair Using Wilcoxon Method**

| **q*** | **Alpha** |
| --- | --- |
| 1,95996 | 0,05 |

| **Level** | **- Level** | **Score Mean Difference** | **Std Err Dif** | **Z** | **p-Value** | **Hodges-Lehmann** | **Lower CL** | **Upper CL** | **Difference Plot** |
| --- | --- | --- | --- | --- | --- | --- | --- | --- | --- |
| 3/4&5 | 1/2 | 1,454762 | 4,704355 | 0,3092373 | 0,7571 | 0,0037970 | -0,027640 | 0,0299694 |  |

**Oneway Analysis of original-glszm-LowGrayLevelZoneEmphasis By GrG <=2 vs >=3**

**Quantiles**

| **Level** | **Minimum** | **10%** | **25%** | **Median** | **75%** | **90%** | **Maximum** |
| --- | --- | --- | --- | --- | --- | --- | --- |
| 1/2 | 0,002538 | 0,003176 | 0,012865 | 0,022004 | 0,036948 | 0,053682 | 0,355903 |
| 3/4&5 | 0,001198 | 0,003486 | 0,007635 | 0,016566 | 0,029003 | 0,052337 | 0,072304 |

**Oneway Anova**

**Summary of Fit**

| Rsquare | 0,022902 |
| --- | --- |
| Adj Rsquare | 0,007393 |
| Root Mean Square Error | 0,044802 |
| Mean of Response | 0,02758 |
| Observations (or Sum Wgts) | 65 |

**Pooled t Test**

3/4&5-1/2

Assuming equal variances

| Difference | -0,01355 | t Ratio | -1,21517 |
| --- | --- | --- | --- |
| Std Err Dif | 0,01115 | DF | 63 |
| Upper CL Dif | 0,00873 | Prob > |t| | 0,2288 |
| Lower CL Dif | -0,03582 | Prob > t | 0,8856 |
| Confidence | 0,95 | Prob < t | 0,1144 |

**Analysis of Variance**

| **Source** | **DF** | **Sum of Squares** | **Mean Square** | **F Ratio** | **Prob > F** |
| --- | --- | --- | --- | --- | --- |
| GrG <=2 vs >=3 | 1 | 0,00296393 | 0,002964 | 1,4766 | 0,2288 |
| Error | 63 | 0,12645365 | 0,002007 |  |  |
| C. Total | 64 | 0,12941758 |  |  |  |

**Means for Oneway Anova**

| **Level** | **Number** | **Mean** | **Std Error** | **Lower 95%** | **Upper 95%** |
| --- | --- | --- | --- | --- | --- |
| 1/2 | 30 | 0,034874 | 0,00818 | 0,01853 | 0,05122 |
| 3/4&5 | 35 | 0,021329 | 0,00757 | 0,00620 | 0,03646 |

Std Error uses a pooled estimate of error variance

**Nonparametric Comparisons For Each Pair Using Wilcoxon Method**

| **q*** | **Alpha** |
| --- | --- |
| 1,95996 | 0,05 |

| **Level** | **- Level** | **Score Mean Difference** | **Std Err Dif** | **Z** | **p-Value** | **Hodges-Lehmann** | **Lower CL** | **Upper CL** | **Difference Plot** |
| --- | --- | --- | --- | --- | --- | --- | --- | --- | --- |
| 3/4&5 | 1/2 | -5,16905 | 4,704355 | -1,09878 | 0,2719 | -0,004669 | -0,012408 | 0,0027803 |  |

**Oneway Analysis of original-glszm-ZoneEntropy By GrG <=2 vs >=3**

**Quantiles**

| **Level** | **Minimum** | **10%** | **25%** | **Median** | **75%** | **90%** | **Maximum** |
| --- | --- | --- | --- | --- | --- | --- | --- |
| 1/2 | 2 | 4,205759 | 4,930171 | 5,515503 | 5,815472 | 6,485413 | 6,78314 |
| 3/4&5 | 3,921928 | 4,752753 | 5,123231 | 5,841707 | 6,255582 | 6,731137 | 7,305317 |

**Oneway Anova**

**Summary of Fit**

| Rsquare | 0,057223 |
| --- | --- |
| Adj Rsquare | 0,042259 |
| Root Mean Square Error | 0,84466 |
| Mean of Response | 5,562973 |
| Observations (or Sum Wgts) | 65 |

**Pooled t Test**

3/4&5-1/2

Assuming equal variances

| Difference | 0,41096 | t Ratio | 1,955474 |
| --- | --- | --- | --- |
| Std Err Dif | 0,21016 | DF | 63 |
| Upper CL Dif | 0,83092 | Prob > |t| | 0,0550 |
| Lower CL Dif | -0,00901 | Prob > t | 0,0275* |
| Confidence | 0,95 | Prob < t | 0,9725 |

**Analysis of Variance**

| **Source** | **DF** | **Sum of Squares** | **Mean Square** | **F Ratio** | **Prob > F** |
| --- | --- | --- | --- | --- | --- |
| GrG <=2 vs >=3 | 1 | 2,728150 | 2,72815 | 3,8239 | 0,0550 |
| Error | 63 | 44,947408 | 0,71345 |  |  |
| C. Total | 64 | 47,675558 |  |  |  |

**Means for Oneway Anova**

| **Level** | **Number** | **Mean** | **Std Error** | **Lower 95%** | **Upper 95%** |
| --- | --- | --- | --- | --- | --- |
| 1/2 | 30 | 5,34169 | 0,15421 | 5,0335 | 5,6499 |
| 3/4&5 | 35 | 5,75264 | 0,14277 | 5,4673 | 6,0380 |

Std Error uses a pooled estimate of error variance

**Nonparametric Comparisons For Each Pair Using Wilcoxon Method**

| **q*** | **Alpha** |
| --- | --- |
| 1,95996 | 0,05 |

| **Level** | **- Level** | **Score Mean Difference** | **Std Err Dif** | **Z** | **p-Value** | **Hodges-Lehmann** | **Lower CL** | **Upper CL** | **Difference Plot** |
| --- | --- | --- | --- | --- | --- | --- | --- | --- | --- |
| 3/4&5 | 1/2 | 8,945238 | 4,704355 | 1,901480 | 0,0572 | 0,3648548 | -0,013277 | 0,7452783 |  |

**Oneway Analysis of original-glszm-SmallAreaLowGrayLevelEmphasis By GrG <=2 vs >=3**

**Quantiles**

| **Level** | **Minimum** | **10%** | **25%** | **Median** | **75%** | **90%** | **Maximum** |
| --- | --- | --- | --- | --- | --- | --- | --- |
| 1/2 | 0,002079 | 0,002822 | 0,012222 | 0,018297 | 0,02378 | 0,041253 | 0,063237 |
| 3/4&5 | 0,001018 | 0,002931 | 0,007232 | 0,013607 | 0,025802 | 0,040655 | 0,071915 |

**Oneway Anova**

**Summary of Fit**

| Rsquare | 0,002762 |
| --- | --- |
| Adj Rsquare | -0,01307 |
| Root Mean Square Error | 0,015671 |
| Mean of Response | 0,019093 |
| Observations (or Sum Wgts) | 65 |

**Pooled t Test**

3/4&5-1/2

Assuming equal variances

| Difference | -0,00163 | t Ratio | -0,41772 |
| --- | --- | --- | --- |
| Std Err Dif | 0,00390 | DF | 63 |
| Upper CL Dif | 0,00616 | Prob > |t| | 0,6776 |
| Lower CL Dif | -0,00942 | Prob > t | 0,6612 |
| Confidence | 0,95 | Prob < t | 0,3388 |

**Analysis of Variance**

| **Source** | **DF** | **Sum of Squares** | **Mean Square** | **F Ratio** | **Prob > F** |
| --- | --- | --- | --- | --- | --- |
| GrG <=2 vs >=3 | 1 | 0,00004285 | 0,000043 | 0,1745 | 0,6776 |
| Error | 63 | 0,01547207 | 0,000246 |  |  |
| C. Total | 64 | 0,01551492 |  |  |  |

**Means for Oneway Anova**

| **Level** | **Number** | **Mean** | **Std Error** | **Lower 95%** | **Upper 95%** |
| --- | --- | --- | --- | --- | --- |
| 1/2 | 30 | 0,019971 | 0,00286 | 0,01425 | 0,02569 |
| 3/4&5 | 35 | 0,018342 | 0,00265 | 0,01305 | 0,02364 |

Std Error uses a pooled estimate of error variance

**Nonparametric Comparisons For Each Pair Using Wilcoxon Method**

| **q*** | **Alpha** |
| --- | --- |
| 1,95996 | 0,05 |

| **Level** | **- Level** | **Score Mean Difference** | **Std Err Dif** | **Z** | **p-Value** | **Hodges-Lehmann** | **Lower CL** | **Upper CL** | **Difference Plot** |
| --- | --- | --- | --- | --- | --- | --- | --- | --- | --- |
| 3/4&5 | 1/2 | -5,10714 | 4,704355 | -1,08562 | 0,2776 | -0,003284 | -0,009128 | 0,0027833 |  |

**Oneway Analysis of original-ngtdm-Coarseness By GrG <=2 vs >=3**

**Quantiles**

| **Level** | **Minimum** | **10%** | **25%** | **Median** | **75%** | **90%** | **Maximum** |
| --- | --- | --- | --- | --- | --- | --- | --- |
| 1/2 | 0,002607 | 0,006212 | 0,021887 | 0,034586 | 0,055211 | 0,083277 | 0,14397 |
| 3/4&5 | 0,00052 | 0,002524 | 0,012291 | 0,02236 | 0,045531 | 0,062973 | 0,118322 |

**Oneway Anova**

**Summary of Fit**

| Rsquare | 0,043584 |
| --- | --- |
| Adj Rsquare | 0,028403 |
| Root Mean Square Error | 0,028714 |
| Mean of Response | 0,035732 |
| Observations (or Sum Wgts) | 65 |

**Pooled t Test**

3/4&5-1/2

Assuming equal variances

| Difference | -0,01211 | t Ratio | -1,69439 |
| --- | --- | --- | --- |
| Std Err Dif | 0,00714 | DF | 63 |
| Upper CL Dif | 0,00217 | Prob > |t| | 0,0951 |
| Lower CL Dif | -0,02638 | Prob > t | 0,9524 |
| Confidence | 0,95 | Prob < t | 0,0476* |

**Analysis of Variance**

| **Source** | **DF** | **Sum of Squares** | **Mean Square** | **F Ratio** | **Prob > F** |
| --- | --- | --- | --- | --- | --- |
| GrG <=2 vs >=3 | 1 | 0,00236709 | 0,002367 | 2,8709 | 0,0951 |
| Error | 63 | 0,05194342 | 0,000824 |  |  |
| C. Total | 64 | 0,05431052 |  |  |  |

**Means for Oneway Anova**

| **Level** | **Number** | **Mean** | **Std Error** | **Lower 95%** | **Upper 95%** |
| --- | --- | --- | --- | --- | --- |
| 1/2 | 30 | 0,042250 | 0,00524 | 0,03177 | 0,05273 |
| 3/4&5 | 35 | 0,030145 | 0,00485 | 0,02045 | 0,03984 |

Std Error uses a pooled estimate of error variance

**Nonparametric Comparisons For Each Pair Using Wilcoxon Method**

| **q*** | **Alpha** |
| --- | --- |
| 1,95996 | 0,05 |

| **Level** | **- Level** | **Score Mean Difference** | **Std Err Dif** | **Z** | **p-Value** | **Hodges-Lehmann** | **Lower CL** | **Upper CL** | **Difference Plot** |
| --- | --- | --- | --- | --- | --- | --- | --- | --- | --- |
| 3/4&5 | 1/2 | -9,99762 | 4,704355 | -2,12518 | 0,0336* | -0,012551 | -0,024937 | -0,000959 |  |

**Oneway Analysis of original-ngtdm-Complexity By GrG <=2 vs >=3**

**Quantiles**

| **Level** | **Minimum** | **10%** | **25%** | **Median** | **75%** | **90%** | **Maximum** |
| --- | --- | --- | --- | --- | --- | --- | --- |
| 1/2 | 3,066231 | 667,7785 | 1045,968 | 1721,917 | 3143,214 | 5429,315 | 7839,335 |
| 3/4&5 | 378,8591 | 599,8312 | 1141,159 | 1781,508 | 4201,19 | 9339,308 | 21094,04 |

**Oneway Anova**

**Summary of Fit**

| Rsquare | 0,03531 |
| --- | --- |
| Adj Rsquare | 0,019998 |
| Root Mean Square Error | 3252,189 |
| Mean of Response | 2978,754 |
| Observations (or Sum Wgts) | 65 |

**Pooled t Test**

3/4&5-1/2

Assuming equal variances

| Difference | 1228,8 | t Ratio | 1,51854 |
| --- | --- | --- | --- |
| Std Err Dif | 809,2 | DF | 63 |
| Upper CL Dif | 2845,7 | Prob > |t| | 0,1339 |
| Lower CL Dif | -388,2 | Prob > t | 0,0669 |
| Confidence | 0,95 | Prob < t | 0,9331 |

**Analysis of Variance**

| **Source** | **DF** | **Sum of Squares** | **Mean Square** | **F Ratio** | **Prob > F** |
| --- | --- | --- | --- | --- | --- |
| GrG <=2 vs >=3 | 1 | 24389550 | 24389550 | 2,3060 | 0,1339 |
| Error | 63 | 666334179 | 10576733 |  |  |
| C. Total | 64 | 690723729 |  |  |  |

**Means for Oneway Anova**

| **Level** | **Number** | **Mean** | **Std Error** | **Lower 95%** | **Upper 95%** |
| --- | --- | --- | --- | --- | --- |
| 1/2 | 30 | 2317,12 | 593,77 | 1130,6 | 3503,7 |
| 3/4&5 | 35 | 3545,87 | 549,72 | 2447,3 | 4644,4 |

Std Error uses a pooled estimate of error variance

**Nonparametric Comparisons For Each Pair Using Wilcoxon Method**

| **q*** | **Alpha** |
| --- | --- |
| 1,95996 | 0,05 |

| **Level** | **- Level** | **Score Mean Difference** | **Std Err Dif** | **Z** | **p-Value** | **Hodges-Lehmann** | **Lower CL** | **Upper CL** | **Difference Plot** |
| --- | --- | --- | --- | --- | --- | --- | --- | --- | --- |
| 3/4&5 | 1/2 | 4,735714 | 4,704355 | 1,006666 | 0,3141 | 399,1174 | -374,840 | 1324,016 |  |

**Oneway Analysis of original-ngtdm-Strength By GrG <=2 vs >=3**

**Quantiles**

| **Level** | **Minimum** | **10%** | **25%** | **Median** | **75%** | **90%** | **Maximum** |
| --- | --- | --- | --- | --- | --- | --- | --- |
| 1/2 | 0,789187 | 4,072181 | 7,782118 | 11,27 | 23,48803 | 29,1275 | 35,99932 |
| 3/4&5 | 1,958236 | 3,048428 | 5,808348 | 9,070206 | 18,48432 | 25,32298 | 31,46339 |

**Oneway Anova**

**Summary of Fit**

| Rsquare | 0,038612 |
| --- | --- |
| Adj Rsquare | 0,023352 |
| Root Mean Square Error | 8,707416 |
| Mean of Response | 13,09461 |
| Observations (or Sum Wgts) | 65 |

**Pooled t Test**

3/4&5-1/2

Assuming equal variances

| Difference | -3,4461 | t Ratio | -1,59067 |
| --- | --- | --- | --- |
| Std Err Dif | 2,1665 | DF | 63 |
| Upper CL Dif | 0,8832 | Prob > |t| | 0,1167 |
| Lower CL Dif | -7,7755 | Prob > t | 0,9417 |
| Confidence | 0,95 | Prob < t | 0,0583 |

**Analysis of Variance**

| **Source** | **DF** | **Sum of Squares** | **Mean Square** | **F Ratio** | **Prob > F** |
| --- | --- | --- | --- | --- | --- |
| GrG <=2 vs >=3 | 1 | 191,8403 | 191,840 | 2,5302 | 0,1167 |
| Error | 63 | 4776,6026 | 75,819 |  |  |
| C. Total | 64 | 4968,4429 |  |  |  |

**Means for Oneway Anova**

| **Level** | **Number** | **Mean** | **Std Error** | **Lower 95%** | **Upper 95%** |
| --- | --- | --- | --- | --- | --- |
| 1/2 | 30 | 14,9502 | 1,5897 | 11,773 | 18,127 |
| 3/4&5 | 35 | 11,5041 | 1,4718 | 8,563 | 14,445 |

Std Error uses a pooled estimate of error variance

**Nonparametric Comparisons For Each Pair Using Wilcoxon Method**

| **q*** | **Alpha** |
| --- | --- |
| 1,95996 | 0,05 |

| **Level** | **- Level** | **Score Mean Difference** | **Std Err Dif** | **Z** | **p-Value** | **Hodges-Lehmann** | **Lower CL** | **Upper CL** | **Difference Plot** |
| --- | --- | --- | --- | --- | --- | --- | --- | --- | --- |
| 3/4&5 | 1/2 | -7,27381 | 4,704355 | -1,54619 | 0,1221 | -2,85227 | -6,89461 | 1,089203 |  |

**Oneway Analysis of original-ngtdm-Contrast By GrG <=2 vs >=3**

**Quantiles**

| **Level** | **Minimum** | **10%** | **25%** | **Median** | **75%** | **90%** | **Maximum** |
| --- | --- | --- | --- | --- | --- | --- | --- |
| 1/2 | 0,049897 | 0,187593 | 0,288336 | 0,542729 | 0,805582 | 1,146767 | 2,290251 |
| 3/4&5 | 0,150017 | 0,200788 | 0,260268 | 0,407301 | 0,604029 | 0,828889 | 2,086431 |

**Oneway Anova**

**Summary of Fit**

| Rsquare | 0,025477 |
| --- | --- |
| Adj Rsquare | 0,010009 |
| Root Mean Square Error | 0,43378 |
| Mean of Response | 0,563537 |
| Observations (or Sum Wgts) | 65 |

**Pooled t Test**

3/4&5-1/2

Assuming equal variances

| Difference | -0,13851 | t Ratio | -1,28337 |
| --- | --- | --- | --- |
| Std Err Dif | 0,10793 | DF | 63 |
| Upper CL Dif | 0,07716 | Prob > |t| | 0,2041 |
| Lower CL Dif | -0,35419 | Prob > t | 0,8980 |
| Confidence | 0,95 | Prob < t | 0,1020 |

**Analysis of Variance**

| **Source** | **DF** | **Sum of Squares** | **Mean Square** | **F Ratio** | **Prob > F** |
| --- | --- | --- | --- | --- | --- |
| GrG <=2 vs >=3 | 1 | 0,309916 | 0,309916 | 1,6470 | 0,2041 |
| Error | 63 | 11,854412 | 0,188165 |  |  |
| C. Total | 64 | 12,164328 |  |  |  |

**Means for Oneway Anova**

| **Level** | **Number** | **Mean** | **Std Error** | **Lower 95%** | **Upper 95%** |
| --- | --- | --- | --- | --- | --- |
| 1/2 | 30 | 0,638120 | 0,07920 | 0,47986 | 0,79638 |
| 3/4&5 | 35 | 0,499609 | 0,07332 | 0,35309 | 0,64613 |

Std Error uses a pooled estimate of error variance

**Nonparametric Comparisons For Each Pair Using Wilcoxon Method**

| **q*** | **Alpha** |
| --- | --- |
| 1,95996 | 0,05 |

| **Level** | **- Level** | **Score Mean Difference** | **Std Err Dif** | **Z** | **p-Value** | **Hodges-Lehmann** | **Lower CL** | **Upper CL** | **Difference Plot** |
| --- | --- | --- | --- | --- | --- | --- | --- | --- | --- |
| 3/4&5 | 1/2 | -8,01667 | 4,704355 | -1,70409 | 0,0884 | -0,112160 | -0,299124 | 0,0107266 |  |

**Oneway Analysis of original-ngtdm-Busyness By GrG <=2 vs >=3**

**Quantiles**

| **Level** | **Minimum** | **10%** | **25%** | **Median** | **75%** | **90%** | **Maximum** |
| --- | --- | --- | --- | --- | --- | --- | --- |
| 1/2 | 0,023412 | 0,026435 | 0,03958 | 0,075571 | 0,098574 | 0,157046 | 1,295467 |
| 3/4&5 | 0,033325 | 0,037162 | 0,053519 | 0,082431 | 0,130214 | 0,264611 | 0,421662 |

**Oneway Anova**

**Summary of Fit**

| Rsquare | 6,852e-5 |
| --- | --- |
| Adj Rsquare | -0,0158 |
| Root Mean Square Error | 0,168827 |
| Mean of Response | 0,113548 |
| Observations (or Sum Wgts) | 65 |

**Pooled t Test**

3/4&5-1/2

Assuming equal variances

| Difference | -0,00276 | t Ratio | -0,06571 |
| --- | --- | --- | --- |
| Std Err Dif | 0,04201 | DF | 63 |
| Upper CL Dif | 0,08118 | Prob > |t| | 0,9478 |
| Lower CL Dif | -0,08670 | Prob > t | 0,5261 |
| Confidence | 0,95 | Prob < t | 0,4739 |

**Analysis of Variance**

| **Source** | **DF** | **Sum of Squares** | **Mean Square** | **F Ratio** | **Prob > F** |
| --- | --- | --- | --- | --- | --- |
| GrG <=2 vs >=3 | 1 | 0,0001231 | 0,000123 | 0,0043 | 0,9478 |
| Error | 63 | 1,7956520 | 0,028502 |  |  |
| C. Total | 64 | 1,7957750 |  |  |  |

**Means for Oneway Anova**

| **Level** | **Number** | **Mean** | **Std Error** | **Lower 95%** | **Upper 95%** |
| --- | --- | --- | --- | --- | --- |
| 1/2 | 30 | 0,115034 | 0,03082 | 0,05344 | 0,17663 |
| 3/4&5 | 35 | 0,112274 | 0,02854 | 0,05525 | 0,16930 |

Std Error uses a pooled estimate of error variance

**Nonparametric Comparisons For Each Pair Using Wilcoxon Method**

| **q*** | **Alpha** |
| --- | --- |
| 1,95996 | 0,05 |

| **Level** | **- Level** | **Score Mean Difference** | **Std Err Dif** | **Z** | **p-Value** | **Hodges-Lehmann** | **Lower CL** | **Upper CL** | **Difference Plot** |
| --- | --- | --- | --- | --- | --- | --- | --- | --- | --- |
| 3/4&5 | 1/2 | 5,354762 | 4,704355 | 1,138256 | 0,2550 | 0,0116116 | -0,009910 | 0,0361752 |  |
